# Asymmetric Synthesis of 2‐Arylindolines and 2,2‐Disubstituted Indolines by Kinetic Resolution

**DOI:** 10.1002/chem.202101248

**Published:** 2021-07-08

**Authors:** Anthony Choi, Ashraf El‐Tunsi, Yuhang Wang, Anthony J. H. M. Meijer, Jia Li, Xiabing Li, Ilaria Proietti Silvestri, Iain Coldham

**Affiliations:** ^1^ Department of Chemistry University of Sheffield Brook Hill Sheffield S3 7HF UK; ^2^ School of Chemistry and Chemical Engineering Shaanxi Normal University Xi'an 710062 PR China; ^3^ Liverpool ChiroChem Department of Chemistry University of Liverpool Liverpool L69 7ZD UK

**Keywords:** asymmetric synthesis, enantioselectivity, heterocycles, kinetic resolution, lithiation

## Abstract

Kinetic resolution of 2‐arylindolines (2,3‐dihydroindoles) was achieved by treatment of their *N*‐*tert*‐butoxycarbonyl (Boc) derivatives with *n*‐butyllithium and sparteine in toluene at −78 °C followed by electrophilic quench. The unreacted starting materials together with the 2,2‐disubstituted products could be isolated with high enantiomer ratios. Variable temperature NMR spectroscopy showed that the rate of Boc rotation was fast (Δ*G* ^≠^≈57 kJ/mol at 195 K). This was corroborated by DFT studies and by in situ ReactIR spectroscopy. The enantioenriched *N*‐Boc‐2‐arylindolines were converted to 2,2‐disubstituted products without significant loss in enantiopurity. Hence, either enantiomer of the 2,2‐disubstituted products could be obtained with high selectivity from the same enantiomer of the chiral ligand sparteine (one from the kinetic resolution and the other from subsequent lithiation‐trapping of the recovered starting material). Secondary amine products were prepared by removing the Boc group with acid to provide a way to access highly enantioenriched 2‐aryl and 2,2‐disubstituted indolines.

## Graphical abstract

**Asymmetric synthesis** of 2‐arylindolines was achieved by kinetic resolution with *n*‐butyllithium and the chiral ligand sparteine followed by electrophilic quench. The recovered starting materials and the 2,2‐disubstituted products were isolated with high enantiomer ratios. Variable temperature NMR spectroscopy and DFT studies were used to determine the rate of Boc rotation.

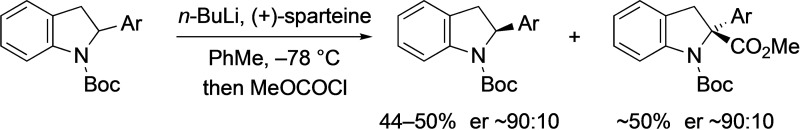

## Introduction

Enantioselective kinetic resolution reactions have shown promise in organolithium chemistry for the preparation of a variety of chiral compounds.^[1]^ Recently, we reported the lithiation and highly selective kinetic resolution of 2‐aryl‐1,2,3,4‐tetrahydroquinolines,^[2]^ building on earlier work with 2‐arylpiperidines.^[3]^ This chemistry makes use of the ability of *n*‐butyllithium coordinated to the chiral ligand sparteine (sp) to differentiate between enantiotopic protons alpha to a *N*‐*tert*‐butoxycarbonyl (*N*‐Boc) group.^[4]^ These results indicate that kinetic resolution using asymmetric lithiation is amenable to a selection of 6‐membered saturated 2‐aryl nitrogen heterocycles. As far as we are aware and despite the importance and prevalence of pyrrolidines in drugs and natural products, the chemistry has not been reported with 5‐membered saturated nitrogen heterocycles. There is an issue with using *N*‐Boc‐2‐arylpyrrolidines for lithiation chemistry in that the rate of rotation of the Boc group is slow.^[5]^ Hence, temperatures that are typical for highly selective lithiation (−78 °C) would be ineffective since only the minor rotamer would undergo lithiation with *n*‐BuLi/sp and the use of higher temperatures may lead to lower selectivities.^[5]^ However, we reasoned that the related indoline ring system, in which the pyrrolidine is fused to a benzene ring, should have a faster rate of Boc rotation due to conjugation of the nitrogen lone pair with the aromatic ring. We therefore set out to determine the kinetics for rotation in this system and its feasibility to conduct the desired kinetic resolution.

**Scheme 1:**
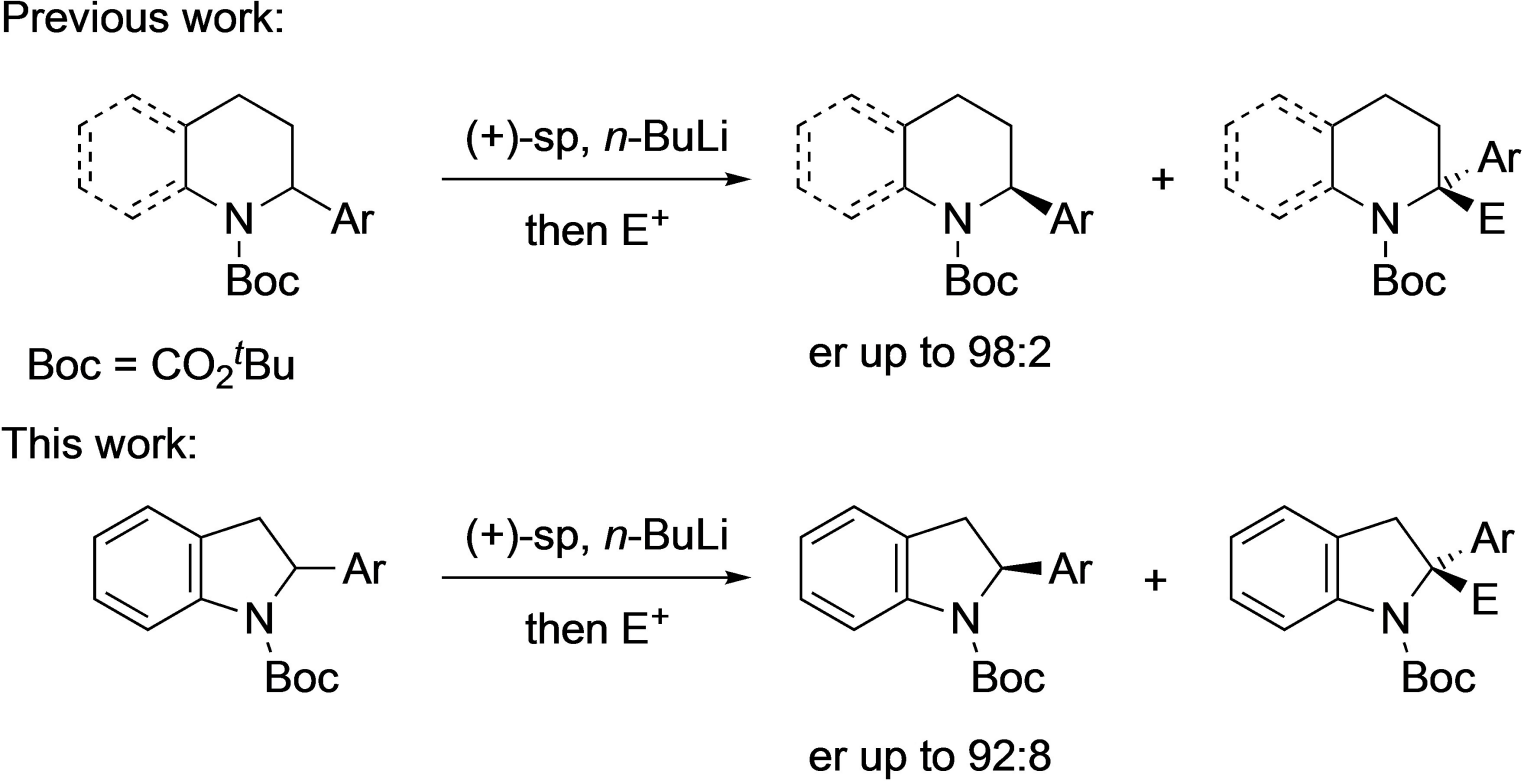
Previous work,[^2^, ^3^] and this work.

Kinetic resolution has been applied to the synthesis of enantiomerically enriched 2‐substituted indolines by using chemoenzymatic methods,^[6]^ acylation or other reaction at the secondary amine with a chiral reagent or catalyst,^[7]^ or oxidation in the presence of a chiral phosphoric acid.^[8]^ Here we report that deprotonation with a chiral base system and trapping with an electrophile provides a highly selective method to give 2‐arylindolines and 2,2‐disubstituted indolines (Scheme 1). The organolithium is configurationally stable at −78 °C and can be trapped to give 2,2‐disubstituted products with excellent enantioselectivities. The presence of the indoline unit in many natural products and biologically active molecules (e. g. vindoline, ajmaline, physostigmine), together with the relatively unexplored enantiomerically enriched 2,2‐disubstituted derivatives with potential bioactivity,^[9]^ makes new methods to access these structures of particular interest.

## Results and Discussion

**Scheme 2:**
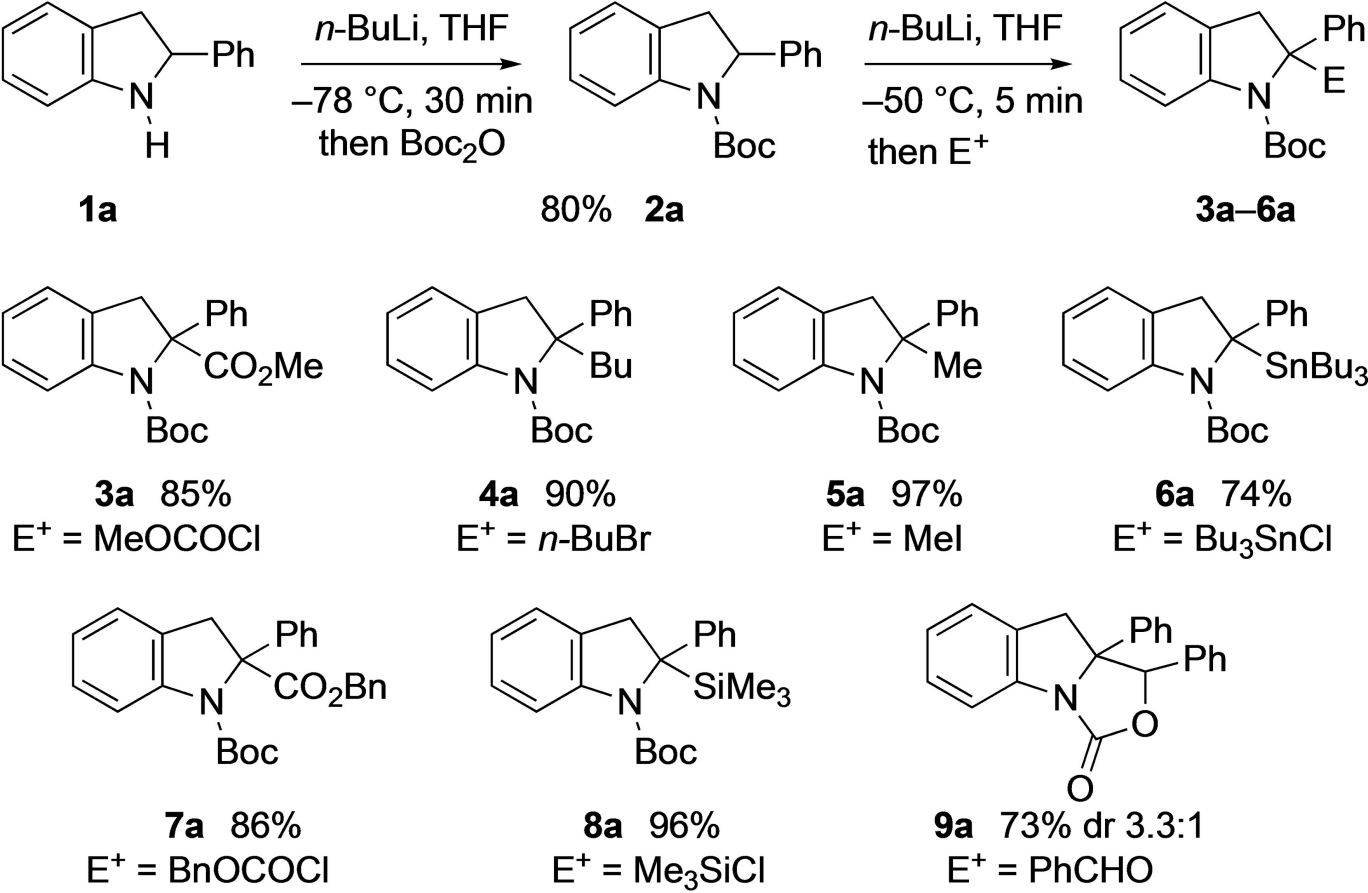
Initial lithiation‐quench studies.

2‐Phenylindoline **1** **a** was prepared from 2‐phenylindole by reduction, according to the literature.[^10^, ^11^] Protection of the nitrogen atom with a Boc group was achieved by deprotonation and addition of Boc_2_O to give the desired indoline **2** **a** (Scheme 1). Lithiation studies were carried out and it was found that suitable conditions involved treating the indoline **2** **a** with *n*‐BuLi in THF at −50 °C for a few minutes followed by addition of an electrophile. This resulted in high yields of the 2,2‐disubstituted products **3** **a**–**6** **a** (Scheme 2). The chemistry was amenable to a selection of different electrophiles including chloroformates (or MeOCOCN)^[12]^ (to give **3** **a** and **7** **a**), alkyl halides (to give **4** **a** and **5** **a**), tributyltin or trimethylsilyl chloride (to give **6** **a** and **8** **a**) or an aldehyde (to give **9** **a**).

**Figure 1:**
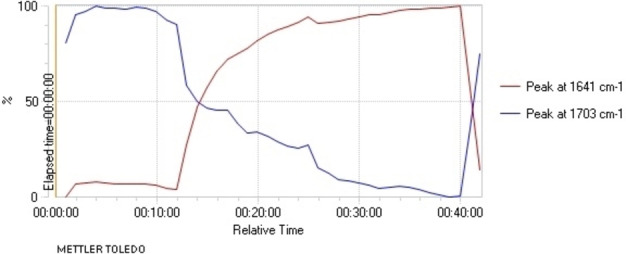
In situ IR spectroscopy of the deprotonation of indoline **2** **a** with *n*‐BuLi, THF at −78 °C; time in h:min:sec; *n*‐BuLi added at time 12 min (*ν*_c=o_ **2** **a** 1703 cm^−1^, *ν*_c=o_ lithiated **2** **a** 1641 cm^−1^); MeOH added at time 40 min.

If the lithiation was carried out at −78 °C for a few minutes followed by electrophilic quench, lower yields of the products were obtained (e. g. **3** **a**, 29 % yield). However, leaving the lithiation for 20 min at −78 °C allowed good yields of the products to be obtained (e. g. **3** **a**, 88 % yield). The lithiation can be monitored by in situ IR spectroscopy (Figure 1).^[13]^ In the IR spectrum, the carbonyl stretching frequency of carbamate **2** **a** at *ν*_c=o_=1703 cm^−1^ is converted gradually to a new stretch at 1641 cm^−1^ on addition of *n*‐BuLi; at −78 °C this requires approximately 20 min for completion.

**Scheme 3:**
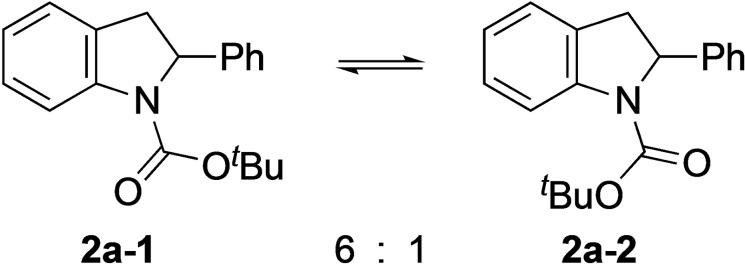
Ratio of rotamers of *N*‐Boc‐2‐phenylindoline **2** **a**.

**Figure 2:**
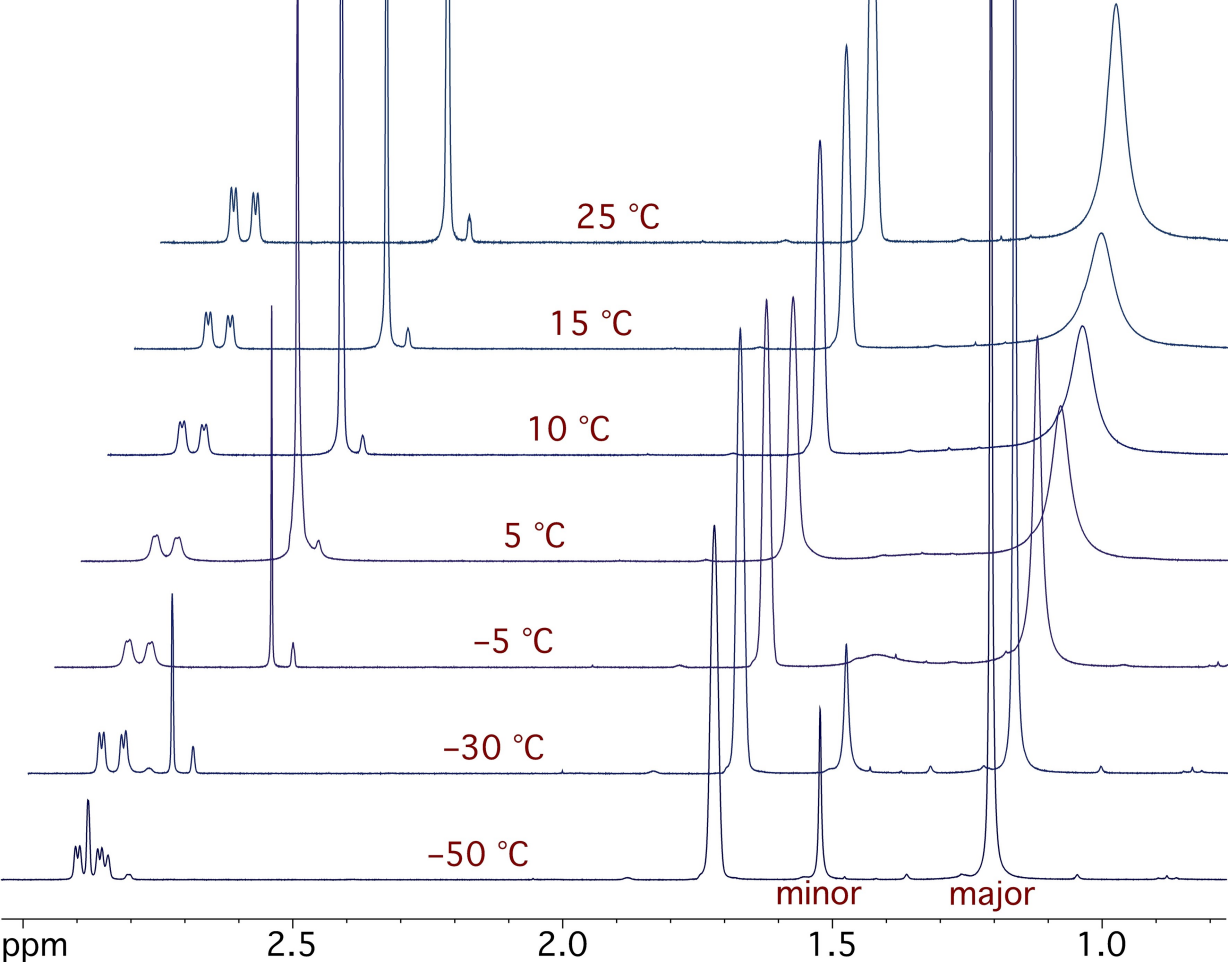
VT NMR spectroscopy of indoline **2** **a** in D_8_‐THF; peak at 1.72 ppm for THF, peaks at 1.52 ppm and 1.21 ppm for *tert*‐butyl of each rotamer.

These results indicate that the Boc group rotates slowly in comparison with lithiation at −78 °C. For lithiation to occur, the carbonyl oxygen atom that coordinates to *n*‐BuLi needs to be located on the side of the benzylic proton.[^14^, ^15^] To quantify the ratio of rotamers and the rate of rotation of the Boc group, we carried out variable temperature (VT) NMR spectroscopic studies (Scheme 3, Figure 2). The ^1^H NMR spectrum showed that the ratio of rotamers was about 6 : 1. This is likely to be in favor of the undesired rotamer **2** **a**–**1**, since lithiation is slow at low temperature (rotamer **2** **a**–**2** is needed for lithiation at C‐2 due to a complex‐induced proximity effect with the *n*‐BuLi coordinating to the carbonyl oxygen atom^[16]^). From line shape analysis and dynamic NMR studies (see Supporting Information) the activation parameters for Boc rotation could be estimated as Δ*H* ^≠^≈53.4 kJ/mol and Δ*S* ^≠^≈−21.5 J/K/mol for the major rotamer converting to the minor rotamer (and Δ*H* ^≠^≈53.4 kJ/mol and Δ*S* ^≠^≈−6.6 J/K/mol for the minor rotamer converting to the major rotamer). This equates to a Gibbs energy of activation for the major to minor rotamer of Δ*G* ^≠^ ≈ 57.6 kJ/mol at −78 °C and hence a half‐life for rotation *t*_1/2_≈7.5 min. The corresponding Gibbs energy of activation for the minor to major rotamer conversion is Δ*G* ^≠^≈54.7 kJ/mol at −78 °C. Thus, the Gibbs energy difference for the two rotamers is Δ*G*≈2.9 kJ/mol at −78 °C, leading to a 1:0.17 ratio at equilibrium in accordance with the ^1^H NMR spectrum. These values correspond well to the data from the in situ IR spectroscopy and from the experimental studies, verifying that a reaction time of about 20 min at −78 °C is required for high yields.

The reason for the preference for rotamer **2** **a**–**1** is not immediately obvious. Any possible steric interaction of the *tert*‐butoxy group with the phenyl group at C‐2 could be outweighed by favourable electrostatic or dispersion interactions, for example between the carbonyl oxygen atom and the proton at C‐7 or between a proton of the *tert*‐butyl group and the aromatic π‐system of the 2‐Ph group.[^5^, ^17^] Therefore, we performed density functional theory (DFT) calculations to elucidate this further, focusing on the energetics of the interconversion in the first instance. Previously,^[5]^ we used the B3LYP functional^[18]^ with the 6‐311G** basis set (B3LYP//6‐311G**).^[19]^ However, the potential dispersion interactions of the Boc group with the 2‐Ph group led us to use a different approach with the B3LYP‐D3BJ functional, as done previously,^[2]^ which includes such interactions (B3LYP‐D3BJ//6‐311G**).^[20]^ In addition, we improved the basis set to def2‐TZVP (B3LYP‐D3BJ//def2‐TZVP).^[21]^ Results for other approaches are discussed in the Supporting Information. In all cases, rotamer **2** **a**–**1** is the lowest energy rotamer. For B3LYP‐D3BJ//def2‐TZVP we obtained a Gibbs energy difference between the rotamers of 3.0 kJ mol^−1^ with an activation free energy of 57.5 kJ mol^−1^ (at 195 K) in very good agreement with the experimentally obtained values. The calculated values for the activation enthalpy for rotamer conversion from the minor to the major rotamer and the associated activation entropy are 55.7 kJ mol^−1^ and −9.5 J K^−1^ mol^−1^, respectively, in reasonable agreement with experiment.

**Figure 3:**
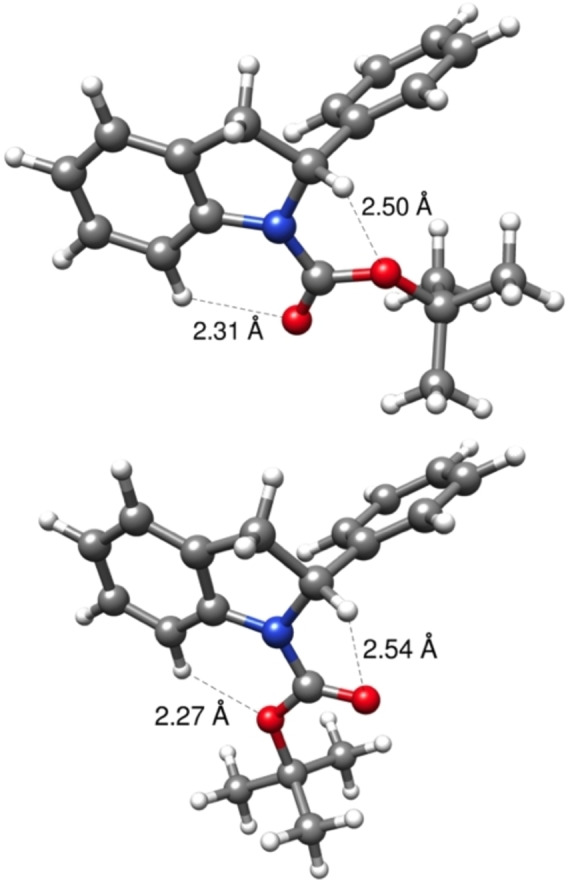
Rotamers **2** **a‐1** and **2** **a‐2** with selected interatomic distances.

Given our results for the energetics of the interconversion between rotamers, the structures of the rotamers **2** **a**–**1** and **2** **a**–**2** were considered more closely using the B3LYP‐D3BJ//def2‐TZVP approach (Figure 3a and 3b respectively). Figure 3 makes clear that there is no clash between the C–H moiety of the *tert*‐butyl group in rotamer **2** **a**–**1** and the phenyl group in the 2‐position, which are at least 3.5 Å from each other. Instead, the slight preference for this (undesired) rotamer is more likely caused by electronic effects. In **2** **a**–**1** the Boc group is co‐planar with the indoline ring, whereas in **2** **a**–**2** it is 9° out of that plane, which suggests a slight loss of conjugation for the **2** **a**–**2** rotamer. It is noted that the C=O…H−C van der Waals bond is shorter for **2** **a**–**1** than for **2** **a**–**2** by 0.2 Å. The electronic energy difference between the two rotamers is 5 kJ mol^−1^, whereas the difference in the D3BJ energies is 7 kJ mol^−1^, showing that electronic energy difference is not only caused by attractive interactions. Together, these geometric changes are apparently sufficient to lead to a *free* energy difference of about 3 kJ mol^−1^ in favor of **2** **a‐1**.

**Scheme 4:**
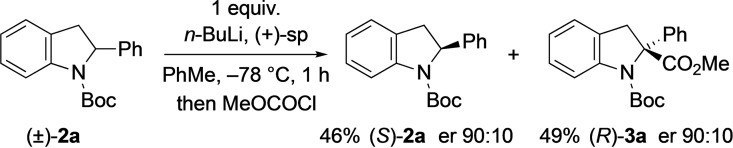
Kinetic resolution of indoline **2** **a**.

Initially, the kinetic resolution of the indoline **2** **a** was carried out at −50 °C using (+)‐sparteine as the chiral ligand (1.3 equiv.) and adding *n*‐BuLi (1 equiv.) to this mixture in PhMe.^[3]^ After just 10 min, there was a high conversion to the product **3** **a** (93 % yield, ∼racemic), with only a small amount of the recovered indoline **2** **a** (5 % yield) for which the enantiomer ratio (er) was moderate (er 82 : 18) but the low yield equates to a selectivity factor S (*k*_rel_) of only about 2.^[22]^ Therefore the reaction was studied at −78 °C and improved yields and er values of recovered indoline **2** **a** were obtained by this method. Optimisation of the conditions led to best results on adding 0.6–1.0 equiv. of *n*‐BuLi to a mixture containing the indoline and 1.0 equiv. (+)‐sparteine for 1 h before adding the electrophile MeOCOCl (Scheme 4). Under these conditions, the recovered indoline (*S*)‐**2** **a** was isolated in 46 % yield (0.1–0.5 g scale) with good enantiomer ratio (er 90 : 10) together with the substituted product **3** **a** with similar er. This equates to a selectivity factor S (*k*_rel_)≈13.

The absolute configuration of the recovered indoline (*S*)‐**2** **a** was determined by single crystal X‐ray analysis (see Supporting Information). This demonstrated that the (*S*) enantiomer had been recovered, as expected based on the reactivity of sparteine in related reactions.^[23]^ These results indicate that the chiral ligand sparteine coordinates to *n*‐BuLi to act as a chiral base that discriminates for steric reasons between the enantiomers of the starting material, with preferential deprotonation of one diastereomeric complex.^[24]^

**Scheme 5:**
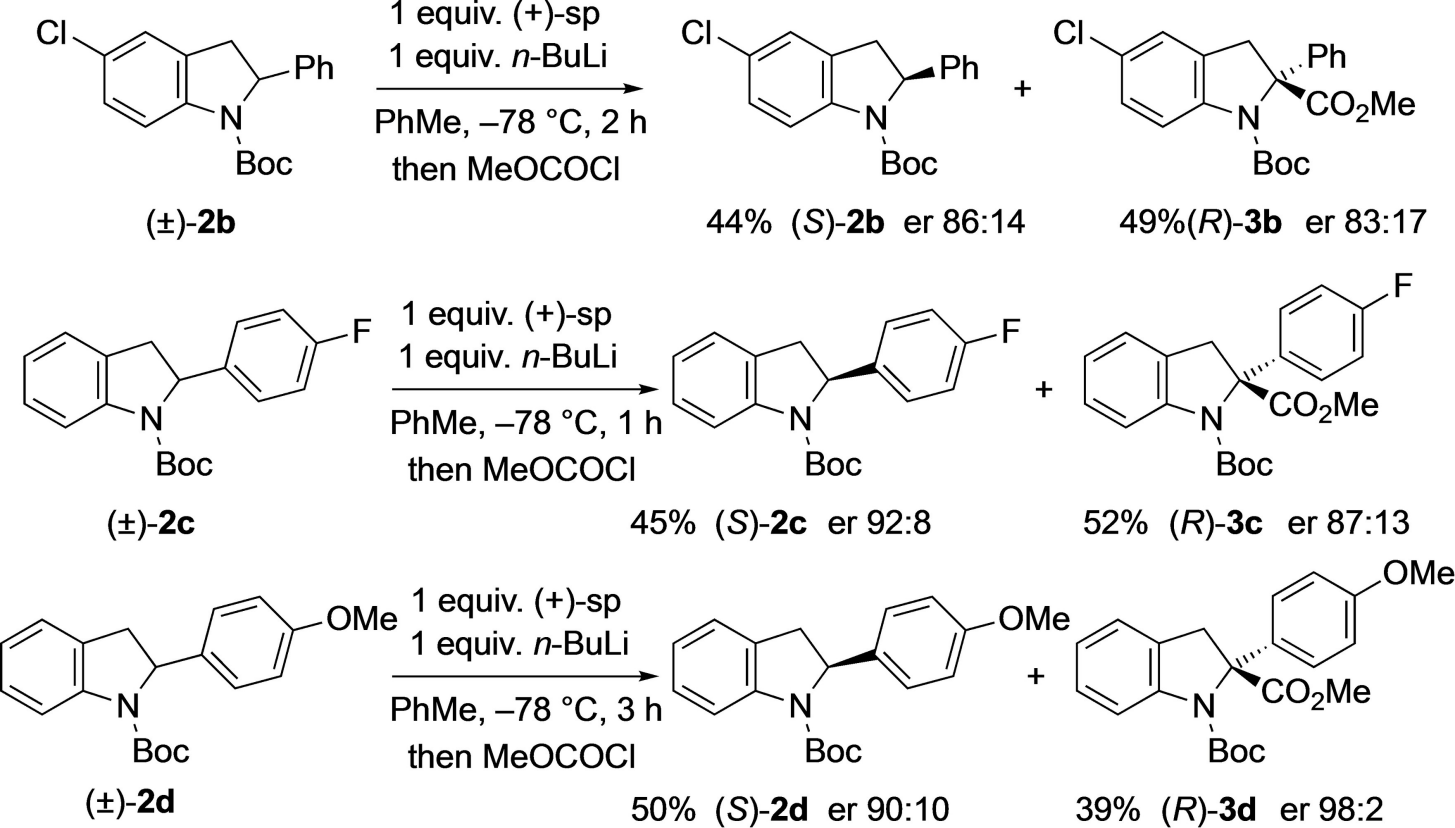
Kinetic resolution of indolines **2** **b**–**d**.

A small selection of indolines **2** **b**–**d** was prepared to explore the scope of the kinetic resolution. These were made by reduction of the corresponding indole followed by Boc protection (see Supporting Information). We were pleased to find that the 5‐substituted derivative **2** **b** was a suitable substrate and resulted in the recovered (*S*)‐**2** **b** after kinetic resolution with good enantioselectivity (Scheme 5). Likewise, the 4‐fluorophenyl derivative **2** **c** gave the recovered (*S*)‐**2** **c** with very good results. However, the 4‐methoxyphenyl derivative **2** **d** was slow to react under these conditions, resulting in 70 % yield of recovered **2** **d** (er 71 : 29) after kinetic resolution with *n*‐BuLi and (+)‐sparteine for 1 h (together with the product **3** **d**, 29 % yield, er 99 : 1). This equates to a relatively high selectivity factor, but clearly further conversion is desirable. Therefore, the kinetic resolution was given more time (3 h rather than 1 h) for lithiation and this improved the yield of the recovered indoline (*S*)‐**2** **d** to 50 % while maintaining a good stereoselectivity (er 90 : 10). The product **3** **d** was also isolated with high selectivity (er 98 : 2). This substrate has a selectivity factor S (*k*_rel_)≈22. This improved value may be due to a slower rate of lithiation of the more electron‐rich substrate thereby allowing improved differentiation between the diastereomeric transition states. We also speculate that the 4‐methoxyphenyl group may slow the rate of rotation of the Boc group (due to a slightly more electron‐rich nitrogen atom and enhanced electrostatic interactions) and this could reduce the local concentration of the rotamer required for lithiation thereby increasing the time required and helping to improve the selectivity. The rate of rotation of the Boc group in indoline **2** **d** was determined by VT NMR spectroscopy (ratio of rotamers ∼6 : 1). From dynamic NMR studies (see Supporting Information), the activation parameters for Boc rotation were determined as ΔH^≠^≈58.7 kJ/mol and ΔS^≠^≈−0.9 J/K/mol for the major rotamer converting to the minor rotamer (and ΔH^≠^≈58.7 kJ/mol and ΔS^≠^≈14.0 J/K/mol for the minor rotamer converting to the major rotamer). Hence, for rotation of the major (undesired) rotamer to the rotamer required for lithiation there is a barrier ΔG^≠^≈58.9 kJ/mol at −78 °C and a half‐life for rotation *t*_1/2_≈17 min. The Gibbs energy for the inverse rotamer conversion was found to be 56.0 kJ mol^−1^, leading to a Gibbs energy difference between the rotamers of ΔG≈2.9 kJ mol^−1^ in agreement with the 1H NMR data. These Gibbs energies are corroborated by our DFT studies using the B3LYP‐D3BJ//def2‐TZVP approach. Here, values of ΔG^≠^=57.2 kJ/mol for the major to minor conversion, ΔG^≠^=53.5 kJ/mol for the minor to major conversion, and ΔG=3.7 kJ mol^−1^ are obtained in good agreement with the experimental data (for Gibbs energies from other approaches, see Supporting Information). Structural analysis of the rotamers **2** **d‐1** and **2** **d‐2** shows only marginal changes compared to **2** **a‐1** and **2** **a‐2**, in line with the small free energy changes for the rotamer conversion.

Our values suggest that rotation is indeed slightly slower for the 4‐methoxyphenyl derivative **2** **d** in comparison with the parent indoline **2** **a**. The results demonstrate that understanding the rate of rotation of the Boc group is helpful for optimization of the reaction conditions. For the case of indoline **2** **d**, a longer reaction time (of about 3 h) is required at −78 °C. This substrate provides very good results under these conditions.

**Scheme 6:**
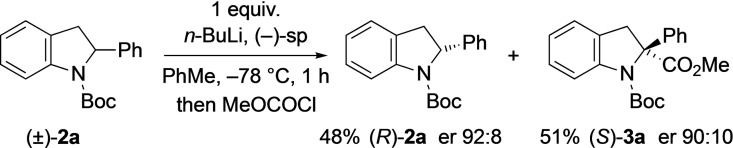
Kinetic resolution of indolines **2** **a** with (−)‐sparteine.

To prepare the opposite enantiomer of the recovered starting material, the chiral ligand (−)‐sparteine can be used. This allowed the formation of the indoline (*R*)‐**2** **a** with good selectivity, together with the disubstituted derivative (*S*)‐**3** **a** (Scheme 6).

**Scheme 7:**
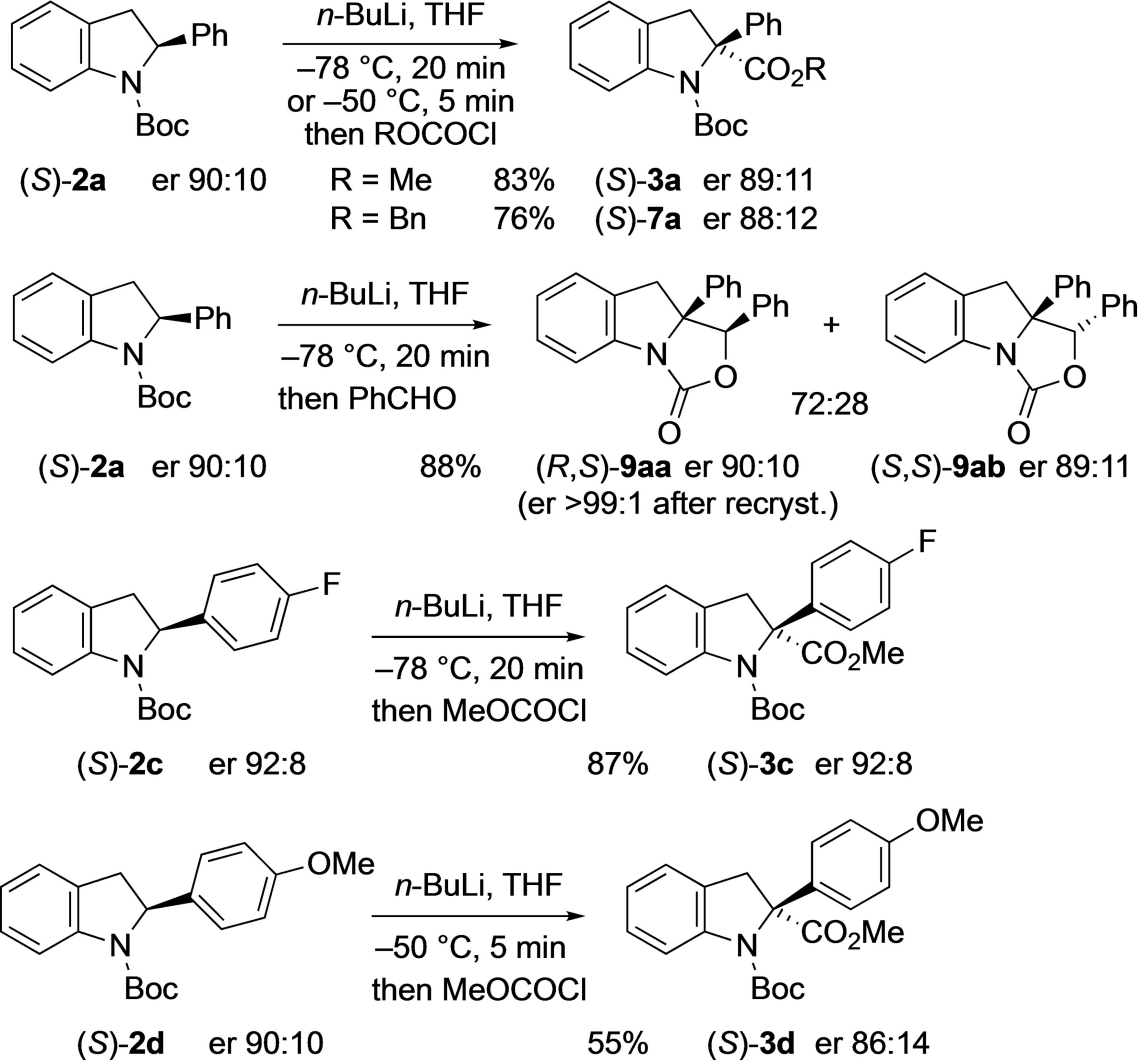
Lithiation–quench of indolines (*S*)‐**2** **a**, **2** **c**, **2** **d**.

An alternative method to prepare either enantiomer of the 2,2‐disubstituted derivatives is to carry out lithiation‐trapping on the enantioenriched starting materials. Hence, lithiation of (*S*)‐**2** **a** (er 90 : 10) with *n*‐BuLi (1 equiv.) in THF followed by electrophilic quench provided the esters (*S*)‐**3** **a** and (*S*)‐**7** **a** without significant loss of enantiomeric purity (Scheme 7). Likewise, electrophilic trapping with benzaldehyde gave the products **9** **aa** and **9** **ab** with very high enantiomer ratios. The major diastereoisomer crystallised to give material of very high selectivity (er 99.4:0.6 by CSP‐HPLC) and single crystal X‐ray analysis was used to determine the major stereoisomer and absolute configuration (see Supporting Information). Similar lithiation–trapping experiments were conducted on the enantioenriched indolines **2** **c** and **2** **d** to give the disubstituted products **3** **c** and **3** **d** (Scheme 7). The reactions were enantiospecific, occurring with retention of configuration.

**Scheme 8:**
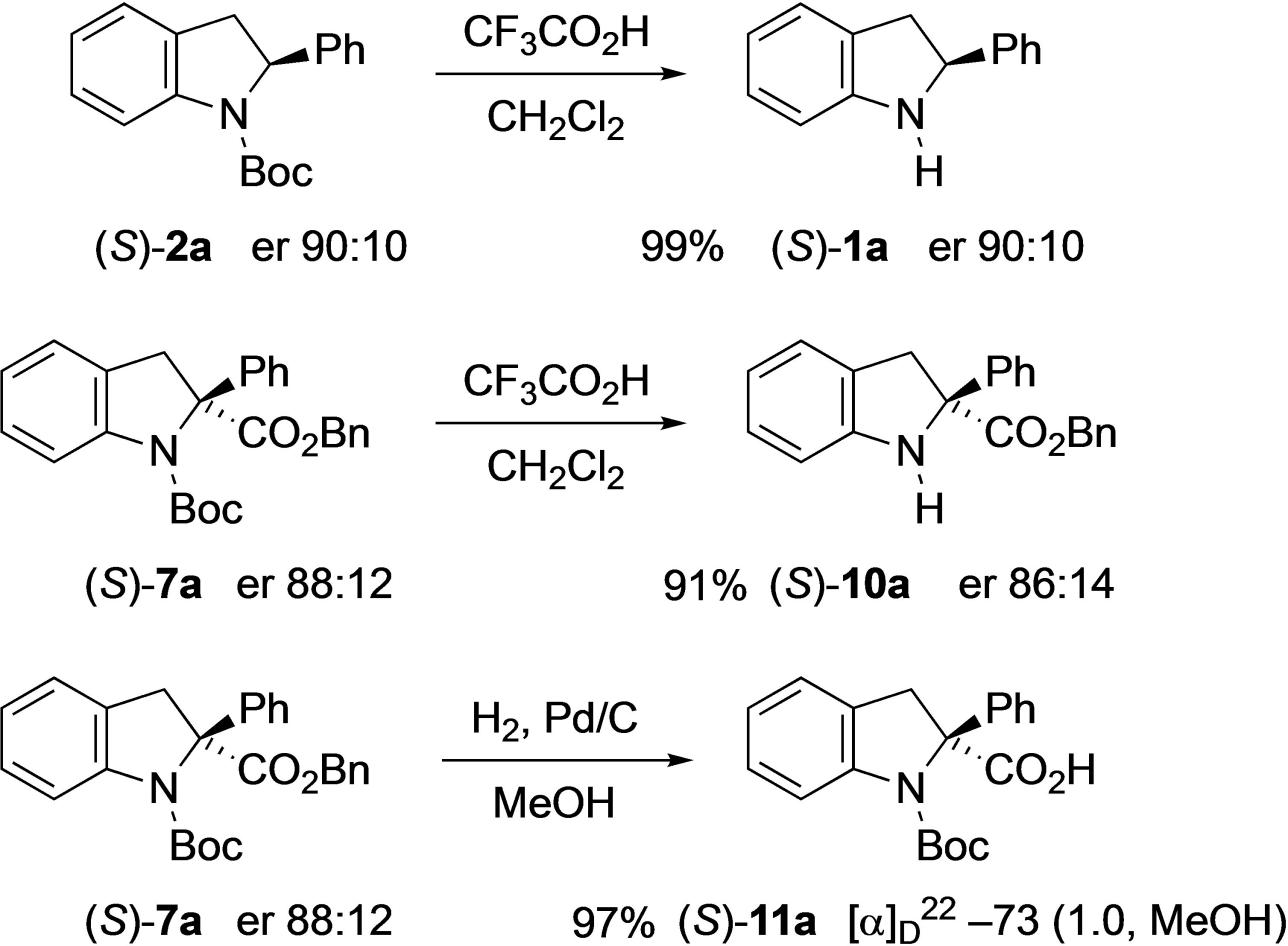
Further transformations of the indoline products.

Finally, we treated several of the products with trifluoroacetic acid which removes the Boc group to give the enantioenriched indolines (*S*)‐**1** **a** and (*S*)‐**10** **a** (Scheme 8). In addition, the ester (*S*)‐**7** **a** could be converted to the acid (*S*)‐**11** **a** using hydrogenolysis. In all cases the reactions occurred without loss of enantiomeric purity. These transformations demonstrate the orthogonal nature of the protecting groups and provide opportunities for further functionalization.

## Conclusions

We have demonstrated an enantioselective kinetic resolution of 2‐arylindolines using *n*‐BuLi and the chiral ligand sparteine. The starting materials and 2,2‐disubstituted products are formed with good enantiomer ratios with a selectivity factor, S up to ∼20. The absolute configuration of the major enantiomer was found to correspond to known asymmetric deprotonations that use sparteine. It is useful to be aware of the rate of rotation of the Boc group and this can be gleaned from in situ IR spectroscopy, variable temperature NMR spectroscopy, and DFT studies. The rate of rotation was fast enough for good yields, despite the fact that it is the minor rotamer that is required for lithiation at C‐2 (ratio ∼6 : 1 in favor of the rotamer with the carbonyl directed towards C‐7). After kinetic resolution, the recovered enantioenriched 2‐arylindolines can be converted to 2,2‐disubstituted products with the opposite absolute configuration in comparison to that obtained in the kinetic resolution; hence, both enantiomers of these 2,2‐disubstituted products can be obtained with high selectivity using the same enantiomer of the chiral ligand sparteine. The enantioenriched 2‐arylindolines and 2,2‐disubstituted indolines have potential to find use in synthesis or medicinal chemistry.

## Computational Methods

All calculations were performed using density functional theory, employing the B3LYP^[18]^ functional as implemented in the E.01 version of Gaussian 09.^[25]^ All dispersion corrections were included through the GD3‐BJ^[20]^ method. Solvent was included *via* the PCM method^[26]^ as implemented in Gaussian with the default parameters for THF. Initial calculations were performed using the 6–311G(d,p)^[19]^ basis set and were repeated using the def2‐TZVP^[21]^ basis set. Unless otherwise stated, all Gibbs energies quoted were evaluated at 195 K and at standard pressure (1 atm).

## Experimental Section

For descriptions of experimental procedures and full characterization of novel compounds, plus NMR spectra, X‐ray data, and DFT data, see the Supporting Information. Deposition Number(s) 2063461 (for (*S*)‐**2** **a**) and 2063462 (for( *R,S*)‐**9** **aa**) contain(s) the supplementary crystallographic data for this paper. These data are provided free of charge by the joint Cambridge Crystallographic Data Centre and Fachinformationszentrum Karlsruhe Access Structures service www.ccdc.cam.ac.uk/structures.

## Conflict of interest

The authors declare no conflict of interest.

## Supporting information

As a service to our authors and readers, this journal provides supporting information supplied by the authors. Such materials are peer reviewed and may be re‐organized for online delivery, but are not copy‐edited or typeset. Technical support issues arising from supporting information (other than missing files) should be addressed to the authors.

Supporting Information
