# Supplementary material for: Asymmetric Synthesis of 2‐Arylindolines and 2,2‐Disubstituted Indolines by Kinetic Resolution
Source: Chemistry. 2021 Jul 8;27(45):11670–5. doi: 10.1002/chem.202101248 (PMC8456874; doi:10.1002/chem.202101248)
Supplement: Supplementary file 1 — Supporting Information [file CHEM-27-11670-s001.pdf]

Chemistry–A European Journal

Supporting Information

Asymmetric Synthesis of 2-Arylindolines and 2,2-Disubstituted Indolines by Kinetic Resolution

Anthony Choi, Ashraf El-Tunsi, Yuhang Wang, Anthony J. H. M. Meijer, Jia Li, Xiabing Li, Ilaria Proietti Silvestri, and Iain Coldham*

Supporting Information

Asymmetric synthesis of 2-arylindolines and 2,2-disubstituted indolines by kinetic resolution

Contents

1. General procedures S-2
2. Experimental procedures and data S-3
   - 2.1 Preparation of racemic substrates **2** S-3
   - 2.2 Preparation of racemic 2,2-disubstituted products S-5
   - 2.3 Kinetic resolution of racemic substrates **2** S-12
   - 2.4 Preparation of enantioenriched 2,2-disubstituted products S-15
   - 2.5 Further functionalization S-17
3. Variable temperature ^{1}H NMR spectra for carbamate **2a** S-20
4. Variable temperature ^{1}H NMR spectra for carbamate **2d** S-23
5. X-ray data for carbamate (*S*)-**2a** S-26
6. X-ray data for carbamate (*R,S*)-**9aa** S-31
7. References S-36
8. HPLC traces S-37
9. NMR spectra S-49
10. DFT data S-69
    - 10.1 Calculations Performed Using the 6-311G(d,p) Basis Set without dispersion S-71
    - 10.2 Calculations Performed Using the 6-311G(d,p) Basis Set with dispersion S-115
    - 10.3 Calculations Performed Using the def2tzvp Basis Set S-159

1. General procedures

Reagents were obtained from commercial suppliers and were used without further purification or after distillation; *n*BuLi was titrated before use. Solvents were obtained from a Grubbs dry solvent system. Thin layer chromatography was performed on Merck silica gel $60F_{254}$ plates and visualised by UV irradiation at 254 nm or by staining with an alkaline $KMnO_4$ dip. Flash column chromatography was performed using DAVISIL or Geduran silica gel (40-63 micron mesh). Melting points were recorded on a Gallenkamp hot stage and were uncorrected. InfraRed spectra were recorded on a Perkin Elmer Spectrum RX Fourier Transform – IR System and only selected peaks are reported. ^{1}H NMR spectra were recorded on a Bruker AC400 (400 MHz) instrument. Chemical shifts are reported in ppm with respect to the residual solvent peaks, with multiplicities given as s = singlet, d = doublet, t = triplet, q = quartet, m = multiplet, br = broad. Coupling constants (*J* values) are quoted to the nearest 0.5 Hz with values in Hertz (Hz). ^{13}C NMR spectra were recorded on the above instrument at 100 MHz. Low and high resolution (accurate mass) mass spectra were recorded on a Walters LCT instrument for Electro–Spray (ES). ReactIR infrared spectroscopic monitoring was performed on a Mettler-Toledo ReactIR iC 4000 spectrometer equipped with a diamond-tipped (DiComp) probe.

Intensity data for X-ray crystal structures were collected at 100 K on a Bruker D8 Venture diffractometer using a Cu K_α microfocus X-ray source.

Suitable crystals were mounted on a MiTiGen microloop using fomblin oil and transferred directly to the cold nitrogen stream at 100 K for data collection on a Bruker D8 VENTURE diffractometer equipped with an Oxford 700+ cryostream, a PHOTON 100 CMOS detector and using Cu-K_α micro-focus X-ray source. Intensity data was collected in shutterless mode with a final fast scan collected at lower incident beam intensity to enable correction for any detector saturation for low-angle data. Data reduction was performed using the Bruker Apex3 software.[1] Intensity data were corrected for absorption using empirical methods (SADABS) based upon symmetry equivalent reflections combined with measurements at different azimuthal angles.[2] The crystal structure was solved using ShelXT[3] and refined against all F^2 values using the SHELXL[4] accessed via the Olex2 program.[5] Non-hydrogen atoms were refined anisotropically. Hydrogen atoms were placed in calculated positions with idealized geometries and then refined by employing a riding model and isotropic displacement parameters.

The selectivity factors (S) in the manuscript were calculated using the formula

$$S = \ln[(1-C)(1-ee)] / \ln[(1-C)(1+ee)]$$

where C is the conversion based on the amount of recovered starting material (*e.g.* for 46% recovered starting material, C = 0.54) and ee is the enantiomeric excess of the recovered starting material (*e.g.* enantiomer ratio, er 90:10 is 80% ee, so ee = 0.8).

All calculations were performed using density functional theory, employing the B3LYP[6] functional as implemented in the D.01 version of Gaussian 09.[7] Calculations included dispersion corrections using the GD3-BJ[8] method. Calculations used the 6-

311G(d,p)[9a,b] or def2TZVP[9c] basis set. Solvent was included *via* the PCM method[10] as implemented in Gaussian with the default parameters for THF.

2. Experimental procedures and data

2.1 Preparation of racemic substrates **2**

tert-Butyl 2-Phenyl-2,3-dihydroindole-1-carboxylate **2a**

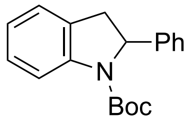

(±)-**2a**

n-BuLi (12.3 mL, 28.2 mmol, 2.3 M in hexane) was added to 2-phenyl-2,3-dihydro-1*H*-indole (5.0 g, 26 mmol) (prepared by reduction of 2-phenylindole with tin powder and HCl in EtOH[11]) in THF (65 mL) at –78 °C. After 30 min, Boc_2O (5.59 g, 25.6 mmol) in THF (25 mL) was added over 10 min. The mixture was allowed to warm to room temperature over 16 h and aqueous saturated $NaHCO_3$ solution (60 mL) was added. The mixture was extracted with Et_2O (3 × 50 mL). The combined organic layers were dried ($MgSO_4$) and the solvent was evaporated. Purification by column chromatography on silica gel, eluting with petrol–EtOAc (98:2), gave the carbamate **2a** (6.05 g, 80%) as an amorphous solid; m.p. 102–104 °C (from petrol–EtOAc), lit.[13] m.p. 111 °C (from hexane–CH_2Cl_2); R_f 0.5 [petrol–EtOAc (90:10)]; FT-IR ν_{max} (ATR)/cm^{-1} 3011, 3029, 2980, 2938, 1700, 1599, 1482, 1388, 1258, 1141, 1061, 760; ^{1}H NMR (400 MHz, $CDCl_3$) δ = 7.96 (1H, br s, CH), 7.34–7.18 (6H, m, 6 × CH), 7.15 (1H, d, *J* = 7.5 Hz, CH), 7.03–6.97 (1H, m, CH), 5.39 (1H, br s, CH), 3.70 (1H, dd, *J* = 16, 11 Hz, CH), 2.99 (1H, dd, *J* = 16, 3 Hz, CH), 1.32 (9H, br s, *t*-Bu); HRMS (ES) Found: MK^+, 334.1201 $C_{19}H_{21}NO_2K$ requires MK^+, 334.1204; LRMS *m/z* (ES), 334.1 (6%), 240 (100%). Data as reported.[12,13]
Resolution between the enantiomers of the carbamate **2a** was achieved using a Beckman system fitted with a Cellulose-1 column (250 mm × 4.6 mm i.d.) as the stationary phase with a mixture of *n*-hexane:isopropanol (99:1 v/v) as the mobile phase at a flow rate of 1 mL·min^{-1}; ambient temperature, detection by UV absorbance at 254 nm. Injection volume was 20 μL of the sample prepared in a 2 g·L^{-1} solution of the eluent. Under these conditions, the components were eluted at 5.9 min and 7.0 min.

tert-Butyl 5-Chloro-2-phenyl-2,3-dihydroindole-1-carboxylate **2b**

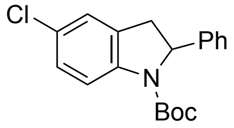

(±)-**2b**

n-BuLi (4.8 mL, 8.1 mmol, 1.7 M in hexane) was added to 5-chloro-2-phenyl-2,3-dihydro-1*H*-indole (1.7 g, 7.4 mmol) in THF (10 mL) at –78 °C. After 30 min, Boc_2O (1.9 g, 8.9 mmol) in THF (5 mL) was added dropwise. The mixture was allowed to

warm to room temperature over 16 h. Aqueous saturated NH_4Cl solution (15 mL) and Et_2O (100 mL) were added. The organic layer was separated, dried ($MgSO_4$) and the solvent was evaporated. Purification by column chromatography on silica gel, eluting with petrol–EtOAc (90:10), gave the carbamate **2b** (1.6 g, 66%) as an amorphous solid; m.p. 70–72 °C (from petrol–EtOAc); R_f 0.4 [petrol–EtOAc (90:10)]; FT-IR ν_{max} (ATR)/cm^{-1} 2976, 2931, 1699, 1475, 1376, 1164, 1138, 1071, 1019, 820; ^{1}H NMR (400 MHz, $CDCl_3$) δ = 7.87 (1H, br s, CH), 7.37–7.14 (6H, m, 6 × CH), 7.11 (1H, br s, CH), 5.56–5.21 (1H, m, CH), 3.67 (1H, dd, *J* = 16.5, 11 Hz, CH), 2.96 (1H, dd, *J* = 16.5, 3 Hz, CH), 1.32 (9H, br s, *t*-Bu); ^{13}C NMR (100 MHz, $CDCl_3$) δ = 152.2, 144.2, 141.9, 131.2 128.6, 127.6, 127.45, 127.4, 125.2, 124.9, 115.5, 81.1, 62.9, 37.5, 28.1; HRMS (ES) Found: MNa^+, 352.1079 $C_{19}H_{20}{}^{35}ClNO_2Na$ requires MNa^+, 352.1080; LRMS *m/z* (ES), 354 (35%), 352 (100%).

Resolution between the enantiomers of the carbamate **2b** was achieved using a Beckman system fitted with a Cellulose-1 column (250 mm × 4.6 mm i.d.) as the stationary phase with a mixture of *n*-hexane:isopropanol (99.3:0.7 v/v) as the mobile phase at a flow rate of 0.7 mL·min^{-1}; ambient temperature, detection by UV absorbance at 254 nm. Injection volume was 20 μL of the sample prepared in a 2 g·L^{-1} solution of the eluent. Under these conditions, the components were eluted at 8.6 min and 9.7 min.

tert-Butyl 2-(4-Fluorophenyl)-2,3-dihydroindole-1-carboxylate **2c**

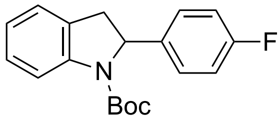

(±)-**2c**

n-BuLi (4.3 mL, 9.8 mmol, 2.3 M in hexane) was added to 2-(4-fluorophenyl)-2,3-dihydro-1*H*-indole (1.9 g, 8.9 mmol) in THF (9 mL) at –78 °C. After 30 min, Boc_2O (2.3 g, 11 mmol) in THF (9 mL) was added over 5 min. The mixture was allowed to warm to room temperature over 16 h and MeOH (20 mL) was added. The solvent was evaporated. Purification by column chromatography on silica gel, eluting with petrol–EtOAc (97:3), gave the carbamate **2c** (2.6 g, 88%) as an amorphous solid; m.p. 96–98 °C (from petrol–EtOAc), no lit. m.p. reported[12]; R_f 0.4 [petrol–EtOAc (90:10)]; ^{1}H NMR (400 MHz, $CDCl_3$) δ = 7.93 (1H, br s, CH), 7.31–7.22 (1H, m, CH), 7.21–7.13 (3H, m, 3 × CH), 7.04–6.95 (3H, m, 3 × CH), 5.37 (1H, br s, CH), 3.69 (1H, dd, *J* = 16.5, 11 Hz, CH), 2.95 (1H, dd, *J* = 16.5, 3 Hz, CH), 1.35 (9H, br s, *t*-Bu). ^{19}F NMR (377 MHz, $CDCl_3$) δ = –115.6. Data as reported.[12]

Resolution between the enantiomers of the carbamate **2c** was achieved using a Beckman system fitted with a Cellulose-1 column (250 mm × 4.6 mm i.d.) as the stationary phase with a mixture of *n*-hexane:isopropanol (99.3:0.7 v/v) as the mobile phase at a flow rate of 0.5 mL·min^{-1}; ambient temperature, detection by UV absorbance at 254 nm. Injection volume was 20 μL of the sample prepared in a 2 g·L^{-1} solution of the eluent. Under these conditions, the components were eluted at 12.6 min and 13.6 min.

tert-Butyl 2-(4-Methoxyphenyl)-2,3-dihydroindole-1-carboxylate **2d**

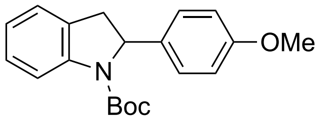

(±)-**2d**

n-BuLi (2.1 mL, 4.8 mmol, 2.3 M in hexane) was added to 2-(4-methoxyphenyl)-2,3-dihydro-1*H*-indole (984 mg, 4.4 mmol) in THF (5 mL) at –78 °C. After 30 min, Boc_2O (1.1 g, 5.2 mmol) in THF (4 mL) was added dropwise. The mixture was allowed to warm to room temperature over 16 h and MeOH (10 mL) was added. The solvent was evaporated. Purification by column chromatography on silica gel, eluting with petrol–EtOAc (95:5), gave the carbamate **2d** (1.3 g, 91%) as an amorphous solid; m.p. 78–80 °C (from petrol–EtOAc), no lit. m.p. reported[14]; R_f 0.6 [petrol–EtOAc (80:20)]; ^{1}H NMR (400 MHz, $CDCl_3$) δ = 7.93 (1H, br s, CH), 7.31–7.21 (1H, m, CH), 7.18–7.10 (3H, m, 3 × CH), 7.00 (1H, t, *J* = 7.5 Hz, CH), 6.83 (2H, d, *J* = 8.5 Hz, 2 × CH), 5.35 (1H, br s, CH), 3.80 (3H, s, CH_3), 3.67 (1H, dd, *J* = 16.5, 10.5 Hz, CH), 2.96 (1H, dd, *J* = 16.5, 2.5 Hz, CH), 1.37 (9H, brs, *t*-Bu). Data as reported.[14]

Resolution between the enantiomers of the carbamate **2d** was achieved using a Beckman system fitted with a Cellulose-1 column (250 mm × 4.6 mm i.d.) as the stationary phase with a mixture of *n*-hexane:isopropanol (99:1 v/v) as the mobile phase at a flow rate of 1 mL·min^{-1}; ambient temperature, detection by UV absorbance at 254 nm. Injection volume was 20 μL of the sample prepared in a 2 g·L^{-1} solution of the eluent. Under these conditions, the components were eluted at 7.5 min and 8.3 min.

2.2 Preparation of racemic 2,2-disubstituted products

1-*tert*-Butyl 2-Methyl 2-Phenyl-3*H*-indole-1,2-dicarboxylate **3a**

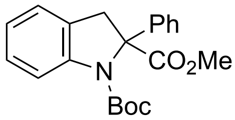

3a

n-BuLi (0.2 mL, 0.41 mmol, 2.1 M in hexanes) was added to *N*-Boc-2-phenylindoline **2a** (100 mg, 0.34 mmol) in THF (4 mL) at –78 °C. After 20 min (or 4 min if the reaction was carried out at –50 °C), MeOCOCl (0.1 mL, 1.3 mmol) was added. The mixture was allowed to warm to room temperature over 16 h and MeOH (1 mL) was added. The solvent was evaporated and the residue was purified by column chromatography on silica gel, eluting with petrol–EtOAc (98:2), to give the carbamate **3a** (110 mg, 88%) as an oil; R_f 0.41 [petrol–EtOAc (90:10)]; FT-IR ν_{max} (ATR)/cm^{-1} 2980, 2950, 1750, 1705, 1485, 1375, 1315, 1240, 1210, 1160, 1140, 1060, 1015, 850; ^{1}H NMR (400 MHz,

CDCl3) δ = 8.07 (1H, br s, CH), 7.50 (2H, d, J = 7.5 Hz, 2 × CH), 7.38–7.31 (2H, m, 2 × CH), 7.31–7.23 (2H, m, 2 × CH), 7.07 (1H, d, J = 7.5 Hz, CH), 7.00 (1H, t, J = 7.5 Hz, CH), 3.90 (1H, d, J = 16.5 Hz, CH), 3.82 (3H, s, CH_3), 3.43 (1H, d, J = 16.5 Hz, CH), 1.30 (9H, s, *t*-Bu); ^{13}C NMR (100 MHz, $CDCl_3$, one signal could not be observed) δ = 172.4, 151.8, 142.7, 141.4, 128.1, 127.8, 127.3, 126.5, 124.3, 123.0, 115.1, 81.8, 73.1, 52.7, 46.4, 28.0; HRMS (ES) Found: MNa^+, 376.1535, $C_{21}H_{23}NO_4Na$ requires MNa^+, 376.1525; LRMS *m*/*z* (ES) 376 (100%), 254 (40%). ^1H NMR data as reported.[15]
Resolution between the enantiomers of the the carbamate **3a** was achieved using a Beckman system fitted with a Lux Cellulose–1 column (250 mm × 4.6 mm i.d.) as the stationary phase with a mixture of n-hexane–isopropanol (99.7:0.3 v/v) as the mobile phase at a flow rate of 0.7 mL·min^{-1}; ambient temperature, detection by UV absorbance at 254 nm. Injection volume 20 µL of the sample prepared in a 2 g·L^{-1} solution of the eluent. Under these conditions, the components were eluted at 16.7 min and 18.6 min.

1-*tert*-Butyl 2-Methyl 5-Chloro-2-phenyl-3*H*-indole-1,2-dicarboxylate **3b**

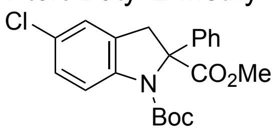

(±)-**3b**

n-BuLi (0.25 mL, 0.61 mmol, 2.4 M in hexanes) was added to indoline **2b** (168 mg, 0.51 mmol) in THF (6 mL) at –50 °C. After 5 min, MeOCOCl (0.08 mL, 1 mmol) was added. The mixture was allowed to warm to room temperature over 16 h and MeOH (2 mL) was added. The solvent was evaporated and the residue was purified by column chromatography on silica gel, eluting with petrol–EtOAc (95:5), to give the carbamate **3b** (177 mg, 90%) as an oil; R_f 0.3 [petrol–EtOAc (90:10)]; FT-IR ν_{max} (ATR)/cm^{-1} 2980, 2950, 1755, 1705, 1475, 1360, 1310, 1245, 1210, 1165, 1135, 1020, 825; ^1H NMR (400 MHz, $CDCl_3$) δ = 7.99 (1H, br s, CH), 7.46 (2H, d, J = 7.5 Hz, 2 × CH), 7.38–7.32 (2H, m, 2 × CH), 7.32–7.26 (1H, m, CH), 7.23 (1H d, J = 8.5 Hz, CH), 7.07–7.02 (1H, m, CH), 3.91–3.78 (4H, m, CH & CH_3), 3.40 (1H, d, J = 16.5 Hz, CH), 1.28 (9H, br s, *t*-Bu); ^{13}C NMR (100 MHz, $CDCl_3$) δ = 172.0, 151.7, 141.5, 141.0, 128.7, 128.0, 127.95, 127.9, 127.5, 126.4, 124.5, 115.9, 82.1, 73.4, 52.8, 45.9, 27.9; HRMS (ES) Found: MNa^+, 410.1143, $C_{21}H_{22}{}^{35}ClNO_4Na$ requires MNa^+, 410.1135; LRMS *m*/*z* (ES) 412 (35%), 410 (100%).
Resolution between the enantiomers of the carbamate **3b** was achieved using a Beckman system fitted with a Cellulose-2 column (250 mm × 4.6 mm i.d.) as the stationary phase with a mixture of *n*-hexane:isopropanol (99.7:0.3 v/v) as the mobile phase at a flow rate of 0.2 mL·min^{-1}; ambient temperature, detection by UV absorbance at 254 nm. Injection volume was 20 µL of the sample prepared in a 2 g·L^{-1} solution of the eluent. Under these conditions, the components were eluted at 43 min and 48 min.

1-*tert*-Butyl 2-Methyl 2-(4-Fluorophenyl)-3*H*-indole-1,2-dicarboxylate **3c**

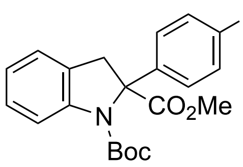

(±)-**3c**

n-BuLi (0.25 mL, 0.61 mmol, 2.4 M in hexanes) was added to indoline **2c** (159 mg, 0.51 mmol) in THF (6 mL) at –50 °C. After 5 min, MeOCOCl (0.08 mL, 1.0 mmol) was added. The mixture was allowed to warm to room temperature over 16 h and MeOH (2 mL) was added. The solvent was evaporated and the residue was purified by column chromatography on silica gel, eluting with petrol–EtOAc (95:5), to give the carbamate **3c** (154 mg, 81%) as an oil; R_f 0.3 [pentane–EtOAc (90:10)]; FT-IR ν_{max} (ATR)/cm^{-1} 2980, 2950, 1750, 1705, 1510, 1485, 1375, 1315, 1225, 1165, 1145, 1055, 1010, 830; ^{1}H NMR (400 MHz, $CDCl_3$) δ = 8.05 (1H, br s, CH), 7.48 (2H, br s, 2 × CH), 7.33–7.24 (1H, m, CH), 7.08 (1H, d, *J* = 7.5 Hz, CH), 7.06–6.98 (3H, m, 3 × CH), 3.88 (1H, d, *J* = 16.5 Hz, CH), 3.82 (3H, s, CH_3), 3.37 (1H, d, *J* = 16.5 Hz CH), 1.31 (9H, br s, *t*-Bu); ^{13}C NMR (100 MHz, $CDCl_3$) δ = 172.3, 161.9 (d, *J* = 246 Hz), 151.6, 142.5, 137.1, 128.4 (d, *J* = 7 Hz), 128.1, 126.6, 124.4, 123.1, 115.1, 114.6 (d, *J* = 21.5 Hz), 81.9, 72.5, 52.7, 46.3, 28.0; ^{19}F NMR (377 MHz, $CDCl_3$) δ = –115.6; HRMS (ES) Found: MNa$^+$, 394.1445, $C_{21}H_{22}FNO_4Na$ requires MNa$^+$, 394.1425; LRMS *m*/*z* (ES) 394 (100%), 316 (40%), 272 (95%).

Resolution between the enantiomers of the the carbamate **3c** was achieved using a Beckman system fitted with a Lux Cellulose–1 column (250 mm × 4.6 mm i.d.) as the stationary phase with a mixture of *n*-hexane–isopropanol (99.4:0.6 v/v) as the mobile phase at a flow rate of 0.8 mL·min^{-1}; ambient temperature, detection by UV absorbance at 254 nm. Injection volume 20 µL of the sample prepared in a 2 g·L^{-1} solution of the eluent. Under these conditions, the components were eluted at 10.2 min and 10.9 min. Alternatively, using *n*-hexane–isopropanol (99.7:0.3 v/v) as the mobile phase at a flow rate of 0.5 mL·min^{-1}, the components were eluted at 21.3 min and 23.6 min.

1-*tert*-Butyl 2-Methyl 2-(4-Methoxyphenyl)-3*H*-indole-1,2-dicarboxylate **3d**

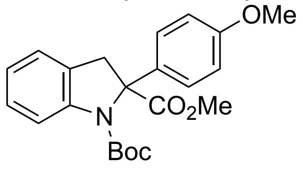

(±)-**3d**

n-BuLi (0.25 mL, 0.61 mmol, 2.4 M in hexanes) was added to indoline **2d** (165 mg, 0.51 mmol) in THF (6 mL) at –50 °C. After 5 min, MeOCOCl (0.08 mL, 1 mmol) was added. The mixture was allowed to warm to room temperature over 16 h and MeOH (2 mL) was added. The solvent was evaporated and the residue was purified by column chromatography on silica gel, eluting with pentane–EtOAc (93:7), to give the

carbamate **3d** (163 mg, 84%) as an oil; R_f 0.25 [pentane–EtOAc (90:10)]; FT-IR ν_{max} (ATR)/cm^{-1} 2975, 2950, 1755, 1705, 1510, 1485, 1370, 1315, 1240, 1185, 1165, 1145, 1060, 1025, 830; ^{1}H NMR (400 MHz, $CDCl_3$) δ = 8.05 (1H, br s, CH), 7.41 (2H, d, *J* = 8.5 Hz, 2 × CH), 7.30–7.23 (1H, m, CH), 7.07 (1H, d, *J* = 7 Hz, CH), 6.99 (1H, t, *J* = 7 Hz, CH), 6.90–6.82 (2H, m, 2 × CH), 3.87 (1H, d, *J* = 16 Hz, CH), 3.83–3.76 (6H, m, 2 × CH_3), 3.39 (1H, d, *J* = 16 Hz, CH), 1.32 (9H, br s, t-Bu); ^{13}C NMR (100 MHz, $CDCl_3$) δ = 172.6, 158.7, 151.8, 142.6, 133.4, 128.0, 127.8, 126.9, 124.4, 123.0, 115.0, 113.1, 81.7, 72.7, 55.2, 52.6, 46.4, 28.1; HRMS (ES) Found: MNa$^+$, 406.1644, $C_{22}H_{25}NO_5Na$ requires MNa$^+$, 406.1625; LRMS *m/z* (ES) 406 (100%), 284 (60%).
Resolution between the enantiomers of the the carbamate **3d** was achieved using a Beckman system fitted with a Lux Cellulose–1 column (250 mm × 4.6 mm i.d.) as the stationary phase with a mixture of n-hexane–isopropanol (99.7:0.3 v/v) as the mobile phase at a flow rate of 1 mL·min^{-1}; ambient temperature, detection by UV absorbance at 254 nm. Injection volume 20 µL of the sample prepared in a 2 g·L^{-1} solution of the eluent. Under these conditions, the components were eluted at 30.7 min and 43.8 min.

tert-Butyl 2-Butyl-2-phenyl-2,3-dihydro-1*H*-indole-1-carboxylate **4a**

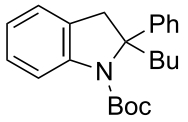

4a

n-BuLi (0.25 mL, 0.61 mmol, 2.4 M in hexanes) was added to indoline **2a** (150 mg, 0.51 mmol) in THF (6 mL) at –50 °C. After 5 min, *n*-BuBr (0.11 mL, 1 mmol) was added. The mixture was allowed to warm to room temperature over 16 h and MeOH (2 mL) was added. The solvent was evaporated and the residue was purified by column chromatography on silica gel, eluting with pentane–EtOAc (93:7), to give the carbamate **4a** (161 mg, 90%) as an oil; R_f 0.8 [pentane–EtOAc (90:10)]; FT-IR ν_{max} (ATR)/cm^{-1} 2965, 2925, 2870, 1700, 1485, 1375, 1365, 1310, 1245, 1160, 1085, 1065, 855; ^{1}H NMR (400 MHz, $CDCl_3$) δ = 8.06 (1H, br s, CH), 7.34–7.27 (4H, m, 4 × CH), 7.27–7.19 (2H, m, 2 × CH), 7.08 (1H, d, *J* = 7.5 Hz, CH), 6.98 (1H, t, *J* = 7.5 Hz, CH), 3.39 (1H, d, *J* = 16.5 Hz, CH), 3.28 (1H, d, *J* = 16.5 Hz, CH), 2.71 (1H, br s, CH), 2.13–2.00 (1H, m, CH), 1.50–1.37 (2H, m, CH_2), 1.38–1.06 (11H, m, CH_2 & *t*-Bu), 0.95 (3H, t, *J* = 7.0 Hz, CH_3); ^{13}C NMR (100 MHz, $CDCl_3$, one signal could not be observed) δ = 152.5, 149.4, 144.6, 128.3, 127.6, 126.4, 124.2, 124.1, 122.4, 114.8, 80.5, 70.0, 46.0, 38.2, 28.0, 25.4, 23.1, 14.1; HRMS (ES) Found: MNa$^+$, 374.2104, $C_{23}H_{29}NO_2Na$ requires MNa$^+$, 374.2091; LRMS *m/z* (ES) 374 (50%), 352 (20%), 296 (100%).

tert-Butyl 2-Methyl-2-phenyl-2,3-dihydro-1*H*-indole-1-carboxylate **5a**

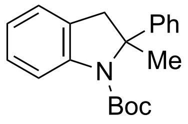

5a

n-BuLi (0.25 mL, 0.61 mmol, 2.4 M in hexanes) was added to indoline **2a** (150 mg, 0.51 mmol) in THF (6 mL) at –50 °C. After 5 min, MeI (0.06 mL, 1 mmol) was added. The mixture was allowed to warm to room temperature over 16 h and MeOH (2 mL) was added. The solvent was evaporated and the residue was purified by column chromatography on silica gel, eluting with pentane–EtOAc (93:7), to give the carbamate **5a** (152 mg, 97%) as an oil; R_f 0.7 [pentane–EtOAc (90:10)]; FT-IR ν_{max} (ATR)/cm^{-1} 2970, 2930, 1700, 1485, 1460, 1385, 1365, 1315, 1235, 1165, 1140, 1080, 1055, 850; ^{1}H NMR (400 MHz, $CDCl_3$) δ = 8.01 (1H, br s, CH), 7.40–7.18 (6H, m, 6 × CH), 7.12 (1H, d, J = 7.5 Hz, CH), 7.01 (1H, t, J = 7.5 Hz, CH), 3.37 (1H, d, J = 16.5 Hz, CH), 3.23 (1H, d, J = 16.5 Hz, CH), 1.94 (3H, s, CH_3), 1.17 (9H, br s, t-Bu); ^{13}C NMR (100 MHz, $CDCl_3$, one signal could not be observed) δ = 152.3, 148.2, 143.2, 128.2, 127.7, 126.5, 124.6, 124.5, 122.5, 115.2, 80.6, 68.0, 48.6, 28.0, 25.2; HRMS (ES) Found: MNa$^+$, 332.1632, $C_{20}H_{23}NO_2Na$ requires MNa$^+$, 332.1621; LRMS m/z (ES) 332 (50%), 254 (100%), 210 (30%).

tert-Butyl 2-Phenyl-2-(tributylstannyl)-2,3-dihydro-1*H*-indole-1-carboxylate **6a**

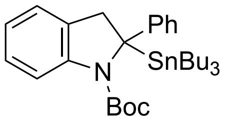

6a

n-BuLi (0.25 mL, 0.61 mmol, 2.4 M in hexanes) was added to indoline **2a** (150 mg, 0.51 mmol) in THF (6 mL) at –50 °C. After 5 min, n-Bu_3SnCl (0.28 mL, 1 mmol) was added. The mixture was allowed to warm to room temperature over 16 h and MeOH (2 mL) was added. The solvent was evaporated and the residue was purified by column chromatography on silica gel with K_2CO_3 (10% w/w), eluting with pentane–EtOAc (99:1), to give the carbamate **6a** (220 mg, 74%) as an oil; R_f 0.7 [pentane–EtOAc (95:5)]; FT-IR ν_{max} (ATR)/cm^{-1} 2950, 2920, 2870, 2855, 1675, 1595, 1480, 1385, 1260, 1170, 1130, 1080, 1020, 875; ^{1}H NMR (400 MHz, $CDCl_3$) δ = 7.61 (1H, d, J = 8.0 Hz, CH), 7.23 (2H, t, J = 8.0 Hz, 2 × CH), 7.15 (1H, t, J = 8.0 Hz, CH), 7.10–7.00 (4H, m, 4 × CH), 6.91 (1H, t, J = 7.5 Hz, CH), 3.79– 3.58 (1H, m, CH), 3.43–3.32 (1H, m, CH), 1.67 (9H, s, t-Bu), 1.54–1.35 (6H, m, 3 × CH_2), 1.35–1.20 (6H, m, 3 × CH_2), 1.01–0.75 (15H, m, 3 × CH_2 & 3 × CH_3); ^{13}C NMR (100 MHz, $CDCl_3$) δ = 153.7, 148.2, 142.0, 131.9, 128.2, 126.9, 124.7, 124.5, 123.6, 122.5, 115.9, 81.6, 42.4, 29.1, 28.6, 27.6, 13.7, 12.5; HRMS (ES) Found: MNa$^+$, 608.2507, $C_{31}H_{47}NO_2Na^{120}Sn$ requires MNa$^+$, 608.2521; LRMS m/z (ES) 608 (100%).

1-*tert*-Butyl 2-Benzyl 2-phenyl-3*H*-indole-1,2-dicarboxylate **7a**

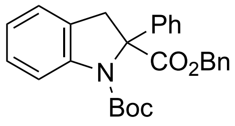

7a

n-BuLi (0.51 mL, 1.2 mmol, 2.4 M in hexanes) was added to indoline **2a** (300 mg, 1 mmol) in THF (12 mL) at –50 °C. After 5 min, BnOCOCl (0.29 mL, 2 mmol) was added. The mixture was allowed to warm to room temperature over 16 h and MeOH (4 mL) was added. The solvent was evaporated and the residue was purified by column chromatography on silica gel, eluting with pentane–EtOAc (97:3), to give the carbamate **7a** (377 mg, 86%) as an oil; R_f 0.4 [pentane–EtOAc (80:20)]; FT-IR ν_{max} (ATR)/cm^{-1} 3065, 3035, 2975, 2935, 1750, 1710, 1485, 1375, 1310, 1245, 1210, 1165, 1140, 1055, 1015, 855; ^{1}H NMR (400 MHz, $CDCl_3$) δ = 8.08 (1H, br s, CH), 7.51 (2H, d, *J* = 7.5 Hz, 2 × CH), 7.39–7.23 (9H, m, 9 × CH), 7.06 (1H, d, *J* = 7.5 Hz, CH), 6.99 (1H, t, *J* = 7.5 Hz, CH), 5.34–5.20 (2H, m, CH_2), 3.86 (1H, d, *J* = 16.5 Hz, CH), 3.42 (1H, d, *J* = 16.5 Hz, CH), 1.20 (9H, br s, *t*-Bu); ^{13}C NMR (100 MHz, $CDCl_3$, two signals could not be observed) δ = 171.6, 151.9, 142.9, 141.4, 135.5, 128.6, 128.3, 128.1, 127.9, 127.3, 126.6, 124.3, 123.0, 115.0, 81.9, 73.2, 67.7, 46.5, 27.9; HRMS (ES) Found: MNa$^+$, 452.1842, $C_{27}H_{27}NO_4Na$ requires MNa$^+$, 452.1832; LRMS *m*/*z* (ES) 452 (100%), 374 (15%), 330 (85%).

Resolution between the enantiomers of the the carbamate **71** was achieved using a Beckman system fitted with a Lux Cellulose–1 column (250 mm × 4.6 mm i.d.) as the stationary phase with a mixture of n-hexane–isopropanol (99.7:0.3 v/v) as the mobile phase at a flow rate of 0.5 mL·min^{-1}; ambient temperature, detection by UV absorbance at 254 nm. Injection volume 20 µL of the sample prepared in a 2 g·L^{-1} solution of the eluent. Under these conditions, the components were eluted at 36.5 min and 58.4 min.

tert-Butyl 2-(Trimethylsilyl)-2-phenyl-2,3-dihydro-1*H*-indole-1-carboxylate **8a**

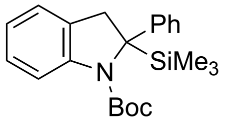

8a

n-BuLi (0.24 mL, 0.61 mmol, 2.5 M in hexanes) was added to indoline **2a** (150 mg, 0.51 mmol) in THF (6 mL) at –50 °C. After 5 min, Me_3SiCl (0.13 mL, 1 mmol) was added. The mixture was allowed to warm to room temperature over 16 h and MeOH (2 mL) was added. The solvent was evaporated and the residue was purified by column chromatography on silica gel, eluting with petrol–EtOAc (97:3), to give the carbamate **8a** (179 mg, 96%) as an oil; R_f 0.6 [petrol–EtOAc (90:10)]; FT-IR ν_{max} (ATR)/cm^{-1} 2970, 2905, 1685, 1600, 1480, 1370, 1335, 1250, 1165, 1120, 1080, 1030, 1000, 915, 895, 845; ^{1}H NMR (400 MHz, $CDCl_3$) δ = 7.60 (1H, d, *J* = 7.5 Hz, CH), 7.32–7.23 (2H, m, 2 × CH), 7.22–7.11 (4H, m, 4 × CH), 7.08 (1H, d, *J* = 7.5 Hz, CH), 6.90 (1H, t, *J* = 7.5 Hz, CH), 3.57 (1H, d, *J* = 16 Hz, CH), 3.23 (1H, d, *J* = 16 Hz, CH), 1.68 (9H, s, *t*-Bu), 0.18 (9H, s, 3 × CH_3); ^{13}C NMR (100 MHz, $CDCl_3$, quaternary signals could not be observed) δ = 153.6, 128.2, 127.1, 125.6, 124.7, 124.6, 122.4, 115.9, 81.4, 41.4, 28.5, –0.6; HRMS (ES) Found: MNa$^+$, 390.1872, $C_{22}H_{29}NO_2NaSi$ requires MNa$^+$, 390.1860; LRMS *m*/*z* (ES) 390 (20%), 296 (100%).

1,9a-Diphenyl-1H,9H-[1,3]oxazolo[3,4-a]indol-3-one **9a**

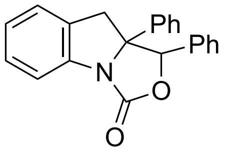

9a

n-BuLi (0.51 mL, 1.2 mmol, 2.4 M) was added to the carbamate (±)-**2a** (300 mg, 1 mmol) in THF (12 mL) at –50 °C. After 5 min, benzaldehyde (0.21 mL, 2 mmol) was added and the mixture was allowed to warm to room temperature over 16 h then MeOH (4 mL) was added. The solvent was removed under reduced pressure and the product was purified by column chromatography on silica gel, eluting with pentane–EtOAc (95:5), to give indoline (1*RS*,9a*SR*)-**9aa** (186 mg, 56%) as needles; m.p. 170–172 °C (from petrol–EtOAc); R_f 0.35 [pentane–EtOAc (95:5)]; FT-IR ν_{max} (ATR)/cm^{-1} 3055, 3030, 1760, 1605, 1595, 1485, 1375, 1295, 1170, 1135, 1075, 1040, , 1020, 1000, 760; ^{1}H NMR (400 MHz, $CDCl_3$) δ = 7.68 (1H, d, *J* 8 Hz, CH), 7.33 (1H, t, *J* = 8 Hz, CH), 7.26– 7.14 (4H, m, 4 × CH), 7.13–7.05 (4H, m, 4 × CH), 7.03–6.93 (4H, m, 4 × CH), 5.90 (1H, s, CH), 3.88 (1H, d, *J* = 16 Hz, CH), 3.57 (1H, d, *J* = 16 Hz, CH); ^{13}C NMR (100 MHz, $CDCl_3$) δ = 155.4, 139.4, 139.2, 133.8, 131.8, 128.8, 128.3, 128.1, 127.8, 127.7, 126.6, 126.4, 125.4, 124.8, 114.9, 90.7, 75.9, 44.1; HRMS (ES) Found: MNa^+, 350.1167. $C_{22}H_{17}NO_2Na$ requires MNa^+, 350.1151; LRMS *m/z* (ES) 677 (100%), 350 (50%), 167 (35%); and the indoline (1*RS*,9a*RS*)-**9ab** (57 mg, 17%) as an oil; R_f 0.45 [pentane–EtOAc (95:5)]; FT-IR ν_{max} (ATR)/cm^{-1} 3055, 3030, 1760, 1605, 1595, 1485, 1375, 1295, 1170, 1075, 1040, 1020, 1000, 760; ^{1}H NMR (400 MHz, $CDCl_3$) δ = 7.66 (1H, d, *J* = 8.0 Hz, CH), 7.57–7.52 (2H, m, 2 × CH), 7.51–7.42 (5H, m, 5 × CH), 7.39–7.34 (3H, m, 3 × CH), 7.33–7.27 (1H, m, CH), 7.04 (1H, t, *J* = 7.5 Hz, CH), 6.97 (1H, d, *J* = 7.5 Hz, CH), 5.78 (1H, s, CH), 3.15 (1H, d, *J* = 16 Hz, CH), 2.94 (1H, d, *J* = 16 Hz, CH); ^{13}C NMR (100 MHz, $CDCl_3$, two signals could not be observed) δ = 156.4, 145.7, 139.8, 136.3, 131.9, 129.3, 129.2, 128.2, 128.1, 125.8, 125.2, 125.1, 124.6, 115.7, 87.6, 40.9; HRMS (ES) Found: MNa^+, 350.1167. $C_{22}H_{17}NO_2Na$ requires MNa^+, 350.1151; LRMS *m/z* (ES) 677 (100%), 350 (50%), 167 (35%).

Resolution between the enantiomers of the carbamate **9aa** was achieved using a Beckman system fitted with a Cellulose-1 column (250 mm × 4.6 mm i.d.) as the stationary phase with a mixture of *n*-hexane:isopropanol (99.3:0.7 v/v) as the mobile phase at a flow rate of 1 mL·min^{-1}; ambient temperature, detection by UV absorbance at 254 nm. Injection volume was 20 µL of the sample prepared in a 2 g·L^{-1} solution of the eluent. Under these conditions, the components were eluted at 16.7 min and 29.3 min.

Resolution between the enantiomers of the carbamate **9ab** was achieved using a Beckman system fitted with a Cellulose-1 column (250 mm × 4.6 mm i.d.) as the stationary phase with a mixture of *n*-hexane:isopropanol (99:1 v/v) as the mobile phase at a flow rate of 0.5 mL·min^{-1}; ambient temperature, detection by UV absorbance at 254 nm. Injection volume was 20 µL of the sample prepared in a 2 g·L^{-1} solution of the eluent. Under these conditions, the components were eluted at 44.8 min and 49.8 min.

2.3 Kinetic resolution of racemic substrates **2**

Resolution of indoline **2a** with (+)-sparteine

tert-Butyl (2*S*)-2-Phenyl-2,3-dihydroindole-1-carboxylate (*S*)-**2a** and 1-*tert*-Butyl 2-Methyl (2*R*)-2-phenyl-3H-indole-1,2-dicarboxylate (*R*)-**3a**

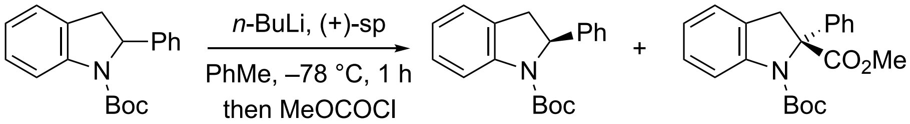

(±)-**2a** (*S*)-**2a** (*R*)-**3a**

n-BuLi (0.14 mL, 0.34 mmol, 2.4 M in hexanes) was added to a mixture of (+)-sparteine (79 mg, 0.34 mmol) and the racemic carbamate **2a** (100 mg, 0.34 mmol) in dry PhMe (8 mL) at –78 °C. After 1 h, MeOCOCl (0.07 mL, 0.85 mmol) was added and the mixture was allowed to warm to room temperature over 16 h then MeOH (1 mL) was added. The solvent was evaporated, and the residue was purified by column chromatography on silica gel, eluting with pentane–EtOAc (96:4), to give recovered carbamate (*S*)-**2a** (46 mg, 46%) as an amorphous solid; m.p. 104–106 °C; data as above (see page S-3); the enantiomeric ratio was determined to be 90:10 by CSP-HPLC as described above (major component eluted at 5.8 min); $[\alpha]_{\mathrm{D}}^{21}$ –64 (1.1, $CHCl_3$), lit.[16] $[\alpha]_{\mathrm{D}}^{20}$ +29.6 (0.5, $CHCl_3$) for 66% ee of *R* enantiomer, lit.[17] $[\alpha]_{\mathrm{D}}^{20}$ –72.4 (0.5, $CHCl_3$) for 97% ee of *S* enantiomer. In addition, the carbamate (*R*)-**3a** (59 mg, 49%) was isolated as an oil, data as above (see page S-5); the enantiomeric ratio was determined to be 90:10 by CSP-HPLC (major component eluted at 16.6 min); $[\alpha]_{\mathrm{D}}^{21}$ +95 (1.4, $CHCl_3$).

Resolution of indoline **2a** with (–)-sparteine

tert-Butyl (2*R*)-2-Phenyl-2,3-dihydroindole-1-carboxylate (*R*)-**2a** and 1-*tert*-Butyl 2-Methyl (2*S*)-2-phenyl-3H-indole-1,2-dicarboxylate (*S*)-**3a**

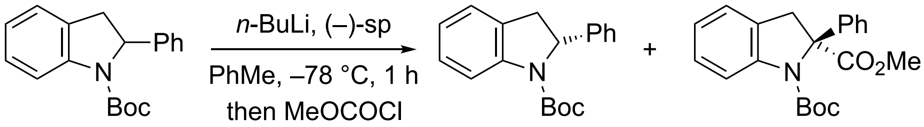

(±)-**2a** (*R*)-**2a** (*S*)-**3a**

n-BuLi (0.14 mL, 0.34 mmol, 2.4 M in hexanes) was added to a mixture of (–)-sparteine (87 mg, 0.37 mmol) and the racemic carbamate **2a** (100 mg, 0.34 mmol) in dry PhMe (8 mL) at –78 °C. After 1 h, MeOCOCl (0.07 mL, 0.85 mmol) was added and the mixture was allowed to warm to room temperature over 16 h then MeOH (1 mL) was

added. The solvent was evaporated, and the residue was purified by column chromatography on silica gel, eluting with pentane–EtOAc (96:4), to give recovered carbamate (*R*)-**2a** (48 mg, 48%) as an amorphous solid; m.p. 102–104 °C; data as above (see page S-3); the enantiomeric ratio was determined to be 92:8 by CSP-HPLC as described above (major component eluted at 6.8 min); $[\alpha]_{\mathrm{D}}^{23}$ +70 (1.2, $CHCl_3$), lit.[16] $[\alpha]_{\mathrm{D}}^{20}$ +29.6 (0.5, $CHCl_3$) for 66% ee of *R* enantiomer, lit.[17] $[\alpha]_{\mathrm{D}}^{20}$ –72.4 (0.5, $CHCl_3$) for 97% ee of *S* enantiomer. In addition, the carbamate (*S*)-**3a** (61 mg, 51%) was isolated as an oil, data as above (see page S-5); the enantiomeric ratio was determined to be 90:10 by CSP-HPLC (major component eluted at 18.1 min); $[\alpha]_{\mathrm{D}}^{23}$ –93 (1.5, $CHCl_3$).

Resolution of indoline **2b** with (+)-sparteine

tert-Butyl (2*S*)-5-Chloro-2-phenyl-2,3-dihydroindole-1-carboxylate (*S*)-**2b** and 1-*tert*-Butyl (2*R*)-2-Methyl 5-Chloro-2-phenyl-3*H*-indole-1,2-dicarboxylate (*R*)-**3b**

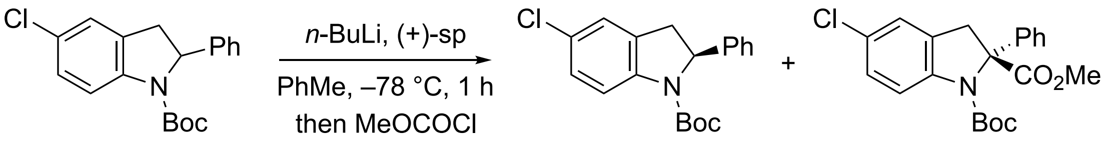

(±)-**2b** (*S*)-**2b** (*R*)-**3b**

n-BuLi (0.14 mL, 0.34 mmol, 2.4 M in hexanes) was added to a mixture of (+)-sparteine (79 mg, 0.34 mmol) and the racemic carbamate **2b** (112 mg, 0.34 mmol) in dry PhMe (8 mL) at –78 °C. After 2 h, MeOCOCl (0.07 mL, 0.85 mmol) was added and the mixture was allowed to warm to room temperature over 16 h then MeOH (1 mL) was added. The solvent was evaporated, and the residue was purified by column chromatography on silica gel, eluting with pentane–EtOAc (96:4), to give recovered carbamate (*S*)-**2b** (49 mg, 44%) as an oil; data as above (see page S-3); the enantiomeric ratio was determined to be 86:14 by CSP-HPLC as described above (major component eluted at 9.6 min); $[\alpha]_{\mathrm{D}}^{22}$ –25 (1.6, $CHCl_3$). In addition, the carbamate (*R*)-**3b** (65 mg, 49%) was isolated as an oil, data as above (see page S-6); the enantiomeric ratio was determined to be 83:17 by CSP-HPLC (major component eluted at 43 min); $[\alpha]_{\mathrm{D}}^{22}$ +56 (1.9, $CHCl_3$).

Resolution of indoline **2c** with (+)-sparteine

tert-Butyl (2*S*)-2-(4-Fluorophenyl)-2,3-dihydroindole-1-carboxylate (*S*)-**2c** and 1-*tert*-Butyl (2*R*)-2-Methyl 2-(4-Fluorophenyl)-3*H*-indole-1,2-dicarboxylate (*R*)-**3c**

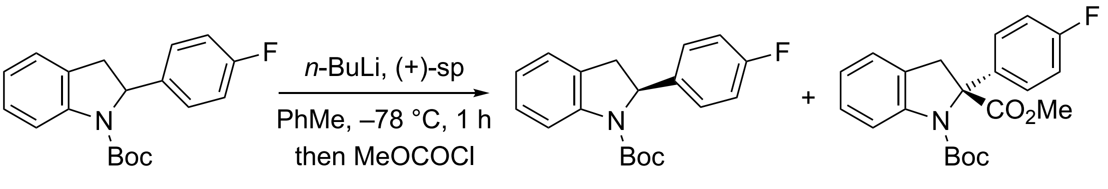

(±)-**2c** (*S*)-**2c** (*R*)-**3c**

n-BuLi (0.14 mL, 0.34 mmol, 2.4 M in hexanes) was added to a mixture of (+)-sparteine (79 mg, 0.34 mmol) and the racemic carbamate **2c** (106 mg, 0.34 mmol) in dry PhMe (8 mL) at –78 °C. After 1 h, MeOCOCl (0.07 mL, 0.85 mmol) was added and the mixture was allowed to warm to room temperature over 16 h then MeOH (1 mL) was added. The solvent was evaporated, and the residue was purified by column chromatography on silica gel, eluting with pentane–EtOAc (96:4), to give recovered carbamate (*S*)-**2c** (48 mg, 45%) as an amorphous solid; m.p. 94–96 °C (from pentane–EtOAc); data as above (see page S-4); the enantiomeric ratio was determined to be 92:8 by CSP-HPLC as described above (major component eluted at 13.4 min); $[\alpha]_D^{22}$ –55 (1.1, $CHCl_3$), lit.[18] $[\alpha]_D^{24}$ –69.4 (1.02, $CHCl_3$) for 93% ee of S enantiomer. In addition, the carbamate (*R*)-**3c** (66 mg, 52%) was isolated as an oil, data as above (see page S-6/7); the enantiomeric ratio was determined to be 87:13 by CSP-HPLC (major component eluted at 20.2 min, minor component at 23 min using hexane–^{i}PrOH 99.7:0.3 at 0.5 mL/min); $[\alpha]_D^{21}$ +83 (1.6, $CHCl_3$).

Resolution of indoline **2d** with (+)-sparteine

tert-Butyl (2*S*)-2-(4-Methoxyphenyl)-2,3-dihydroindole-1-carboxylate (*S*)-**2d** and 1-*tert*-Butyl (2*R*)-2-Methyl 2-(4-Methoxyphenyl)-3*H*-indole-1,2-dicarboxylate (*R*)-**3d**

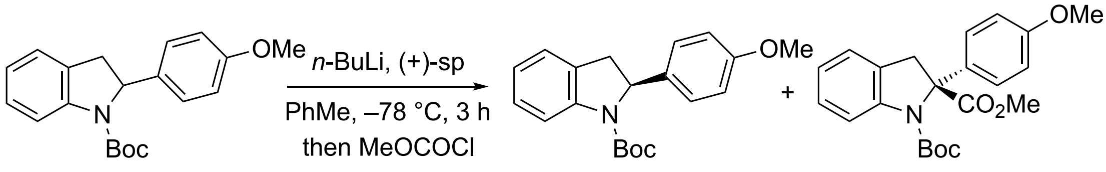

(±)-**2d** (*S*)-**2d** (*R*)-**3d**

n-BuLi (0.14 mL, 0.34 mmol, 2.4 M in hexanes) was added to a mixture of (+)-sparteine (79 mg, 0.34 mmol) and the racemic carbamate **2d** (111 mg, 0.34 mmol) in dry PhMe (8 mL) at –78 °C. After 3 h, MeOCOCl (0.07 mL, 0.85 mmol) was added and the mixture was allowed to warm to room temperature over 16 h then MeOH (1 mL) was added. The solvent was evaporated, and the residue was purified by column chromatography on silica gel, eluting with pentane–EtOAc (96:4), to give recovered carbamate (*S*)-**2d** (56 mg, 50%) as an oil; data as above (see page S-5); the enantiomeric ratio was determined to be 90:10 by CSP-HPLC as described above (major component eluted at 7.5 min); $[\alpha]_D^{25}$ –49 (1.4, $CHCl_3$), lit.[16] $[\alpha]_D^{20}$ +72.8 (0.5, $CHCl_3$) for 94% ee of *R* enantiomer. In addition, the carbamate (*R*)-**3d** (51 mg, 39%) was isolated as an oil, data as above (see page S-7); the enantiomeric ratio was determined to be 98:2 by CSP-HPLC (major component eluted at 30 min); $[\alpha]_D^{22}$ +105 (1.2, $CHCl_3$).

2.4 Preparation of enantioenriched 2,2-disubstituted products

1-*tert*-Butyl 2-Methyl (2*S*)-2-phenyl-3H-indole-1,2-dicarboxylate (*S*)-**3a**

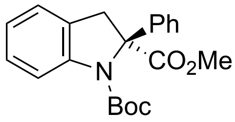

(*S*)-**3a**

n-BuLi (0.07 mL, 0.18 mmol, 2.4 M in hexane) was added to carbamate (*S*)-**2a** (43 mg, 0.15 mmol, er 90:10) in THF (1.8 mL) at –50 °C. After 5 min, MeOCOCl (0.02 mL, 0.3 mmol) was added and the mixture was allowed to warm to room temperature over 16 h then MeOH (1 mL) was added. The solvent was evaporated, and the residue was purified by column chromatography on silica gel, eluting with petrol–EtOAc (95:5), to give carbamate (*S*)-**3a** (44 mg, 85%) as an oil; data as above (see page S-5); the enantiomeric ratio was determined to be 87:13 by CSP-HPLC as described above (major component eluted at 18 min); $[\alpha]_D^{19}$ –74 (1.0, $CHCl_3$).

1-*tert*-Butyl (2*S*)-2-Methyl 2-(4-Fluorophenyl)-3*H*-indole-1,2-dicarboxylate (*S*)-**3c**

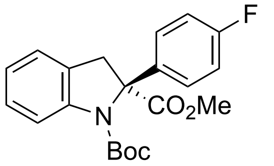

(*S*)-**3c**

n-BuLi (0.1 mL, 0.25 mmol, 2.5 M in hexane) was added to carbamate (*S*)-**2c** (65 mg, 0.21 mmol, er 92:8) in dry THF (8 mL) at –78 °C. After 20 min, MeOCOCl (0.05 mL, 0.63 mmol) was added and the mixture was allowed to warm to room temperature over 16 h then MeOH (1 mL) was added. The solvent was evaporated, and the residue was purified by column chromatography on silica gel, eluting with petrol–EtOAc (98:2), to give carbamate (*S*)-**3c** (68 mg, 87%) as an oil; data as above (see page S-7); the enantiomeric ratio was determined to be 92:8 by CSP-HPLC as described above (major component eluted at 10.9 min); $[\alpha]_D^{21}$ –84 (1.1, $CHCl_3$).

1-*tert*-Butyl (2*S*)-2-Methyl 2-(4-Methoxyphenyl)-3*H*-indole-1,2-dicarboxylate (*S*)-**3d**

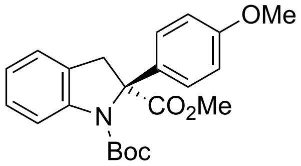

(*S*)-**3d**

n-BuLi (0.09 mL, 0.2 mmol, 2.4 M in hexanes) was added to indoline (*S*)-**2d** (56 mg, 0.17 mmol, er 90:10) in THF (2 mL) at –50 °C. After 5 min, MeOCOCl (0.03 mL, 0.34 mmol) was added. The mixture was allowed to warm to room temperature over 16 h and MeOH (1 mL) was added. The solvent was evaporated, and the residue was

purified by column chromatography on silica gel, eluting with petrol–EtOAc (90:10), to give the carbamate (*S*)-**3d** (36 mg, 55%) as an oil; data as above (see page S-7); the enantiomeric ratio was determined to be 86:14 by CSP-HPLC as described above (major component eluted at 41.2 min); $[\alpha]_D^{22}$ –51 (1.1, $CHCl_3$).

1-*tert*-Butyl 2-Benzyl (2*S*)-2-Phenyl-3H-indole-1,2-dicarboxylate (*S*)-**7a**

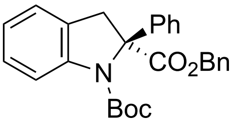

(*S*)-**7a**

n-BuLi (0.07 mL, 0.18 mmol, 2.4 M in hexanes) was added to indoline (*S*)-**2a** (43 mg, 0.15 mmol, er 90:10) in THF (1.8 mL) at –50 °C. After 5 min, BnOCOCl (0.04 mL, 0.29 mmol) was added. The mixture was allowed to warm to room temperature over 16 h and MeOH (1 mL) was added. The solvent was evaporated, and the residue was purified by column chromatography on silica gel, eluting with petrol–EtOAc (97:3), to give the carbamate (*S*)-**7a** (48 mg, 76%) as an oil; data as above (see page S-9); the enantiomeric ratio was determined to be 88:12 by CSP-HPLC as described above (major component eluted at 57 min); $[\alpha]_D^{25}$ –59 (1.2, $CHCl_3$).

(1*R*,9a*S*)-1,9a-Diphenyl-1H,9H-[1,3]oxazolo[3,4-a]indol-3-one **9aa** and (1*S*,9a*S*)-1,9a-Diphenyl-1H,9H-[1,3]oxazolo[3,4-a]indol-3-one **9ab**

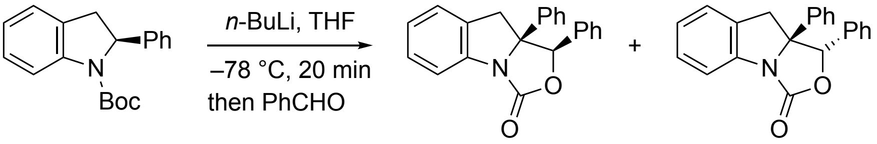

(*S*)-**2a** (*R*,*S*)-**9aa** (*S*,*S*)-**9ab**

n-BuLi (0.16 mL, 0.41 mmol, 2.5 M in hexane) was added to carbamate (*S*)-**2a** (100 mg, 0.34 mmol, er 90:10) in dry PhMe (8 mL) at –78 °C. After 20 min, PhCHO (0.1 mL, 1.0 mmol) was added and the mixture was allowed to warm to room temperature over 16 h and MeOH (1 mL) was added. The solvent was evaporated and the residue was purified by column chromatography on silica gel, eluting with petrol–EtOAc (98:2), to give carbamate (1*R*,9a*S*)-**7a** (63 mg, 63%) as an amorphous solid; data as above (see page S-11); the enantiomeric ratio was determined to be er 90:10 by CSP-HPLC; m.p. 177–179 °C; after recrystallisation from petrol–CH_2Cl_2, er 99.4:0.6, $[\alpha]_D^{21}$ –159.5 (2.2, $CHCl_3$); and the carbamate (1*S*,9a*S*)-**8a** (25 mg, 25%) as an oil; data as above (see page S-11); the enantiomeric ratio was determined to be 89:11 by CSP-HPLC.

2.5 Further functionalization

(*S*)-2-Phenyl-2,3-dihydroindole **1a**

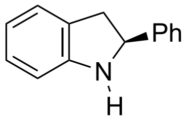

(*S*)-**1a**

Trifluoroacetic acid (0.09 mL, 1.2 mmol) was added to the carbamate (*S*)-**2a** (35 mg, 0.12 mmol) in CH_2Cl_2 (1.2 mL) at 0 °C then the mixture was warmed to room temp. After 16 h, the solvent was evaporated and the residue was purified by column chromatography on silica gel, eluting with petrol−EtOAc (97:3), to give amine (*S*)-**1a** (23 mg, quantitative yield) as an amorphous solid; m.p. 46−48 °C [petrol−EtOAc], lit.[19] 42−43 °C; ^{1}H NMR (400 MHz, $CDCl_3$) δ = 7.48–7.40 (2H, m, 2 × CH), 7.39–7.32 (2H, m, 2 × CH), 7.31–7.24 (1H, m, CH), 7.15–7.04 (2H, m, 2 × CH), 6.75 (1H, t, *J* = 7.5 Hz, CH), 6.68 (1H, d, *J* = 7.5 Hz, CH), 4.96 (1H, t, *J* = 9 Hz, CH), 4.15 (1H, br s, NH), 3.45 (1H, dd, *J* = 15.5, 9 Hz, CH), 3.00 (1H, dd, *J* = 15.5, 9 Hz, CH); ^{13}C NMR (100 MHz, $CDCl_3$) δ = 151.0, 144.6, 128.6, 128.1, 127.6, 127.4, 126.3, 124.6, 118.8, 108.9, 63.6, 39.6; $[\alpha]_D^{21}$ –60 (0.5, $CHCl_3$). Data consistent with the literature.[19] The enantiomeric ratio was determined to be 90:10 by CSP-HPLC (major component eluted at 14.0 min) using a Beckman system fitted with a Cellulose-1 column (250 mm × 4.60 mm i.d.) as the stationary phase with a mixture of *n*-hexane:isopropanol (85:15 v/v) as the mobile phase at a flow rate of 1 mL·min^{-1}; ambient temperature, detection by UV absorbance at 254 nm. Injection volume was 20 μL of the sample prepared in a 2 g·L^{-1} solution of the eluent. Under these conditions, the components were eluted at 14.2 min and 25.5 min.

(±)-2-Benzyl 2-Phenyl-3*H*-indole-2-carboxylate **10a**

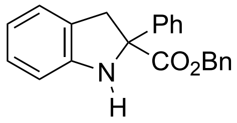

(±)-**10a**

Trifluoroacetic acid (0.18 mL, 2.3 mmol) was added to the carbamate (±)-**7a** (100 mg, 0.23 mmol) in CH_2Cl_2 (2.4 mL) at 0 °C then the mixture was warmed to room temp. After 16 h, the solvent was evaporated and the residue was purified by column chromatography on silica gel, eluting with petrol−EtOAc (95:5), to give amine (±)-**10a** (72 mg, 95%) as an amorphous solid; m.p. 110−112 °C [petrol−EtOAc]; R_f 0.5 [petrol–EtOAc (80:10)]; FT-IR v_{max} (ATR)/cm^{-1} 3390, 3070, 3025, 2965, 2925, 2905, 1725, 1610, 1485, 1405, 1255, 1230, 1170, 1070, 1035, 880; ^{1}H NMR (400 MHz, $CDCl_3$) δ = 7.59–7.53 (2H, m, 2 × CH), 7.39– 7.29 (6H, m, 6 × CH), 7.21–7.14 (2H, m, 2 × CH), 7.14–7.07 (2H, m, 2 × CH), 6.83–6.74 (2H, m, 2 × CH), 5.21–5.09 (3H, m, NH & CH_2), 4.14 (1H, d, *J* = 16 Hz, CH), 3.30 (1H, d, *J* = 16 Hz, CH); ^{13}C NMR (100 MHz, $CDCl_3$,

one signal could not be observed) δ = 174.6, 149.3, 141.9, 135.3, 128.6, 128.5, 128.3, 127.8, 127.75, 126.6, 125.7, 124.5, 119.6, 109.8, 73.7, 67.6, 41.7; HRMS *m/z* (ES) Found: MH^+, 330.1504. $C_{22}H_{19}NO_2$ requires MH^+ 330.1489; LRMS *m/z* (ES) 330 (100%).
Resolution between the enantiomers of indoline **10a** was achieved using a Beckman system fitted with a Cellulose-1 column (250 mm × 4.60 mm i.d.) as the stationary phase with a mixture of *n*-hexane:isopropanol (85:15 v/v) as the mobile phase at a flow rate of 0.7 mL·min–1; ambient temperature, detection by UV absorbance at 254 nm. Injection volume was 20 μL of the sample prepared in a 2 g·L–1 solution of the eluent. Under these conditions, the components were eluted at 9.5 min and 10.7 min.

(*S*)-2-Benzyl 2-Phenyl-3*H*-indole-2-carboxylate **10a**

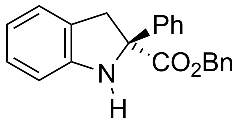

(*S*)-**10a**

Trifluoroacetic acid (0.08 mL, 1.1 mmol) was added to the carbamate (*S*)-**7a** (46 mg, 0.11 mmol) in CH_2Cl_2 (1.1 mL) at 0 °C then the mixture was warmed to room temp. After 16 h, the solvent was evaporated and the residue was purified by column chromatography on silica gel, eluting with petrol−EtOAc (90:10), to give amine (*S*)-**10a** (33 mg, 91%) as an amorphous solid; m.p. 108−110 °C [petrol−EtOAc]; data as above; the enantiomeric ratio was determined to be 86:14 by CSP-HPLC as described above (major component eluted at 9.4 min); $[\alpha]_D^{21}$ –108 (0.8, $CHCl_3$).

(±)-1-*tert*-Butyl 2-Phenyl-3*H*-indole-1,2-dicarboxylate **11a**

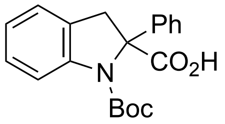

(±)-**11a**

To a solution of carbamate (±)-**7a** (165 mg, 0.38 mmol) in MeOH (13 mL) was added 10% Pd/C (110 mg, 0.1 mmol) and The mixture was stirred at room temp. under a hydrogen gas atmosphere (1 atm). After 16 h, the mixture was filtered and the solvent was evaporated to give the acid (±)-**11a** (127 mg, 97%) as an oil; FT-IR ν_{max} (ATR)/cm^{-1} 3395, 2980, 2935, 2860, 1700, 1675, 1600, 1490, 1370, 1320, 1245, 1170, 1145, 1015, 855; ^{1}H NMR (400 MHz, DMSO-d_6, one CH overlapping with water signal) δ = 13.32 (1H, br s, OH), 7.87 (1H, br s, CH), 7.44–7.36 (2H, m, 2 × CH), 7.32 (2H, t, *J* = 7.5 Hz, 2 × CH), 7.28–7.19 (2H, m, 2 × CH), 7.13 (1H, d, *J* = 7.5 Hz, CH), 6.97 (1H, t, *J* = 7.5 Hz, CH), 3.84 (1H, d, *J* = 16.5 Hz, CH), 1.23 (9H, br s, *t*-Bu); ^{13}C NMR (100 MHz, DMSO-d_6, four quaternary carbon signals not observed) δ = 173.1, 151.9, 128.1, 128.0, 127.4, 127.1, 125.0, 123.2, 114.8, 73.4, 46.3, 28.1; HRMS *m/z* (ES) Found:

MNa^+ 362.1380. $C_{20}H_{21}NO_4Na$ requires MNa^+ 362.1363; LRMS *m/z* (ES) 160 (15%), 182 (30%), 240 (50%), 284 (50%), 303 (15%), 362 (100%).

(2*S*)-1-*tert*-Butyl 2-Phenyl-3*H*-indole-1,2-dicarboxylate **11a**

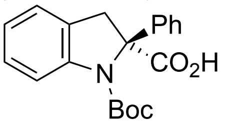

(*S*)-**11a**

To a solution of carbamate (*S*)-**7a** (40 mg, 0.09 mmol) in MeOH (3 mL) was added 10% Pd/C (20 mg, 0.02 mmol). The mixture was stirred at room temp. under a hydrogen gas atmosphere (1 atm). After 16 h, the mixture was filtered and the solvent was evaporated to give the acid (*S*)-**11a** (31 mg, 97% yield) as an oil; data as above; $[\alpha]_D^{22}$ –73 (1.0, MeOH).

3. Variable temperature ^{1}H NMR spectra for carbamate **2a**

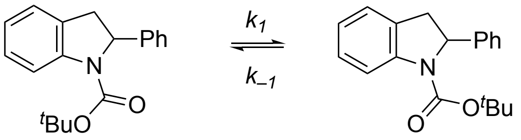

ratio 1 : 6
so $k_1 = 6k_{-1}$

Coalescence of signals in the ^{1}H NMR spectrum of carbamate **2a** in D_8-THF was followed by taking spectra at various temperatures. The ratio of the rotamers is ~ 1:6. The ^{1}H NMR spectra in the region 3.00–0.70 ppm (the peak at 1.7 ppm corresponds to residual THF) are shown below:

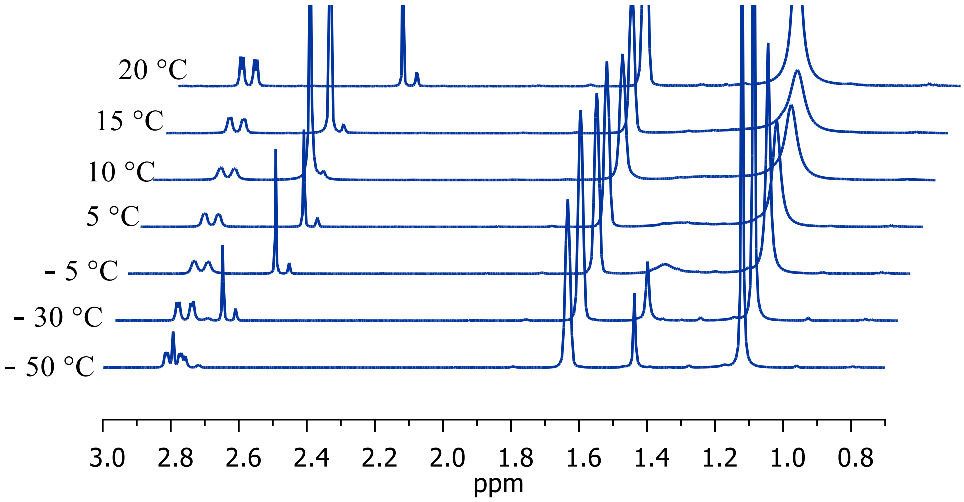

By using line shape analysis by DNMR (with the software iNMR) on the *t*-Bu peaks (~1.4 and 1.1 ppm), the rate constants k_1 that fit best by overlaying the simulated spectra with the spectroscopic data can be estimated as follows:

T/K	1/T	k_1	ln(k_1/T)		k_{-1}	ln(k_{-1}/T)
243	0.004115	5	-3.88362		0.8333	-5.67538
253	0.003953	20	-2.53766		3.3333	-4.32942
258	0.003876	38	-1.91537		6.3333	-3.70713
263	0.003802	75	-1.25467		12.5	-3.04643
268	0.003731	125	-0.76267		20.8333	-2.55443
273	0.003663	185	-0.38912		30.8333	-2.18088
278	0.003597	256	-0.08244		42.6667	-1.87420
283	0.003534	427	0.411337		71.1667	-1.38042
288	0.003472	711	0.903712		118.5	-0.88805
298	0.003356	1387	1.537805		231.167	-0.25395
303	0.003300	1748	1.752495		291.333	-0.03926
323	0.003096	4347	2.599589		724.5	0.80783

Table S-1. Line shape analysis from VT-NMR spectroscopy of carbamate ***2a****.*

Eyring plot for minor rotamer to major rotamer (k_1):

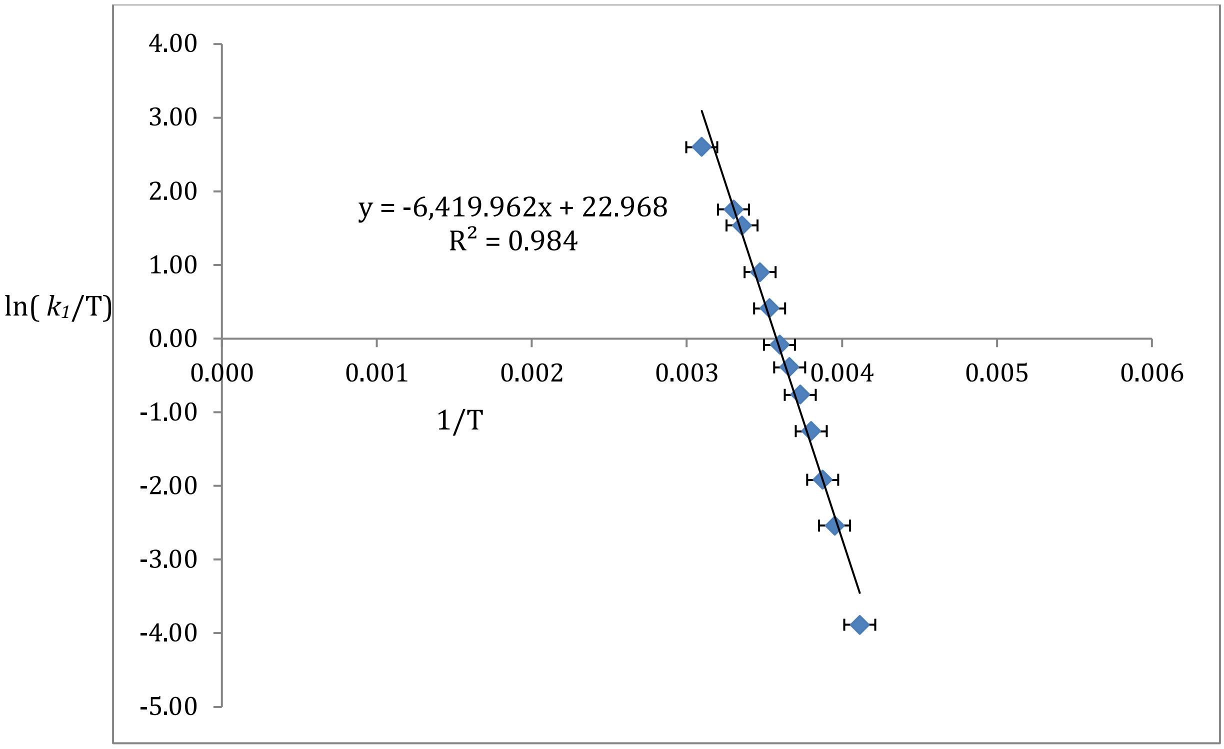

Eyring plot for major rotamer to minor rotamer (k_{-1}):

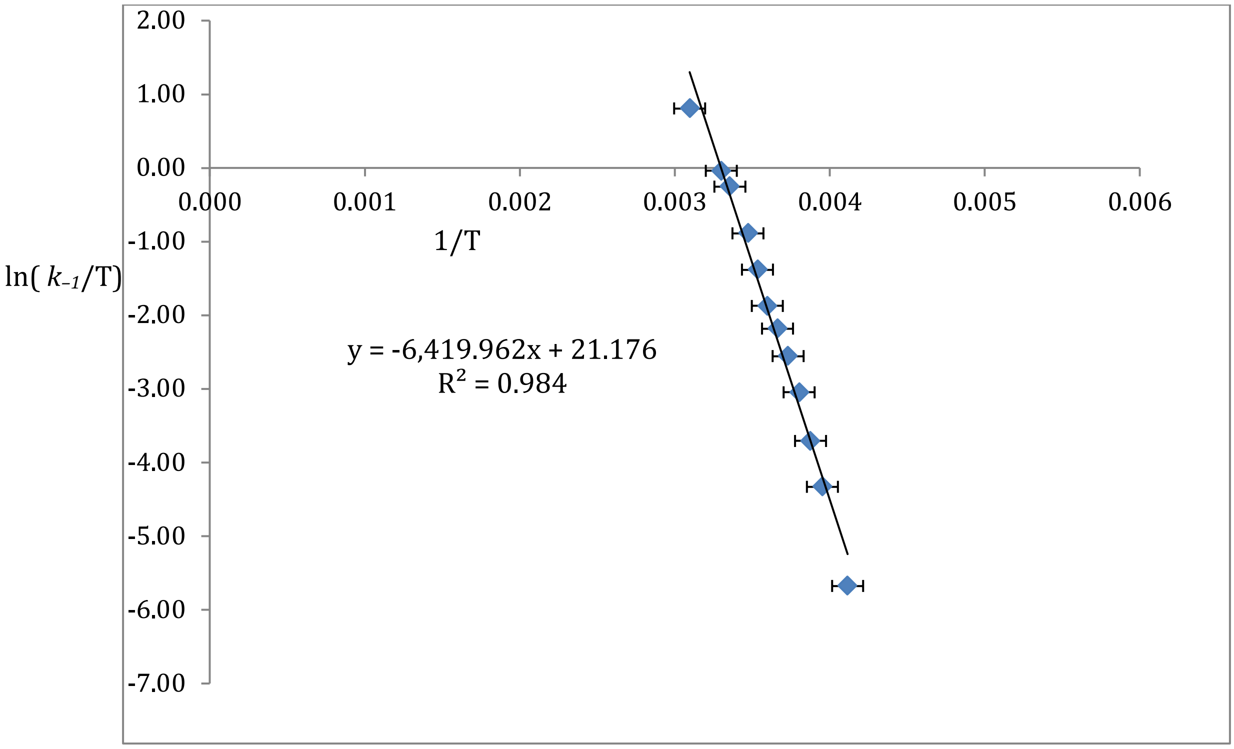

By using the Eyring equation, these plots gave the following data:

Forward direction (minor to major rotamer, k_1):
slope –6419.96, intercept 22.97
Approximate activation parameters for Boc rotation in THF:
$\Delta H^{\ddagger}$ 53.4 kJ/mol
$\Delta S^{\ddagger}$ –6.6 J/K·mol.
Hence the barrier to rotation $\Delta G^{\ddagger} \approx$ 54.7 kJ/mol at –78 °C.
The half-life for rotation is about 75 sec at –78 °C.

Reverse direction (major to minor rotamer, k_{-1}):
slope –6419.96, intercept 21.18
Approximate activation parameters for Boc rotation in THF:
$\Delta H^{\ddagger}$ 53.4 kJ/mol
$\Delta S^{\ddagger}$ –21.5 J/K·mol.
Hence the barrier to rotation $\Delta G^{\ddagger} \approx$ 57.6 kJ/mol at –78 °C.
The half-life for rotation is about 449 sec (7.5 min) at –78 °C.

4. Variable temperature ^{1}H NMR spectra for carbamate **2d**

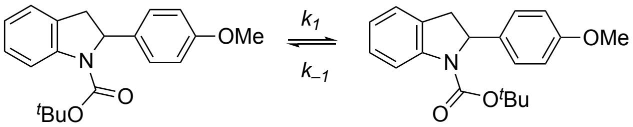

ratio 1 : 6
so $k_1 = 6k_{-1}$

Coalescence of signals in the ^{1}H NMR spectrum of carbamate **2d** in D$_8$-THF was followed by taking spectra at various temperatures. The ratio of the rotamers is ~ 1:6. The ^{1}H NMR spectra in the region 3.0–1.0 ppm (the peak at 1.7 ppm corresponds to residual THF) are shown below:

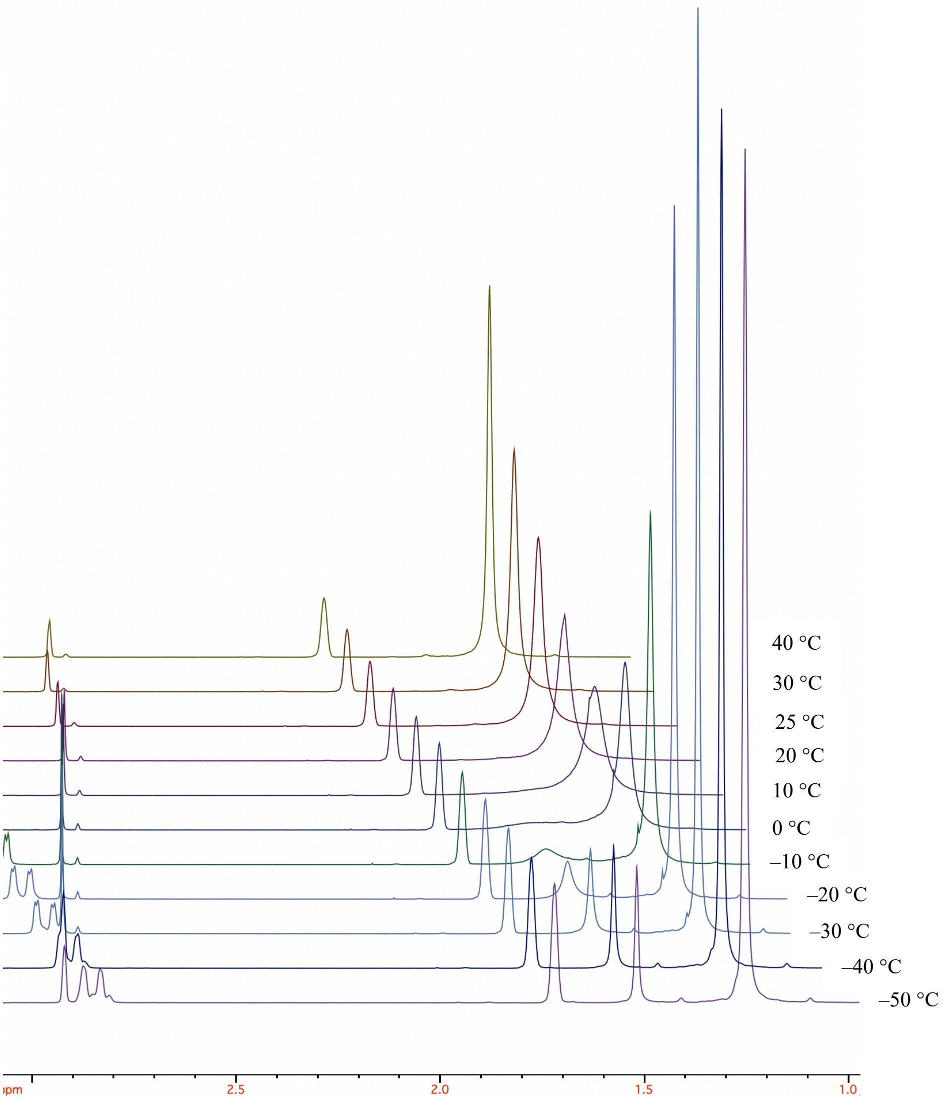

By using line shape analysis by DNMR (with the software iNMR) on the *t*-Bu peaks (~1.4 and 1.1 ppm), the rate constants k_1 that fit best by overlaying the simulated spectra with the spectroscopic data can be estimated as follows:

T/K	1/T	k_1	ln(k_1/T)		k_{-1}	ln(k_{-1}/T)
223	0.004484	0.6	-5.918		0.1	-7.70976
233	0.004292	1.1	-5.35573		0.1833	-7.14749
243	0.004115	5	-3.88362		0.8333	-5.67538
253	0.003953	23	-2.3979		3.8333	-4.18965
263	0.003802	77	-1.22835		12.833	-3.02011
273	0.003663	360	0.276632		60	-1.51513
283	0.003534	460	0.48578		76.667	-1.30598
293	0.003413	725	0.905999		120.83	-0.88576
298	0.003356	1380	1.532745		230	-0.25901
303	0.003300	2360	2.052684		393.33	0.26092
313	0.003195	6640	3.054664		1106.67	1.26290

Table S-2. Line shape analysis from VT-NMR spectroscopy of carbamate ***2d****.*

Eyring plot for minor rotamer to major rotamer (k_1):

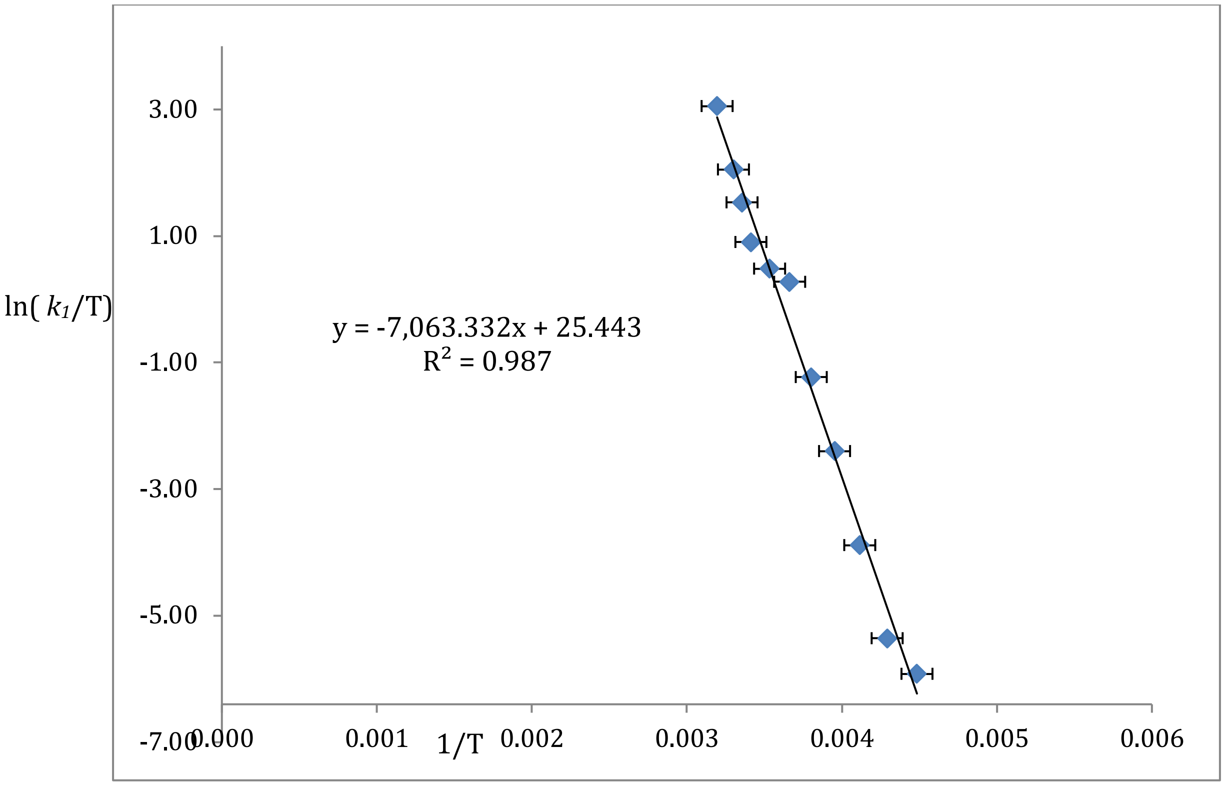

Eyring plot for major rotamer to minor rotamer (k_{-1}):

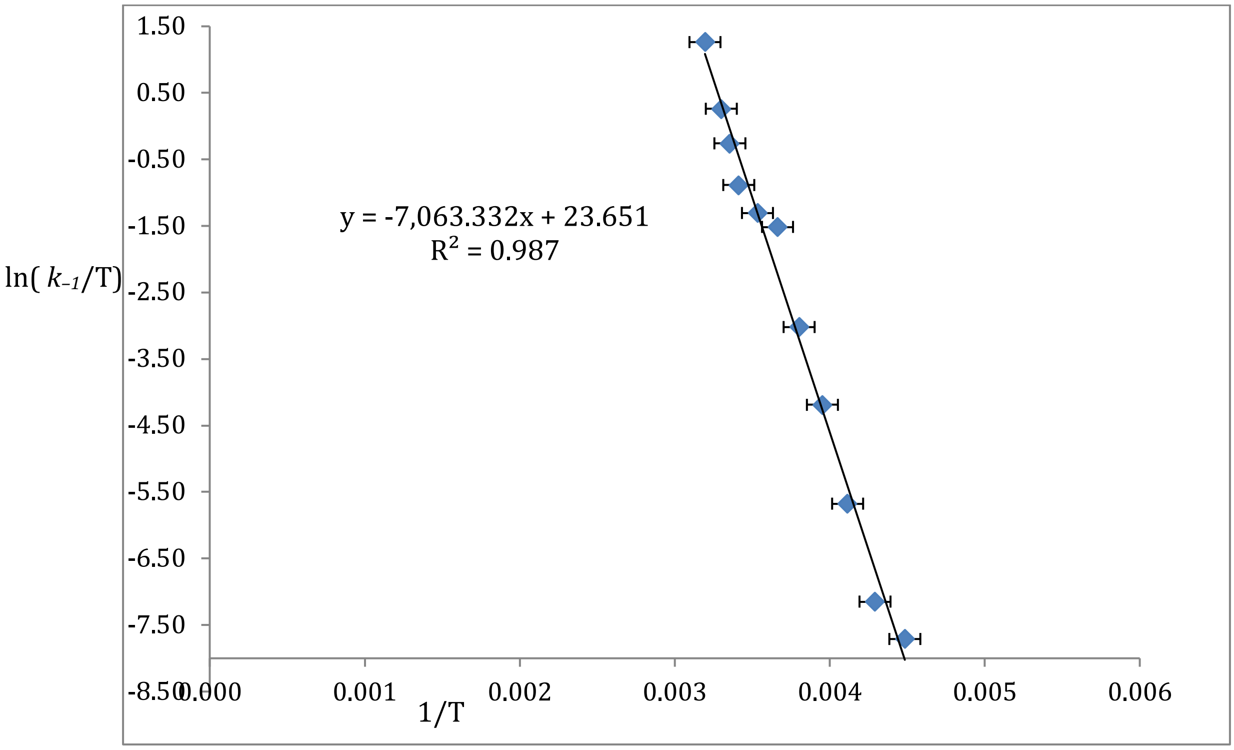

By using the Eyring equation, these plots gave the following data:

Forward direction (minor to major rotamer, k_1):
slope –7063.33, intercept 25.44
Approximate activation parameters for Boc rotation in THF:
$\Delta H^{\ddagger}$ 58.7 kJ/mol
$\Delta S^{\ddagger}$ 14.0 J/K·mol.
Hence the barrier to rotation $\Delta G^{\ddagger} \approx$ 56.0 kJ/mol at –78 °C.
The half-life for rotation is about 171 sec at –78 °C.

Reverse direction (major to minor rotamer, k_{-1}):
slope –7063.33, intercept 23.65
Approximate activation parameters for Boc rotation in THF:
$\Delta H^{\ddagger}$ 58.7 kJ/mol
$\Delta S^{\ddagger}$ –0.9 J/K·mol.
Hence the barrier to rotation $\Delta G^{\ddagger} \approx$ 58.9 kJ/mol at –78 °C.
The half-life for rotation is about 1024 sec (17 min) at –78 °C.

5. X-ray data for carbamate (*S*)-**2a**

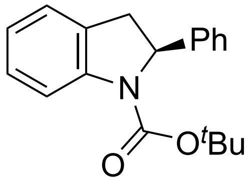

(*S*)-**2a**

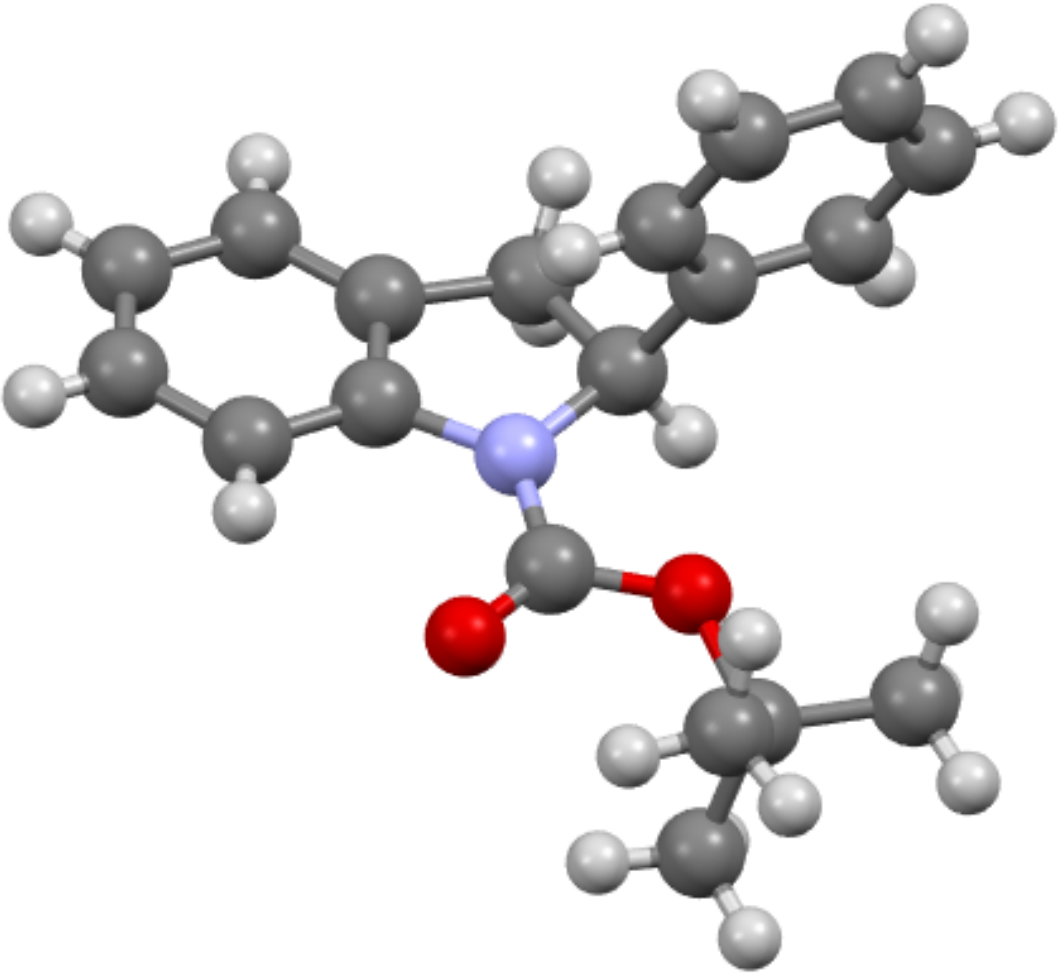

CCDC 2063461

Table S3 Crystal data and structure refinement for (*S*)-**2a** (OIC310v).

Identification code	OIC310v
Empirical formula	$C_{19}H_{21}NO_2$
Formula weight	295.37
Temperature/K	100.04

Crystal system	orthorhombic
Space group	$P2_12_12_1$
a/Å	5.8602(9)
b/Å	16.445(2)
c/Å	16.445
α/°	90
β/°	90
γ/°	90
Volume/Å^3	1584.7(3)
Z	4
ρ_{calc}g/cm^3	1.238
μ/mm^{-1}	0.632
F(000)	632.0
Crystal size/mm^3	0.25 × 0.23 × 0.19
Radiation	CuKα (λ = 1.54178)
2Θ range for data collection/°	7.602 to 135.514
Index ranges	-7 ≤ h ≤ 6, -19 ≤ k ≤ 19, -19 ≤ l ≤ 19
Reflections collected	59531
Independent reflections	2819 [R_{int} = 0.0784, R_{sigma} = 0.0230]
Data/restraints/parameters	2819/0/202
Goodness-of-fit on F^2	1.170
Final R indexes [I>=2σ (I)]	R_1 = 0.0312, wR_2 = 0.0761
Final R indexes [all data]	R_1 = 0.0321, wR_2 = 0.0775
Largest diff. peak/hole / e Å^{-3}	0.18/-0.26
Flack parameter	0.15(6)

Table S-4 Fractional Atomic Coordinates (×10^4) and Equivalent Isotropic Displacement Parameters (Å^2×10^3) for OIC310v. U_{eq} is defined as 1/3 of of the trace of the orthogonalised U_{IJ} tensor.

**Atom**	***x***	***y***	***z***	**U(eq)**
O1	6606(2)	5052.5(8)	4199.2(7)	22.3(3)
O2	4262(2)	4218.1(7)	3465.0(8)	21.1(3)
N1	3610(3)	5547.5(9)	3461.7(9)	18.2(3)
C1	1710(3)	5400.2(11)	2888.9(10)	18.0(4)
C2	328(3)	6207.2(11)	2911.3(11)	21.4(4)
C3	1837(3)	6793.7(11)	3362.9(10)	18.4(4)
C4	1553(3)	7620.2(11)	3500.8(11)	21.5(4)
C5	3198(4)	8039.8(11)	3943.3(11)	23.3(4)
C6	5080(4)	7627.9(11)	4253.6(11)	22.5(4)
C7	5373(3)	6791.8(11)	4129.2(10)	19.6(4)
C8	3731(3)	6384.8(11)	3676.1(10)	17.2(4)

C9	2553(3)	5197.7(10)	2044.4(10)	16.8(4)
C10	1161(3)	4739.7(11)	1537.1(11)	21.2(4)
C11	1808(4)	4578.5(12)	745.6(11)	25.4(4)
C12	3858(4)	4871.8(12)	451.2(11)	26.4(4)
C13	5274(4)	5325.5(13)	953.5(11)	25.5(4)
C14	4614(3)	5484.7(12)	1749.8(11)	21.4(4)
C15	4979(3)	4941.5(11)	3752.9(10)	18.0(4)
C16	5225(3)	3445.7(11)	3768.7(12)	22.3(4)
C17	4590(4)	3351.0(12)	4657.5(12)	29.3(5)
C18	7782(3)	3405.0(12)	3632.0(13)	27.8(4)
C19	4007(4)	2814.4(12)	3247.9(14)	28.6(5)

Table S-5 Anisotropic Displacement Parameters ($Å^2 \times 10^3$) for OIC310v. The Anisotropic displacement factor exponent takes the form: $-2\pi^2[h^2a^{*2}U_{11}+2hka^*b^*U_{12}+\ldots]$.

**Atom**	**U_{11}**	**U_{22}**	**U_{33}**	**U_{23}**	**U_{13}**	**U_{12}**
O1	24.6(7)	22.2(7)	20.0(6)	-0.5(5)	-4.6(5)	0.4(6)
O2	25.7(7)	15.5(6)	21.9(6)	-0.4(5)	-4.6(5)	0.2(5)
N1	22.3(8)	16.3(7)	16.0(7)	-1.4(6)	-2.9(6)	-0.5(6)
C1	18.8(9)	18.8(9)	16.4(8)	-0.3(7)	-2.0(7)	-1.9(7)
C2	20.6(10)	22.6(9)	20.9(9)	-3.3(7)	-1.9(8)	2.0(7)
C3	19.6(9)	21.3(9)	14.3(8)	0.9(7)	4.2(7)	0.3(7)
C4	24.2(9)	21.4(9)	18.8(9)	0.7(7)	4.1(8)	2.0(8)
C5	31.9(11)	16.5(9)	21.6(9)	-1.0(7)	3.6(8)	-0.4(8)
C6	28.4(11)	22.3(10)	16.9(8)	-1.9(7)	1.5(8)	-2.9(8)
C7	23.7(10)	22.0(9)	13.0(8)	-0.1(7)	-0.3(7)	-1.6(7)
C8	22.5(9)	17.2(9)	11.9(8)	0.3(6)	5.3(7)	-0.6(7)
C9	20.4(9)	14.2(8)	15.7(8)	1.0(7)	-2.0(7)	2.6(7)
C10	21.3(9)	20.3(9)	22.1(9)	-0.8(7)	-4.4(7)	-0.7(7)
C11	30.8(11)	26.2(10)	19.2(9)	-3.1(8)	-6.6(8)	3.1(8)
C12	35.0(12)	28.4(10)	15.7(9)	1.1(7)	1.3(8)	8.8(8)
C13	24.7(10)	29.9(11)	22.0(9)	6.3(8)	2.9(8)	2.7(8)
C14	21.5(9)	22.7(10)	20.2(9)	1.1(7)	-3.2(7)	-2.6(8)
C15	22.1(9)	18.5(9)	13.3(7)	0.4(7)	1.7(7)	-0.6(8)
C16	23.9(10)	15.7(9)	27.2(9)	2.2(7)	1.4(8)	3.3(8)
C17	33.2(11)	25.0(10)	29.8(10)	8.0(8)	2.1(9)	1.2(8)
C18	22.9(10)	25.4(10)	35.3(11)	-0.8(9)	2.4(8)	2.0(8)
C19	24.9(11)	19.5(10)	41.3(12)	-5.4(8)	1.8(9)	0.0(8)

Table S-6 Bond Lengths for OIC310v.

Atom	Atom	Length/Å	Atom	Atom	Length/Å
O1	C15	1.217(2)	C5	C6	1.391(3)
O2	C15	1.348(2)	C6	C7	1.401(3)
O2	C16	1.477(2)	C7	C8	1.389(3)
N1	C1	1.478(2)	C9	C10	1.389(3)
N1	C8	1.423(2)	C9	C14	1.384(3)
N1	C15	1.366(2)	C10	C11	1.381(3)
C1	C2	1.555(3)	C11	C12	1.382(3)
C1	C9	1.511(2)	C12	C13	1.388(3)
C2	C3	1.505(3)	C13	C14	1.390(3)
C3	C4	1.388(3)	C16	C17	1.516(3)
C3	C8	1.396(3)	C16	C18	1.517(3)
C4	C5	1.391(3)	C16	C19	1.523(3)

Table S-7 Bond Angles for OIC310v.

Atom	Atom	Atom	Angle/°	Atom	Atom	Atom	Angle/°
C15	O2	C16	121.42(14)	C10	C9	C1	118.65(16)
C8	N1	C1	110.73(14)	C14	C9	C1	122.12(16)
C15	N1	C1	123.10(14)	C14	C9	C10	119.16(17)
C15	N1	C8	126.13(15)	C11	C10	C9	120.59(18)
N1	C1	C2	103.73(13)	C10	C11	C12	120.11(19)
N1	C1	C9	112.05(15)	C11	C12	C13	119.92(18)
C9	C1	C2	112.34(14)	C12	C13	C14	119.68(19)
C3	C2	C1	104.63(15)	C9	C14	C13	120.54(18)
C4	C3	C2	129.64(17)	O1	C15	O2	126.05(16)
C4	C3	C8	120.44(17)	O1	C15	N1	124.20(16)
C8	C3	C2	109.92(16)	O2	C15	N1	109.74(15)
C3	C4	C5	119.22(18)	O2	C16	C17	108.70(15)
C6	C5	C4	119.98(18)	O2	C16	C18	111.43(16)
C5	C6	C7	121.46(18)	O2	C16	C19	102.53(15)
C8	C7	C6	117.78(17)	C17	C16	C18	112.37(18)
C3	C8	N1	109.57(16)	C17	C16	C19	110.92(16)
C7	C8	N1	129.32(16)	C18	C16	C19	110.46(16)
C7	C8	C3	121.11(17)				

Table S-8 Hydrogen Atom Coordinates (Å×10^4) and Isotropic Displacement Parameters (Å^2×10^3) for OIC310v.

**Atom**	***x***	***y***	***z***	**U(eq)**
H1	739.26	4945.01	3096.03	22
H2A	13.48	6405.11	2353.92	26
H2B	-1139.63	6130.74	3199.67	26
H4	250.6	7896.1	3295.47	26
H5	3036.96	8607.54	4033.57	28
H6	6189.75	7920.13	4556.59	27
H7	6653.35	6512.38	4347.41	23
H10	-248.72	4535.45	1736.2	25
H11	842.37	4265.48	403.08	31
H12	4297.36	4763.02	-94.11	32
H13	6687.46	5526.25	753.9	31
H14	5585.97	5792.91	2094.62	26
H17A	5344.68	3776.05	4977.22	44
H17B	5083.69	2815.57	4851.24	44
H17C	2932.59	3400.21	4718.52	44
H18A	8131.57	3569.46	3072.96	42
H18B	8314.1	2847.21	3721.94	42
H18C	8552.52	3771.96	4012.93	42
H19A	2355.04	2860.43	3329.31	43
H19B	4512.47	2268.72	3405.73	43
H19C	4368.69	2907.85	2673.66	43

6. X-ray data for carbamate (*R,S*)-**9aa**

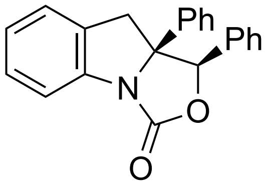

(*R*,*S*)-**9aa**

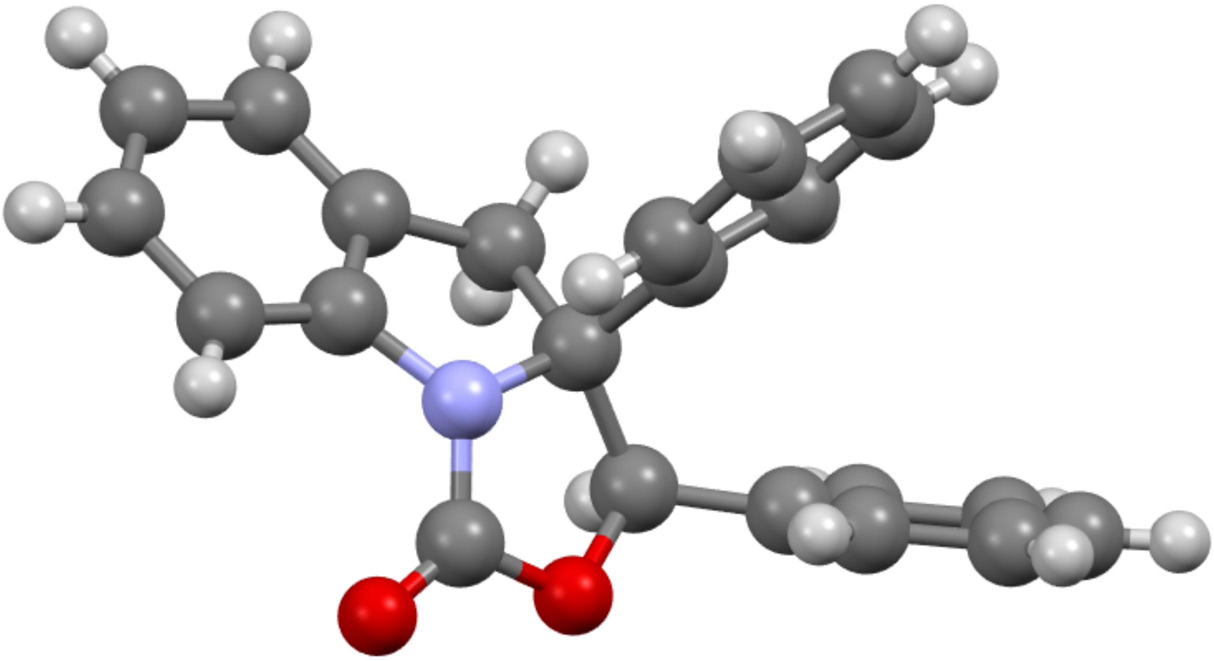

CCDC 2063462

Table S-9 Crystal data and structure refinement for (*R,S*)-**9aa** (OIC312v_0m).

Identification code	OIC312v_0m
Empirical formula	$C_{22}H_{17}NO_2$
Formula weight	327.36
Temperature/K	100.03
Crystal system	monoclinic
Space group	$P2_1$

a/Å	11.0623(10)
b/Å	6.2201(6)
c/Å	12.3625(11)
α/°	90
β/°	104.795(3)
γ/°	90
Volume/Å^3	822.44(13)
Z	2
ρ_{calc}g/cm^3	1.322
μ/mm^{-1}	0.674
F(000)	344.0
Crystal size/mm^3	0.3 × 0.25 × 0.23
Radiation	CuKα (λ = 1.54178)
2Θ range for data collection/°	7.396 to 133.466
Index ranges	-13 ≤ h ≤ 13, -7 ≤ k ≤ 7, -13 ≤ l ≤ 14
Reflections collected	19740
Independent reflections	2890 [R_{int} = 0.0457, R_{sigma} = 0.0274]
Data/restraints/parameters	2890/1/226
Goodness-of-fit on F^2	1.087
Final R indexes [I>=2σ (I)]	R_1 = 0.0285, wR_2 = 0.0698
Final R indexes [all data]	R_1 = 0.0292, wR_2 = 0.0703
Largest diff. peak/hole / e Å^{-3}	0.15/-0.21
Flack parameter	0.01(7)

Table S-10 Fractional Atomic Coordinates (×10^4) and Equivalent Isotropic Displacement Parameters (Å^2×10^3) for OIC312v_0m. U_{eq} is defined as 1/3 of of the trace of the orthogonalised U_{IJ} tensor.

Atom	*x*	*y*	*z*	U(eq)
O1	5438.6(12)	976(2)	530.9(11)	18.4(3)
O2	6785.8(12)	3674(2)	1169.2(11)	17.5(3)
N1	5053.0(14)	3544(3)	1753.3(12)	14.5(3)
C1	5705.8(17)	2586(3)	1087.4(15)	15.0(4)
C2	6719.5(17)	5793(3)	1665.0(15)	15.5(4)
C3	5645.0(17)	5540(3)	2279.8(15)	14.7(4)
C4	4567.7(17)	7217(3)	1983.3(15)	15.8(4)
C5	3410.9(17)	5802(3)	1733.2(14)	15.6(4)
C6	2161.9(19)	6314(3)	1617.4(17)	19.9(4)
C7	1257.7(18)	4722(4)	1283.9(17)	22.6(4)
C8	1590.9(19)	2669(4)	1032.9(16)	21.2(4)
C9	2845.8(18)	2121(3)	1151.4(15)	17.9(4)
C10	3727.5(17)	3709(3)	1525.5(14)	15.2(4)
C11	8001.6(18)	6377(3)	2351.1(16)	17.6(4)

C12	8478(2)	8423(4)	2250.6(18)	24.5(5)
C13	9638(2)	9001(4)	2931(2)	34.2(6)
C14	10323(2)	7570(5)	3701(2)	34.7(6)
C15	9870(2)	5510(4)	3780.1(18)	29.6(5)
C16	8713.2(18)	4924(4)	3105.6(17)	21.8(4)
C17	6090.3(17)	5319(3)	3550.6(15)	14.9(4)
C18	5920.3(17)	3451(3)	4101.3(16)	16.8(4)
C19	6264.6(18)	3355(4)	5267.3(16)	20.0(4)
C20	6786.4(18)	5131(4)	5883.0(16)	20.5(4)
C21	6975.8(17)	7016(3)	5343.4(16)	19.8(4)
C22	6618.5(17)	7113(3)	4184.2(17)	18.3(4)

Table S-11 Anisotropic Displacement Parameters (Å^2×10^3) for OIC312v_0m. The Anisotropic displacement factor exponent takes the form: -2π^2[h^{2}a*^{2}U$_{11}$+2hka*b*U$_{12}$+…].

Atom	U_{11}	U_{22}	U_{33}	U_{23}	U_{13}	U_{12}
O1	23.7(7)	16.3(7)	15.4(6)	-2.3(5)	5.3(5)	-0.8(6)
O2	17.8(7)	16.4(7)	20.3(7)	-3.9(6)	8.3(5)	-2.4(6)
N1	14.9(8)	14.8(8)	13.8(7)	-0.5(6)	3.4(6)	-0.4(6)
C1	15.9(9)	15.1(9)	13.5(9)	4.0(7)	2.9(7)	0.4(8)
C2	18.8(9)	13.6(9)	15.1(9)	-0.2(7)	6.3(7)	0.5(8)
C3	15.9(9)	12.8(9)	15.4(9)	-0.2(7)	4.3(7)	-0.9(7)
C4	16.4(9)	16.4(9)	15.2(9)	0.0(7)	4.9(7)	1.1(8)
C5	17.8(9)	17.3(10)	11.9(8)	1.5(7)	3.8(7)	-0.2(8)
C6	19.7(10)	20.9(11)	19.1(9)	0.5(8)	4.8(7)	2.7(8)
C7	15.5(9)	29.6(11)	22.3(10)	1.7(9)	3.9(8)	1.2(8)
C8	17.0(9)	24.5(11)	19.9(10)	1.5(8)	0.8(7)	-4.7(8)
C9	19.9(9)	17.4(9)	16.3(9)	1.7(8)	4.5(7)	-1.5(8)
C10	14.7(9)	20.2(10)	10.9(9)	2.0(8)	3.3(7)	0.7(8)
C11	17.7(9)	19.2(10)	18.7(10)	-3.6(8)	9.7(8)	-1.5(7)
C12	23.9(11)	21.2(11)	32.4(12)	-3.1(9)	14.3(9)	-2.4(9)
C13	28.0(12)	29.7(13)	50.4(15)	-14.3(11)	20.0(11)	-13.2(10)
C14	16.8(10)	53.2(16)	34.7(12)	-16.2(12)	7.5(9)	-9.4(11)
C15	17.4(10)	46.1(15)	25.6(11)	1.1(10)	6.2(8)	3.8(10)
C16	17.3(10)	28.4(11)	21.5(10)	0.7(8)	7.9(8)	1.1(8)
C17	10.8(8)	20.3(9)	14.3(9)	-0.3(7)	4.5(7)	1.4(7)
C18	13.8(9)	18.4(10)	18.4(9)	-0.4(8)	4.6(7)	0.3(8)
C19	17.8(10)	23.2(10)	20.3(10)	5.8(8)	7.1(8)	4.8(8)
C20	15.7(9)	31.5(11)	14.4(9)	0.3(8)	4.2(7)	5.5(8)
C21	14.4(9)	25.8(11)	18.6(10)	-6.8(8)	2.9(7)	-0.3(8)
C22	15.6(9)	20.0(10)	19.8(10)	-1.2(8)	5.5(7)	1.1(8)

Table S-12 Bond Lengths for OIC312v_0m.

Atom	Atom	Length/Å	Atom	Atom	Length/Å
O1	C1	1.208(2)	C8	C9	1.400(3)
O2	C1	1.354(2)	C9	C10	1.382(3)
O2	C2	1.463(2)	C11	C12	1.395(3)
N1	C1	1.363(3)	C11	C16	1.390(3)
N1	C3	1.475(2)	C12	C13	1.390(3)
N1	C10	1.424(2)	C13	C14	1.380(4)
C2	C3	1.574(2)	C14	C15	1.388(4)
C2	C11	1.500(3)	C15	C16	1.386(3)
C3	C4	1.556(3)	C17	C18	1.384(3)
C3	C17	1.528(2)	C17	C22	1.402(3)
C4	C5	1.518(3)	C18	C19	1.395(3)
C5	C6	1.389(3)	C19	C20	1.381(3)
C5	C10	1.389(3)	C20	C21	1.391(3)
C6	C7	1.393(3)	C21	C22	1.387(3)
C7	C8	1.386(3)			

Table S-13 Bond Angles for OIC312v_0m.

Atom	Atom	Atom	Angle/°	Atom	Atom	Atom	Angle/°
C1	O2	C2	110.11(14)	C7	C8	C9	120.94(19)
C1	N1	C3	112.98(15)	C10	C9	C8	117.20(19)
C1	N1	C10	126.12(15)	C5	C10	N1	109.05(16)
C10	N1	C3	110.20(15)	C9	C10	N1	128.14(18)
O1	C1	O2	122.66(17)	C9	C10	C5	122.81(17)
O1	C1	N1	128.02(18)	C12	C11	C2	119.67(18)
O2	C1	N1	109.25(16)	C16	C11	C2	120.92(17)
O2	C2	C3	103.71(14)	C16	C11	C12	119.39(19)
O2	C2	C11	108.29(15)	C13	C12	C11	119.6(2)
C11	C2	C3	118.23(15)	C14	C13	C12	120.7(2)
N1	C3	C2	100.15(14)	C13	C14	C15	120.0(2)
N1	C3	C4	103.49(14)	C16	C15	C14	119.7(2)
N1	C3	C17	111.61(15)	C15	C16	C11	120.7(2)
C4	C3	C2	116.53(15)	C18	C17	C3	122.19(17)
C17	C3	C2	114.88(14)	C18	C17	C22	118.89(17)
C17	C3	C4	109.21(15)	C22	C17	C3	118.80(17)
C5	C4	C3	102.38(15)	C17	C18	C19	120.58(19)
C6	C5	C4	130.73(18)	C20	C19	C18	120.05(19)
C10	C5	C4	109.98(15)	C19	C20	C21	120.18(17)
C10	C5	C6	119.13(17)	C22	C21	C20	119.59(18)
C5	C6	C7	119.20(19)	C21	C22	C17	120.70(19)
C8	C7	C6	120.61(19)				

Table S-14 Hydrogen Atom Coordinates (Å×10^4) and Isotropic Displacement Parameters (Å^2×10^3) for OIC312v_0m.

Atom	*x*	*y*	*z*	U(eq)
H2	6452.09	6870.12	1049.62	19
H4A	4587.46	8191.39	2620.77	19
H4B	4611.28	8082	1322.13	19
H6	1927.35	7732.38	1763.82	24
H7	404.7	5046.59	1227.93	27
H8	958.97	1619.25	776.8	25
H9	3081.23	717.11	981.89	21
H12	8013.13	9414.13	1720.38	29
H13	9962.38	10395.95	2866.22	41
H14	11104.61	7993.84	4176.18	42
H15	10350.9	4507.63	4293.86	36
H16	8403.37	3514.39	3159.66	26
H18	5566.1	2222.71	3681.69	20
H19	6139.99	2067.95	5638.07	24
H20	7016.57	5064.04	6677.05	25
H21	7347.59	8230.02	5766.29	24
H22	6732.78	8409.01	3815.67	22

7. References

1. Bruker (2017) Apex3 v2017.3-0. Bruker AXS Inc., Madison, Wisconsin, USA.
2. L. Krause, R. Herbst-Irmer, G. M. Sheldrick, D. Stalke, *J. Appl. Cryst.*, **2015**, *48*, 3.
3. G. M. Sheldrick, *Acta Cryst.*, **2015**, *A71*, 3.
4. G. M. Sheldrick, *Acta Cryst.*, **2015**, *C71*, 3.
5. O.V. Dolomanov, L. J. Bourhis, R. J. Gildea, J. A. K. Howard, H. Puschmann, *J. Appl. Cryst.*, **2009**, *42*, 339.
6. A. D. Becke, *J. Chem. Phys.* **1993**, *98*, 5648.
7. M. J. Frisch, G. W. Trucks, H. B. Schlegel, G. E. Scuseria, M. A. Robb, J. R. Cheeseman, G. Scalmani, V. Barone, B. Mennucci, G. A. Petersson, H. Nakatsuji, M. Caricato, X. Li, H. P. Hratchian, A. F. Izmaylov, J. Bloino, G. Zheng, J. L. Sonnenberg, M. Hada, M. Ehara, K. Toyota, R. Fukuda, J. Hasegawa, M. Ishida, T. Nakajima, Y. Honda, O. Kitao, H. Nakai, T. Vreven, J. A. Montgomery Jr, J. E. Peralta, F. Ogliaro, M. Bearpark, J. J. Heyd, E. Brothers, K. N. Kudin, V. N. Staroverov, R. Kobayashi, J. Normand, K. Raghavachari, A. Rendell, J. C. Burant, S. S. Iyengar, J. Tomasi, M. Cossi, N. Rega, J. M. Millam, M. Klene, J. E. Knox, J. B. Cross, V. Bakken, C. Adamo, J. Jaramillo, R. Gomperts, R. E. Stratmann, O. Yazyev, A. J. Austin, R. Cammi, C. Pomelli, J. W. Ochterski, R. L. Martin, K. Morokuma, V. G. Zakrzewski, G. A. Voth, P. Salvador, J. J. Dannenberg, S. Dapprich, A. D. Daniels, Ö. Farkas, J. B. Foresman, J. V. Ortiz, J. Cioslowski, D. J. Fox, *Gaussian 09, Revision D.01*, Gaussian, Inc., Wallingford CT, 2009.
8. S. Grimme, S. Ehrlich, L. Goerigk, *J. Comput. Chem.* **2011**, *32*, 1456.
9. *a*) A. D. McLean, G. S. Chandler, *J. Chem. Phys.* **1980**, *72*, 5639; *b*) K. Raghavachari, J. S. Binkley, R. Seeger, J. A. Pople, *J. Chem. Phys.* **1980**, *72*, 650; *c*) F. Weigend, R. Ahlrichs, *Phys. Chem. Chem. Phys.*, **2005**, 7, 3297.
10. *a*) G. Scalmani, M. J. Frisch, *J. Chem. Phys.* **2010**, *132*, 114110; *b*) M. Cossi, N. Rega, G. Scalmani, V. Barone, *J. Comput. Chem.* **2003**, *24*, 669 and references therein.
11. J. Slätt, J. Bergman, *Tetrahedron* **2002**, *58*, 9187.
12. R. Kuwano, M. Kashiwabara, *Org. Lett.* **2006**, *8*, 2653.
13. S. Coulton, T. L. Gilchrist, K. Graham, *Tetrahedron* **1997**, *53*, 791.
14. H. Fuwa, M. Sasaki, *Org. Lett.* **2007**, *9*, 3347.
15. K. E. Eckert, A. J. Lepore, B. L. Ashfeld, *Helv. Chim. Acta* **2019**, *102*, e1900192.
16. X. Wen, Y. Wang, X. P. Zhang, *Chem. Sci.* **2018**, *9*, 5082.
17. Y. Ge, Z. Wang, Z. Han, K. Ding, *Chem. Eur. J.* **2020**, *26*, 15482.
18. R. Kuwano, M. Kashiwabara, *Org. Lett.* **2006**, *8*, 2653.
19. V. Gotor-Fernández, P. Fernández-Torres, V. Gotor, *Tetrahedron: Asym.* **2006**, *17*, 2558.

8. HPLC traces

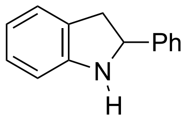

(±)-**1a**

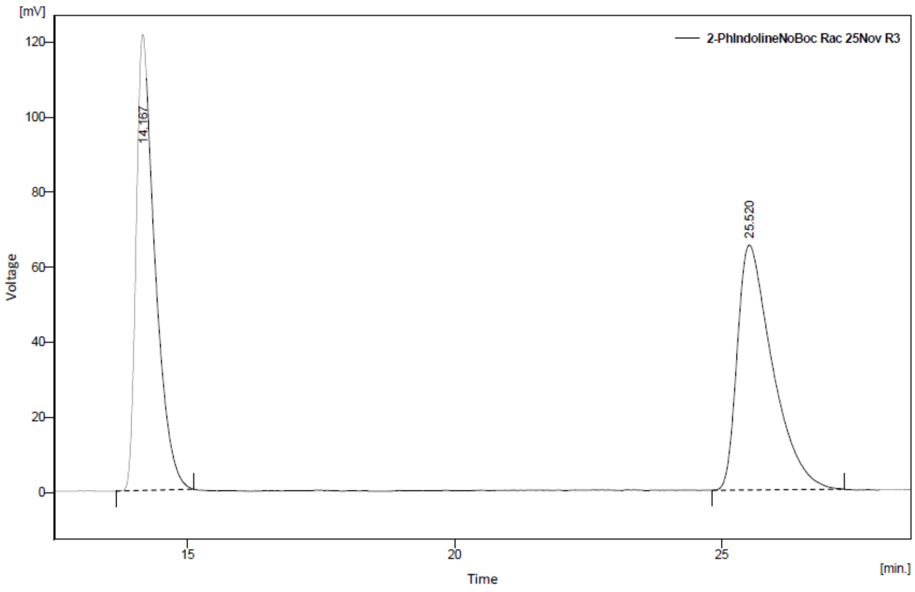

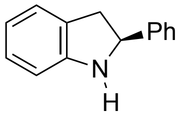

(*S*)-**1a**

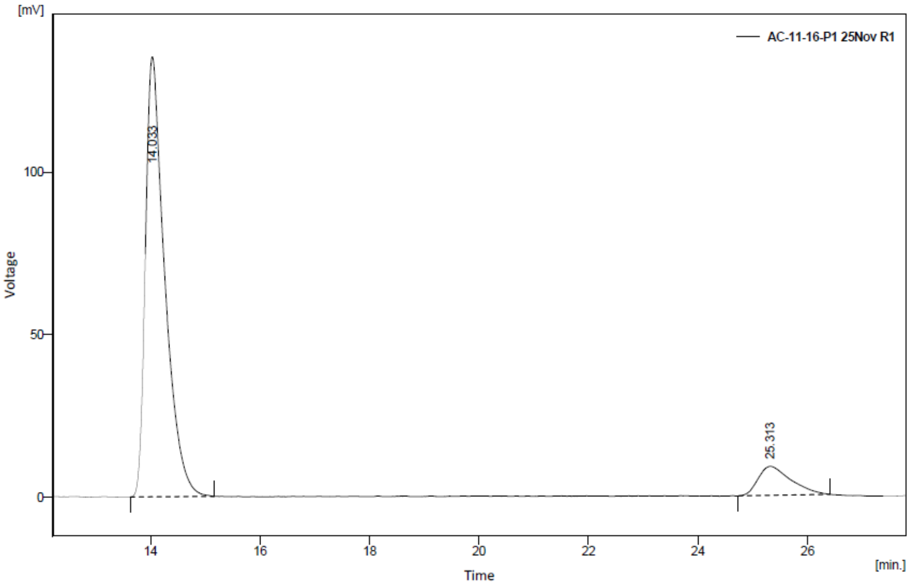

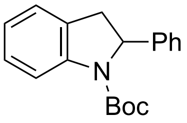
Ph
N
Boc

(±)-**2a**

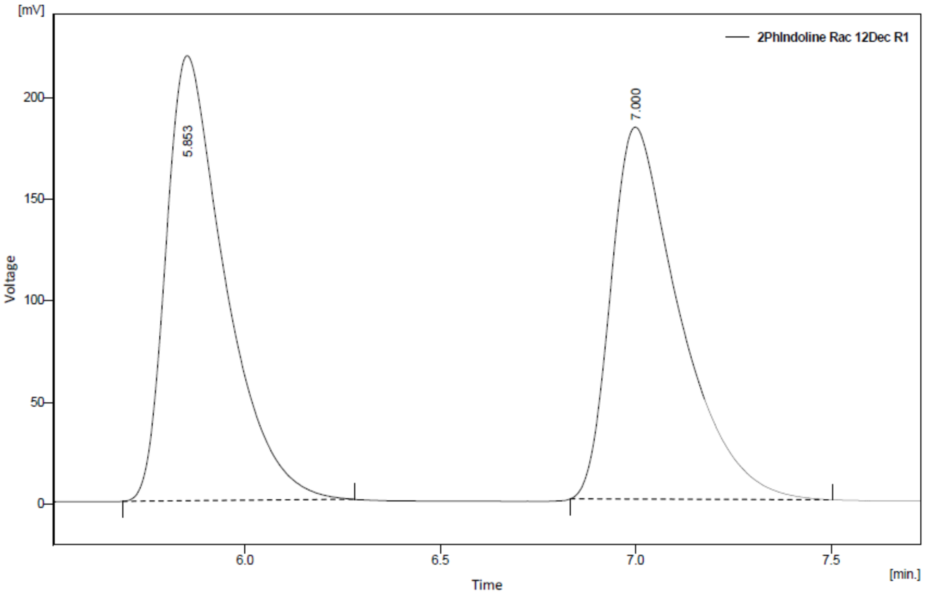
[mV]
2PhIndoline Rac 12Dec R1
200
150
100
50
0
Voltage
5.853
7.000
6.0
6.5
7.0
7.5
Time
[min.]

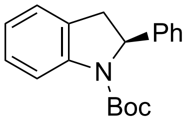
Ph
N
Boc

(*S*)-**2a**

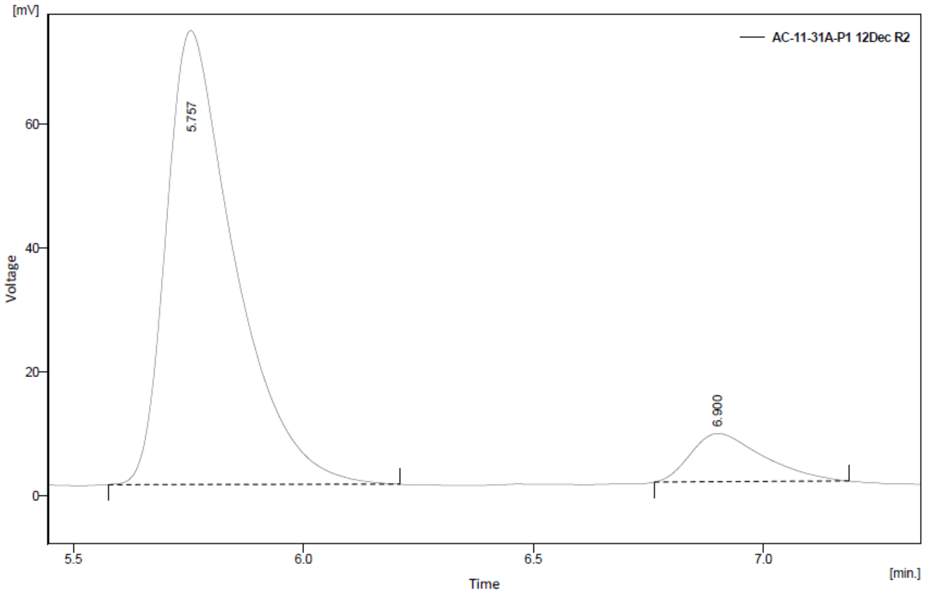
[mV]
AC-11-31A-P1 12Dec R2
60
40
20
0
Voltage
5.757
6.900
5.5
6.0
6.5
7.0
Time
[min.]

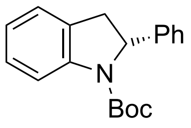

(*R*)-**2a**

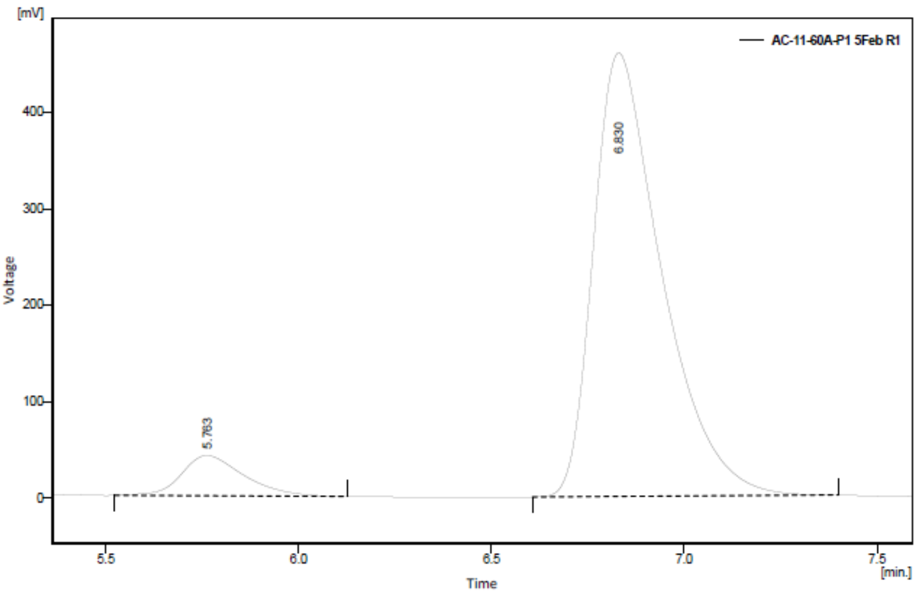
[mV]
AC-11-60A-P1 5Feb R1
400
300
200
100
0
Voltage
6.830
5.763
5.5
6.0
6.5
7.0
7.5
[min.]
Time

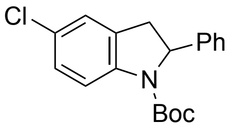
Cl
Ph
N
Boc

(±)-**2b**

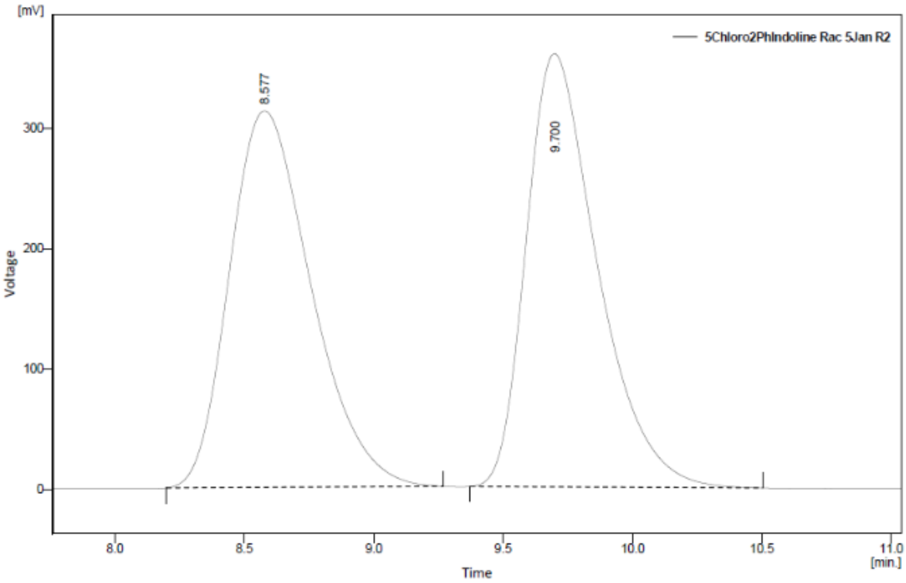
[mV]
5Chloro2PhIndoline Rac 5Jan R2
8.577
9.700
300
200
100
0
Voltage
8.0
8.5
9.0
9.5
10.0
10.5
11.0
[min.]
Time

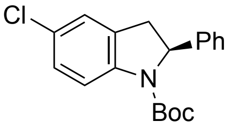
Cl
Ph
N
Boc

(*S*)-**2b**

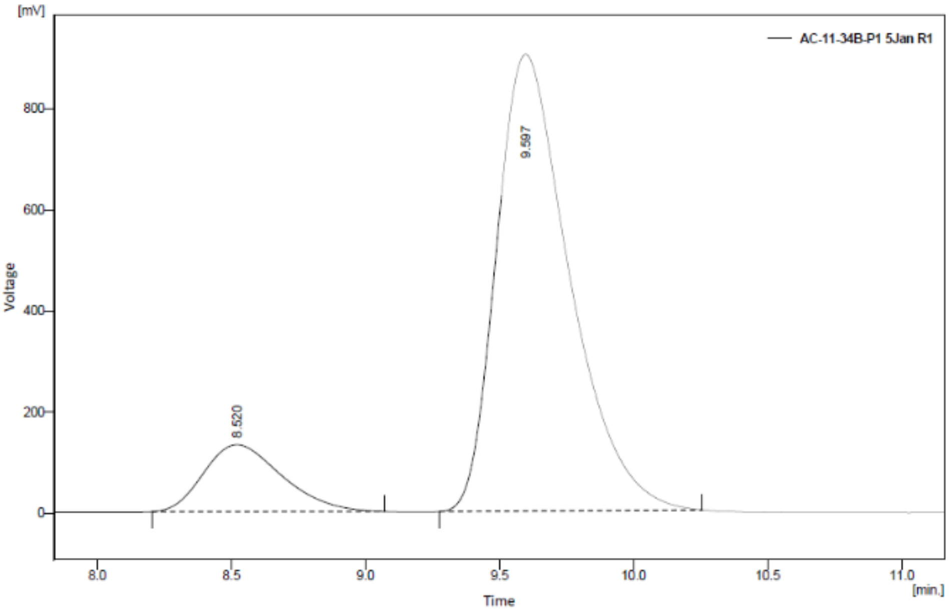
[mV]
AC-11-34B-P1 5Jan R1
9.597
8.520
800
600
400
200
0
Voltage
8.0
8.5
9.0
9.5
10.0
10.5
11.0
[min.]
Time

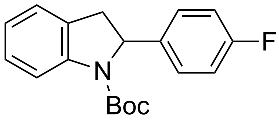

F
N
Boc

(±)-**2c**

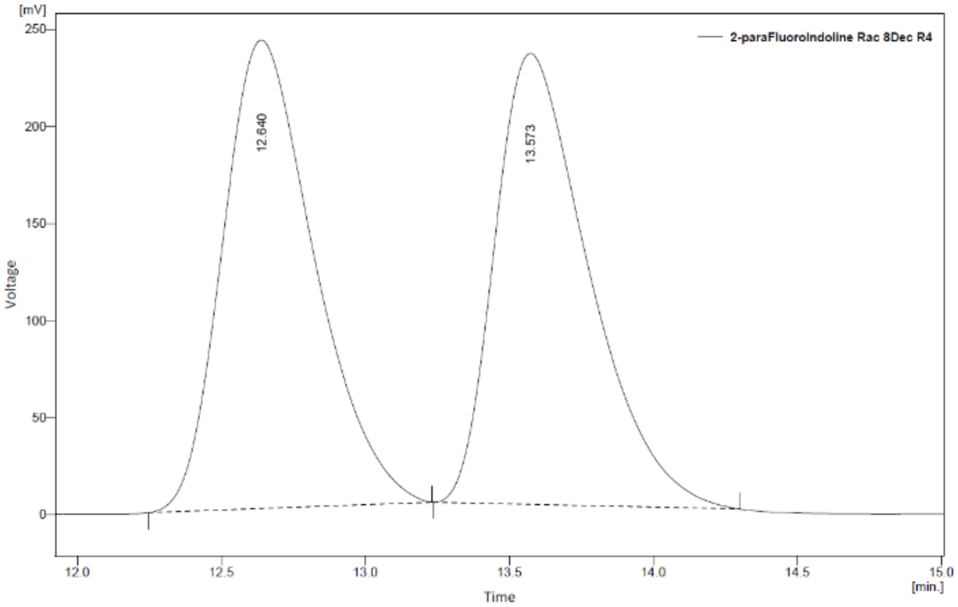

[mV]
250
200
150
100
50
0
Voltage
12.640
13.573
2-paraFluoroIndoline Rac 8Dec R4
12.0
12.5
13.0
13.5
14.0
14.5
15.0
[min.]
Time

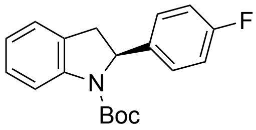

F
N
Boc

(*S*)-**2c**

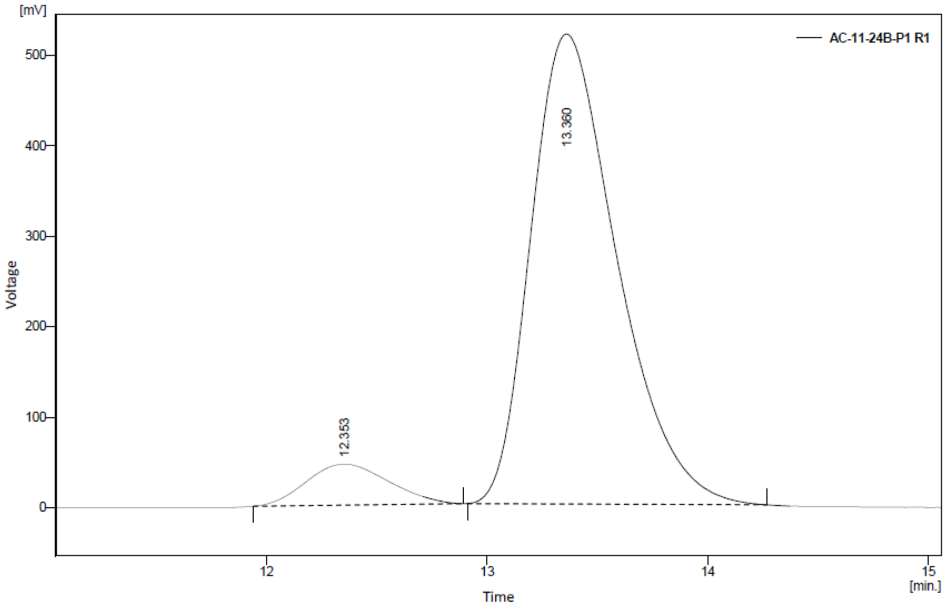

[mV]
500
400
300
200
100
0
Voltage
12.353
13.360
AC-11-24B-P1 R1
12
13
14
15
[min.]
Time

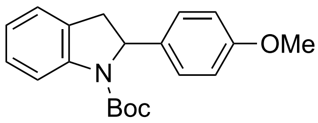
OMe
N
Boc

(±)-**2d**

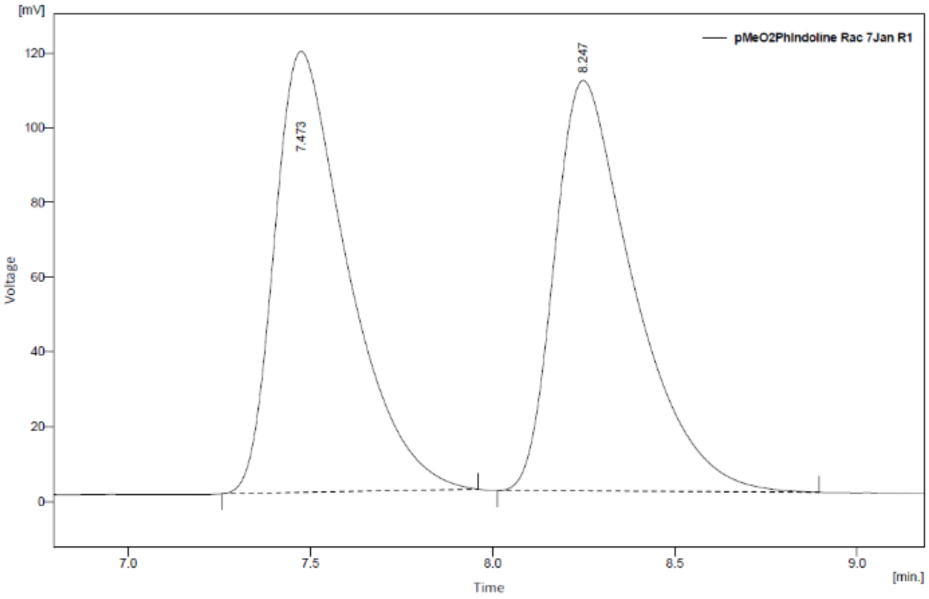
[mV]
pMeO2PhIndoline Rac 7Jan R1
7.473
8.247
Voltage
Time
[min.]

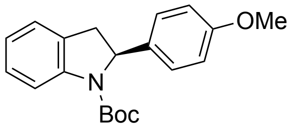
OMe
N
Boc

(*S*)-**2d**

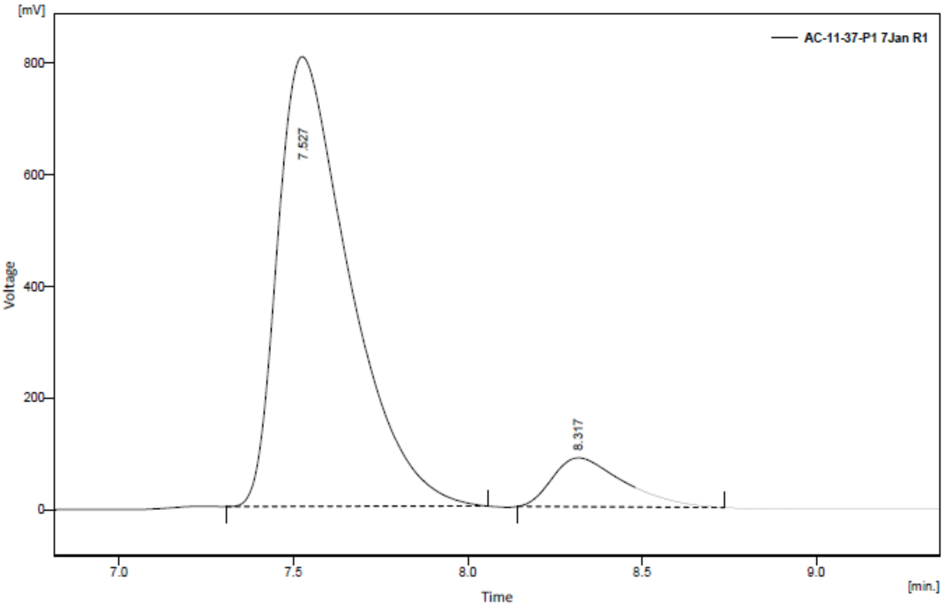
[mV]
AC-11-37-P1 7Jan R1
7.527
8.317
Voltage
Time
[min.]

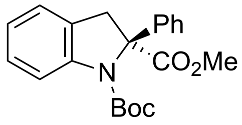

(S)-**3a**

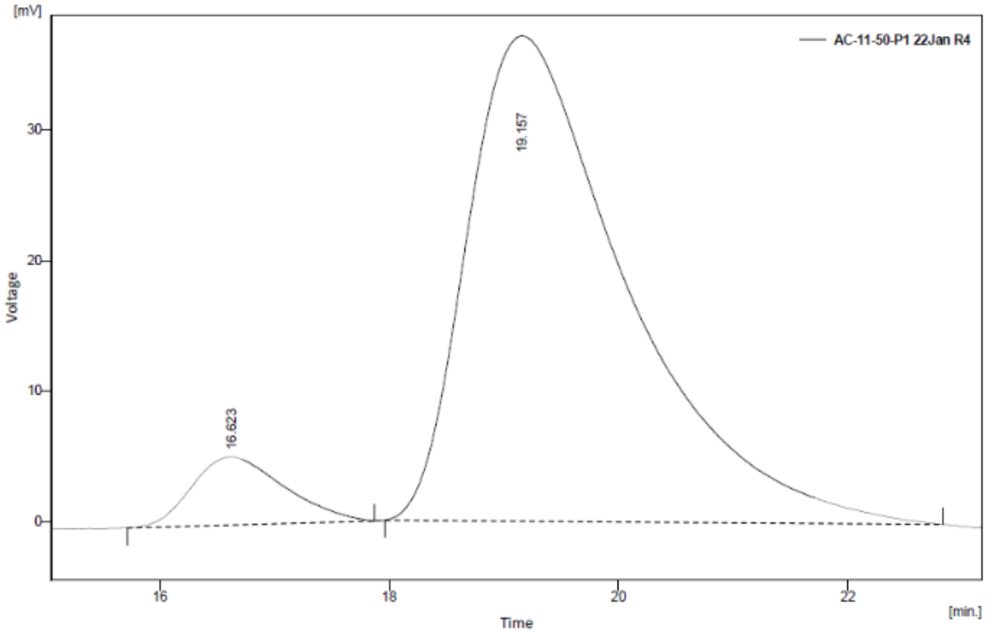

[mV]
AC-11-50-P1 22Jan R4
19.157
16.623
Voltage
Time
[min.]

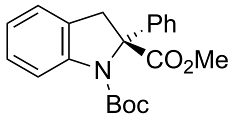

(R)-**3a**

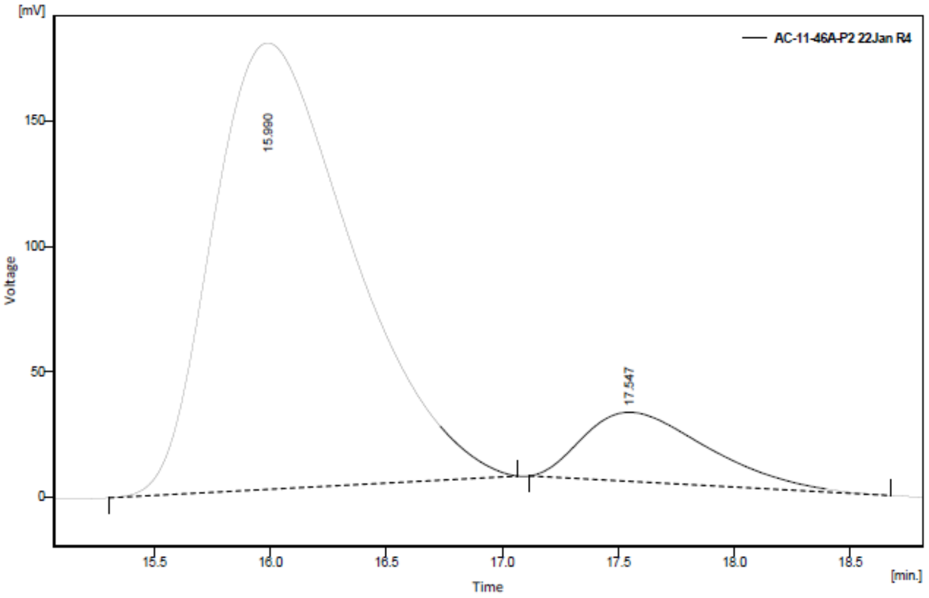

[mV]
AC-11-46A-P2 22Jan R4
15.990
17.547
Voltage
Time
[min.]

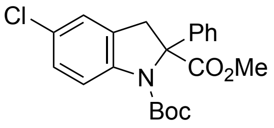
Cl
Ph
CO2Me
N
Boc

(±)-**3b**

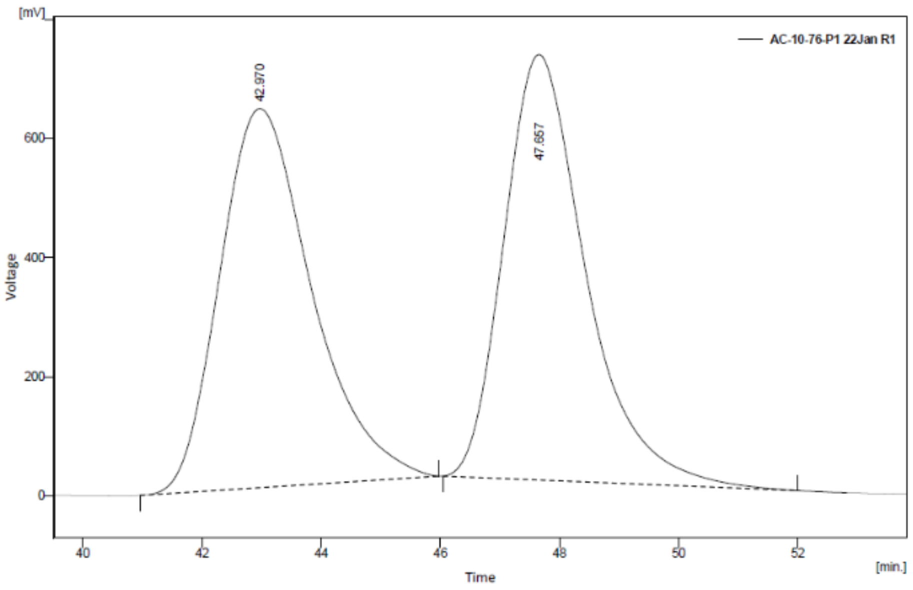
[mV]
AC-10-76-P1 22Jan R1
42.970
47.667
600
400
200
0
Voltage
40
42
44
46
48
50
52
[min.]
Time

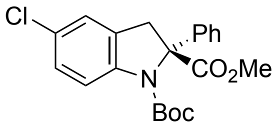
Cl
Ph
CO2Me
N
Boc

(*R*)-**3b**

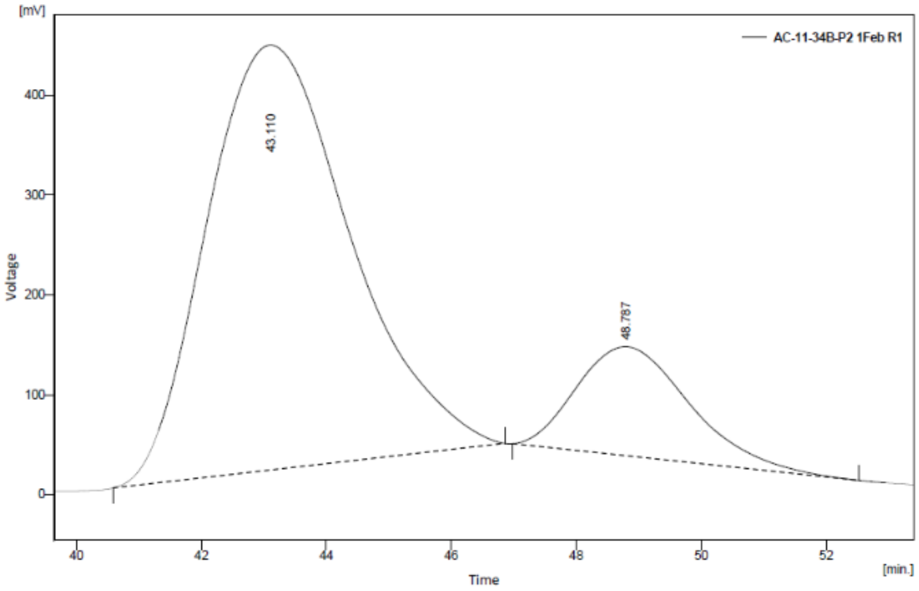
[mV]
AC-11-34B-P2 1Feb R1
43.110
48.787
400
300
200
100
0
Voltage
40
42
44
46
48
50
52
[min.]
Time

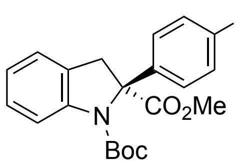

F
CO2Me
N
Boc

(*S*)-**3c**

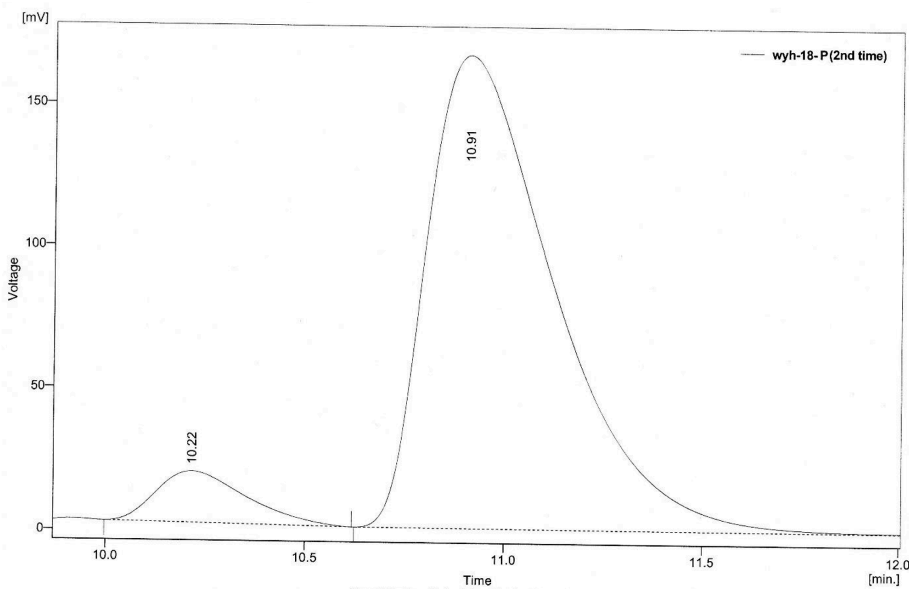

[mV]
wyh-18-P(2nd time)
150
100
50
0
Voltage
10.91
10.22
10.0
10.5
11.0
11.5
12.0
Time
[min.]

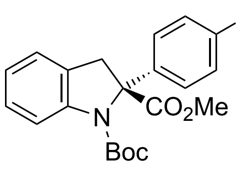

F
CO2Me
N
Boc

(*R*)-**3c**

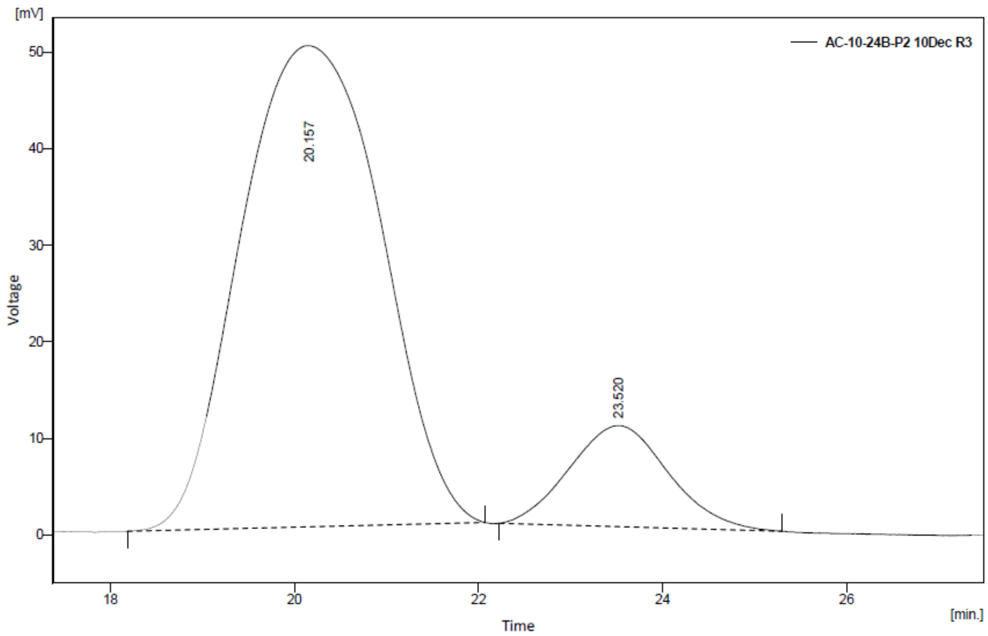

[mV]
AC-10-24B-P2 10Dec R3
50
40
30
20
10
0
Voltage
20.157
23.520
18
20
22
24
26
Time
[min.]

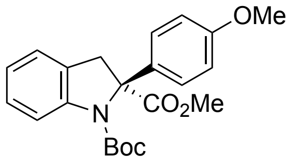
OMe
CO_2Me
N
Boc

(*S*)-**3d**

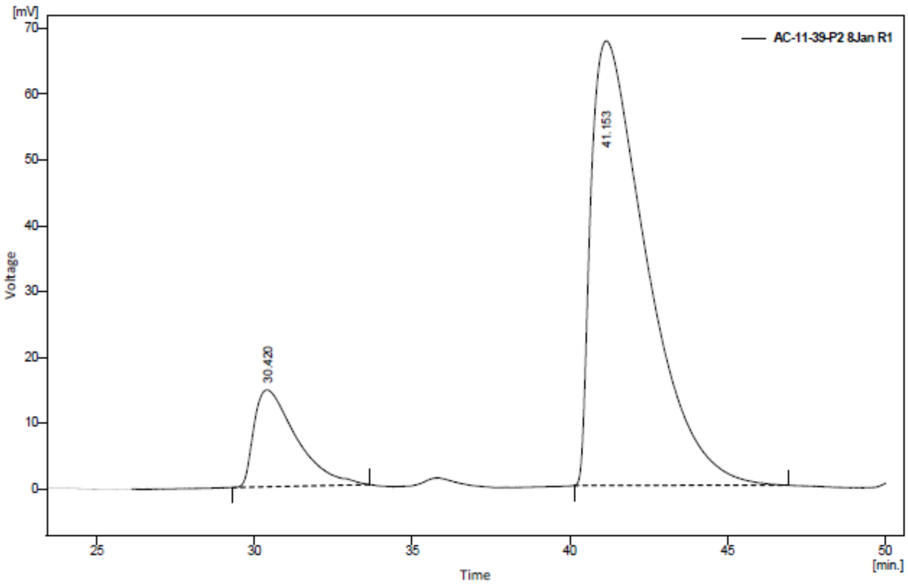
[mV]
AC-11-39-P2 8Jan R1
41.153
30.420
Voltage
Time
[min.]
70
60
50
40
30
20
10
0
25
30
35
40
45
50

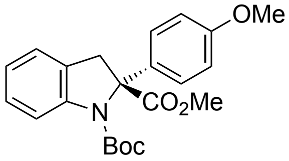
OMe
CO_2Me
N
Boc

(*R*)-**3d**

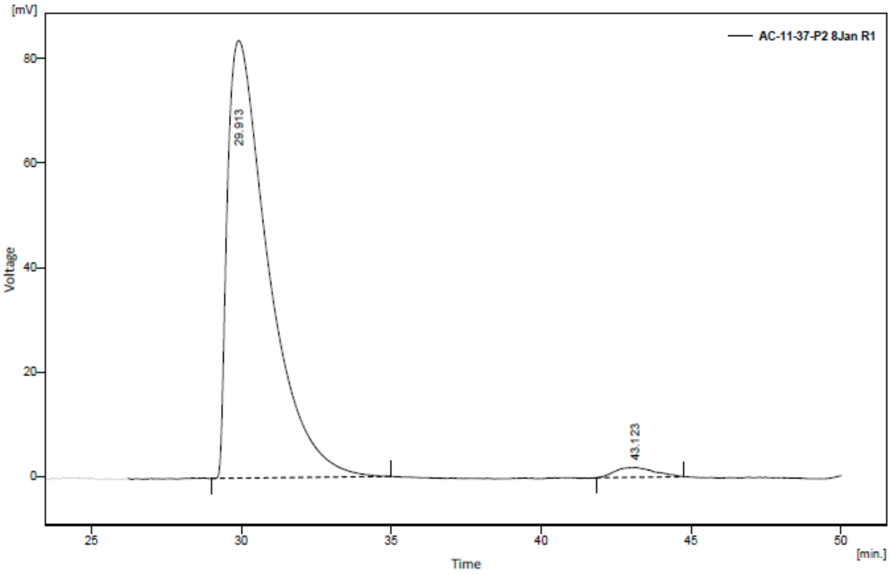
[mV]
AC-11-37-P2 8Jan R1
29.913
43.123
Voltage
Time
[min.]
80
60
40
20
0
25
30
35
40
45
50

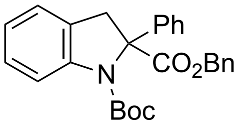

(±)-**7a**

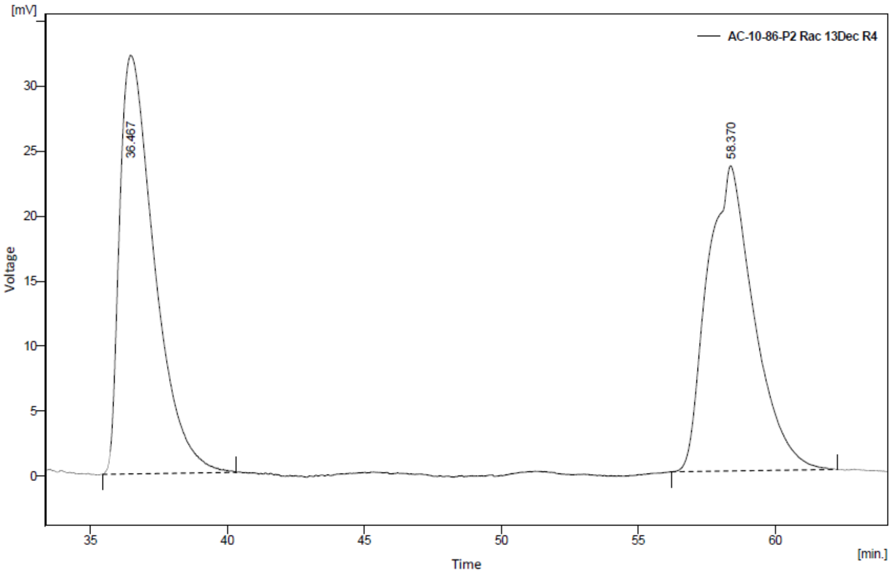

[mV]
AC-10-86-P2 Rac 13Dec R4
36.467
58.370
Voltage
Time
[min.]

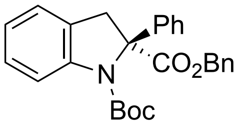

(*S*)-**7a**

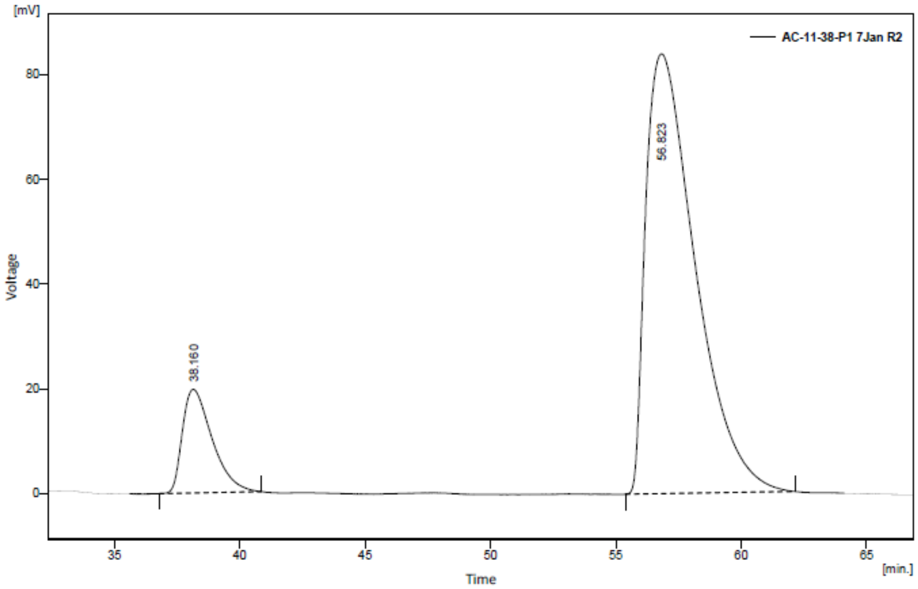

[mV]
AC-11-38-P1 7Jan R2
38.160
56.823
Voltage
Time
[min.]

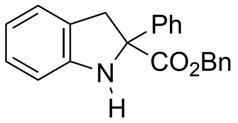
Ph
CO2Bn
N
H

(±)-**10a**

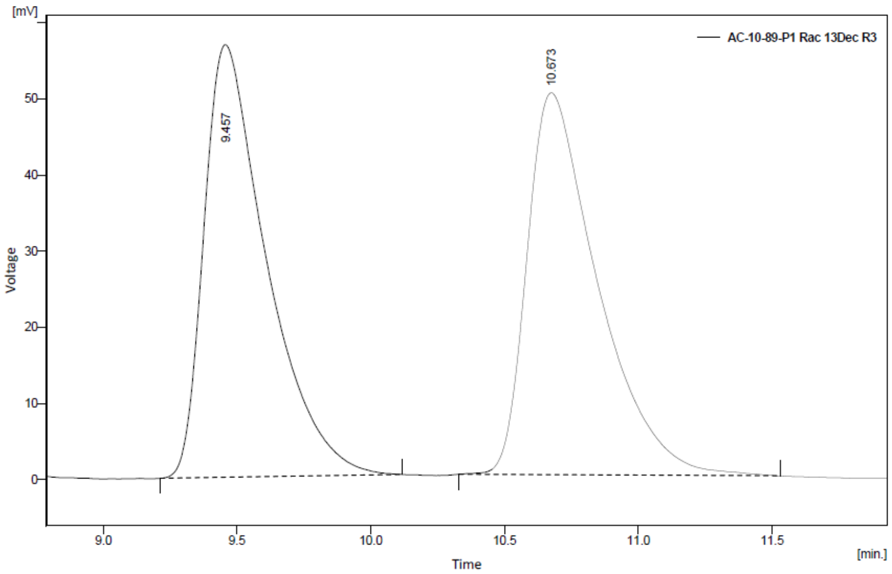
[mV]
AC-10-89-P1 Rac 13Dec R3
9.457
10.673
Voltage
0
10
20
30
40
50
9.0
9.5
10.0
10.5
11.0
11.5
[min.]
Time

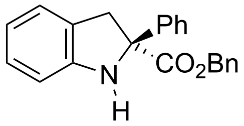
Ph
CO2Bn
N
H

(*S*)-**10a**

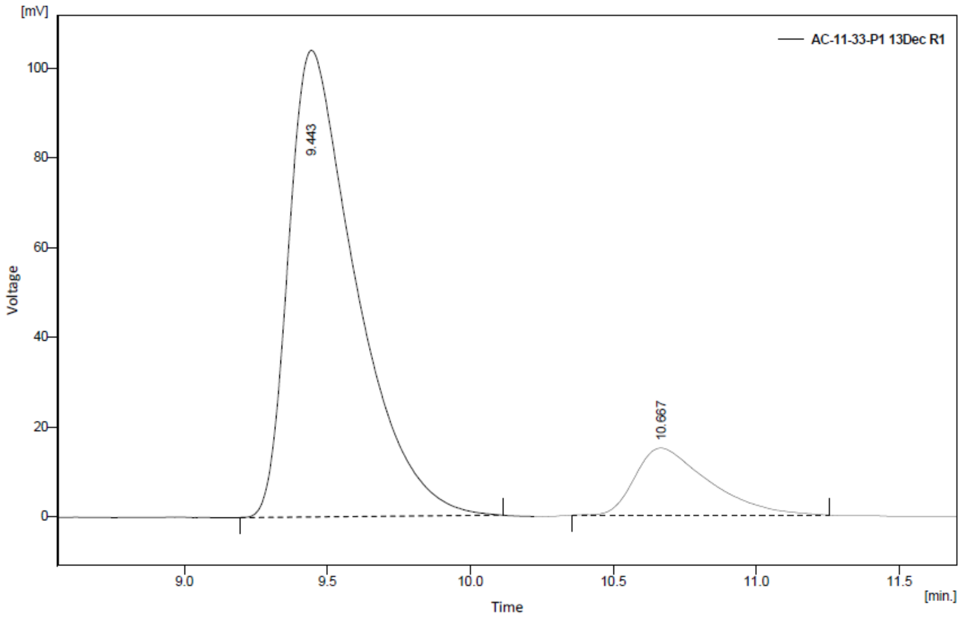
[mV]
AC-11-33-P1 13Dec R1
9.443
10.667
Voltage
0
20
40
60
80
100
9.0
9.5
10.0
10.5
11.0
11.5
[min.]
Time

9. NMR spectra (room temperature in $CDCl_3$ unless stated)

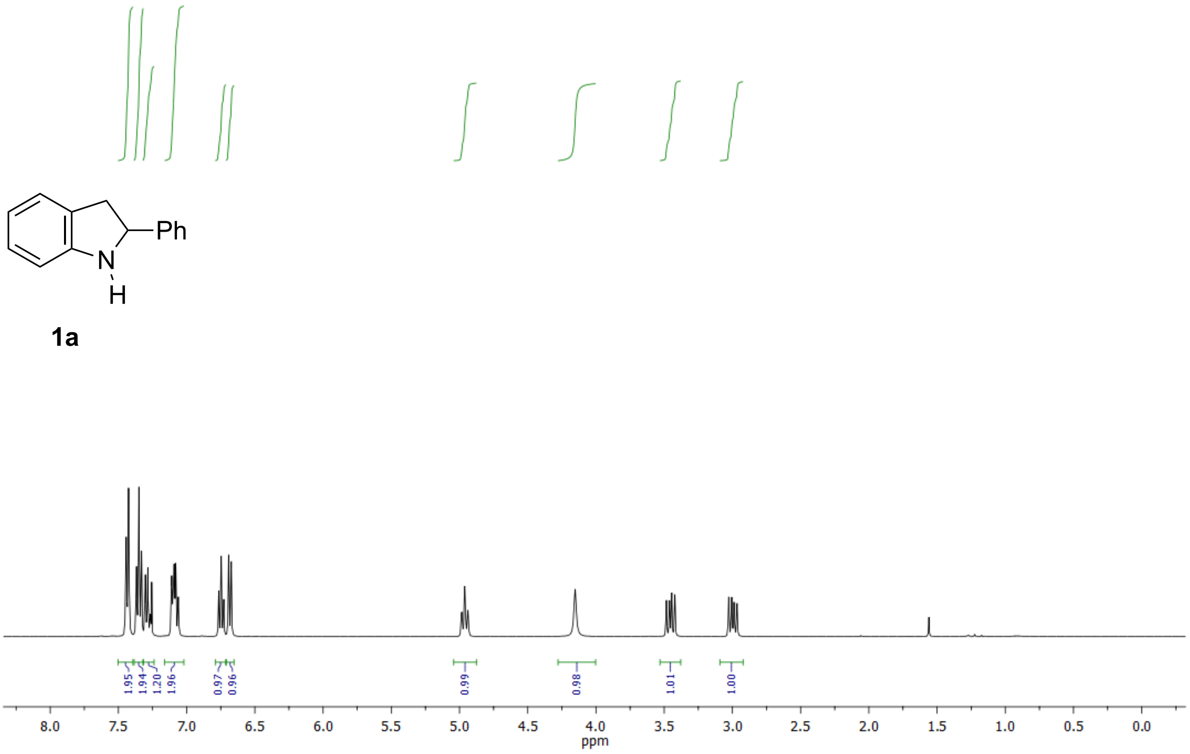

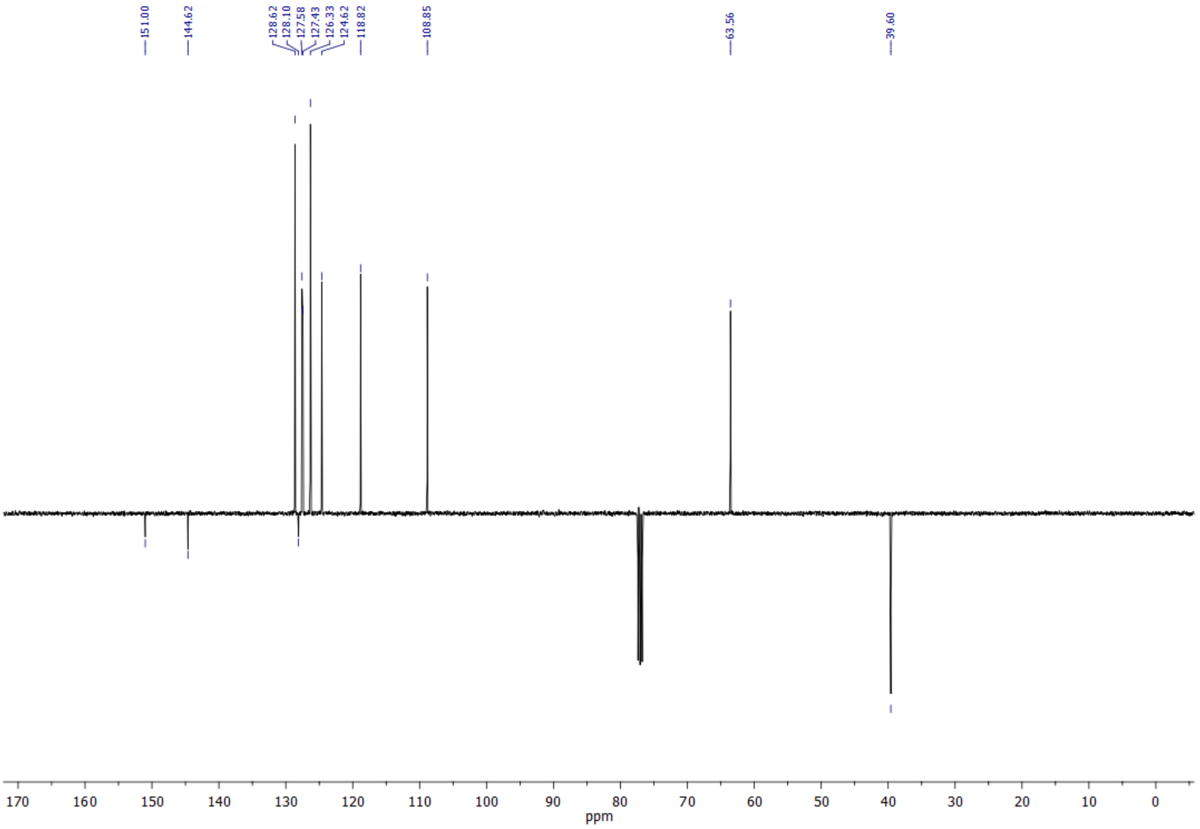

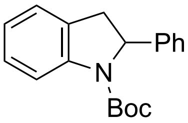
Ph
N
Boc

2a

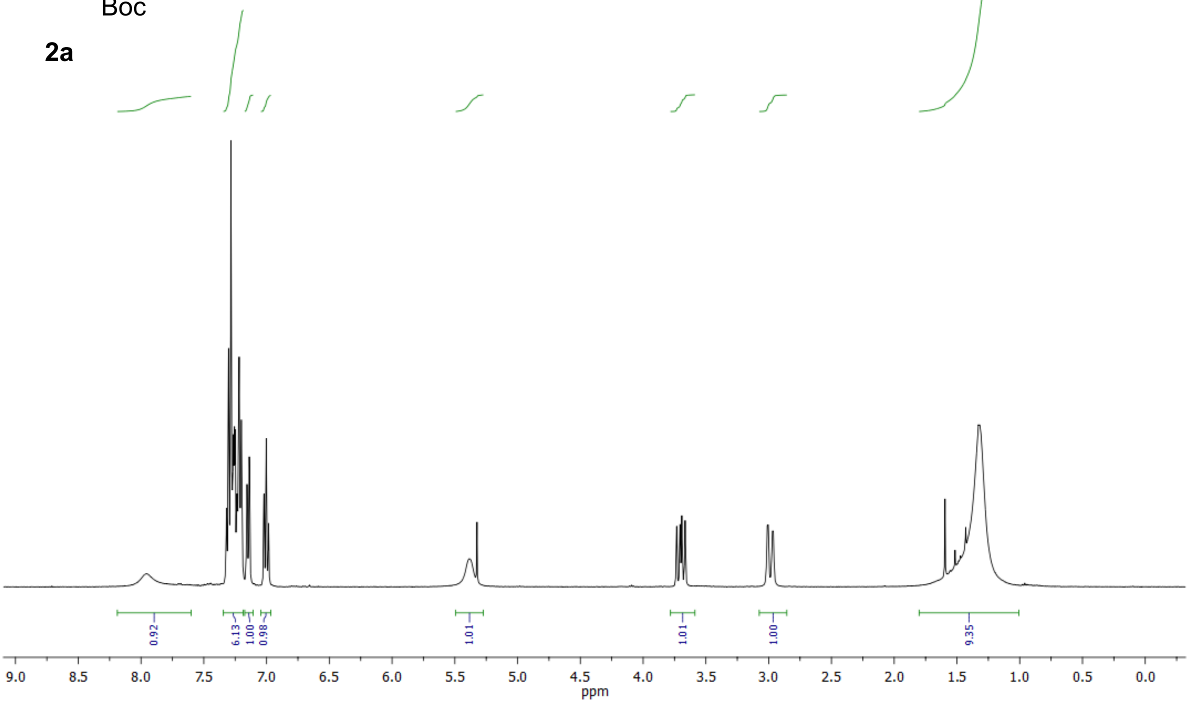
0.92
6.13
1.00
0.98
1.01
1.01
1.00
9.35
9.0
8.5
8.0
7.5
7.0
6.5
6.0
5.5
5.0
4.5
4.0
3.5
3.0
2.5
2.0
1.5
1.0
0.5
0.0
ppm

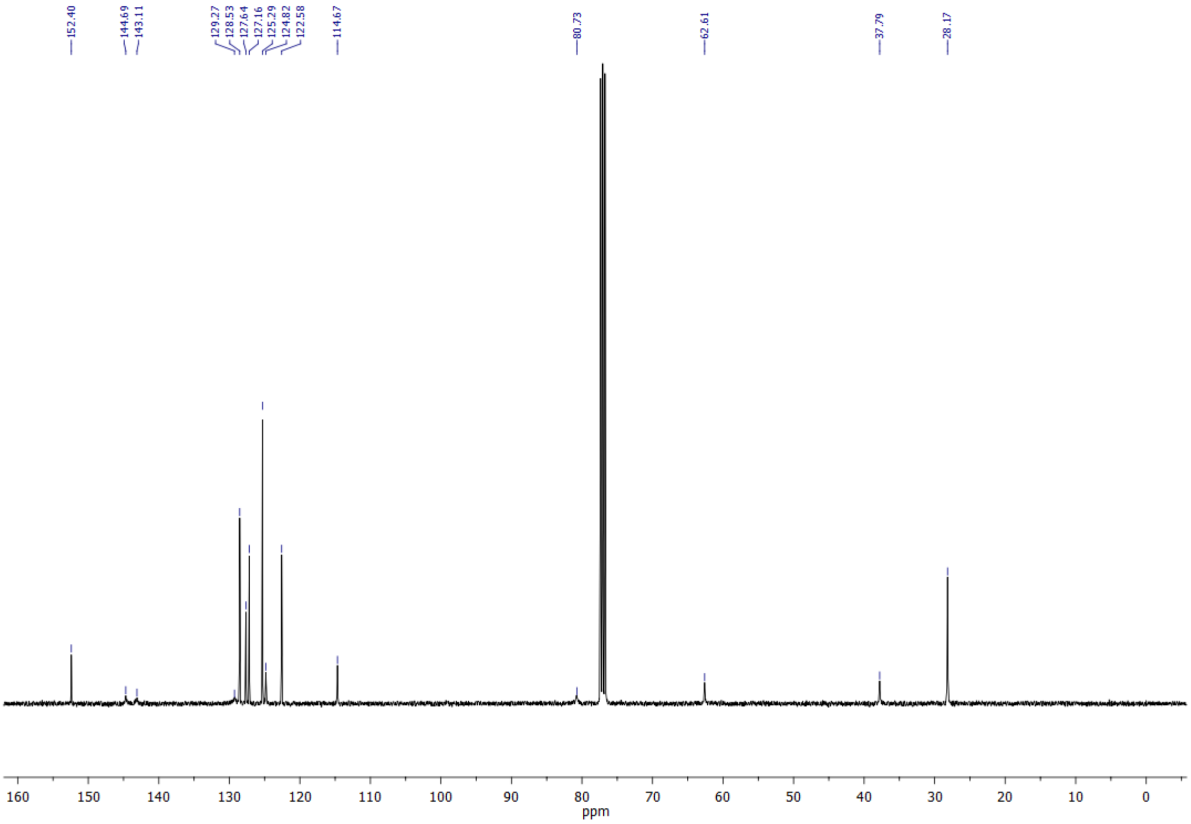
152.40
144.69
143.11
129.27
128.53
127.64
127.16
125.29
124.82
122.58
114.67
80.73
62.61
37.79
28.17
160
150
140
130
120
110
100
90
80
70
60
50
40
30
20
10
0
ppm

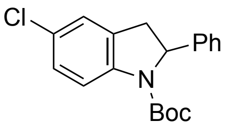
Cl
Ph
N
Boc

2b

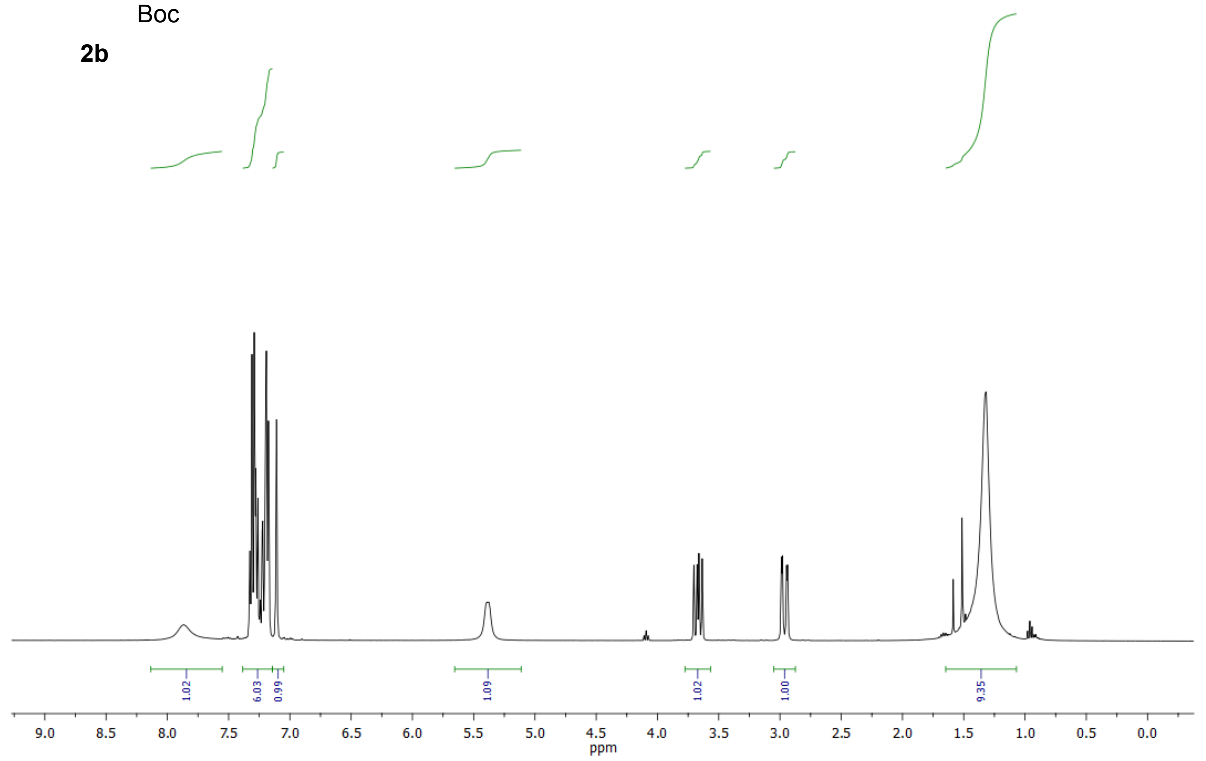
1.02
6.03
0.99
1.09
1.02
1.00
9.35
9.0 8.5 8.0 7.5 7.0 6.5 6.0 5.5 5.0 4.5 4.0 3.5 3.0 2.5 2.0 1.5 1.0 0.5 0.0
ppm

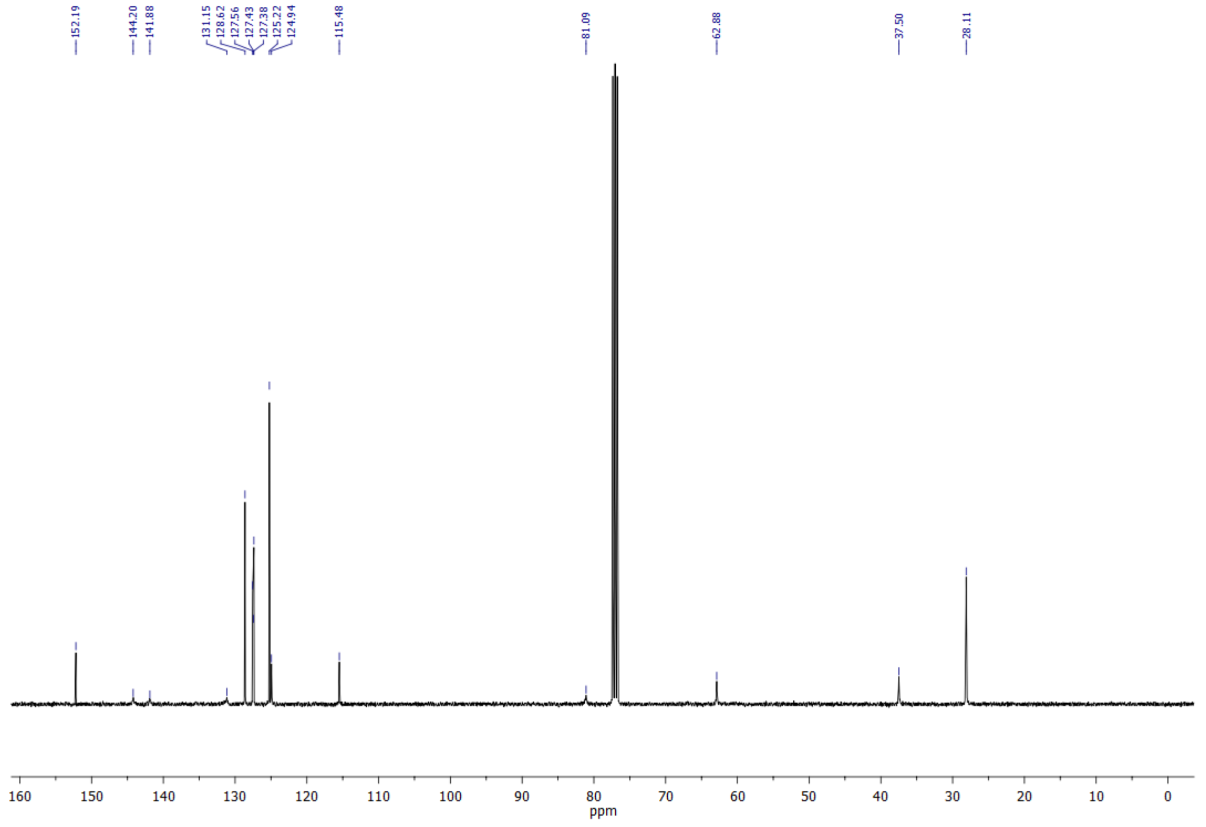
152.19
144.20
141.88
131.15
128.62
127.56
127.43
127.38
125.22
124.94
115.48
81.09
62.88
37.50
28.11
160 150 140 130 120 110 100 90 80 70 60 50 40 30 20 10 0
ppm

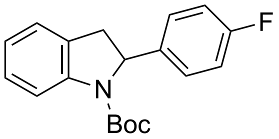
F
N
Boc

2c

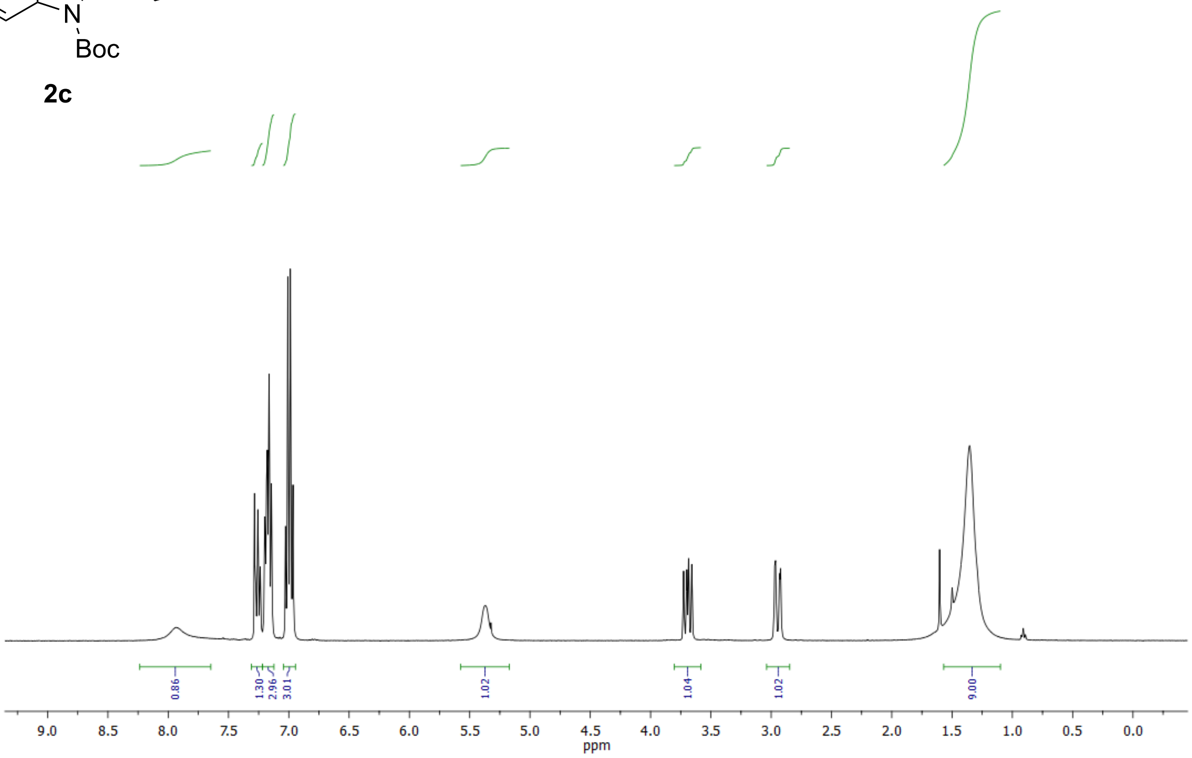
0.86
1.30
2.96
3.01
1.02
1.04
1.02
9.00
9.0 8.5 8.0 7.5 7.0 6.5 6.0 5.5 5.0 4.5 4.0 3.5 3.0 2.5 2.0 1.5 1.0 0.5 0.0
ppm

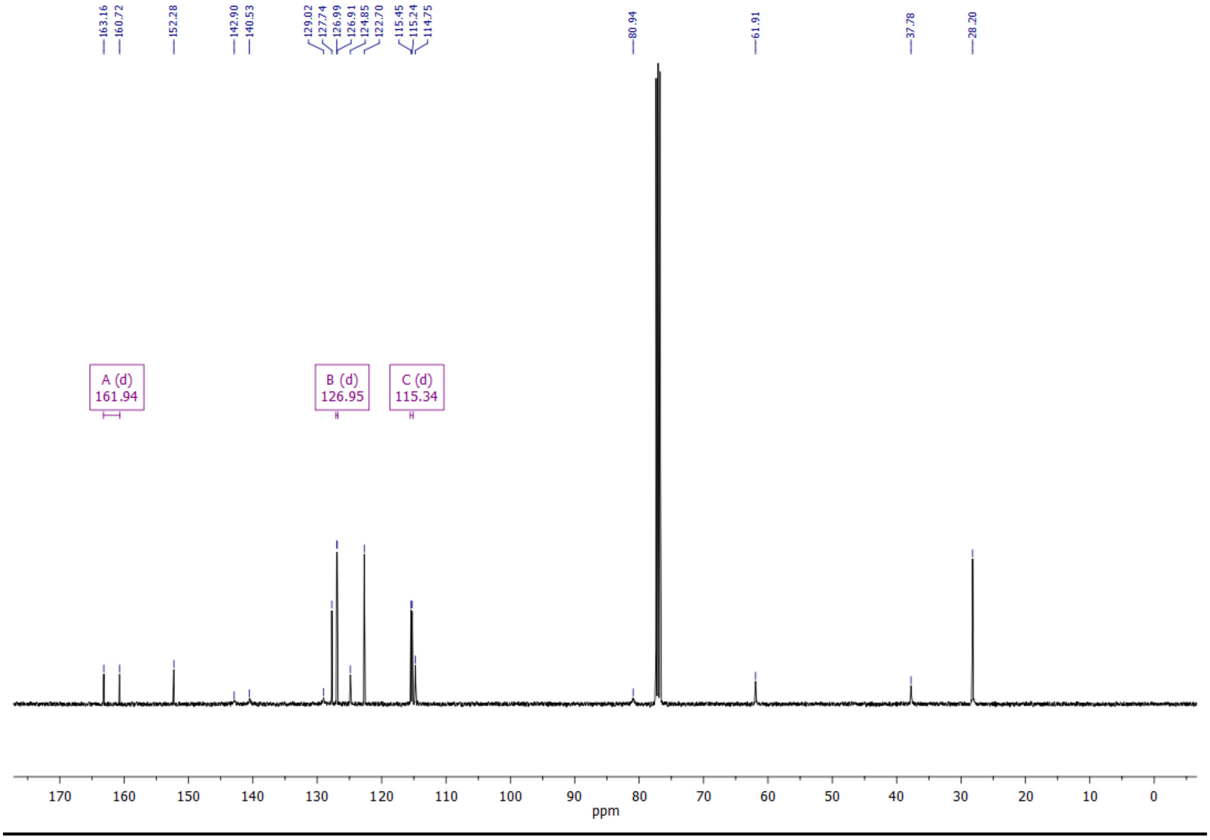
163.16
160.72
152.28
142.90
140.53
129.02
127.74
126.99
126.91
124.85
122.70
115.45
115.24
114.75
80.94
61.91
37.78
28.20
A (d)
161.94
B (d)
126.95
C (d)
115.34
170 160 150 140 130 120 110 100 90 80 70 60 50 40 30 20 10 0
ppm

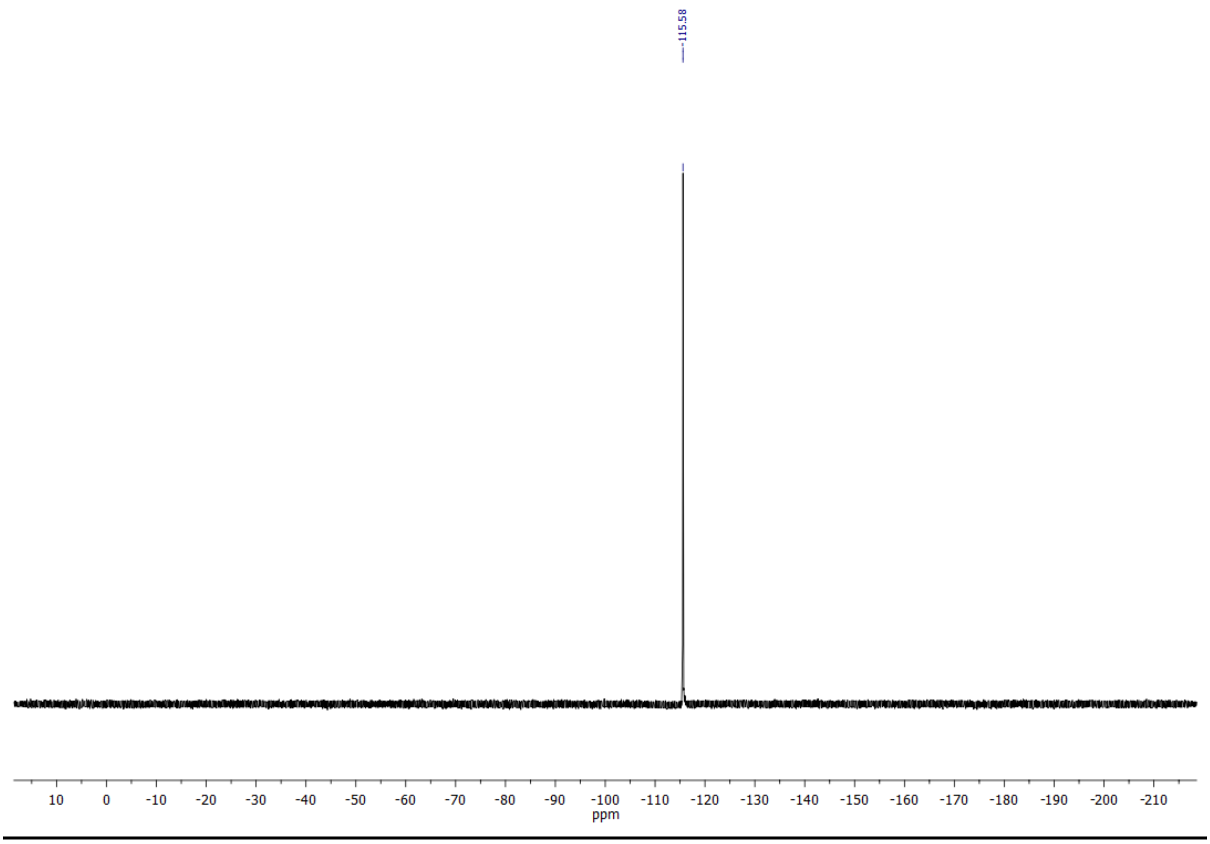
-115.58
10 0 -10 -20 -30 -40 -50 -60 -70 -80 -90 -100 -110 -120 -130 -140 -150 -160 -170 -180 -190 -200 -210
ppm

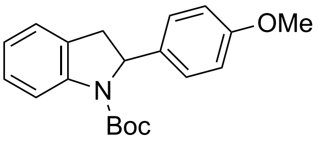
OMe
N
Boc

2d

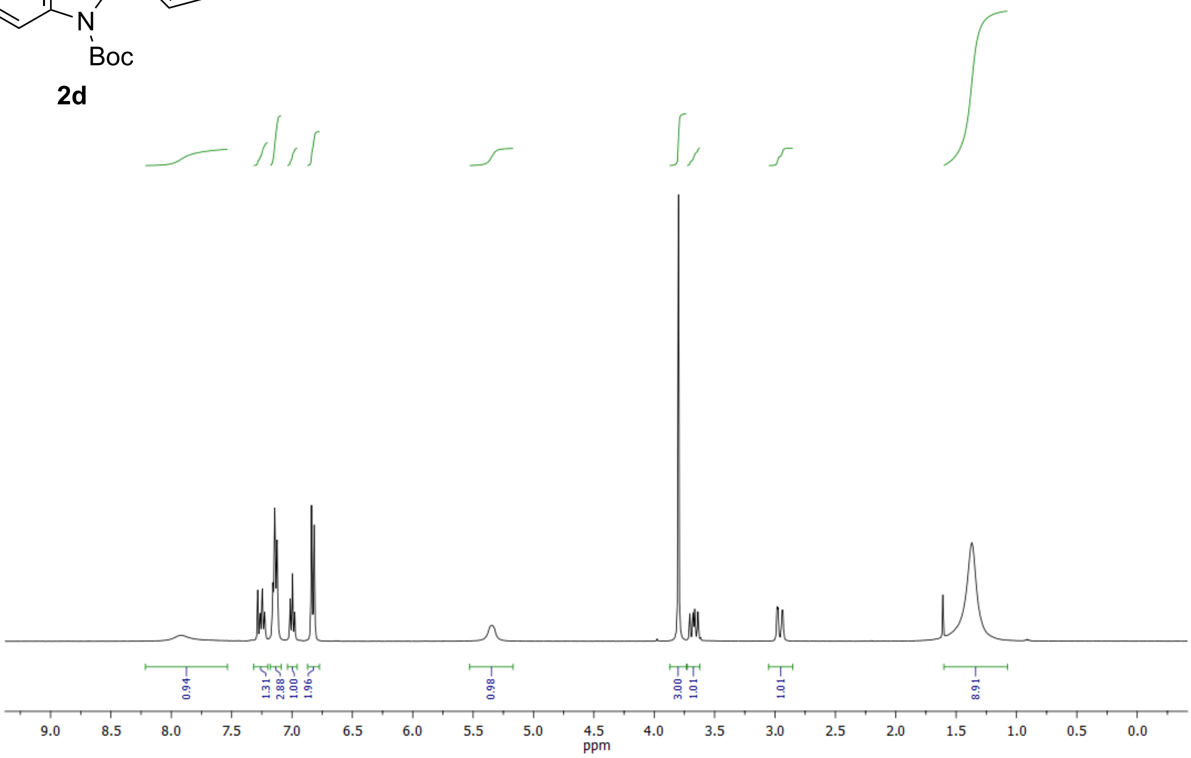
0.94
1.31
2.88
1.00
1.96
0.98
3.00
1.01
1.01
8.91
9.0 8.5 8.0 7.5 7.0 6.5 6.0 5.5 5.0 4.5 4.0 3.5 3.0 2.5 2.0 1.5 1.0 0.5 0.0
ppm

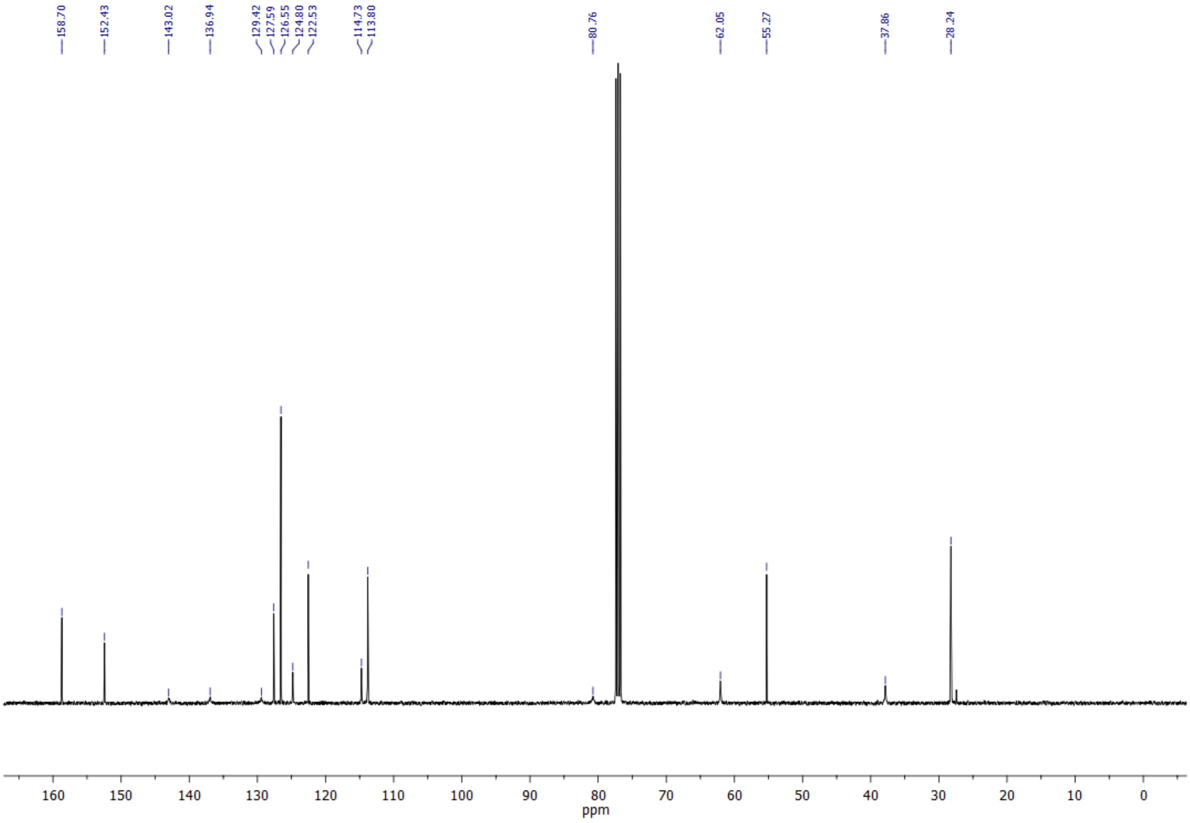
158.70
152.43
143.02
136.94
129.42
127.59
126.55
124.80
122.53
114.73
113.80
80.76
62.05
55.27
37.86
28.24
160 150 140 130 120 110 100 90 80 70 60 50 40 30 20 10 0
ppm

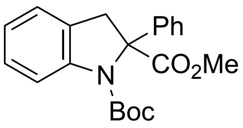
Ph
CO2Me
N
Boc

3a

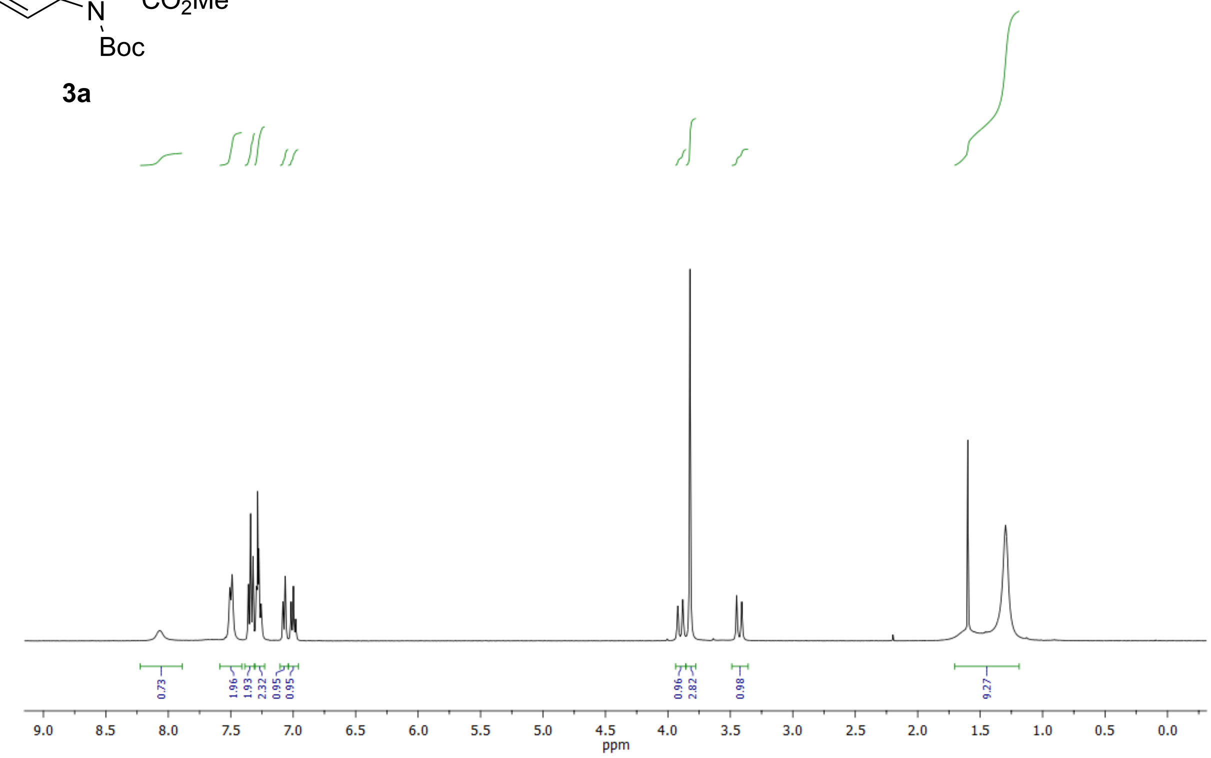
0.73
1.96
1.93
2.32
0.95
0.95
0.96
2.82
0.98
9.27
9.0 8.5 8.0 7.5 7.0 6.5 6.0 5.5 5.0 4.5 4.0 3.5 3.0 2.5 2.0 1.5 1.0 0.5 0.0
ppm

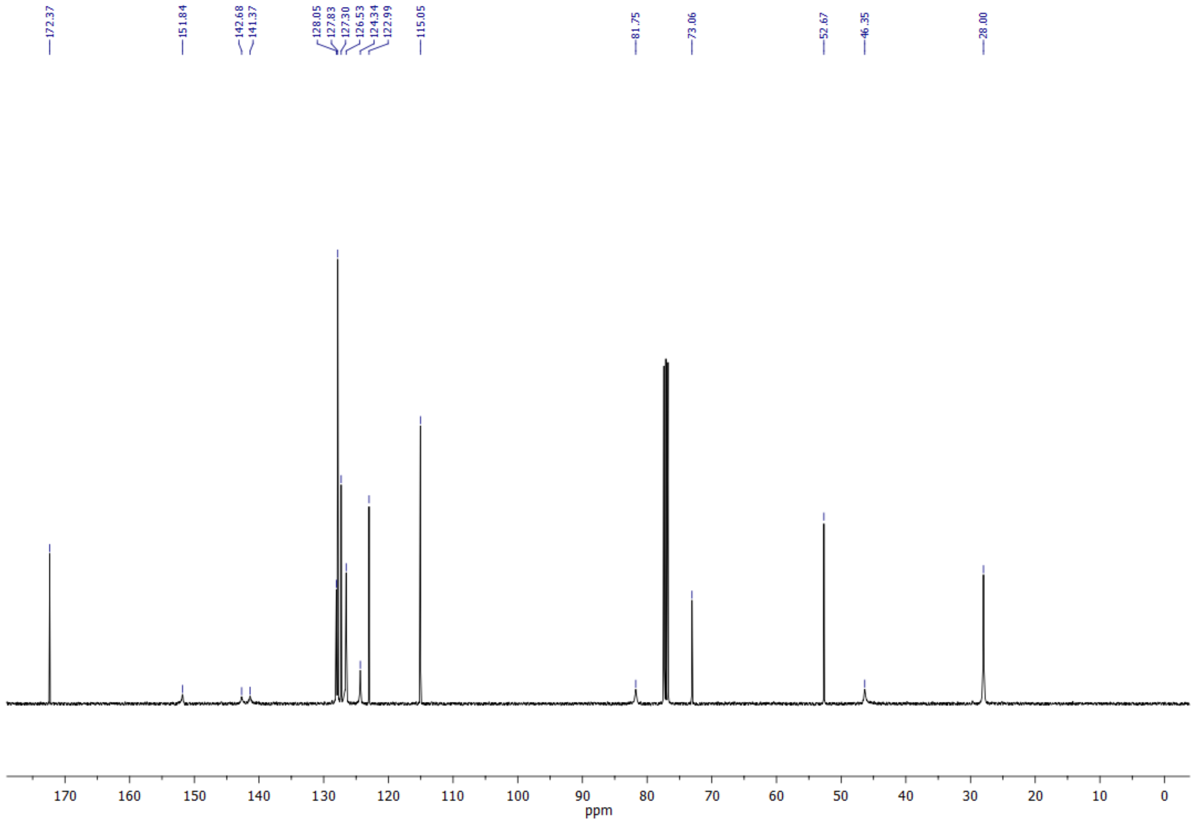
172.37
151.84
142.68
141.37
128.05
127.83
127.30
126.53
124.34
122.99
115.05
81.75
73.06
52.67
46.35
28.00
170 160 150 140 130 120 110 100 90 80 70 60 50 40 30 20 10 0
ppm

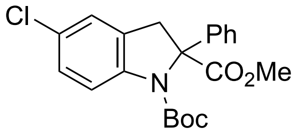

3b

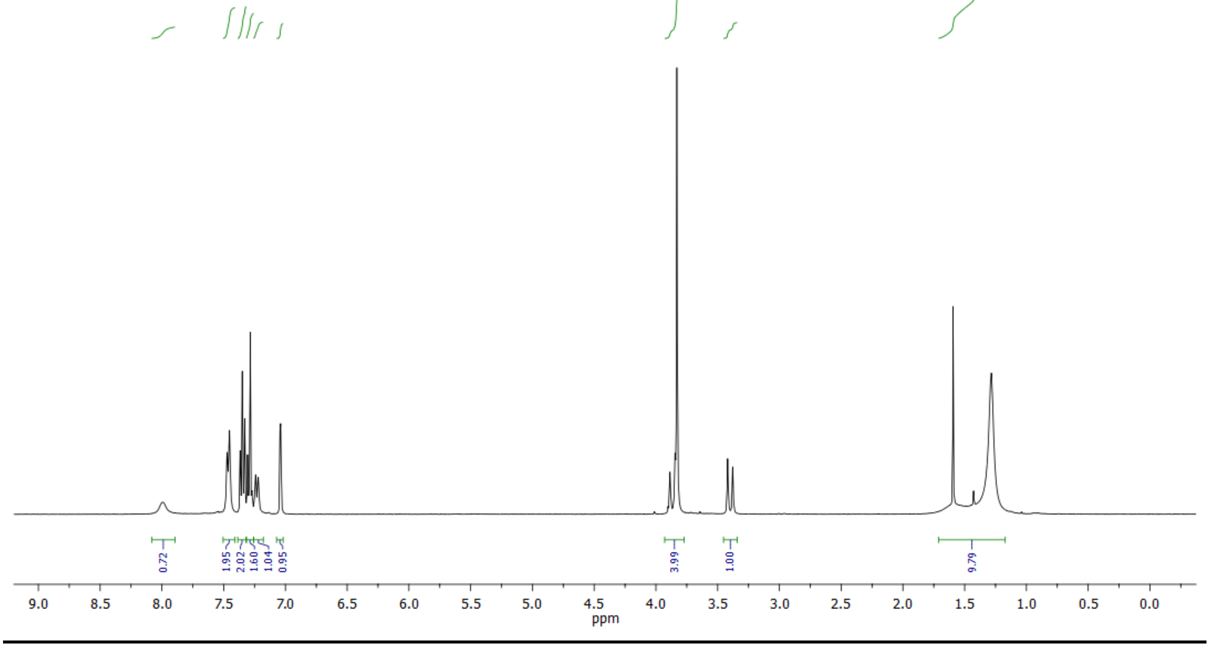

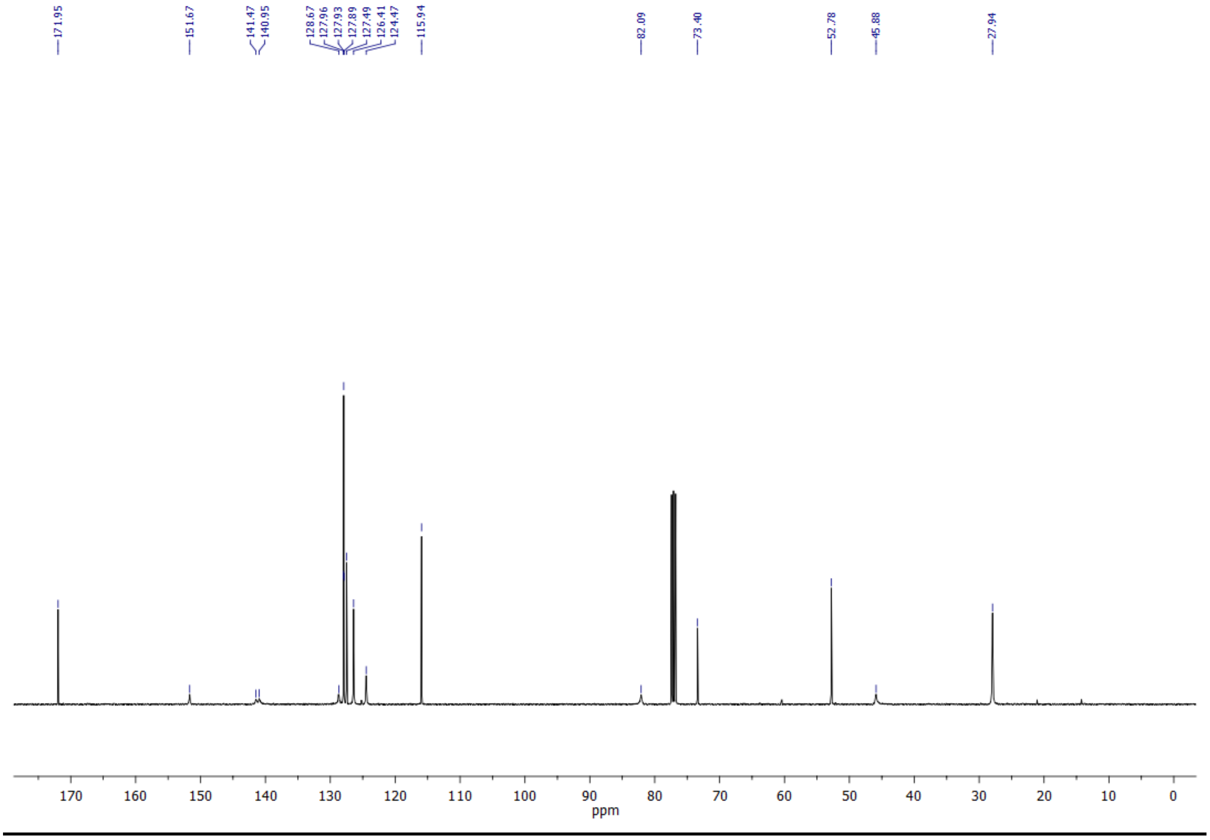

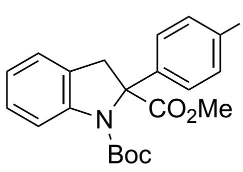
F
CO_2Me
N
Boc

3c

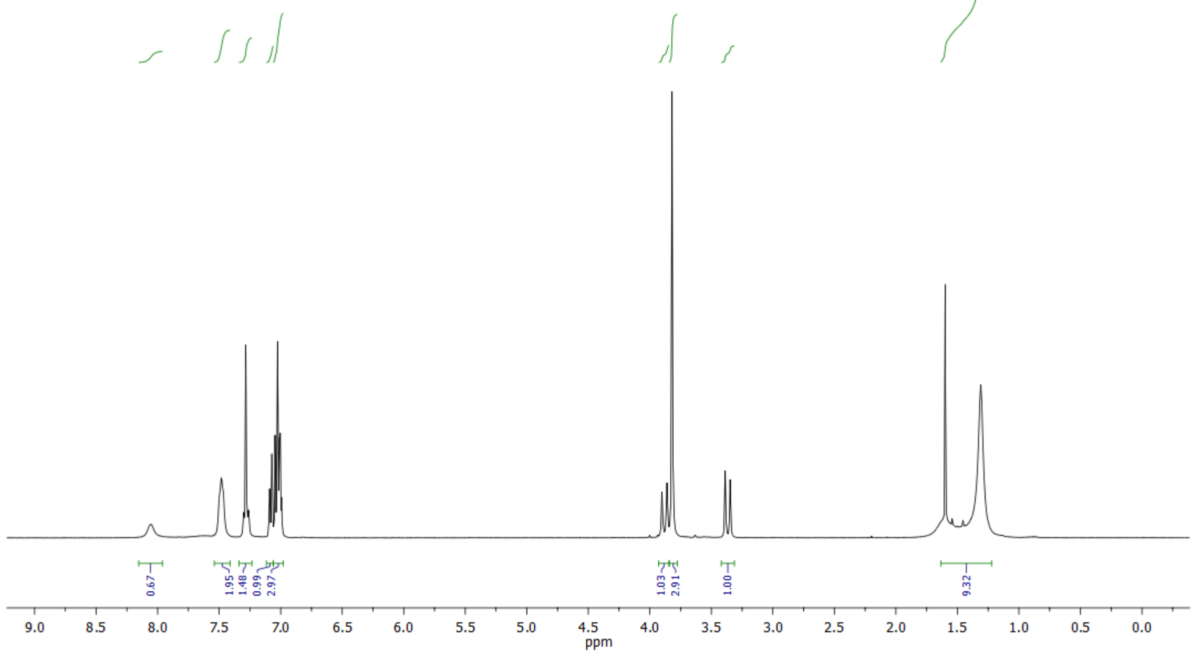
0.67
1.95
1.48
0.99
2.97
1.03
2.91
1.00
9.32
9.0 8.5 8.0 7.5 7.0 6.5 6.0 5.5 5.0 4.5 4.0 3.5 3.0 2.5 2.0 1.5 1.0 0.5 0.0
ppm

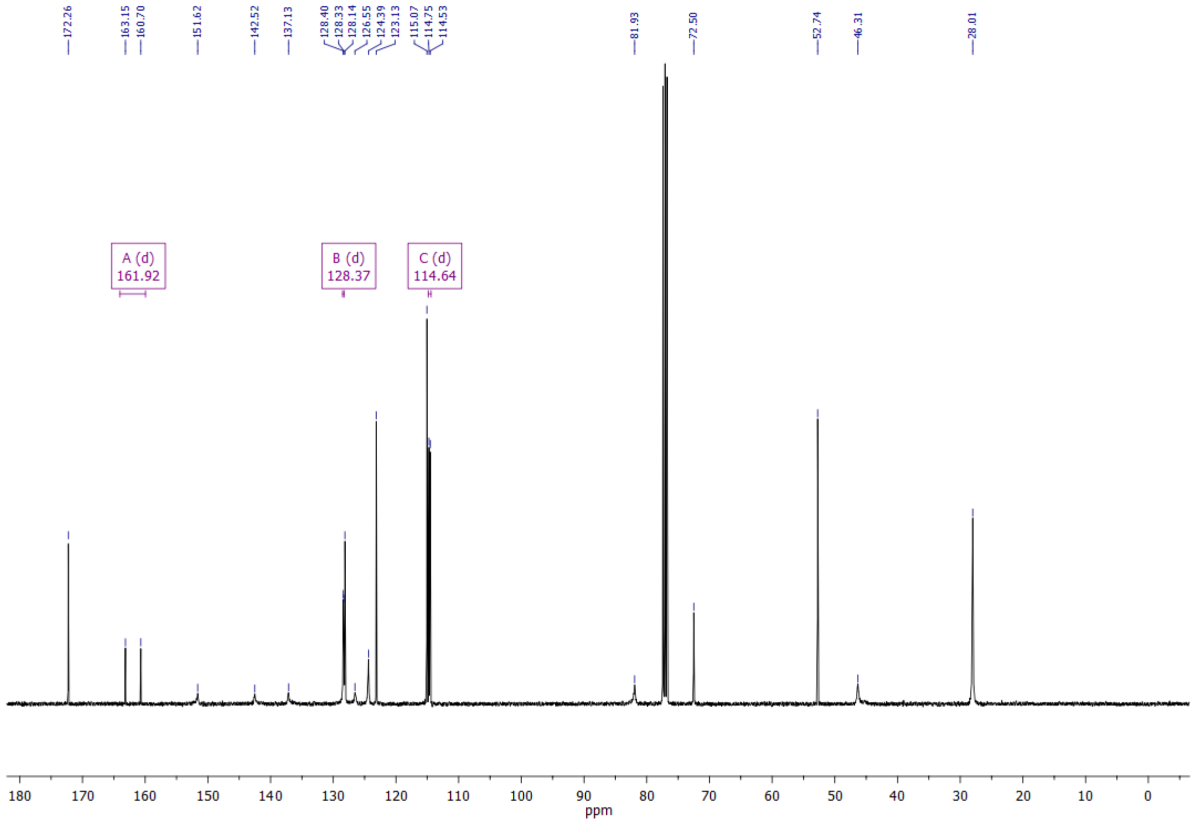
172.26
163.15
160.70
151.62
142.52
137.13
128.40
128.33
128.14
126.55
124.39
123.13
115.07
114.75
114.53
81.93
72.50
52.74
46.31
28.01
A (d)
161.92
B (d)
128.37
C (d)
114.64
180 170 160 150 140 130 120 110 100 90 80 70 60 50 40 30 20 10 0
ppm

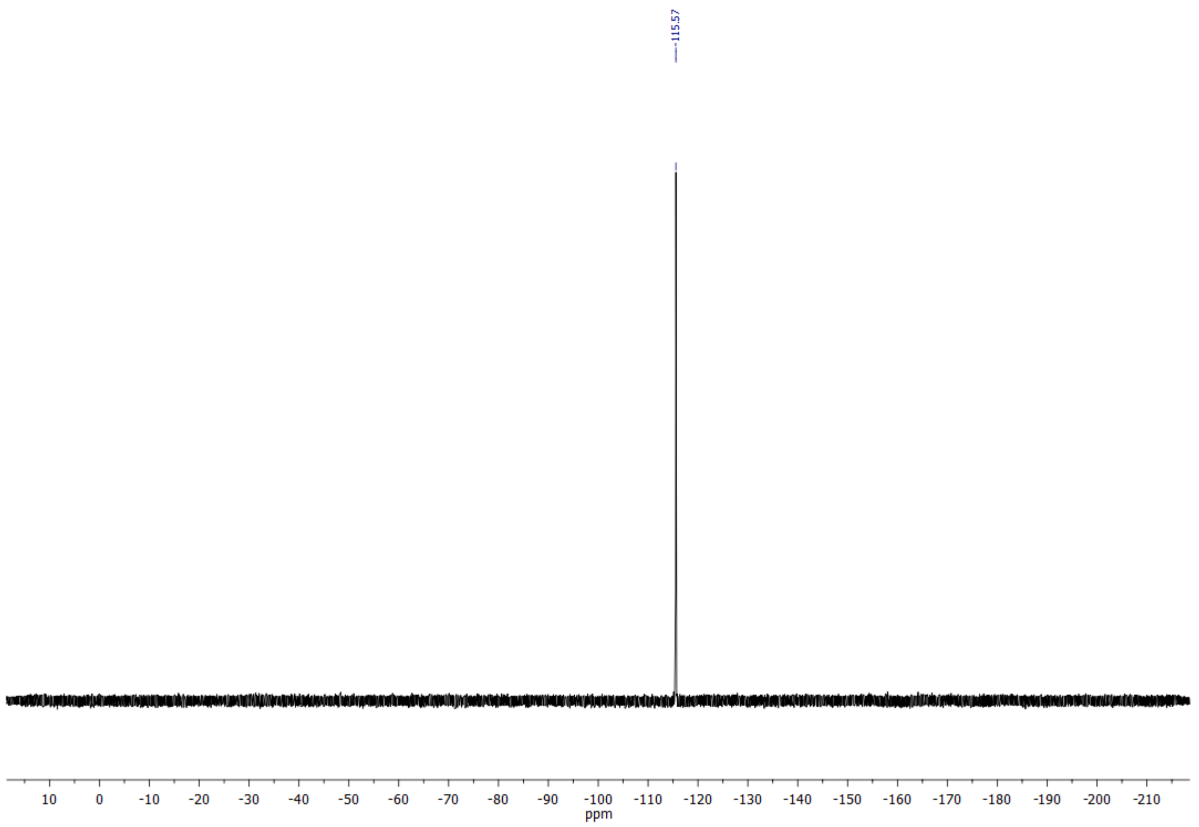

-115.57
10
0
-10
-20
-30
-40
-50
-60
-70
-80
-90
-100
ppm
-110
-120
-130
-140
-150
-160
-170
-180
-190
-200
-210

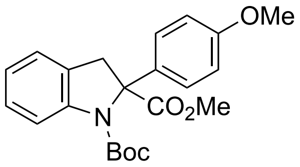
OMe
CO2Me
N
Boc

3d

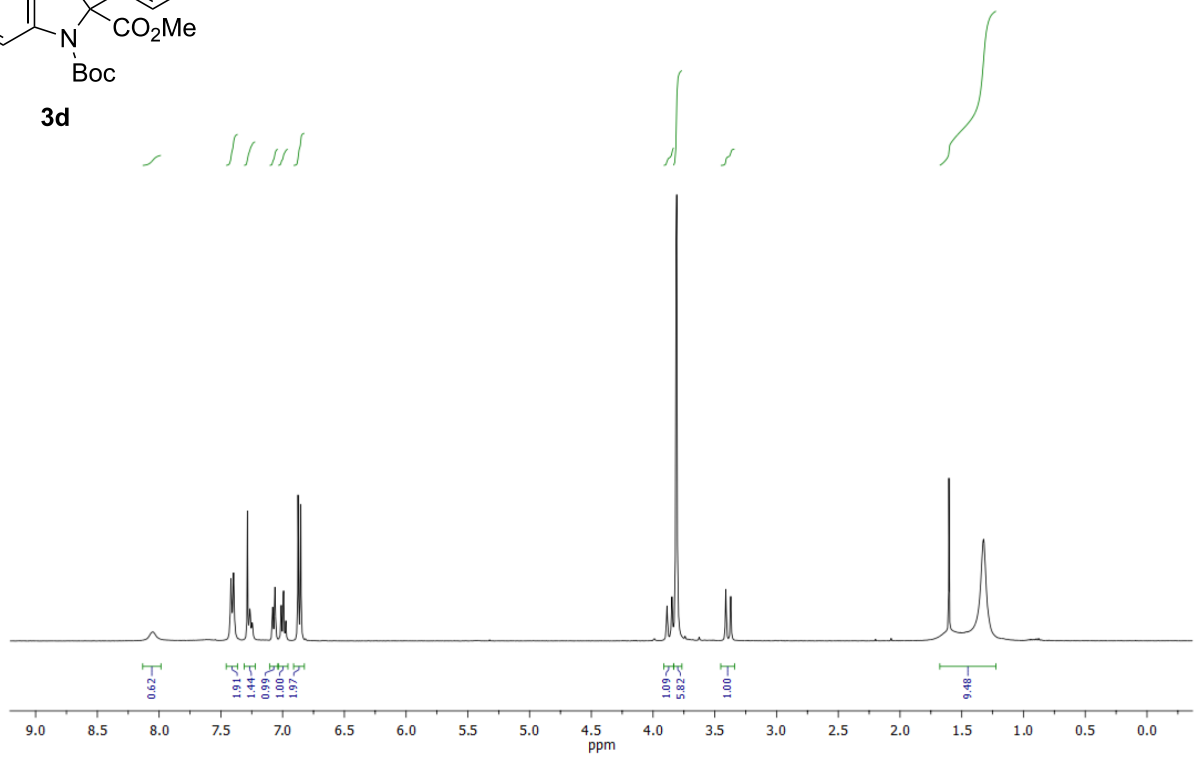
0.62
1.91
1.44
0.99
1.00
1.97
1.09
5.82
1.00
9.48
9.0 8.5 8.0 7.5 7.0 6.5 6.0 5.5 5.0 4.5 4.0 3.5 3.0 2.5 2.0 1.5 1.0 0.5 0.0
ppm

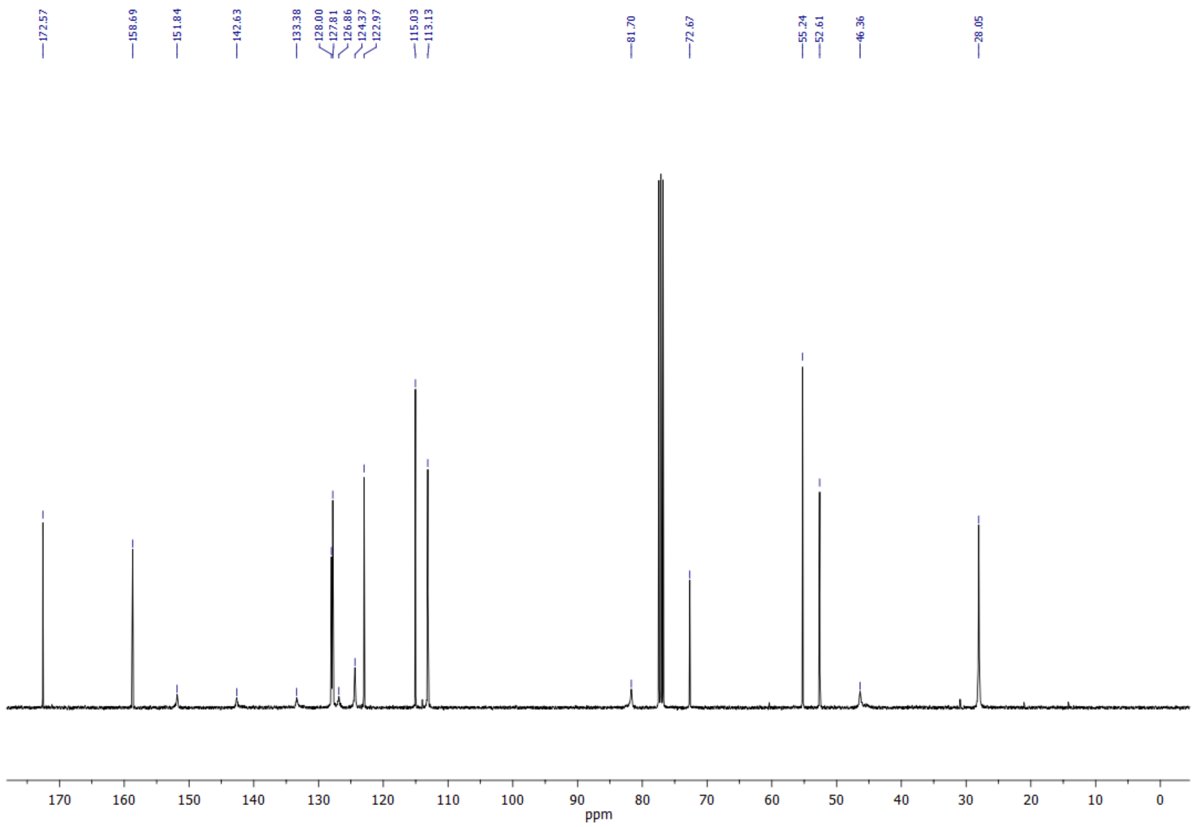
172.57
158.69
151.84
142.63
133.38
128.00
127.81
126.86
124.37
122.97
115.03
113.13
81.70
72.67
55.24
52.61
46.36
28.05
170 160 150 140 130 120 110 100 90 80 70 60 50 40 30 20 10 0
ppm

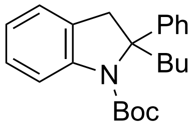
Ph
Bu
N
Boc

4a

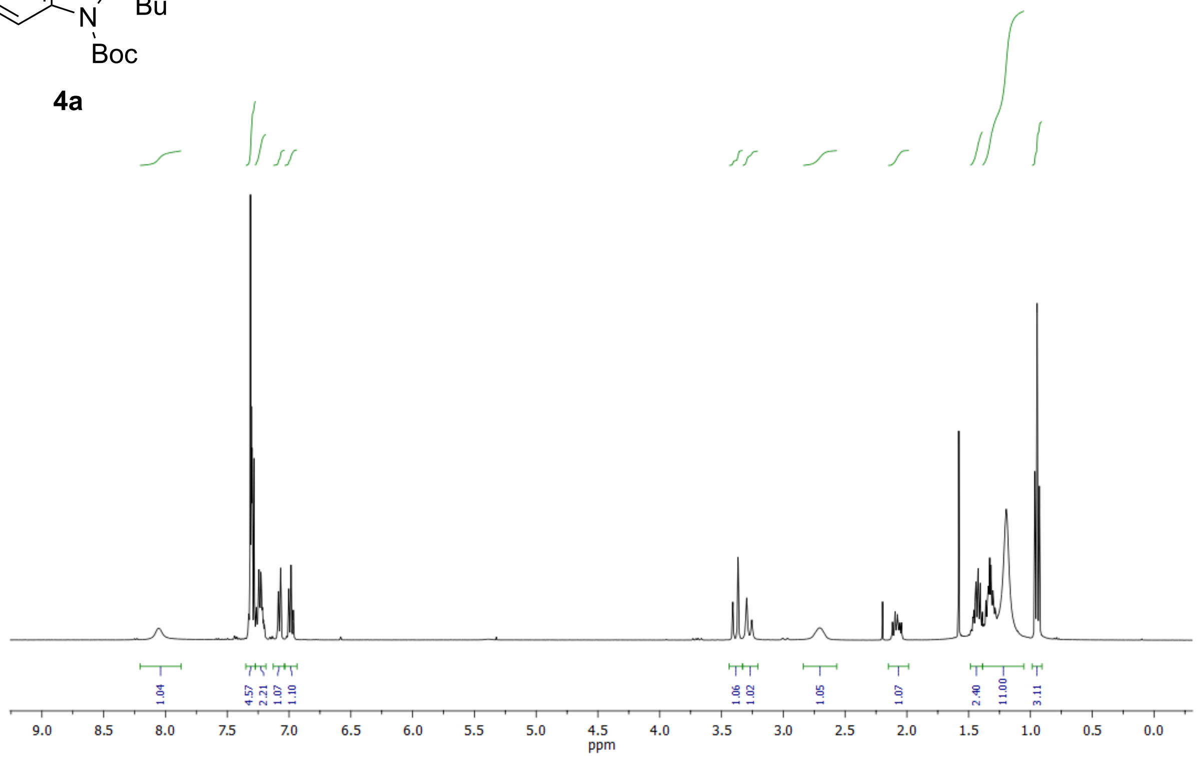
1.04
4.57
2.21
1.07
1.10
1.06
1.02
1.05
1.07
2.40
11.00
3.11
9.0
8.5
8.0
7.5
7.0
6.5
6.0
5.5
5.0
4.5
4.0
3.5
3.0
2.5
2.0
1.5
1.0
0.5
0.0
ppm

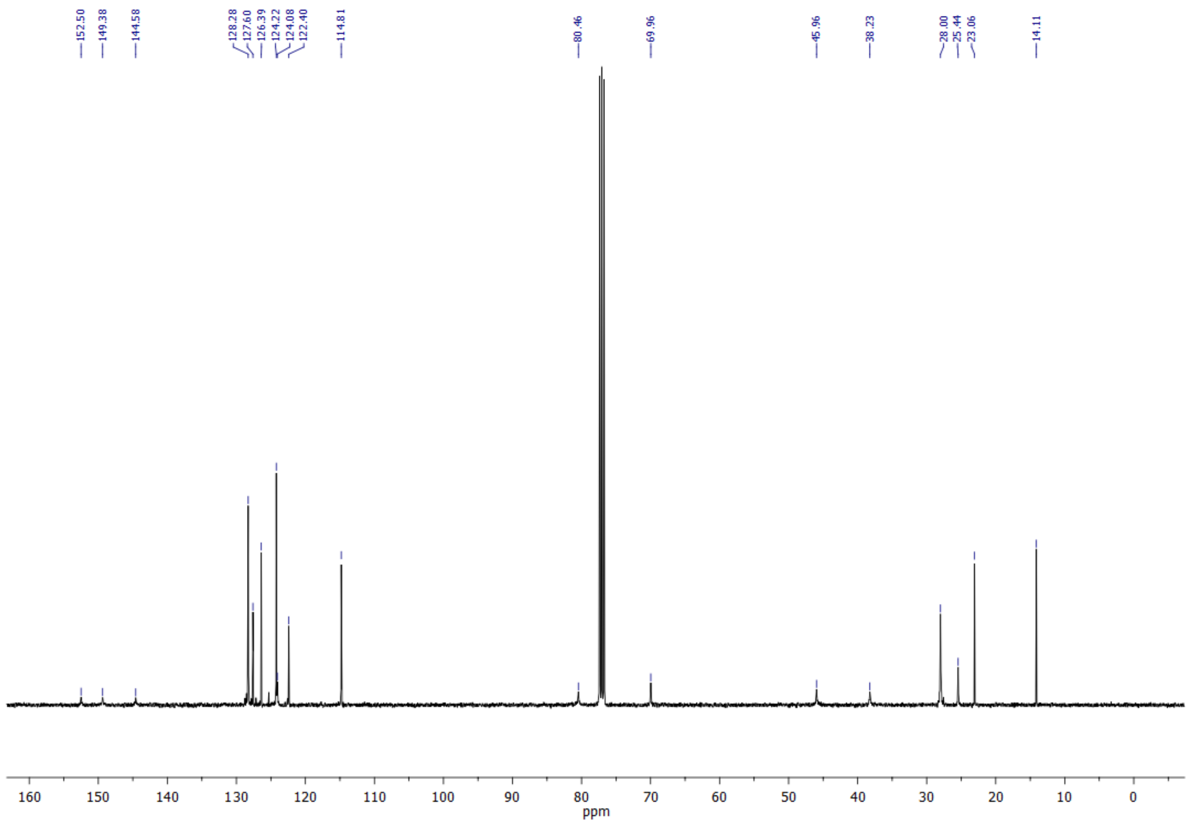
152.50
149.38
144.58
128.28
127.60
126.39
124.22
124.08
122.40
114.81
80.46
69.96
45.96
38.23
28.00
25.44
23.06
14.11
160
150
140
130
120
110
100
90
80
70
60
50
40
30
20
10
0
ppm

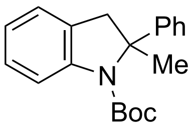
Ph
Me
N
Boc

5a

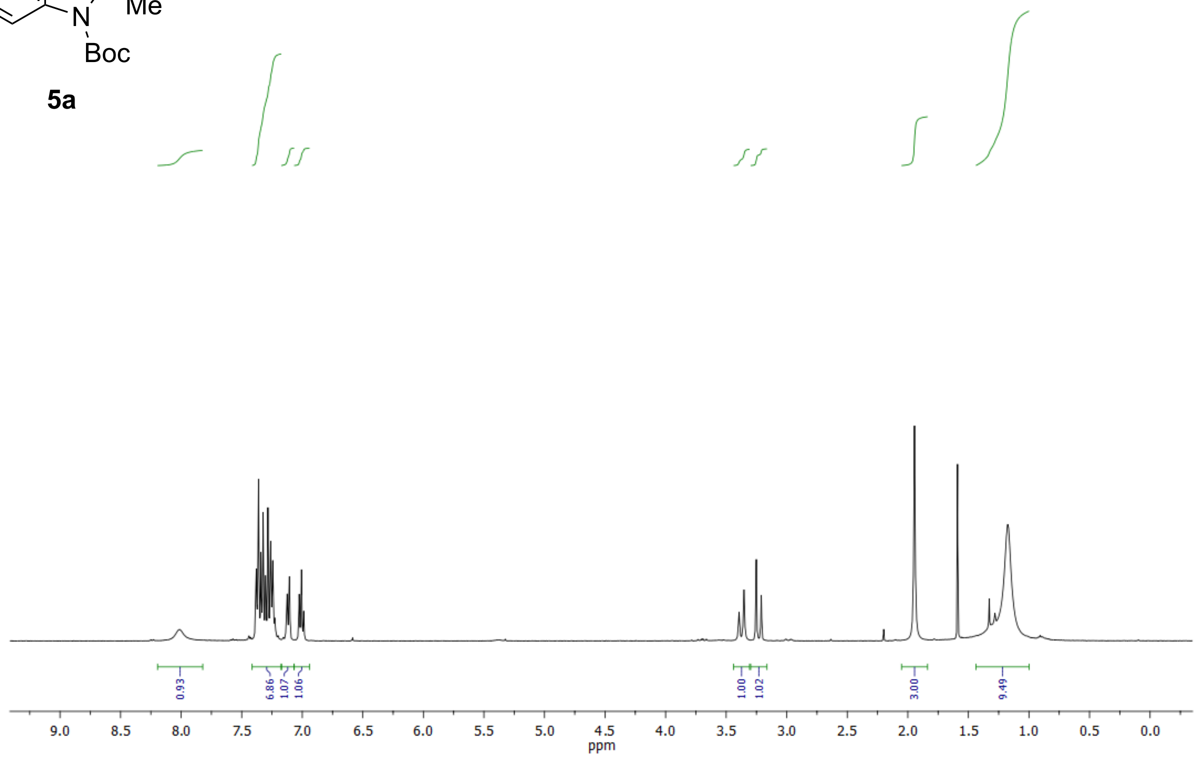
0.93
6.86
1.07
1.06
1.00
1.02
3.00
9.49
9.0 8.5 8.0 7.5 7.0 6.5 6.0 5.5 5.0 4.5 4.0 3.5 3.0 2.5 2.0 1.5 1.0 0.5 0.0
ppm

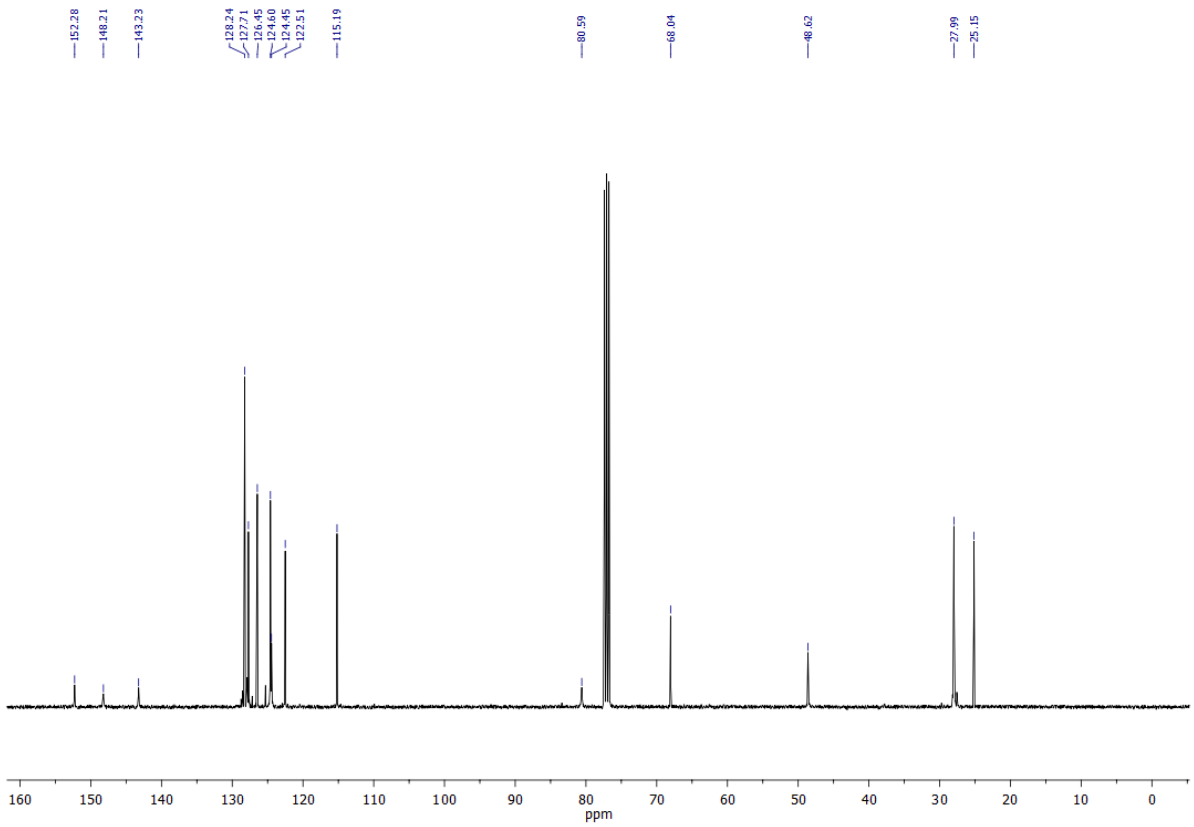
152.28
148.21
143.23
128.24
127.71
126.45
124.60
124.45
122.51
115.19
80.59
68.04
48.62
27.99
25.15
160 150 140 130 120 110 100 90 80 70 60 50 40 30 20 10 0
ppm

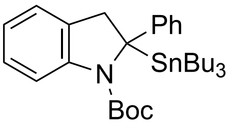
Ph
SnBu3
N
Boc

6a

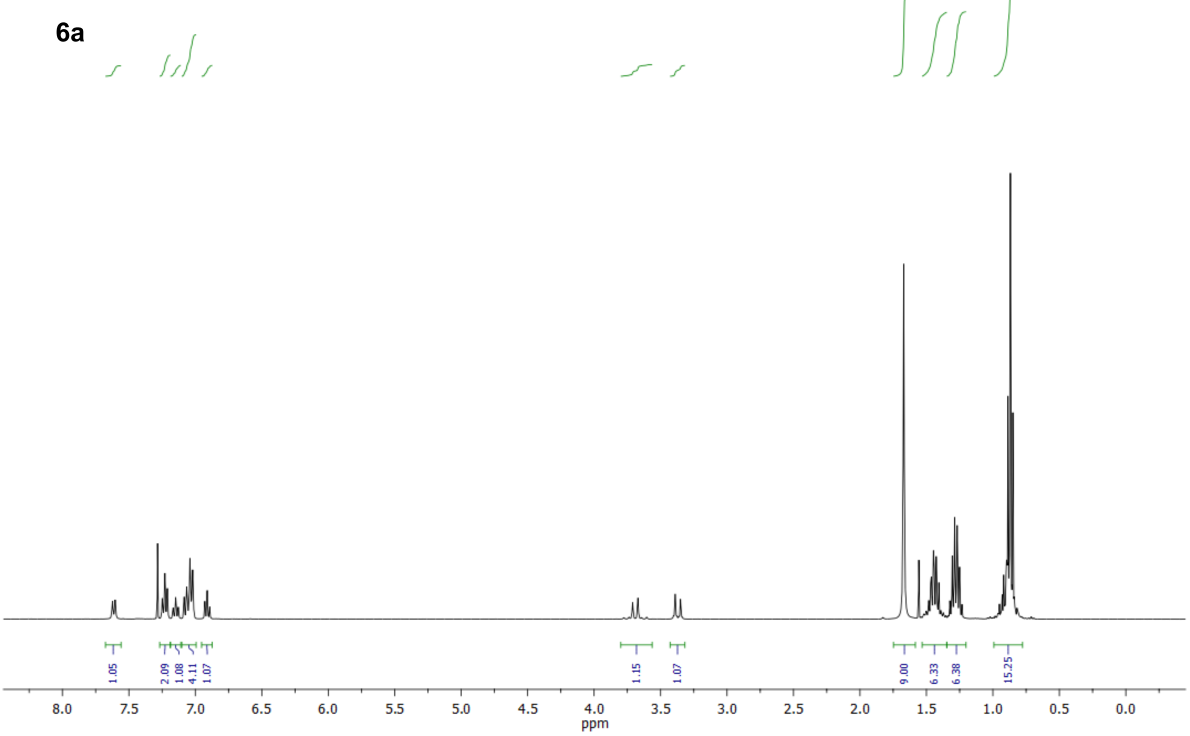
1.05
2.09
1.08
4.11
1.07
1.15
1.07
9.00
6.33
6.38
15.25
8.0
7.5
7.0
6.5
6.0
5.5
5.0
4.5
4.0
3.5
3.0
2.5
2.0
1.5
1.0
0.5
0.0
ppm

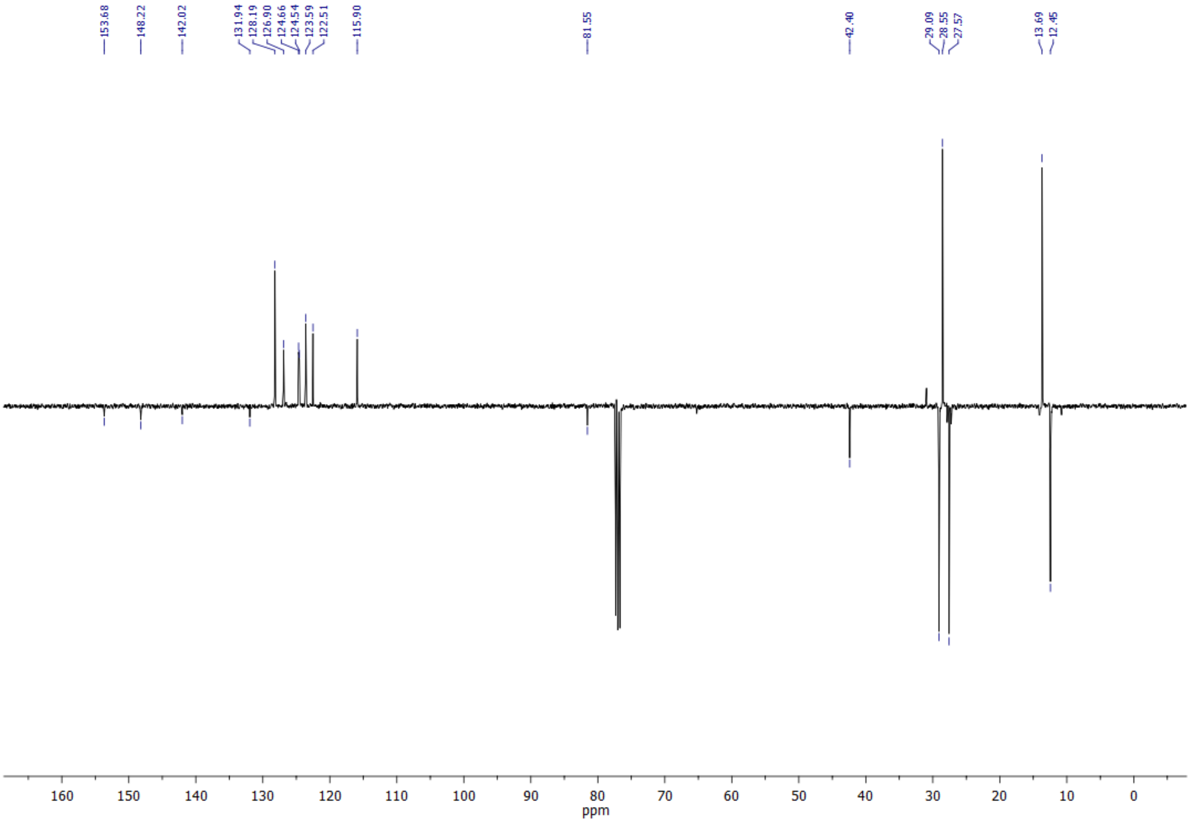
153.68
148.22
142.02
131.94
128.19
126.90
124.66
124.54
123.59
122.51
115.90
81.55
42.40
29.09
28.55
27.57
13.69
12.45
160
150
140
130
120
110
100
90
80
70
60
50
40
30
20
10
0
ppm

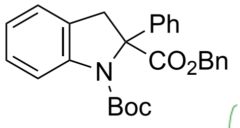

Ph
CO2Bn
N
Boc

7a

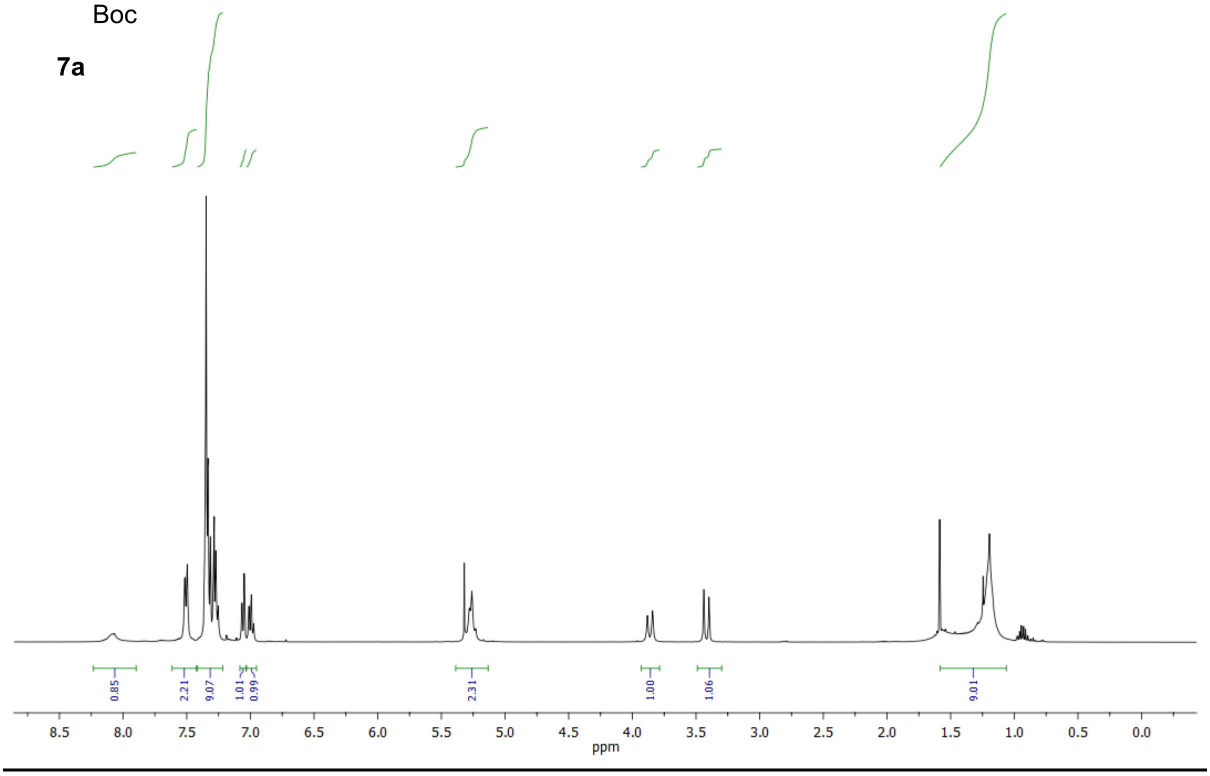

0.85
2.21
9.07
1.01
0.99
2.31
1.00
1.06
9.01
8.5
8.0
7.5
7.0
6.5
6.0
5.5
5.0
4.5
4.0
3.5
3.0
2.5
2.0
1.5
1.0
0.5
0.0
ppm

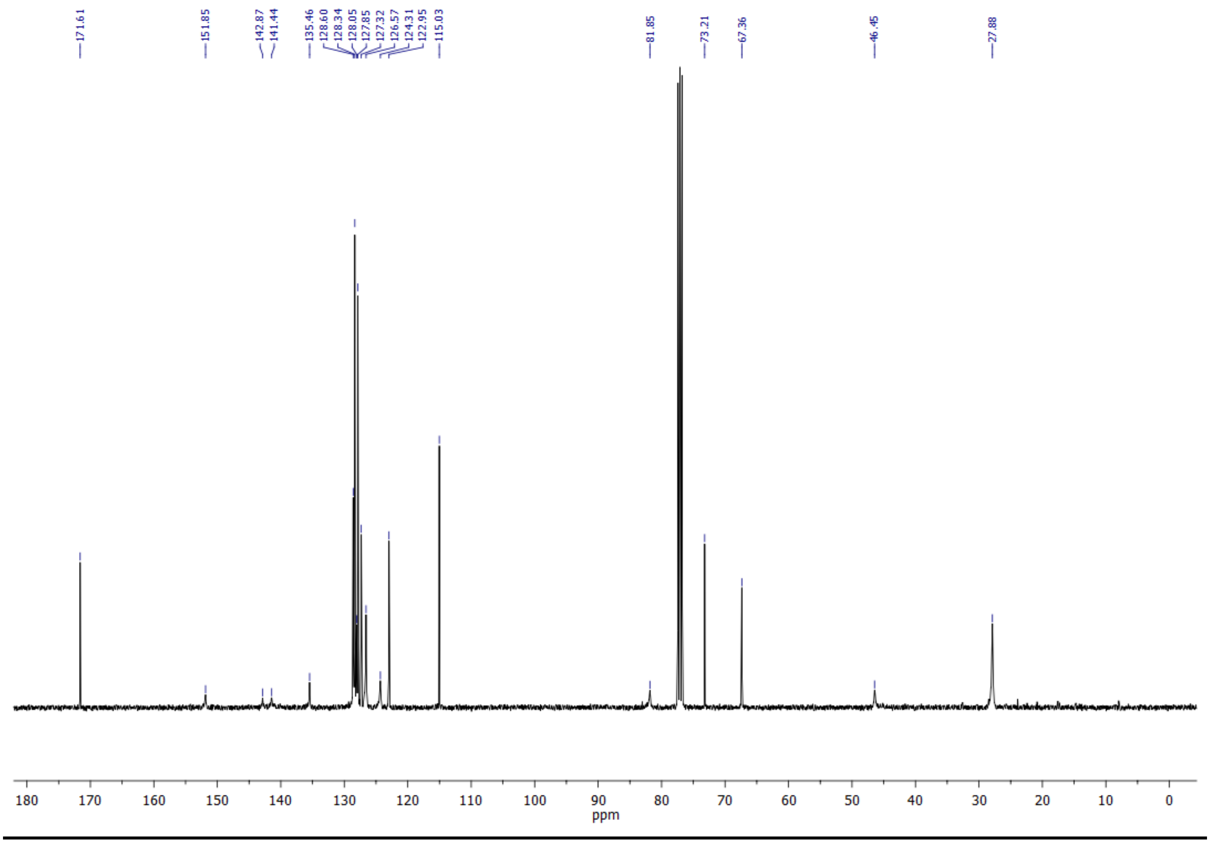

171.61
151.85
142.87
141.44
135.46
128.60
128.34
128.05
127.85
127.32
126.57
124.31
122.95
115.03
81.85
73.21
67.36
46.45
27.88
180
170
160
150
140
130
120
110
100
90
80
70
60
50
40
30
20
10
0
ppm

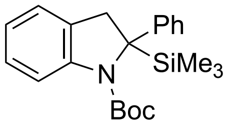
Ph
SiMe3
N
Boc

8a

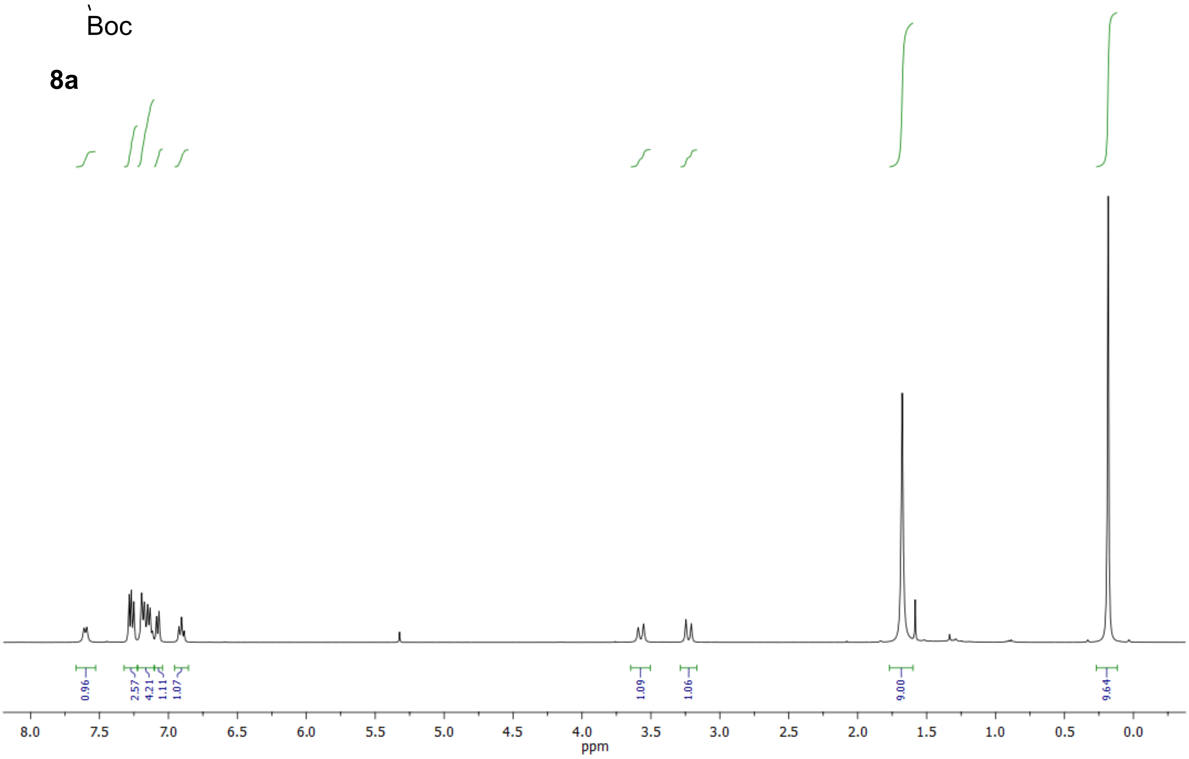
0.96
2.57
4.21
1.11
1.07
1.09
1.06
9.00
9.64
8.0
7.5
7.0
6.5
6.0
5.5
5.0
4.5
4.0
3.5
3.0
2.5
2.0
1.5
1.0
0.5
0.0
ppm

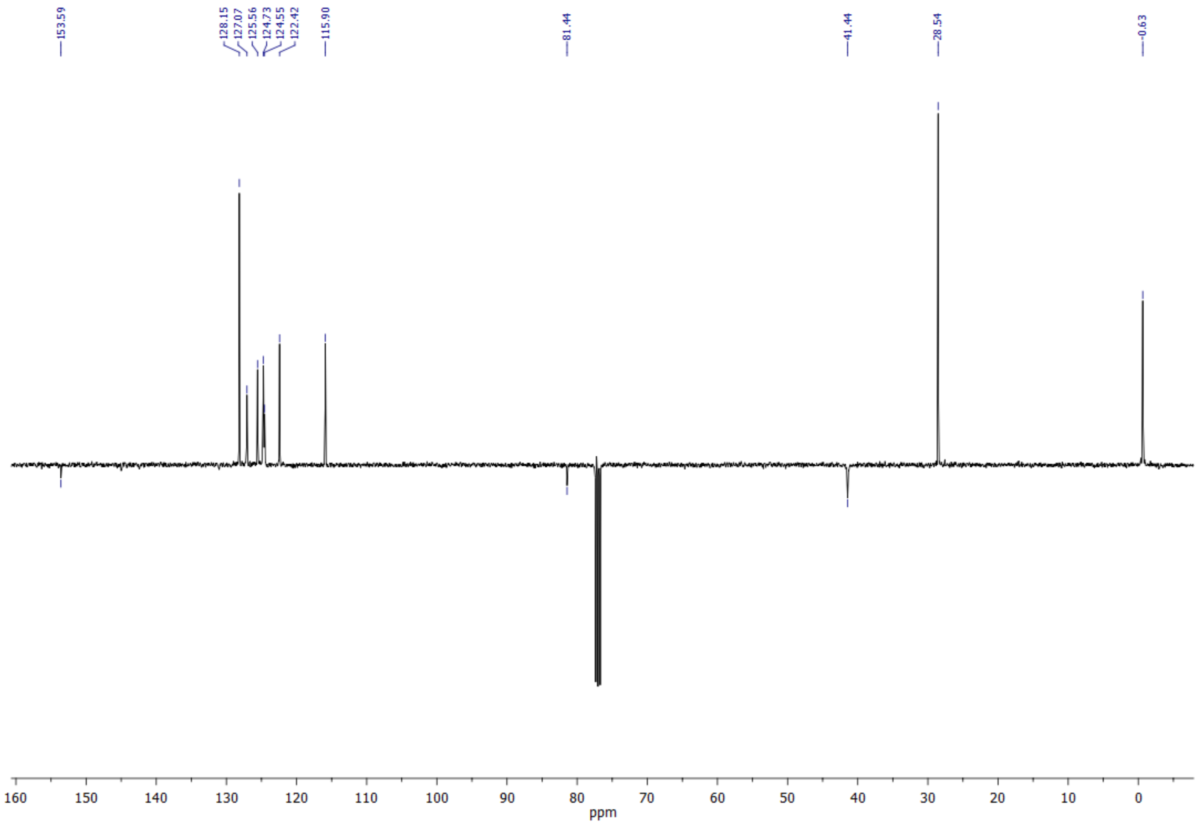
153.59
128.15
127.07
125.56
124.73
124.55
122.42
115.90
81.44
41.44
28.54
0.63
160
150
140
130
120
110
100
90
80
70
60
50
40
30
20
10
0
ppm

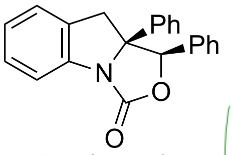

9aa (major)

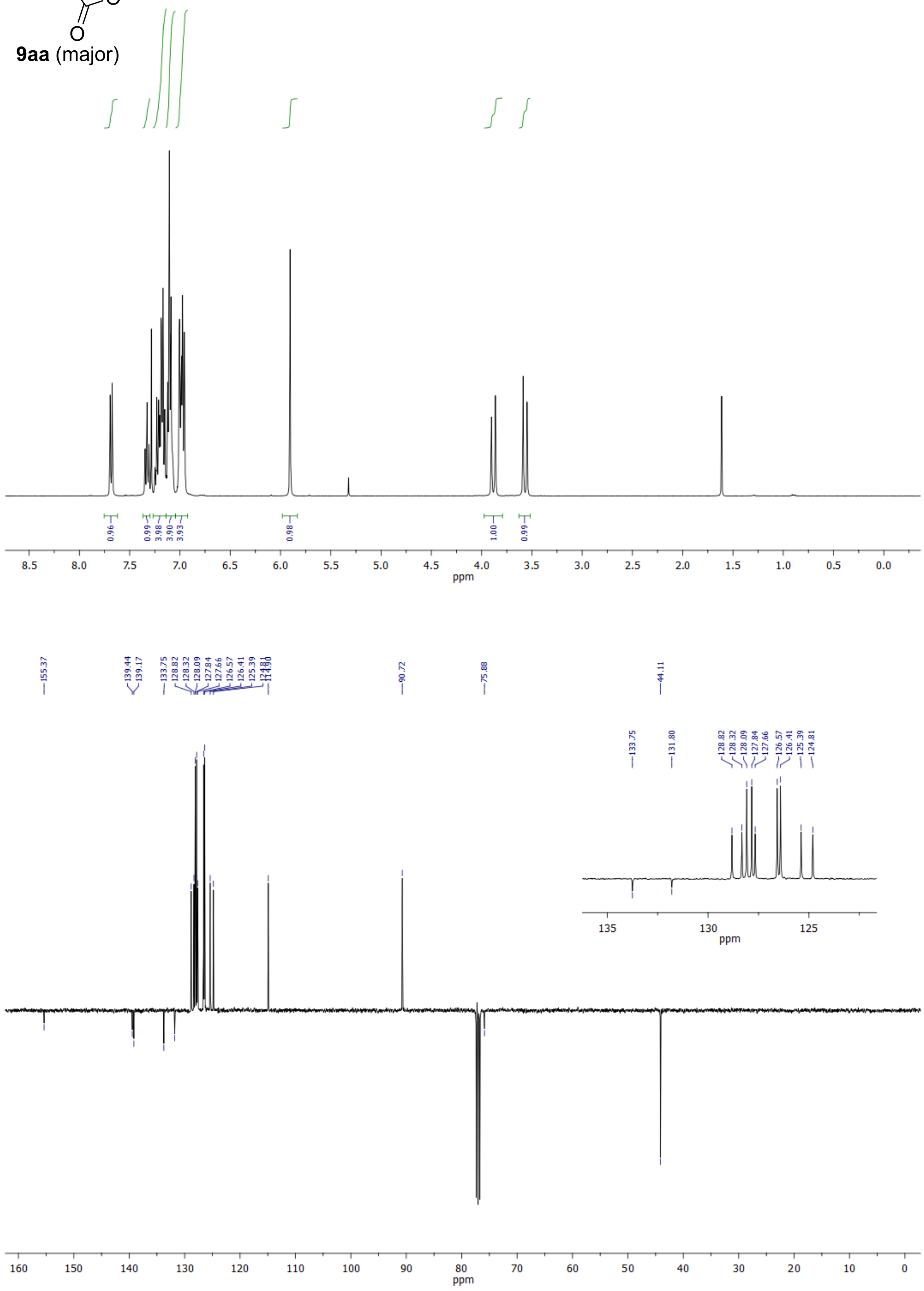

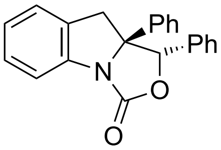

9ab (minor)

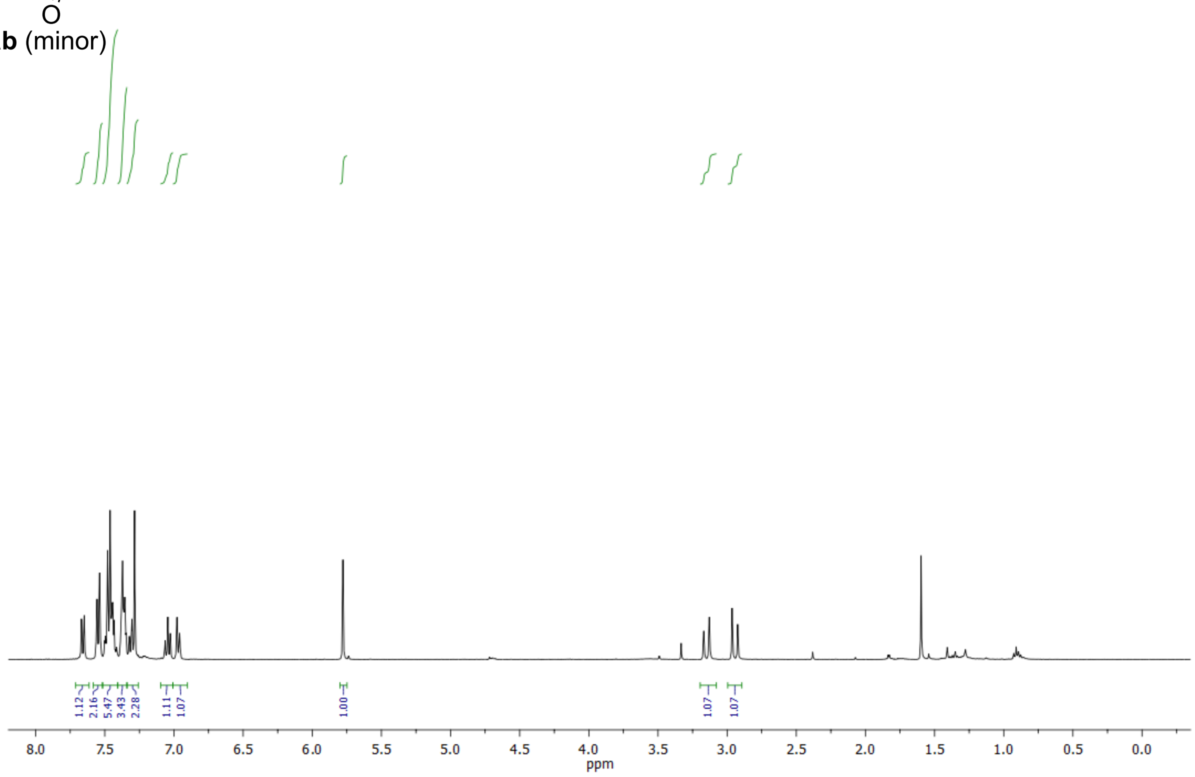

1.12
2.16
5.47
3.43
2.28
1.11
1.07
1.00
1.07
1.07
8.0 7.5 7.0 6.5 6.0 5.5 5.0 4.5 4.0 3.5 3.0 2.5 2.0 1.5 1.0 0.5 0.0
ppm

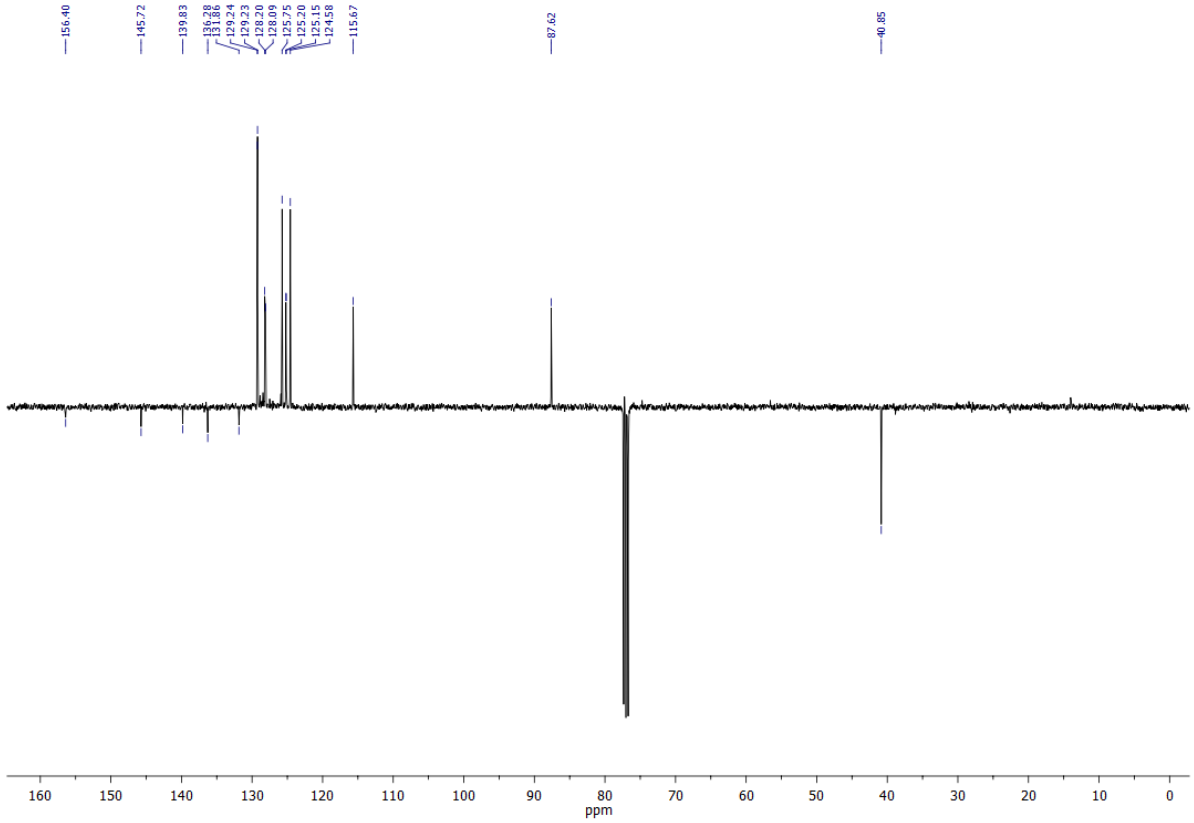

156.40
145.72
139.83
136.28
131.86
129.24
129.23
128.20
128.09
125.75
125.20
125.15
124.58
115.67
87.62
40.85
160 150 140 130 120 110 100 90 80 70 60 50 40 30 20 10 0
ppm

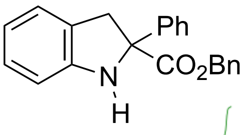
Ph
CO2Bn
N
H

10a

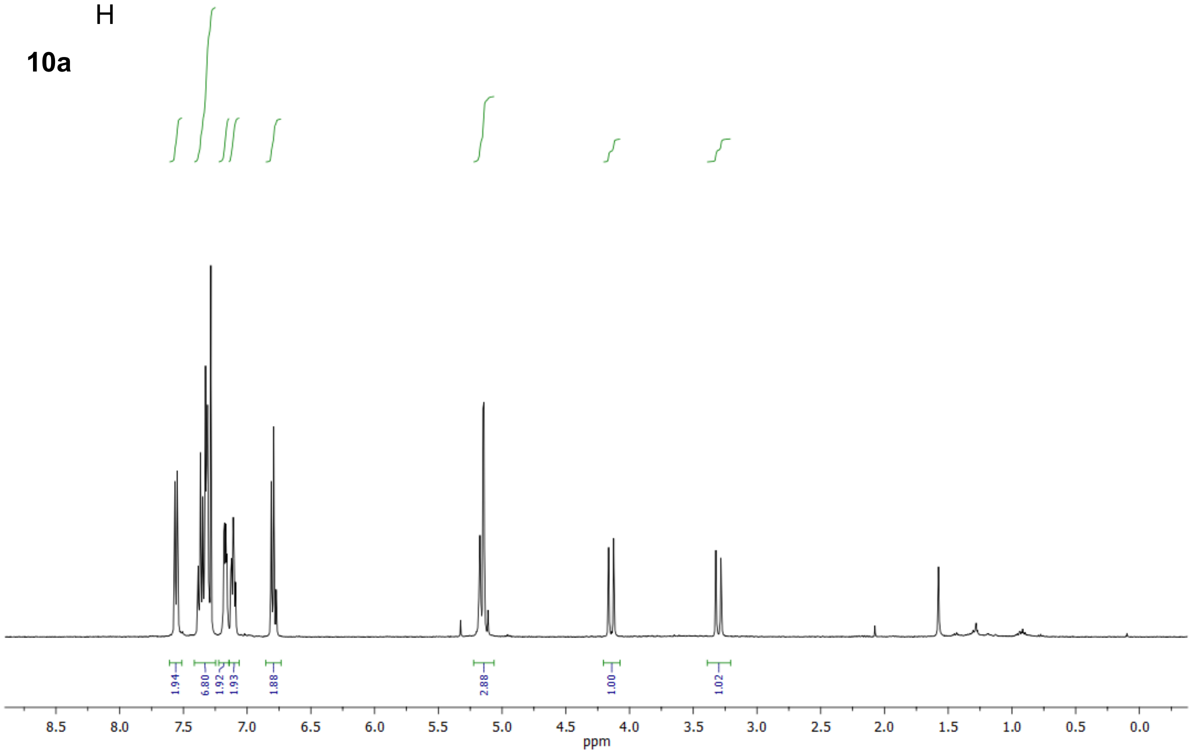
1.94
6.80
1.92
1.93
1.88
2.88
1.00
1.02
8.5 8.0 7.5 7.0 6.5 6.0 5.5 5.0 4.5 4.0 3.5 3.0 2.5 2.0 1.5 1.0 0.5 0.0
ppm

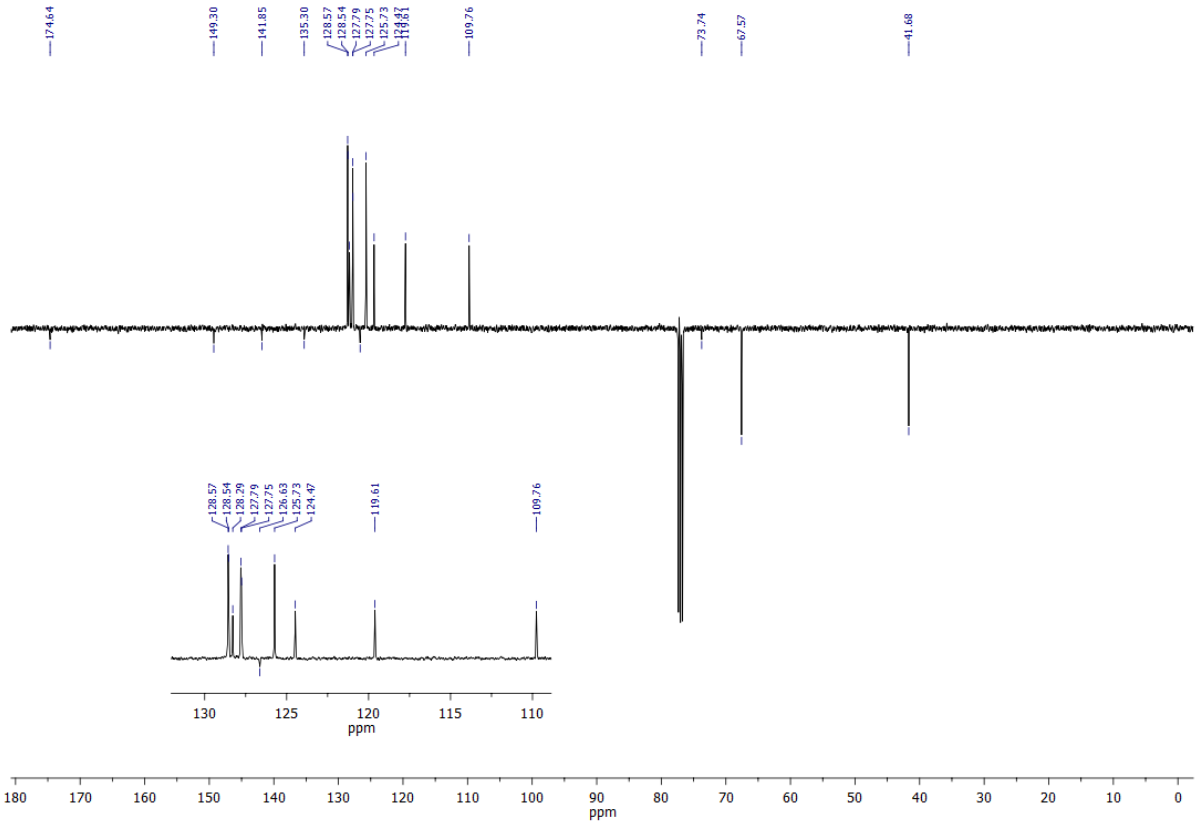
174.64
149.30
141.85
135.30
128.57
128.54
127.79
127.75
125.73
124.47
119.61
109.76
73.74
67.57
41.68
128.57
128.54
128.29
127.79
127.75
126.63
125.73
124.47
119.61
109.76
130 125 120 115 110
ppm
180 170 160 150 140 130 120 110 100 90 80 70 60 50 40 30 20 10 0
ppm

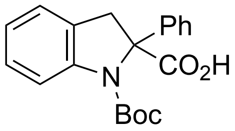

11a

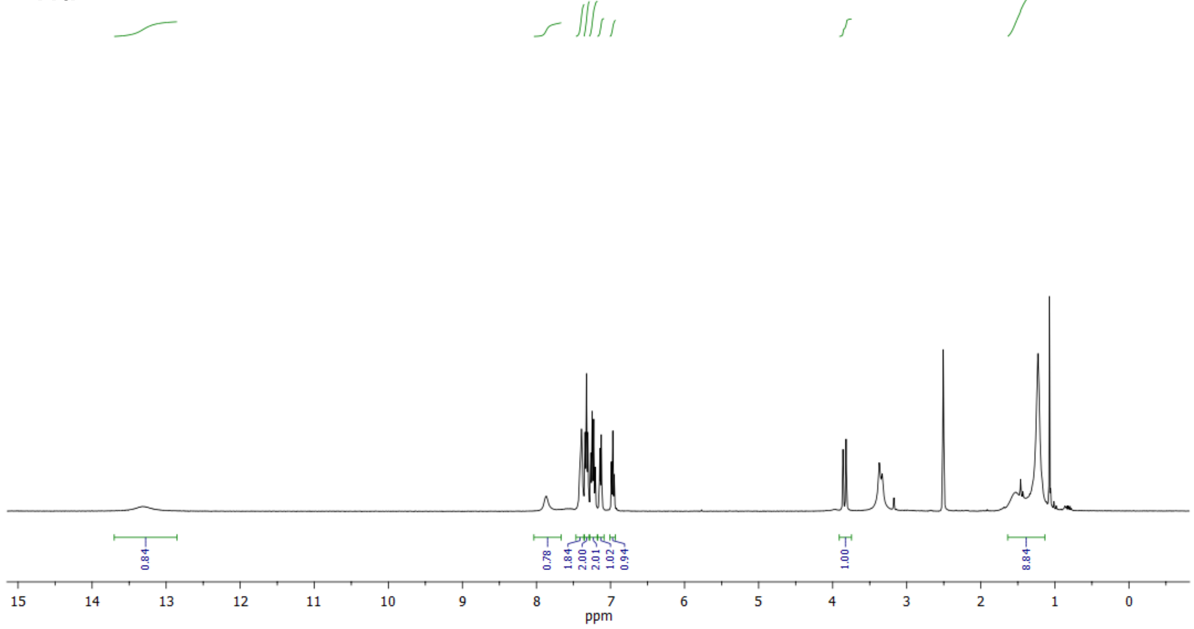

NMR spectra in DMSO-d_6

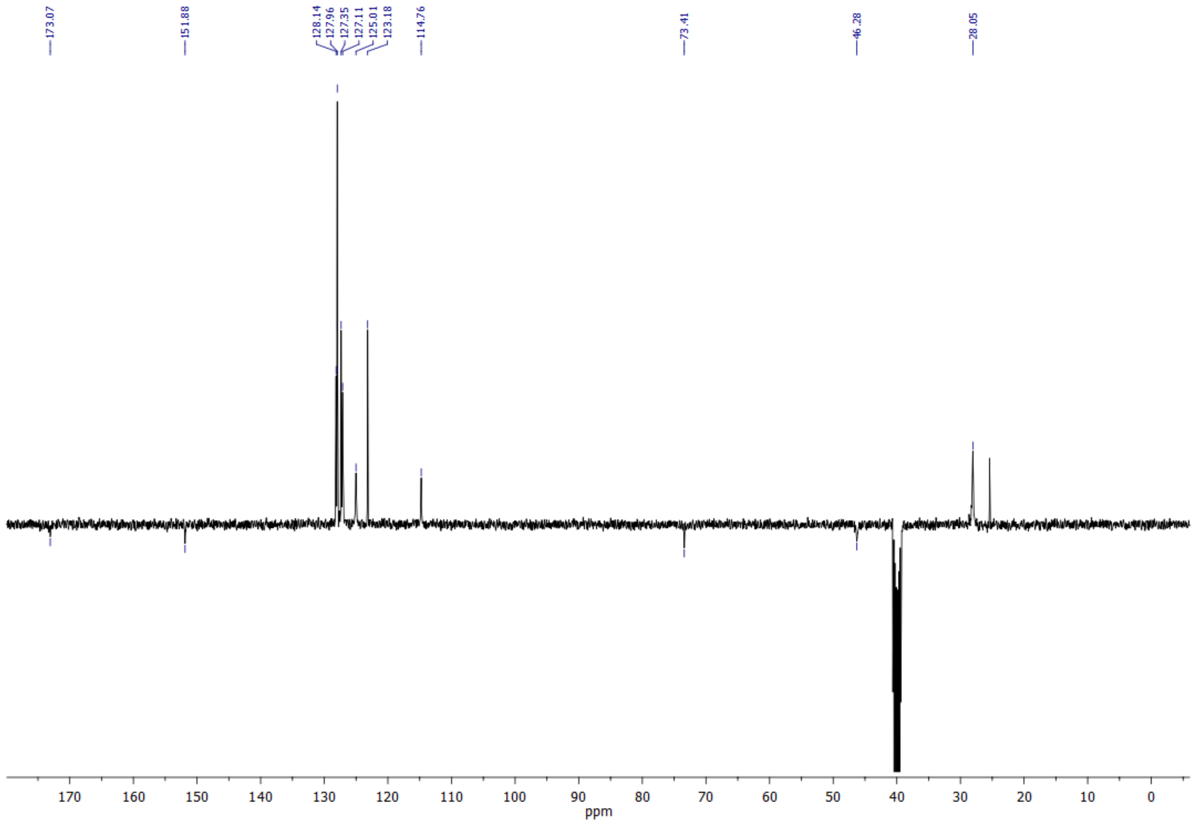

10. DFT data

Calculations were performed on the rotamers of **2a** and **2d**, including the transition states for rotation of the Boc group from rotamer 1 to rotamer 2 for both **2a** and **2d**. The thermochemistry data for each structure were collected at 223 K and 195 K.

Calculations on the rotamers for **2a** using the 6-311G(d,p) and def2tzvp basis sets at 195 K are shown in Table S-15.

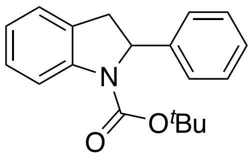

2a-1

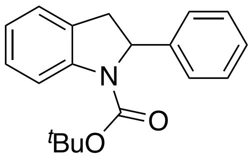

2a-2

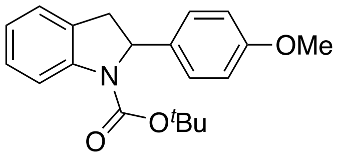

2d-1

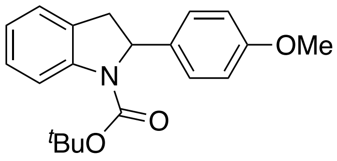

2d-2

Method	ΔG (kJ/mol)	ΔG‡ (kJ/mol)
B3LYP//6-311G**	3.4	55.5
B3LYP-D3BJ//6-311G**	7.9	58.2
B3LYP-D3BJ//def2TZVP	3.0	57.5
Experiment	2.9	57.6

Table S-15. Gibbs energy difference between rotamers and activation Gibbs Energy at 195 K for conversion from major–minor rotamer (**2a-1** to **2a-2**) for the B3LYP//6-311G**, B3LYP-D3BJ//6-311G**, and B3LYP-D3BJ//def2TZVP methods.

From Table S-15, it is clear that the B3LYP//6-311G** method reproduces the relative free energy between the rotamers well, but it underestimates the barrier. Surprisingly, inclusion of a dispersion correction improves only the barrier height for rotamer inversion. In contrast, the relative free energy between the rotamers increases, resulting in much worse agreement with experiment. Improving the basis set (def2TZVP) leads to excellent agreement with the experimental data.

In summary, using the def2tzvp basis set including the D3BJ correction:
Calculations showed structure **2a-1** was lower in energy than structure **2a-2**. At 223 K the difference in energy between the two rotamers was ~2.7 $kJmol^{-1}$. This corresponds to K = 0.23.
At 195 K the activation parameters for rotation of the Boc group from rotamer **2a-1** to **2a-2** through an anticlockwise transition state were calculated to be:
$\Delta G^{\ddagger} \approx 57.5$ $kJmol^{-1}$, $\Delta H^{\ddagger} \approx 55.7$ $kJmol^{-1}$ and $\Delta S^{\ddagger} \approx -9.45$ $Jmol^{-1}K^{-1}$.
These compare to those determined by NMR spectroscopy (see page S-22) at 195 K:
$\Delta G^{\ddagger} \approx 57.6$ $kJmol^{-1}$, $\Delta H^{\ddagger} \approx 53.4$ $kJmol^{-1}$ and $\Delta S^{\ddagger} \approx -21.5$ $Jmol^{-1}K^{-1}$.

Calculations showed structure **2d-1** was lower in energy than structure **2d-2**. At 223 K the difference in energy between the two rotamers was ~3.4 $kJmol^{-1}$. This corresponds to K = 0.16.
At 195 K the activation parameters for rotation of the Boc group from rotamer **2d-1** to **2d-2** through an anticlockwise transition state were calculated to be:
$\Delta G^{\ddagger} \approx 57.2$ $kJmol^{-1}$, $\Delta H^{\ddagger} \approx 55.3$ $kJmol^{-1}$ and $\Delta S^{\ddagger} \approx -9.64$ $Jmol^{-1}K^{-1}$.
These compare to those determined by NMR spectroscopy (see page S-25) at 195 K:
$\Delta G^{\ddagger} \approx 58.9$ $kJmol^{-1}$, $\Delta H^{\ddagger} \approx 58.7$ $kJmol^{-1}$ and $\Delta S^{\ddagger} \approx -0.9$ $Jmol^{-1}K^{-1}$.

For completeness, calculations using the 6-311G(d,p) and def2tzvp basis sets are shown below.

10.1 Calculations Performed Using the 6-311G(d,p) Basis Set without dispersion

N-Boc-2-phenylindoline 2a-1

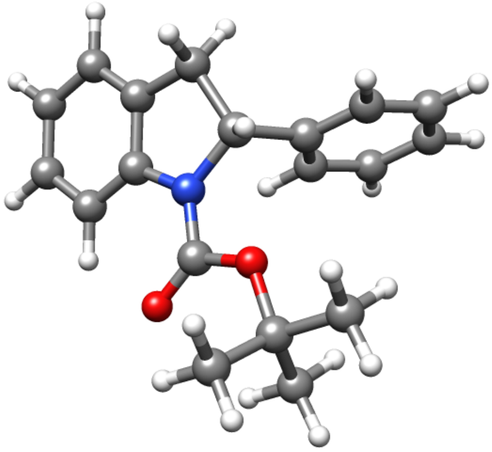

Route : # opt freq b3lyp/6-311g(d,p) scrf=(solvent=thf)
geom=connectivity int=ultrafine

SMILES : CC(C)(C)OC(=O)N2c1ccccc1CC2c3ccccc3

Formula : $C_{19}H_{21}NO_2$

Charge : 0

Multiplicity : 1

Dipole : 3.6089 Debye

Energy : -942.15661094 a.u.

Gibbs Energy : -941.84776800 a.u.

Number of imaginary frequencies : 0

Cartesian Coordinates (XYZ format)

43

C	-4.71816100	-0.77282600	-0.21560500
C	-3.95039400	-1.68665800	-0.93544100
C	-4.09477100	0.14473400	0.63662200
C	-2.71320500	0.13298500	0.75208800
C	-1.95081800	-0.78423600	0.01858100
C	-2.55633600	-1.70777100	-0.83265900
C	-1.81178400	0.97428900	1.62095600
C	-0.37724800	0.64842000	1.10197000
H	-1.91169300	0.68878500	2.67282000
H	-2.02503000	2.04176300	1.54935900
N	-0.57796400	-0.58083000	0.28873700
H	-5.79738000	-0.77279800	-0.31509800
H	-4.68520400	0.85594500	1.20429900

H	-4.43757400	-2.39655700	-1.59458700
H	-1.96074500	-2.41401700	-1.38963600
C	0.43670400	-1.42514500	-0.09514700
O	0.27466400	-2.42376100	-0.77315300
O	1.61238000	-0.98631200	0.39325500
C	2.88617200	-1.68673000	0.10336700
C	2.84870500	-3.10034900	0.68749900
H	2.60547900	-3.06393200	1.75244700
H	3.83333500	-3.56242600	0.57942800
H	2.11282600	-3.71883300	0.17700100
C	3.15356900	-1.67976300	-1.40317100
H	4.15172600	-2.08183200	-1.59457600
H	3.11801000	-0.65800500	-1.78900900
H	2.42412800	-2.28626500	-1.93719400
C	3.91019300	-0.82302600	0.83921300
H	3.69267700	-0.79575200	1.90924700
H	3.89806000	0.19903100	0.45517400
H	4.91181100	-1.23580200	0.69987200
H	0.28620100	0.41206700	1.93183600
C	0.23210100	1.79357100	0.30706500
C	-0.03359900	1.97624900	-1.05324700
C	1.03945100	2.72552100	0.96631800
C	0.49445800	3.07049900	-1.73686600
C	1.56642500	3.82124800	0.28525500
C	1.29446000	3.99751500	-1.07056700
H	1.26244300	2.58982100	2.01992500
H	2.19475100	4.53134500	0.81135700
H	1.70665000	4.84634300	-1.60426300
H	-0.64807000	1.25934200	-1.58520800
H	0.28145200	3.19701100	-2.79255000

Frequencies

Mode	IR frequency	IR intensity	Raman intensity
1	22.46090000	0.23300000	0.00000000
2	31.11220000	0.26650000	0.00000000
3	34.19010000	0.01170000	0.00000000
4	38.76890000	1.00410000	0.00000000
5	70.33750000	2.29910000	0.00000000
6	94.46140000	0.73900000	0.00000000
7	99.99650000	0.62450000	0.00000000
8	127.10140000	0.42180000	0.00000000
9	174.38000000	1.19900000	0.00000000
10	203.15780000	0.31150000	0.00000000
11	214.44720000	5.94370000	0.00000000
12	219.46070000	0.64270000	0.00000000
13	224.38880000	7.18580000	0.00000000
14	250.13750000	0.25010000	0.00000000
15	266.61460000	0.12370000	0.00000000
16	274.79950000	1.22650000	0.00000000

17	293.46970000	1.92270000	0.00000000
18	329.76020000	14.45710000	0.00000000
19	342.89990000	6.50690000	0.00000000
20	353.58470000	2.56050000	0.00000000
21	406.69220000	4.26250000	0.00000000
22	414.45210000	0.23160000	0.00000000
23	428.62300000	1.05090000	0.00000000
24	433.98570000	5.11990000	0.00000000
25	457.61560000	4.34760000	0.00000000
26	469.73050000	5.48740000	0.00000000
27	486.88580000	1.15390000	0.00000000
28	543.06470000	3.68040000	0.00000000
29	560.74580000	3.83130000	0.00000000
30	595.72370000	15.23150000	0.00000000
31	623.48890000	8.64680000	0.00000000
32	631.23750000	13.05180000	0.00000000
33	634.59830000	4.50790000	0.00000000
34	714.77000000	59.01210000	0.00000000
35	725.26030000	6.49320000	0.00000000
36	750.26500000	17.99240000	0.00000000
37	763.28510000	9.36350000	0.00000000
38	764.11990000	71.85440000	0.00000000
39	776.77240000	44.85990000	0.00000000
40	782.02620000	24.54270000	0.00000000
41	794.28800000	8.14810000	0.00000000
42	817.68830000	3.39900000	0.00000000
43	847.30740000	72.26890000	0.00000000
44	859.47830000	0.41060000	0.00000000
45	865.43110000	1.29270000	0.00000000
46	880.93020000	2.58490000	0.00000000
47	925.11860000	0.05430000	0.00000000
48	927.28400000	0.14430000	0.00000000
49	929.49100000	3.18910000	0.00000000
50	946.89160000	0.75710000	0.00000000
51	970.18890000	0.19560000	0.00000000
52	976.22820000	7.33000000	0.00000000
53	986.59260000	0.02210000	0.00000000
54	992.33970000	0.19910000	0.00000000
55	1007.27230000	0.56330000	0.00000000
56	1015.43760000	0.42820000	0.00000000
57	1020.06850000	6.04440000	0.00000000
58	1029.09850000	193.21520000	0.00000000
59	1047.70280000	10.07030000	0.00000000

60	1050.17530000	8.87700000	0.00000000
61	1050.32070000	7.01280000	0.00000000
62	1058.09870000	23.64610000	0.00000000
63	1100.94390000	53.33340000	0.00000000
64	1109.35690000	4.04850000	0.00000000
65	1152.66880000	396.37440000	0.00000000
66	1173.32000000	52.84700000	0.00000000
67	1175.43430000	4.10870000	0.00000000
68	1177.72550000	0.96960000	0.00000000
69	1184.09970000	282.38960000	0.00000000
70	1193.76750000	49.94400000	0.00000000
71	1200.51710000	6.77370000	0.00000000
72	1225.74000000	8.45580000	0.00000000
73	1241.00660000	11.87960000	0.00000000
74	1264.74360000	27.82440000	0.00000000
75	1270.74480000	108.32220000	0.00000000
76	1282.72050000	40.53410000	0.00000000
77	1299.20110000	195.87640000	0.00000000
78	1311.93430000	37.29170000	0.00000000
79	1331.85570000	27.61950000	0.00000000
80	1348.01880000	150.50500000	0.00000000
81	1351.55660000	3.82910000	0.00000000
82	1386.10190000	247.00300000	0.00000000
83	1394.92660000	27.89130000	0.00000000
84	1398.35950000	41.78160000	0.00000000
85	1408.32190000	500.69750000	0.00000000
86	1421.17300000	23.46460000	0.00000000
87	1466.87330000	0.40730000	0.00000000
88	1479.03150000	24.28520000	0.00000000
89	1484.21720000	2.24660000	0.00000000
90	1485.44260000	0.42420000	0.00000000
91	1485.73180000	12.52780000	0.00000000
92	1488.79550000	25.98610000	0.00000000
93	1492.47900000	32.24850000	0.00000000
94	1497.95580000	2.01910000	0.00000000
95	1511.60610000	198.39750000	0.00000000
96	1518.22620000	50.87130000	0.00000000
97	1526.53180000	15.43750000	0.00000000
98	1624.79060000	0.70440000	0.00000000
99	1631.75810000	2.82580000	0.00000000
100	1639.92420000	38.37520000	0.00000000
101	1643.23020000	5.85700000	0.00000000
102	1729.75000000	582.58110000	0.00000000

103	3035.11600000	37.31960000	0.00000000
104	3035.66230000	13.11940000	0.00000000
105	3037.07330000	36.12680000	0.00000000
106	3044.01920000	23.33580000	0.00000000
107	3091.54580000	16.81890000	0.00000000
108	3097.91740000	12.94660000	0.00000000
109	3100.20340000	23.67020000	0.00000000
110	3106.89010000	20.48250000	0.00000000
111	3108.63260000	53.45150000	0.00000000
112	3112.90360000	62.41890000	0.00000000
113	3138.84490000	4.34560000	0.00000000
114	3142.49550000	24.47790000	0.00000000
115	3156.29960000	9.43300000	0.00000000
116	3163.49940000	1.46400000	0.00000000
117	3164.73590000	0.44540000	0.00000000
118	3171.57500000	27.28230000	0.00000000
119	3173.66030000	26.30980000	0.00000000
120	3182.83720000	39.30530000	0.00000000
121	3187.45000000	38.83080000	0.00000000
122	3190.45880000	17.62930000	0.00000000
123	3241.22340000	1.37250000	0.00000000

N-Boc-2-phenylindoline 2a-2

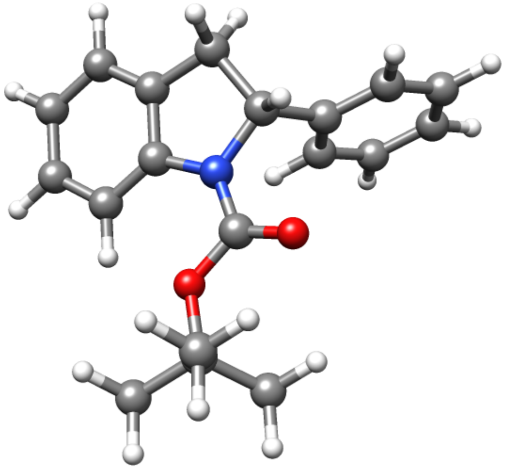

Route : # opt freq b3lyp/6-311g(d,p) scrf=(solvent=thf) geom=connectivity int=ultrafine

SMILES : CC(C)(C)OC(=O)N2c1ccccc1CC2c3ccccc3

Formula : $C_{19}H_{21}NO_2$

Charge : 0

Multiplicity : 1

Dipole : 1.5407 Debye

Energy : -942.15495272 a.u.

Gibbs Energy : -941.84666100 a.u.

Number of imaginary frequencies : 0

Cartesian Coordinates (XYZ format)

43

C	0.14083700	4.33697500	0.49617800
C	1.18592300	3.54947400	0.97474100
C	-0.85986100	3.75996000	-0.29190500
C	-0.79631400	2.40720000	-0.58869700
C	0.25836200	1.62149300	-0.10357600
C	1.26042700	2.18356000	0.68709500
C	-1.72513300	1.56837300	-1.43040500
C	-1.25479600	0.10749200	-1.16487600
H	-1.62080500	1.81836700	-2.49083200
H	-2.77471400	1.70148200	-1.16428600
N	0.07419100	0.28113100	-0.52292100
H	0.10169800	5.39319400	0.73565100
H	-1.67818800	4.36360600	-0.66962100
H	1.95917400	3.99720500	1.58906800
H	2.06982500	1.58183000	1.06783600
C	0.94864400	-0.78171300	-0.53590800
O	0.65660300	-1.85385000	-1.03484200

O	2.11756300	-0.47883400	0.05258000
C	3.22281600	-1.46729900	0.13185500
C	2.78259400	-2.67398000	0.96256000
H	2.41751200	-2.34806700	1.93975000
H	3.63920200	-3.33382900	1.12224600
H	1.99656400	-3.23418200	0.45959100
C	3.67704400	-1.85521700	-1.27650100
H	4.57217900	-2.47850100	-1.20744100
H	3.92874600	-0.96141200	-1.85293900
H	2.90287800	-2.41069900	-1.80257300
C	4.31660600	-0.68163300	0.85469300
H	3.97941100	-0.37247300	1.84656900
H	4.59235600	0.20879500	0.28559700
H	5.20446000	-1.30688800	0.97139000
H	-1.11225100	-0.43240100	-2.09977400
C	-2.22621000	-0.68200000	-0.30084300
C	-2.24755700	-0.55509700	1.09160500
C	-3.16163500	-1.51984500	-0.91467600
C	-3.18847700	-1.24796800	1.85121800
C	-4.10500700	-2.21167400	-0.15727500
C	-4.12173500	-2.07678900	1.22990000
H	-3.14670500	-1.63819100	-1.99348100
H	-4.81942000	-2.86203400	-0.64967900
H	-4.85098000	-2.61773400	1.82227300
H	-1.52227500	0.07950000	1.58766900
H	-3.19050000	-1.14186900	2.93042800

Frequencies

Mode	IR frequency	IR intensity	Raman intensity
1	16.77550000	0.08110000	0.00000000
2	25.82590000	0.33710000	0.00000000
3	32.64770000	0.00110000	0.00000000
4	35.96980000	0.81770000	0.00000000
5	77.27900000	1.97130000	0.00000000
6	91.42810000	0.79130000	0.00000000
7	96.16910000	0.65170000	0.00000000
8	128.51460000	0.73130000	0.00000000
9	173.10410000	1.44770000	0.00000000
10	208.92750000	1.38040000	0.00000000
11	210.04110000	2.17960000	0.00000000
12	218.96820000	5.69120000	0.00000000
13	236.37990000	2.78770000	0.00000000
14	252.30180000	0.61430000	0.00000000
15	273.11680000	0.40170000	0.00000000
16	274.47290000	0.10580000	0.00000000
17	294.38460000	3.36220000	0.00000000
18	326.81490000	12.81970000	0.00000000
19	342.44480000	8.03130000	0.00000000

20	351.69160000	4.05870000	0.00000000
21	406.39740000	6.88760000	0.00000000
22	413.62330000	0.47090000	0.00000000
23	423.69810000	2.01880000	0.00000000
24	432.27920000	5.83690000	0.00000000
25	456.15460000	4.19300000	0.00000000
26	470.61840000	6.78930000	0.00000000
27	486.87880000	8.39600000	0.00000000
28	542.45920000	2.65450000	0.00000000
29	561.39470000	6.27650000	0.00000000
30	594.54540000	15.16510000	0.00000000
31	617.43970000	8.15130000	0.00000000
32	633.31410000	0.87510000	0.00000000
33	661.43150000	24.50790000	0.00000000
34	714.01980000	58.45340000	0.00000000
35	722.27850000	2.24300000	0.00000000
36	729.07730000	12.91620000	0.00000000
37	760.91390000	89.72830000	0.00000000
38	765.56670000	11.31830000	0.00000000
39	779.00280000	28.79050000	0.00000000
40	780.92960000	22.58160000	0.00000000
41	795.33850000	0.71450000	0.00000000
42	821.36040000	3.23300000	0.00000000
43	844.83410000	13.83040000	0.00000000
44	858.01250000	0.84650000	0.00000000
45	867.52450000	67.29690000	0.00000000
46	875.01130000	12.12050000	0.00000000
47	925.81650000	0.04670000	0.00000000
48	927.83970000	0.17080000	0.00000000
49	928.68060000	4.09720000	0.00000000
50	943.76410000	0.35800000	0.00000000
51	970.73210000	0.14980000	0.00000000
52	976.83590000	11.69840000	0.00000000
53	984.97270000	0.02790000	0.00000000
54	987.98840000	0.01460000	0.00000000
55	1006.72310000	1.53350000	0.00000000
56	1015.74000000	0.47330000	0.00000000
57	1021.07250000	44.45860000	0.00000000
58	1035.15870000	64.42710000	0.00000000
59	1049.42200000	14.69060000	0.00000000
60	1050.51850000	0.59180000	0.00000000
61	1051.72760000	5.57690000	0.00000000
62	1062.37800000	34.72830000	0.00000000

63	1097.09470000	48.96800000	0.00000000
64	1109.08620000	4.93860000	0.00000000
65	1140.75500000	223.52890000	0.00000000
66	1174.77660000	30.64450000	0.00000000
67	1176.94720000	70.81160000	0.00000000
68	1177.59250000	3.66770000	0.00000000
69	1183.52360000	300.24270000	0.00000000
70	1192.41570000	44.86290000	0.00000000
71	1199.89850000	3.53260000	0.00000000
72	1226.18490000	3.90350000	0.00000000
73	1240.70060000	40.95030000	0.00000000
74	1265.24950000	24.52060000	0.00000000
75	1274.29530000	95.78020000	0.00000000
76	1287.60380000	85.96600000	0.00000000
77	1297.09720000	173.08510000	0.00000000
78	1312.06380000	16.14710000	0.00000000
79	1338.75450000	36.70550000	0.00000000
80	1351.16640000	29.57770000	0.00000000
81	1355.86540000	221.76510000	0.00000000
82	1384.24810000	404.81190000	0.00000000
83	1395.22880000	28.61110000	0.00000000
84	1398.25130000	68.44910000	0.00000000
85	1404.80590000	127.45270000	0.00000000
86	1420.83790000	21.50520000	0.00000000
87	1467.37260000	0.31260000	0.00000000
88	1478.49380000	31.17940000	0.00000000
89	1485.29100000	2.34000000	0.00000000
90	1486.20680000	0.87530000	0.00000000
91	1486.66240000	2.72280000	0.00000000
92	1488.61610000	54.48850000	0.00000000
93	1493.22110000	30.77500000	0.00000000
94	1498.64640000	2.14790000	0.00000000
95	1510.83550000	193.40360000	0.00000000
96	1518.46710000	57.32830000	0.00000000
97	1526.46800000	15.55160000	0.00000000
98	1624.96710000	1.82610000	0.00000000
99	1630.31580000	13.04580000	0.00000000
100	1641.58070000	66.92820000	0.00000000
101	1643.54540000	3.11680000	0.00000000
102	1721.67290000	743.86110000	0.00000000
103	3034.77560000	14.48610000	0.00000000
104	3035.23710000	36.05830000	0.00000000
105	3036.78050000	36.02210000	0.00000000

106	3043.75420000	20.18020000	0.00000000
107	3090.68910000	16.27040000	0.00000000
108	3097.90840000	8.26650000	0.00000000
109	3099.25480000	26.23100000	0.00000000
110	3102.04750000	22.85060000	0.00000000
111	3107.51990000	47.84260000	0.00000000
112	3112.12470000	69.91190000	0.00000000
113	3139.68930000	1.14900000	0.00000000
114	3142.75210000	25.38310000	0.00000000
115	3156.49480000	9.15230000	0.00000000
116	3163.79890000	1.68120000	0.00000000
117	3163.96300000	0.43490000	0.00000000
118	3171.63770000	26.24320000	0.00000000
119	3173.09020000	25.50790000	0.00000000
120	3181.76850000	42.80450000	0.00000000
121	3188.05790000	37.88920000	0.00000000
122	3189.71580000	19.00220000	0.00000000
123	3248.51350000	1.92800000	0.00000000

N-Boc-2-phenylindoline Clockwise TS from 2a-1 to 2a-2

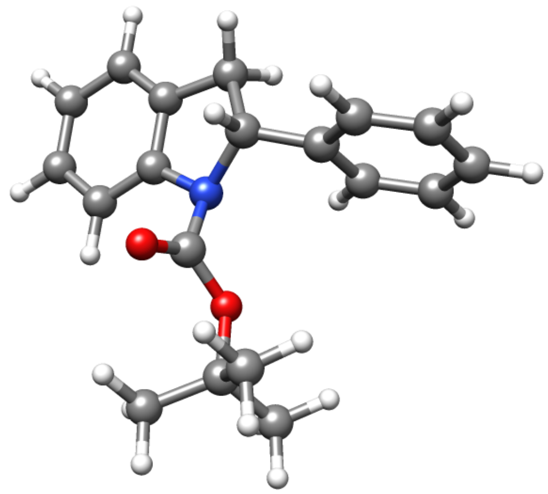

Route : # opt=qst3 freq b3lyp/6-311g(d,p) scrf=(solvent=thf) geom=connectivity int=ultrafine

SMILES : CC(C)(C)OC(=O)N2c1ccccc1CC2c3ccccc3

Formula : $C_{19}H_{21}NO_2$

Charge : 0

Multiplicity : 1

Dipole : 2.5506 Debye

Energy : -942.13549651 a.u.

Gibbs Energy : -941.82586300 a.u.

Number of imaginary frequencies : 1

Cartesian Coordinates (XYZ format)

43

C	4.86187400	0.39862000	0.31271100
C	4.31778200	-0.87716000	0.45450900
C	4.02450600	1.49573700	0.07423100
C	2.65700800	1.29529000	-0.02818300
C	2.12063100	0.00974700	0.12056300
C	2.93896300	-1.09030000	0.36214600
C	1.52379700	2.27087700	-0.23033200
C	0.33909200	1.34407100	-0.61424800
H	1.70902000	3.01760800	-1.00403100
H	1.30065200	2.80361900	0.70068900
N	0.71027800	0.04756700	0.03580900
H	5.93379100	0.54123900	0.38379700
H	4.44357300	2.49013000	-0.03799300
H	4.97118400	-1.72346500	0.63501900
H	2.52770600	-2.08663600	0.47381000
C	0.04051100	-1.10630500	-0.50149100
O	0.21885100	-1.52026300	-1.62190900

O	-0.78605100	-1.60674800	0.40810000
C	-1.65858400	-2.78525500	0.12086100
C	-2.61586900	-2.44852700	-1.02244300
H	-3.17263600	-1.53601800	-0.79706900
H	-3.33283700	-3.26482900	-1.13947000
H	-2.08346800	-2.31769000	-1.96322500
C	-0.78921700	-4.00700400	-0.17647200
H	-1.42921800	-4.88880200	-0.26009800
H	-0.08099400	-4.17846300	0.63785100
H	-0.23912800	-3.88809900	-1.10861200
C	-2.41303800	-2.95517500	1.43766400
H	-2.99152300	-2.05818900	1.66761400
H	-1.71687600	-3.14079100	2.25828800
H	-3.09832100	-3.80249100	1.36492600
H	0.35192400	1.19823500	-1.70233100
C	-1.02589400	1.85218200	-0.20388800
C	-1.43927000	1.82657200	1.13315200
C	-1.88049600	2.40043300	-1.16386600
C	-2.67819100	2.34507700	1.49951300
C	-3.12044600	2.92479500	-0.79812100
C	-3.52214300	2.89857000	0.53525900
H	-1.57489100	2.41594400	-2.20512700
H	-3.77177900	3.34675100	-1.55529000
H	-4.48707100	3.30108500	0.82219700
H	-0.79168100	1.38479600	1.88114500
H	-2.98786300	2.31570600	2.53833100

Frequencies

Mode	IR frequency	IR intensity	Raman intensity
1	-42.15670000	1.59340000	0.00000000
2	24.66790000	0.09230000	0.00000000
3	31.16470000	0.25460000	0.00000000
4	48.26230000	0.15890000	0.00000000
5	57.61810000	0.86980000	0.00000000
6	76.92370000	2.00190000	0.00000000
7	105.18100000	0.26370000	0.00000000
8	147.55750000	3.39970000	0.00000000
9	161.11100000	2.82660000	0.00000000
10	187.85650000	0.12900000	0.00000000
11	202.47410000	2.81600000	0.00000000
12	204.87140000	0.16430000	0.00000000
13	226.60040000	5.91770000	0.00000000
14	250.13730000	0.30680000	0.00000000
15	268.37970000	0.03510000	0.00000000
16	277.36280000	3.65470000	0.00000000
17	286.94460000	0.51270000	0.00000000
18	305.73590000	2.15070000	0.00000000
19	320.52140000	8.25090000	0.00000000

20	351.35710000	3.04490000	0.00000000
21	416.10050000	0.80030000	0.00000000
22	417.90840000	5.16220000	0.00000000
23	425.67100000	3.69170000	0.00000000
24	438.72080000	1.76190000	0.00000000
25	465.02460000	5.69070000	0.00000000
26	471.08010000	7.13000000	0.00000000
27	526.21970000	8.41570000	0.00000000
28	548.21870000	9.66590000	0.00000000
29	557.61890000	19.13830000	0.00000000
30	571.66510000	15.70370000	0.00000000
31	607.22320000	3.31430000	0.00000000
32	633.60170000	0.45950000	0.00000000
33	651.74220000	15.46160000	0.00000000
34	708.68840000	5.29180000	0.00000000
35	716.56780000	65.77910000	0.00000000
36	740.61190000	43.55610000	0.00000000
37	746.18290000	31.88290000	0.00000000
38	754.79910000	44.92500000	0.00000000
39	773.90200000	45.14040000	0.00000000
40	782.32210000	12.88360000	0.00000000
41	823.86670000	8.69320000	0.00000000
42	834.31720000	3.28730000	0.00000000
43	848.24050000	59.65090000	0.00000000
44	858.71420000	3.06020000	0.00000000
45	862.13140000	0.52260000	0.00000000
46	880.74480000	5.49440000	0.00000000
47	928.15440000	0.04150000	0.00000000
48	929.75220000	0.28540000	0.00000000
49	931.79430000	6.48900000	0.00000000
50	934.98830000	3.29870000	0.00000000
51	961.13590000	28.63120000	0.00000000
52	972.46560000	0.12900000	0.00000000
53	979.03930000	0.38030000	0.00000000
54	989.46060000	0.22320000	0.00000000
55	1004.83570000	25.39660000	0.00000000
56	1010.53850000	8.76660000	0.00000000
57	1015.93760000	1.37550000	0.00000000
58	1037.27690000	15.62810000	0.00000000
59	1045.68150000	29.22220000	0.00000000
60	1050.50360000	6.08620000	0.00000000
61	1051.29560000	5.00280000	0.00000000
62	1054.13490000	19.95000000	0.00000000

63	1072.31310000	40.12630000	0.00000000
64	1099.90720000	6.27560000	0.00000000
65	1136.22360000	33.08310000	0.00000000
66	1164.56630000	823.59230000	0.00000000
67	1175.71650000	5.49010000	0.00000000
68	1176.88600000	5.97690000	0.00000000
69	1184.94680000	33.72220000	0.00000000
70	1194.34330000	4.18060000	0.00000000
71	1200.21930000	0.90500000	0.00000000
72	1226.24000000	8.23410000	0.00000000
73	1258.76970000	205.93110000	0.00000000
74	1264.49900000	238.33550000	0.00000000
75	1267.98010000	50.46430000	0.00000000
76	1273.93780000	298.73140000	0.00000000
77	1282.82610000	97.28050000	0.00000000
78	1306.70060000	54.59190000	0.00000000
79	1328.12720000	6.75710000	0.00000000
80	1343.27710000	12.35200000	0.00000000
81	1347.67750000	12.91730000	0.00000000
82	1368.72470000	52.13370000	0.00000000
83	1397.98240000	27.41340000	0.00000000
84	1399.70820000	38.78710000	0.00000000
85	1401.02220000	15.48280000	0.00000000
86	1423.17530000	19.65570000	0.00000000
87	1468.05110000	0.40520000	0.00000000
88	1477.05380000	8.07570000	0.00000000
89	1485.02980000	1.33800000	0.00000000
90	1485.17080000	0.56830000	0.00000000
91	1485.78140000	17.12060000	0.00000000
92	1490.37400000	27.95990000	0.00000000
93	1491.57620000	17.08220000	0.00000000
94	1497.61530000	2.56190000	0.00000000
95	1510.92910000	131.71000000	0.00000000
96	1517.60790000	23.02320000	0.00000000
97	1525.47000000	11.65500000	0.00000000
98	1624.57140000	1.20260000	0.00000000
99	1630.19250000	5.36200000	0.00000000
100	1642.73790000	57.76230000	0.00000000
101	1644.02470000	2.50000000	0.00000000
102	1762.16960000	407.22640000	0.00000000
103	2995.32460000	40.66450000	0.00000000
104	3026.83440000	38.29280000	0.00000000
105	3037.55950000	13.42360000	0.00000000

106	3039.74780000	31.41090000	0.00000000
107	3046.09800000	16.63730000	0.00000000
108	3093.61780000	23.86840000	0.00000000
109	3101.56860000	12.62320000	0.00000000
110	3103.86280000	17.05220000	0.00000000
111	3111.36230000	43.10310000	0.00000000
112	3114.62870000	57.66070000	0.00000000
113	3136.28160000	0.99980000	0.00000000
114	3139.05660000	28.96470000	0.00000000
115	3157.73980000	8.08880000	0.00000000
116	3162.10640000	4.80590000	0.00000000
117	3165.00850000	0.67300000	0.00000000
118	3168.95470000	15.66470000	0.00000000
119	3174.60450000	30.24930000	0.00000000
120	3181.58980000	38.54220000	0.00000000
121	3185.00300000	40.75210000	0.00000000
122	3190.36440000	25.79710000	0.00000000
123	3191.76100000	11.47750000	0.00000000

N-Boc-2-phenylindoline Anticlockwise TS from 2a-1 to 2a-2

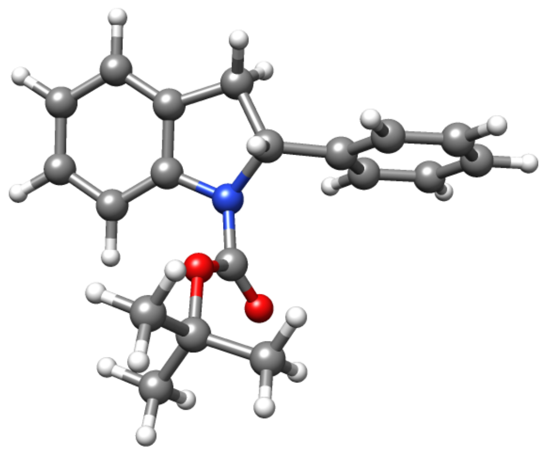

Route : # opt=qst3 freq b3lyp/6-311g(d,p) scrf=(solvent=thf) geom=connectivity int=ultrafine

SMILES : CC(C)(C)OC(=O)N2c1ccccc1CC2c3ccccc3

Formula : $C_{19}H_{21}NO_2$

Charge : 0

Multiplicity : 1

Dipole : 3.3412 Debye

Energy : -942.13520116 a.u.

Gibbs Energy : -941.82540300 a.u.

Number of imaginary frequencies : 1

Cartesian Coordinates (XYZ format)

43

C	-3.22795400	3.31888500	0.15551500
C	-3.53494400	2.21843700	-0.64354000
C	-1.95314500	3.43954700	0.72319500
C	-1.01126400	2.44959200	0.49204100
C	-1.33102600	1.34779600	-0.31208200
C	-2.59038400	1.21755900	-0.89123000
C	0.43281700	2.33600000	0.91566900
C	0.76092000	0.84500900	0.62750400
H	0.61338300	2.58570100	1.96231800
H	1.06713500	2.98648300	0.30334500
N	-0.21150300	0.49957400	-0.45495500
H	-3.97605200	4.08143000	0.33785900
H	-1.70886200	4.29584300	1.34297100
H	-4.52320100	2.13041100	-1.08098500
H	-2.83772800	0.37040200	-1.52009800
C	-0.41615300	-0.89756100	-0.70856400
O	-0.02418100	-1.44328500	-1.70846200
O	-1.06587200	-1.47045900	0.30574400

C	-1.40626800	-2.92534400	0.32292100
C	-2.33812500	-3.24702700	-0.84510000
H	-3.21742900	-2.59884600	-0.82157800
H	-2.67725500	-4.28211900	-0.75693800
H	-1.83304100	-3.12864500	-1.80247800
C	-0.11941900	-3.74942200	0.29587400
H	-0.36745000	-4.80524400	0.42974800
H	0.54300300	-3.44986500	1.11157200
H	0.40746700	-3.63376700	-0.65001400
C	-2.12905400	-3.07355200	1.65956500
H	-3.01415800	-2.43481200	1.69080200
H	-1.47109400	-2.79798600	2.48647000
H	-2.44327900	-4.11041800	1.79729400
H	0.51046600	0.25771400	1.52024000
C	2.20272300	0.57477200	0.25476000
C	2.69113500	0.85106200	-1.02713800
C	3.08453900	0.08404400	1.22189400
C	4.03475900	0.64651400	-1.32972600
C	4.43167600	-0.11605200	0.92199800
C	4.91037300	0.16535500	-0.35564100
H	2.71442100	-0.14419200	2.21643600
H	5.10269500	-0.49743600	1.68352000
H	5.95583500	0.00517500	-0.59385900
H	2.00981000	1.21036800	-1.78896200
H	4.39904300	0.85905500	-2.32871500

Frequencies

Mode	IR frequency	IR intensity	Raman intensity
1	-38.14950000	1.55890000	0.00000000
2	26.72490000	0.30910000	0.00000000
3	34.01550000	0.19610000	0.00000000
4	49.06650000	0.50070000	0.00000000
5	58.00680000	1.34570000	0.00000000
6	76.41380000	0.90620000	0.00000000
7	100.27490000	1.22100000	0.00000000
8	149.52230000	2.61630000	0.00000000
9	158.57160000	2.22730000	0.00000000
10	186.52070000	0.56450000	0.00000000
11	198.63500000	3.49600000	0.00000000
12	207.31010000	0.13470000	0.00000000
13	235.24520000	4.20730000	0.00000000
14	253.98570000	1.00260000	0.00000000
15	265.89760000	3.21600000	0.00000000
16	267.79020000	1.26410000	0.00000000
17	289.28970000	1.03220000	0.00000000
18	320.52070000	8.33920000	0.00000000
19	351.45710000	2.63210000	0.00000000
20	369.55890000	5.95920000	0.00000000

21	401.67070000	1.76180000	0.00000000
22	415.88770000	0.58860000	0.00000000
23	425.75890000	7.14800000	0.00000000
24	437.58130000	2.03990000	0.00000000
25	462.81300000	4.43830000	0.00000000
26	470.10690000	7.27300000	0.00000000
27	512.08750000	14.56450000	0.00000000
28	544.90880000	2.68140000	0.00000000
29	555.94500000	16.03930000	0.00000000
30	573.11330000	8.06930000	0.00000000
31	616.67620000	10.25790000	0.00000000
32	633.09790000	0.60640000	0.00000000
33	639.80560000	9.24510000	0.00000000
34	690.84950000	1.68030000	0.00000000
35	714.97300000	58.35650000	0.00000000
36	732.54090000	5.47770000	0.00000000
37	754.61040000	82.06370000	0.00000000
38	761.25880000	10.56760000	0.00000000
39	774.66240000	48.43930000	0.00000000
40	783.11730000	15.26950000	0.00000000
41	829.50030000	10.43630000	0.00000000
42	833.12570000	7.80800000	0.00000000
43	844.51200000	84.17800000	0.00000000
44	860.76260000	0.57780000	0.00000000
45	862.99910000	4.23370000	0.00000000
46	879.69550000	10.34910000	0.00000000
47	928.17460000	0.05650000	0.00000000
48	929.62720000	0.43070000	0.00000000
49	931.79230000	5.40010000	0.00000000
50	935.73200000	4.57790000	0.00000000
51	961.36200000	24.64170000	0.00000000
52	971.94860000	0.13290000	0.00000000
53	979.62250000	0.15620000	0.00000000
54	988.47090000	0.24070000	0.00000000
55	1004.01590000	37.66170000	0.00000000
56	1010.35580000	8.10200000	0.00000000
57	1016.02740000	1.02610000	0.00000000
58	1037.04990000	14.09000000	0.00000000
59	1046.25600000	25.76510000	0.00000000
60	1049.95610000	12.86000000	0.00000000
61	1050.89060000	1.79240000	0.00000000
62	1054.45920000	15.29590000	0.00000000
63	1072.56840000	48.85380000	0.00000000

64	1100.03370000	11.48560000	0.00000000
65	1133.08520000	95.19730000	0.00000000
66	1154.91460000	749.25140000	0.00000000
67	1175.23120000	4.71330000	0.00000000
68	1176.99600000	2.49480000	0.00000000
69	1183.50820000	10.43050000	0.00000000
70	1194.89790000	2.76310000	0.00000000
71	1199.19060000	3.19880000	0.00000000
72	1225.70760000	10.82290000	0.00000000
73	1239.97670000	203.20830000	0.00000000
74	1262.12310000	30.01040000	0.00000000
75	1267.90610000	28.75000000	0.00000000
76	1277.85830000	105.62890000	0.00000000
77	1286.59640000	237.70620000	0.00000000
78	1307.58480000	37.50880000	0.00000000
79	1328.59430000	7.13380000	0.00000000
80	1343.25920000	7.33410000	0.00000000
81	1348.51360000	6.38890000	0.00000000
82	1368.96300000	57.30560000	0.00000000
83	1397.94820000	25.26850000	0.00000000
84	1398.69380000	15.26250000	0.00000000
85	1400.92870000	32.09140000	0.00000000
86	1423.32350000	18.74420000	0.00000000
87	1468.67570000	0.57350000	0.00000000
88	1476.87470000	9.24630000	0.00000000
89	1484.44430000	0.37710000	0.00000000
90	1485.03020000	1.72210000	0.00000000
91	1485.61530000	19.43650000	0.00000000
92	1490.49860000	57.72010000	0.00000000
93	1492.02320000	5.12670000	0.00000000
94	1497.52730000	2.55690000	0.00000000
95	1510.50540000	155.93890000	0.00000000
96	1517.62120000	21.76650000	0.00000000
97	1525.45900000	12.44140000	0.00000000
98	1624.40730000	0.75880000	0.00000000
99	1629.96750000	12.22980000	0.00000000
100	1642.60080000	81.37590000	0.00000000
101	1644.18440000	0.81570000	0.00000000
102	1779.20080000	524.53010000	0.00000000
103	2998.57640000	38.47850000	0.00000000
104	3026.55020000	39.66960000	0.00000000
105	3037.66790000	13.31670000	0.00000000
106	3039.60390000	32.08130000	0.00000000

107	3045.73080000	15.31470000	0.00000000
108	3094.19700000	23.15450000	0.00000000
109	3101.79330000	8.30940000	0.00000000
110	3103.30340000	21.70860000	0.00000000
111	3110.65610000	44.01560000	0.00000000
112	3114.13590000	58.70610000	0.00000000
113	3136.32700000	1.14700000	0.00000000
114	3139.17140000	26.58080000	0.00000000
115	3156.56070000	9.29280000	0.00000000
116	3162.05130000	5.20170000	0.00000000
117	3164.95290000	0.55250000	0.00000000
118	3168.77200000	14.68660000	0.00000000
119	3174.39580000	30.32470000	0.00000000
120	3181.05010000	37.21340000	0.00000000
121	3184.71920000	38.77220000	0.00000000
122	3190.18350000	27.13520000	0.00000000
123	3191.47670000	12.63880000	0.00000000

Thermochemistry Data for *N*-Boc-2-phenylindoline Calculations

N-Boc-2-phenylindoline Rotamer **2a-1**:

	Temperature		
	298 K	223 K	195 K
ε_0	–942.156610937 a.u.	–942.156610937 a.u.	–942.156610937 a.u.
ε_{ZPE}	0.358629 a.u.	0.358629 a.u.	0.358629 a.u.
E_{tot}	0.378537 a.u.	0.370315 a.u.	0.367831 a.u.
H_{corr}	0.379481 a.u.	0.371021 a.u.	0.368449 a.u.
G_{corr}	0.308843 a.u.	0.325431 a.u.	0.330983 a.u.
S_{tot}	148.670 calmol^{-1}K^{-1}	128.290 calmol^{-1}K^{-1}	120.564 calmol^{-1}K^{-1}
$\varepsilon_0 + \varepsilon_{ZPE}$	–941.797982 a.u.	–941.797982 a.u.	–941.797982 a.u.
$\varepsilon_0 + E_{tot}$	–941.778074 a.u.	–941.786296 a.u.	–941.788780 a.u.
$\varepsilon_0 + H_{corr}$	–941.777130 a.u.	–941.785590 a.u.	–941.788162 a.u.
$\varepsilon_0 + G_{corr}$	–941.847768 a.u.	–941.831180 a.u.	–941.825628 a.u.

N-Boc-2-phenylindoline Rotamer **2a-2**:

	Temperature		
	298 K	223 K	195 K
ε_0	–942.154952721 a.u.	–942.154952721 a.u.	–942.154952721 a.u.
ε_{ZPE}	0.358600 a.u.	0.358600 a.u.	0.358600 a.u.
E_{tot}	0.378521 a.u.	0.370300 a.u.	0.367817 a.u.
H_{corr}	0.379466 a.u.	0.371006 a.u.	0.368434 a.u.
G_{corr}	0.308292 a.u.	0.325014 a.u.	0.330617 a.u.
S_{tot}	149.798 calmol^{-1}K^{-1}	129.419 calmol^{-1}K^{-1}	121.695 calmol^{-1}K^{-1}
$\varepsilon_0 + \varepsilon_{ZPE}$	–941.796353 a.u.	–941.796353 a.u.	–941.796353 a.u.
$\varepsilon_0 + E_{tot}$	–941.776431 a.u.	–941.784653 a.u.	–941.787136 a.u.
$\varepsilon_0 + H_{corr}$	–941.775487 a.u.	–941.783947 a.u.	–941.786519 a.u.
$\varepsilon_0 + G_{corr}$	–941.846661 a.u.	–941.829939 a.u.	–941.824336 a.u.

TS for *N*-Boc-2-phenylindoline Clockwise Rotation of Boc Group from Rotamer **2a-1** to Rotamer **2a-2**:

	Temperature	
	298 K	195 K
ε_0	–942.135496507 a.u.	–942.135496507 a.u.
ε_{ZPE}	0.357788 a.u.	0.357788 a.u.
E_{tot}	0.376942 a.u.	0.366528 a.u.
H_{corr}	0.377886 a.u.	0.367146 a.u.
G_{corr}	0.309633 a.u.	0.331006 a.u.
S_{tot}	143.650 calmol^{-1}K^{-1}	116.298 calmol^{-1}K^{-1}
$\varepsilon_0 + \varepsilon_{ZPE}$	–941.777708 a.u.	–941.777709 a.u.
$\varepsilon_0 + E_{tot}$	–941.758555 a.u.	–941.768969 a.u.
$\varepsilon_0 + H_{corr}$	–941.757610 a.u.	–941.768351 a.u.
$\varepsilon_0 + G_{corr}$	–941.825863 a.u.	–941.804491 a.u.

TS for *N*-Boc-2-phenylindoline Anticlockwise Rotation of Boc Group from Rotamer **2a-1** to Rotamer **2a-2**:

	Temperature	
	298 K	195 K
ε_0	–942.135201164 a.u.	–942.135201164 a.u.
ε_{ZPE}	0.357790 a.u.	0.357790 a.u.
E_{tot}	0.376907 a.u.	0.366498 a.u.
H_{corr}	0.377851 a.u.	0.367116 a.u.
G_{corr}	0.309798 a.u.	0.331102 a.u.
S_{tot}	143.230 calmol^{-1}K^{-1}	115.892 calmol^{-1}K^{-1}
$\varepsilon_0 + \varepsilon_{ZPE}$	–941.777411 a.u.	–941.777411 a.u.
$\varepsilon_0 + E_{tot}$	–941.758294 a.u.	–941.768703 a.u.
$\varepsilon_0 + H_{corr}$	–941.757350 a.u.	–941.768085 a.u.
$\varepsilon_0 + G_{corr}$	–941.825403 a.u.	–941.804099 a.u.

N-Boc-2-(*p*-methoxyphenyl)indoline 2d-1

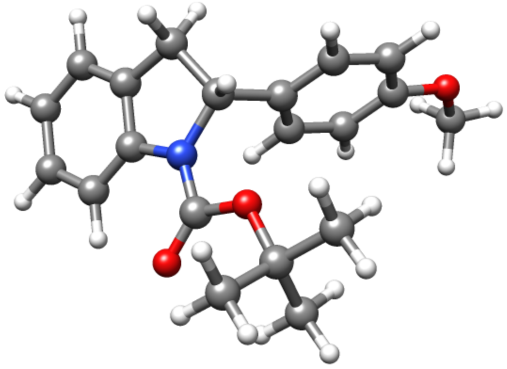

Route : # opt freq b3lyp/6-311g(d,p) scrf=(solvent=thf)
geom=connectivity int=ultrafine

SMILES : COc3ccc(C2Cc1ccccc1N2C(=O)OC(C)(C)C)cc3

Formula : $C_{20}H_{23}NO_3$

Charge : 0

Multiplicity : 1

Dipole : 2.9607 Debye

Energy : -1056.71131073 a.u.

Gibbs Energy : -1056.37383500 a.u.

Number of imaginary frequencies : 0

Cartesian Coordinates (XYZ format)

47

C	2.10046100	-2.07372400	0.77995000
C	2.25378900	-1.00150800	-0.10792600
C	1.09716800	-1.71719900	1.84823500
C	0.43374200	-0.40802200	1.32037300
H	1.60001300	-1.53374000	2.80320000
H	0.35298900	-2.49744800	2.01364300
N	1.37098400	0.04157300	0.25425600
H	0.40829500	0.34936200	2.10176200
C	-0.98155900	-0.62431100	0.80956600
C	2.84734800	-3.23014200	0.61572400
C	3.75964800	-3.31169200	-0.44167900
C	3.91063900	-2.23565400	-1.31463200
C	3.16151200	-1.06484300	-1.16464000
H	2.72765300	-4.06080800	1.30306000
H	4.34869500	-4.21077600	-0.58074000
H	4.61944300	-2.30249000	-2.13253500
H	3.27696300	-0.23179300	-1.84025000
C	1.39427900	1.31289000	-0.26719400
O	0.48773400	2.08713500	0.35911400
O	2.14012500	1.67125600	-1.16115000

C	0.26817500	3.49971300	-0.03159000
C	-0.84435400	3.93483300	0.92184700
H	-1.73504700	3.31954400	0.77959200
H	-1.10741200	4.97796800	0.73283700
H	-0.51922000	3.84234600	1.96048600
C	1.54107700	4.31274900	0.21287900
H	1.87471000	4.19269800	1.24673400
H	1.33124700	5.37212200	0.04427200
H	2.34191000	4.00301900	-0.45608400
C	-0.20467700	3.57222500	-1.48499900
H	-1.08222500	2.93736100	-1.62979800
H	0.57901200	3.25666400	-2.17152200
H	-0.48582500	4.60149800	-1.72205100
C	-1.24963100	-1.08951000	-0.47673400
C	-2.06554500	-0.40521400	1.66929900
C	-2.55549700	-1.33657800	-0.90531500
C	-3.62523600	-1.11853800	-0.03289600
C	-3.36900900	-0.64825900	1.26159100
H	-0.43536700	-1.25914500	-1.17172500
H	-2.72141500	-1.69315400	-1.91282100
O	-4.93675900	-1.32391700	-0.34430400
H	-1.88746100	-0.03292000	2.67320200
H	-4.20674000	-0.47265900	1.92611200
C	-5.25860600	-1.79622300	-1.65121200
H	-6.34289400	-1.88645300	-1.67494500
H	-4.80887600	-2.77543900	-1.84368900
H	-4.93649800	-1.08851000	-2.42169300

Frequencies

Mode	IR frequency	IR intensity	Raman intensity
1	16.93460000	0.94350000	0.00000000
2	23.24050000	0.60920000	0.00000000
3	30.32940000	0.03150000	0.00000000
4	37.91930000	0.71110000	0.00000000
5	57.94830000	2.78130000	0.00000000
6	81.78170000	2.63870000	0.00000000
7	94.12460000	0.88630000	0.00000000
8	104.49830000	2.98230000	0.00000000
9	126.54920000	0.46890000	0.00000000
10	144.08070000	0.76430000	0.00000000
11	174.73970000	1.48750000	0.00000000
12	206.40480000	0.09230000	0.00000000
13	212.31440000	1.10800000	0.00000000
14	221.21310000	7.35110000	0.00000000
15	231.21140000	1.55490000	0.00000000
16	243.96890000	3.29540000	0.00000000
17	251.95420000	1.05000000	0.00000000
18	264.48020000	4.42620000	0.00000000

19	271.12930000	0.06220000	0.00000000
20	289.61370000	2.05380000	0.00000000
21	322.28380000	0.08940000	0.00000000
22	331.81170000	20.26120000	0.00000000
23	351.12190000	2.82080000	0.00000000
24	378.83380000	0.64320000	0.00000000
25	407.97860000	4.51850000	0.00000000
26	425.09910000	0.49110000	0.00000000
27	433.74680000	3.82020000	0.00000000
28	440.28300000	1.36780000	0.00000000
29	457.34980000	4.86710000	0.00000000
30	469.37420000	2.89800000	0.00000000
31	477.20500000	4.43130000	0.00000000
32	512.38260000	2.10980000	0.00000000
33	536.67980000	18.61130000	0.00000000
34	559.16930000	5.32950000	0.00000000
35	568.89600000	25.91250000	0.00000000
36	618.69360000	9.49310000	0.00000000
37	629.06260000	16.46760000	0.00000000
38	648.60480000	1.64810000	0.00000000
39	715.36720000	1.99200000	0.00000000
40	741.44930000	11.49470000	0.00000000
41	749.97470000	15.72640000	0.00000000
42	750.68560000	13.93620000	0.00000000
43	763.93670000	22.66840000	0.00000000
44	767.57790000	72.71170000	0.00000000
45	785.94610000	18.98030000	0.00000000
46	815.85870000	7.47340000	0.00000000
47	827.47180000	2.23930000	0.00000000
48	829.17790000	13.09410000	0.00000000
49	845.81790000	16.01950000	0.00000000
50	848.58790000	128.84810000	0.00000000
51	865.73980000	0.40990000	0.00000000
52	881.39020000	3.04380000	0.00000000
53	925.67120000	0.05870000	0.00000000
54	927.86160000	0.13750000	0.00000000
55	946.81090000	0.51490000	0.00000000
56	953.83290000	0.06090000	0.00000000
57	970.67850000	0.19190000	0.00000000
58	971.63070000	0.02270000	0.00000000
59	977.34020000	9.45280000	0.00000000
60	992.30360000	0.22250000	0.00000000
61	1019.26370000	6.58190000	0.00000000

62	1024.54490000	35.84680000	0.00000000
63	1029.70990000	177.68580000	0.00000000
64	1048.01120000	13.45800000	0.00000000
65	1050.31920000	71.93820000	0.00000000
66	1051.00720000	0.36230000	0.00000000
67	1058.41380000	22.76670000	0.00000000
68	1105.74650000	24.42530000	0.00000000
69	1131.59570000	78.39680000	0.00000000
70	1151.88950000	341.76060000	0.00000000
71	1169.98210000	1.01170000	0.00000000
72	1173.81580000	50.07740000	0.00000000
73	1175.69270000	14.02910000	0.00000000
74	1183.67860000	260.39330000	0.00000000
75	1193.83420000	74.06340000	0.00000000
76	1196.02120000	78.55880000	0.00000000
77	1203.04770000	22.44840000	0.00000000
78	1225.07860000	20.66940000	0.00000000
79	1240.37380000	19.12980000	0.00000000
80	1264.78060000	32.65220000	0.00000000
81	1269.67440000	246.09990000	0.00000000
82	1270.81310000	202.51780000	0.00000000
83	1282.00490000	42.42420000	0.00000000
84	1299.72830000	201.87870000	0.00000000
85	1318.49440000	96.83320000	0.00000000
86	1332.06990000	33.80190000	0.00000000
87	1334.28070000	24.47380000	0.00000000
88	1348.47390000	125.97960000	0.00000000
89	1381.73730000	190.00650000	0.00000000
90	1395.23040000	28.20070000	0.00000000
91	1398.25460000	36.81940000	0.00000000
92	1407.15380000	576.80320000	0.00000000
93	1421.53680000	25.05520000	0.00000000
94	1452.40520000	10.41280000	0.00000000
95	1467.16930000	0.35170000	0.00000000
96	1476.81310000	18.16930000	0.00000000
97	1480.26610000	5.67100000	0.00000000
98	1484.73090000	2.35650000	0.00000000
99	1485.68590000	10.25420000	0.00000000
100	1485.71110000	1.52770000	0.00000000
101	1488.78060000	28.73820000	0.00000000
102	1492.33270000	35.40080000	0.00000000
103	1498.50700000	2.75670000	0.00000000
104	1499.58990000	50.89750000	0.00000000

105	1511.17240000	204.44180000	0.00000000
106	1518.74150000	48.09500000	0.00000000
107	1542.48670000	163.06470000	0.00000000
108	1616.45470000	30.23420000	0.00000000
109	1631.27880000	2.18580000	0.00000000
110	1639.89640000	37.19130000	0.00000000
111	1651.10980000	77.27020000	0.00000000
112	1728.56980000	577.81180000	0.00000000
113	3010.92790000	67.38420000	0.00000000
114	3033.05810000	38.73990000	0.00000000
115	3035.75410000	15.88940000	0.00000000
116	3036.76190000	33.38980000	0.00000000
117	3044.01550000	23.57010000	0.00000000
118	3071.98090000	50.00890000	0.00000000
119	3092.02220000	17.32620000	0.00000000
120	3097.47000000	13.22780000	0.00000000
121	3100.38180000	25.56890000	0.00000000
122	3104.80890000	24.67460000	0.00000000
123	3108.79330000	52.50970000	0.00000000
124	3113.31220000	61.40020000	0.00000000
125	3138.48860000	31.04360000	0.00000000
126	3138.54720000	1.42720000	0.00000000
127	3141.63770000	27.94210000	0.00000000
128	3158.76030000	18.51320000	0.00000000
129	3163.19710000	1.47720000	0.00000000
130	3171.30330000	27.30680000	0.00000000
131	3178.28470000	11.03770000	0.00000000
132	3187.22280000	43.22830000	0.00000000
133	3187.54550000	8.82500000	0.00000000
134	3207.09910000	8.71500000	0.00000000
135	3241.47480000	1.37600000	0.00000000

N-Boc-2-(*p*-methoxyphenyl)indoline 2d-2

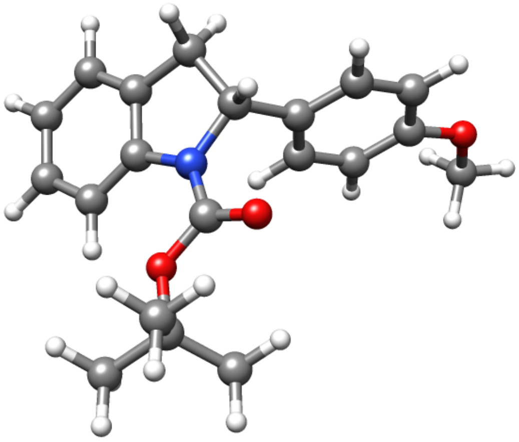

Route : # opt freq b3lyp/6-311g(d,p) scrf=(solvent=thf)
geom=connectivity int=ultrafine

SMILES : COc3ccc(C2Cc1ccccc1N2C(=O)OC(C)(C)C)cc3

Formula : $C_{20}H_{23}NO_3$

Charge : 0

Multiplicity : 1

Dipole : 2.7072 Debye

Energy : -1056.70957881 a.u.

Gibbs Energy : -1056.37243600 a.u.

Number of imaginary frequencies : 0

Cartesian Coordinates (XYZ format)

47

C	-0.65862200	2.67695900	0.52478800
C	-1.31416700	1.55819300	-0.00848400
C	0.29452800	2.24941200	1.61251700
C	0.35705800	0.69999200	1.46763900
H	-0.09319100	2.52810500	2.59762900
H	1.28423000	2.69672700	1.51081500
N	-0.82050700	0.38630400	0.61492700
H	0.21579800	0.21331900	2.43162800
C	1.66343200	0.20430800	0.86973300
C	-0.94659500	3.94986400	0.05704800
C	-1.90105300	4.11032000	-0.95237900
C	-2.54890700	2.99377600	-1.47671800
C	-2.26529600	1.70404800	-1.01814600
H	-0.43633300	4.81220700	0.47271300
H	-2.13421500	5.10033100	-1.32660800
H	-3.28627000	3.11901300	-2.26186400
H	-2.76754500	0.84617400	-1.43528100
C	-1.32080000	-0.89532200	0.63516400

O	-2.39582200	-1.02800300	-0.16034000
O	-0.81994100	-1.77642400	1.31072600
C	-3.11674200	-2.32032800	-0.27798000
C	-4.24407700	-1.98114500	-1.25289400
H	-4.87538400	-1.18580600	-0.85078500
H	-4.86456600	-2.86384800	-1.42212100
H	-3.83942100	-1.65453100	-2.21346700
C	-2.18744700	-3.38115300	-0.87048600
H	-1.76559700	-3.02986000	-1.81550800
H	-2.75890500	-4.29121200	-1.07027700
H	-1.37484000	-3.61993700	-0.18684500
C	-3.68389300	-2.72848800	1.08301800
H	-4.29341000	-1.92124200	1.49698400
H	-2.88887100	-2.96948600	1.78618500
H	-4.32215100	-3.60705600	0.95875500
C	1.90745900	0.21396400	-0.50274600
C	2.68969100	-0.23163700	1.71724100
C	3.13547900	-0.19266000	-1.02807700
C	4.15116800	-0.61838100	-0.16762900
C	3.91722900	-0.63573300	1.21329200
H	1.13075800	0.53321400	-1.18818300
H	3.28292400	-0.17528600	-2.09941200
O	5.38541300	-1.03747200	-0.56922700
H	2.52314400	-0.26254800	2.78932400
H	4.70755500	-0.97961400	1.87002300
C	5.67629100	-1.05310800	-1.96535600
H	6.69764100	-1.41905800	-2.05123700
H	5.61334300	-0.04944700	-2.39780300
H	5.00323800	-1.72637500	-2.50569500

Frequencies

Mode	IR frequency	IR intensity	Raman intensity
1	15.55580000	0.29720000	0.00000000
2	21.04050000	0.95360000	0.00000000
3	30.47820000	0.59870000	0.00000000
4	36.20940000	0.03060000	0.00000000
5	59.67640000	1.97890000	0.00000000
6	81.80120000	2.53730000	0.00000000
7	95.38850000	0.99320000	0.00000000
8	98.70530000	2.84860000	0.00000000
9	125.73370000	0.81960000	0.00000000
10	141.89070000	0.74510000	0.00000000
11	172.42030000	2.00960000	0.00000000
12	209.42610000	2.90550000	0.00000000
13	211.39390000	0.23440000	0.00000000
14	223.13360000	6.32090000	0.00000000
15	229.82990000	1.63940000	0.00000000
16	246.41890000	1.63100000	0.00000000
17	252.28140000	1.28090000	0.00000000

18	269.08860000	3.72390000	0.00000000
19	274.54000000	0.08020000	0.00000000
20	293.92450000	2.49640000	0.00000000
21	322.89870000	8.29940000	0.00000000
22	331.35300000	5.73230000	0.00000000
23	349.68710000	2.30560000	0.00000000
24	358.82240000	11.35420000	0.00000000
25	414.45930000	1.92460000	0.00000000
26	424.76740000	0.75010000	0.00000000
27	430.92620000	3.74050000	0.00000000
28	442.55220000	9.19050000	0.00000000
29	456.02210000	4.73210000	0.00000000
30	469.23680000	4.99420000	0.00000000
31	477.31070000	2.67920000	0.00000000
32	506.97080000	7.29910000	0.00000000
33	536.98860000	17.36520000	0.00000000
34	560.14530000	10.97370000	0.00000000
35	565.65260000	23.21820000	0.00000000
36	613.26510000	10.39000000	0.00000000
37	645.28410000	4.03930000	0.00000000
38	662.80450000	19.93720000	0.00000000
39	711.56590000	2.95660000	0.00000000
40	725.43440000	5.63210000	0.00000000
41	742.51870000	16.55010000	0.00000000
42	751.02350000	26.49980000	0.00000000
43	764.23970000	69.07900000	0.00000000
44	769.38500000	15.31790000	0.00000000
45	787.07850000	9.77240000	0.00000000
46	817.32540000	2.43730000	0.00000000
47	825.80440000	2.52860000	0.00000000
48	830.09550000	10.16000000	0.00000000
49	844.49280000	50.13580000	0.00000000
50	846.58490000	37.20490000	0.00000000
51	868.02790000	72.85360000	0.00000000
52	875.75790000	11.44500000	0.00000000
53	925.52990000	0.04410000	0.00000000
54	927.32980000	0.50750000	0.00000000
55	943.73120000	0.13740000	0.00000000
56	952.58950000	0.44820000	0.00000000
57	970.23570000	0.10660000	0.00000000
58	970.25500000	0.14030000	0.00000000
59	977.11250000	13.75560000	0.00000000
60	987.95790000	0.02630000	0.00000000

61	1020.50020000	48.49040000	0.00000000
62	1024.94930000	7.51150000	0.00000000
63	1035.56190000	63.89390000	0.00000000
64	1050.21780000	42.67810000	0.00000000
65	1050.61790000	7.24690000	0.00000000
66	1051.46880000	34.15970000	0.00000000
67	1062.07210000	43.13270000	0.00000000
68	1100.71090000	45.17230000	0.00000000
69	1131.53200000	51.54990000	0.00000000
70	1141.14160000	191.81500000	0.00000000
71	1169.32220000	0.92830000	0.00000000
72	1175.76390000	58.35850000	0.00000000
73	1176.64070000	55.10000000	0.00000000
74	1182.92520000	293.29140000	0.00000000
75	1192.15410000	57.03880000	0.00000000
76	1195.82850000	59.64890000	0.00000000
77	1202.37850000	19.32170000	0.00000000
78	1225.08250000	11.76400000	0.00000000
79	1240.08310000	49.20140000	0.00000000
80	1265.06310000	27.15200000	0.00000000
81	1269.58890000	355.57450000	0.00000000
82	1274.14810000	88.58210000	0.00000000
83	1287.32980000	85.60170000	0.00000000
84	1296.36570000	177.23600000	0.00000000
85	1318.59990000	74.41710000	0.00000000
86	1333.32510000	16.61750000	0.00000000
87	1341.15830000	39.03560000	0.00000000
88	1354.74980000	231.54720000	0.00000000
89	1382.00260000	363.33880000	0.00000000
90	1395.12930000	28.95640000	0.00000000
91	1398.21920000	67.63770000	0.00000000
92	1402.14450000	177.39310000	0.00000000
93	1421.00000000	22.39770000	0.00000000
94	1454.04200000	6.23110000	0.00000000
95	1467.36030000	0.34400000	0.00000000
96	1476.66830000	17.80240000	0.00000000
97	1479.93290000	15.61070000	0.00000000
98	1485.19370000	2.30320000	0.00000000
99	1485.27540000	11.14620000	0.00000000
100	1486.63960000	1.99750000	0.00000000
101	1488.29320000	50.06720000	0.00000000
102	1493.22490000	35.58610000	0.00000000
103	1498.89980000	49.22290000	0.00000000

104	1499.46800000	2.18740000	0.00000000
105	1510.48810000	201.78340000	0.00000000
106	1519.23100000	52.18550000	0.00000000
107	1542.61250000	162.70620000	0.00000000
108	1616.65630000	22.08700000	0.00000000
109	1630.12700000	12.35650000	0.00000000
110	1641.57100000	62.78140000	0.00000000
111	1651.33250000	80.61920000	0.00000000
112	1722.08920000	750.80580000	0.00000000
113	3010.10740000	68.81990000	0.00000000
114	3032.71720000	38.39270000	0.00000000
115	3034.89010000	14.97210000	0.00000000
116	3036.52400000	35.50910000	0.00000000
117	3043.54400000	20.40220000	0.00000000
118	3070.94630000	50.76140000	0.00000000
119	3091.44910000	16.74460000	0.00000000
120	3097.52940000	9.04230000	0.00000000
121	3099.18430000	26.38180000	0.00000000
122	3099.88420000	24.98190000	0.00000000
123	3107.34950000	47.88790000	0.00000000
124	3112.09190000	69.47810000	0.00000000
125	3138.05590000	31.49830000	0.00000000
126	3139.92710000	0.61430000	0.00000000
127	3142.72050000	25.74290000	0.00000000
128	3160.03640000	17.70510000	0.00000000
129	3163.51980000	1.77730000	0.00000000
130	3171.28590000	26.62680000	0.00000000
131	3176.92350000	12.30990000	0.00000000
132	3187.73360000	44.41940000	0.00000000
133	3187.82730000	6.84420000	0.00000000
134	3206.09650000	9.52200000	0.00000000
135	3248.98480000	2.08560000	0.00000000

N-Boc-2-(*p*-methoxyphenyl)indoline Clockwise TS from 2d-1 to 2d-2

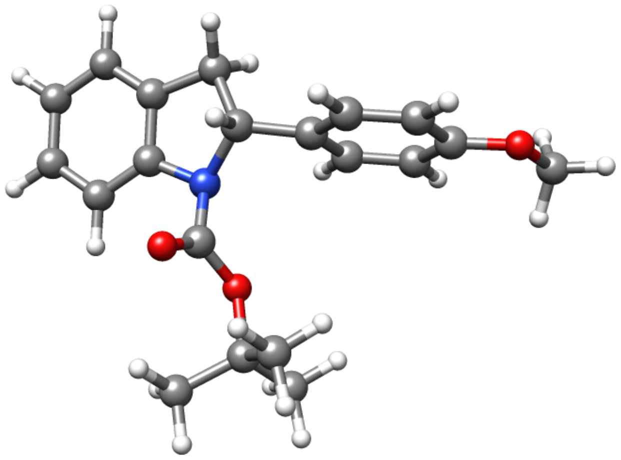

Route : # opt=qst3 freq b3lyp/6-311g(d,p) scrf=(solvent=thf)
geom=connectivity int=ultrafine

SMILES : COc3ccc(C2Cc1ccccc1N2C(=O)OC(C)(C)C)cc3

Formula : $C_{20}H_{23}NO_3$

Charge : 0

Multiplicity : 1

Dipole : 3.9008 Debye

Energy : -1056.69041046 a.u.

Gibbs Energy : -1056.35179000 a.u.

Number of imaginary frequencies : 1

Cartesian Coordinates (XYZ format)

47

C	2.47921700	-2.15803700	-0.01249100
C	2.48901400	-0.77010300	-0.20548600
C	1.07621900	-2.58483300	0.34276200
C	0.40639600	-1.24867600	0.76060700
H	1.02250400	-3.32253200	1.14500100
H	0.56638500	-3.00534500	-0.53127400
N	1.19674600	-0.23389700	-0.00940200
H	0.59607700	-1.08794400	1.83025300
C	-1.08213000	-1.16422800	0.51179800
C	3.63554600	-2.89646600	-0.20792100
C	4.81358500	-2.23895300	-0.58474800
C	4.81319600	-0.85705800	-0.76917900
C	3.65071400	-0.10237800	-0.58366700
H	3.63158100	-3.97154500	-0.06188300
H	5.72664600	-2.80446100	-0.72953100
H	5.72903100	-0.35257100	-1.05650500
H	3.66345000	0.97141400	-0.72898900

C	1.09661800	1.11018000	0.49027600
O	0.45133200	1.86648400	-0.38924700
O	1.53422400	1.45989300	1.56029800
C	0.15167200	3.30586100	-0.12495800
C	-0.60177400	3.71499500	-1.38875700
H	0.02512300	3.57693200	-2.27222200
H	-0.88661700	4.76744300	-1.32543700
H	-1.50711300	3.11614600	-1.50677900
C	-0.74104400	3.42756800	1.11005300
H	-1.63436900	2.80895400	0.99730300
H	-1.05829000	4.46762100	1.21889200
H	-0.21374400	3.12959400	2.01486000
C	1.45985000	4.08478500	0.01233700
H	2.09197800	3.92587200	-0.86476200
H	2.00802900	3.78898900	0.90543800
H	1.23524300	5.15210600	0.07950500
C	-1.60522700	-1.04713300	-0.77701600
C	-1.98044200	-1.24534800	1.58246500
C	-2.98014700	-1.01832600	-1.00476400
C	-3.86465200	-1.11058600	0.07603900
C	-3.35296900	-1.22314200	1.37418800
H	-0.92974900	-0.96224100	-1.62025000
H	-3.34680000	-0.92195500	-2.01770700
O	-5.22314700	-1.09410600	-0.03107500
H	-1.60024400	-1.32652300	2.59569900
H	-4.04749600	-1.28592200	2.20343400
C	-5.80262000	-0.98193800	-1.32998700
H	-6.87977500	-0.99141000	-1.17527400
H	-5.52019400	-1.82601800	-1.96699500
H	-5.51426900	-0.04459000	-1.81608700

Frequencies

Mode	IR frequency	IR intensity	Raman intensity
1	-42.16630000	1.76730000	0.00000000
2	24.74110000	0.23190000	0.00000000
3	33.71290000	1.41490000	0.00000000
4	42.30880000	0.27600000	0.00000000
5	46.57470000	0.55590000	0.00000000
6	65.65550000	2.31900000	0.00000000
7	91.89570000	2.09270000	0.00000000
8	103.05190000	1.47220000	0.00000000
9	139.57740000	4.93510000	0.00000000
10	146.11080000	1.81760000	0.00000000
11	158.86580000	1.17670000	0.00000000
12	173.54730000	0.52870000	0.00000000
13	201.66610000	0.14720000	0.00000000
14	223.65300000	7.32810000	0.00000000
15	232.97770000	0.60030000	0.00000000
16	245.91620000	2.39070000	0.00000000

17	250.19510000	1.07840000	0.00000000
18	264.72310000	3.67200000	0.00000000
19	267.80280000	2.23200000	0.00000000
20	268.32260000	0.89330000	0.00000000
21	300.55510000	0.60330000	0.00000000
22	318.63180000	8.94690000	0.00000000
23	350.99980000	3.12240000	0.00000000
24	365.51080000	0.38320000	0.00000000
25	419.04280000	3.97580000	0.00000000
26	427.96320000	0.84380000	0.00000000
27	428.88090000	5.23650000	0.00000000
28	456.20730000	2.29300000	0.00000000
29	464.89510000	6.12980000	0.00000000
30	471.31550000	3.30840000	0.00000000
31	486.72390000	5.22060000	0.00000000
32	529.08120000	4.40540000	0.00000000
33	538.66110000	16.53780000	0.00000000
34	558.28970000	24.19020000	0.00000000
35	562.29440000	30.88520000	0.00000000
36	606.89070000	1.79480000	0.00000000
37	617.43120000	24.98590000	0.00000000
38	649.07020000	5.87400000	0.00000000
39	707.58560000	12.25330000	0.00000000
40	739.67120000	24.56750000	0.00000000
41	743.15900000	15.71580000	0.00000000
42	745.87450000	21.84620000	0.00000000
43	749.54880000	42.95450000	0.00000000
44	771.02450000	28.97740000	0.00000000
45	801.41190000	19.27940000	0.00000000
46	823.78030000	7.27480000	0.00000000
47	828.51430000	3.79210000	0.00000000
48	837.86530000	15.97240000	0.00000000
49	845.41120000	88.90350000	0.00000000
50	849.85870000	42.61310000	0.00000000
51	858.76280000	3.75600000	0.00000000
52	883.44020000	1.19070000	0.00000000
53	927.27300000	0.02040000	0.00000000
54	929.20210000	0.25140000	0.00000000
55	932.50730000	6.40500000	0.00000000
56	956.78010000	0.90080000	0.00000000
57	962.37880000	35.25170000	0.00000000
58	971.49450000	0.11200000	0.00000000
59	976.05420000	0.38200000	0.00000000

60	978.89140000	0.50870000	0.00000000
61	1006.09200000	35.01160000	0.00000000
62	1024.64090000	1.22390000	0.00000000
63	1038.46030000	13.84500000	0.00000000
64	1046.04260000	43.53380000	0.00000000
65	1050.23050000	4.93130000	0.00000000
66	1051.76620000	58.45200000	0.00000000
67	1053.01980000	36.32970000	0.00000000
68	1071.29900000	45.70760000	0.00000000
69	1128.66800000	23.01450000	0.00000000
70	1136.65720000	26.02570000	0.00000000
71	1163.97950000	792.74050000	0.00000000
72	1168.92540000	1.47510000	0.00000000
73	1175.06070000	1.07180000	0.00000000
74	1182.78920000	28.79440000	0.00000000
75	1191.97450000	93.56970000	0.00000000
76	1200.30910000	1.03250000	0.00000000
77	1202.15800000	17.09040000	0.00000000
78	1229.18930000	8.69680000	0.00000000
79	1258.02970000	187.06470000	0.00000000
80	1263.31140000	242.59540000	0.00000000
81	1268.11090000	46.32150000	0.00000000
82	1271.47570000	596.81860000	0.00000000
83	1274.80010000	104.41440000	0.00000000
84	1282.60750000	113.84690000	0.00000000
85	1309.94070000	111.13900000	0.00000000
86	1331.31090000	10.74450000	0.00000000
87	1331.87690000	51.61700000	0.00000000
88	1343.13320000	11.54250000	0.00000000
89	1368.91370000	57.77370000	0.00000000
90	1396.17840000	19.78460000	0.00000000
91	1397.07840000	23.27120000	0.00000000
92	1399.85320000	27.92460000	0.00000000
93	1421.81550000	19.55160000	0.00000000
94	1454.86360000	11.67600000	0.00000000
95	1467.29510000	0.30010000	0.00000000
96	1476.74450000	17.38890000	0.00000000
97	1478.16210000	2.22380000	0.00000000
98	1484.72670000	1.63790000	0.00000000
99	1485.08660000	0.74260000	0.00000000
100	1485.52850000	11.25630000	0.00000000
101	1489.71750000	28.75120000	0.00000000
102	1490.94660000	20.58420000	0.00000000

103	1497.71520000	2.17760000	0.00000000
104	1498.66830000	50.94340000	0.00000000
105	1510.49590000	138.57690000	0.00000000
106	1517.57070000	22.62190000	0.00000000
107	1543.03420000	163.76970000	0.00000000
108	1615.60950000	19.03320000	0.00000000
109	1629.99490000	2.49440000	0.00000000
110	1642.86500000	54.54920000	0.00000000
111	1650.64140000	96.46180000	0.00000000
112	1761.63480000	407.38790000	0.00000000
113	2991.85950000	43.08210000	0.00000000
114	3011.46860000	67.68240000	0.00000000
115	3025.07680000	39.73020000	0.00000000
116	3037.05070000	12.98030000	0.00000000
117	3038.59940000	30.16830000	0.00000000
118	3045.89620000	17.35570000	0.00000000
119	3072.90030000	49.87950000	0.00000000
120	3093.04400000	24.44950000	0.00000000
121	3100.66280000	12.25480000	0.00000000
122	3102.77190000	15.75380000	0.00000000
123	3111.30670000	43.13490000	0.00000000
124	3114.20850000	61.29650000	0.00000000
125	3136.28700000	1.52550000	0.00000000
126	3139.05830000	31.77430000	0.00000000
127	3139.20990000	26.98550000	0.00000000
128	3161.62990000	5.01620000	0.00000000
129	3162.10360000	16.18320000	0.00000000
130	3168.40220000	15.09070000	0.00000000
131	3180.69890000	14.72790000	0.00000000
132	3180.76660000	35.31420000	0.00000000
133	3189.18480000	14.88520000	0.00000000
134	3189.87080000	24.60520000	0.00000000
135	3206.51030000	8.99840000	0.00000000

N-Boc-2-(*p*-methoxyphenyl)indoline Anticlockwise TS from 2d-1 to 2d-2

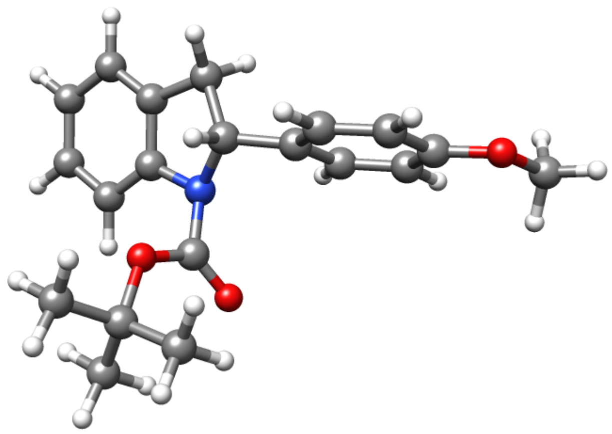

```
Route : # opt=qst3 freq b3lyp/6-311g(d,p) scrf=(solvent=thf)
          geom=connectivity int=ultrafine
```

SMILES : COc3ccc(C2Cc1ccccc1N2C(=O)OC(C)(C)C)cc3

Formula : $C_{20}H_{23}NO_3$

Charge : 0

Multiplicity : 1

Dipole : 1.6746 Debye

Energy : -1056.69013047 a.u.

Gibbs Energy : -1056.35146400 a.u.

Number of imaginary frequencies : 1

Cartesian Coordinates (XYZ format)

47

C	-1.66564600	2.45254100	0.47656800
C	-1.91318800	1.35295100	-0.35608600
C	-0.26501200	2.33593400	1.02615800
C	0.08670300	0.84515700	0.76735600
H	-0.17723000	2.58523500	2.08483400
H	0.42208000	2.98574000	0.47274400
N	-0.78608300	0.50487500	-0.40104300
H	-0.24890200	0.25804300	1.63211600
C	1.55229900	0.56238400	0.52709200
C	-2.62336200	3.44326900	0.62418300
C	-3.84215900	3.32620900	-0.05627800
C	-4.07731200	2.22837100	-0.88277200
C	-3.11556300	1.22660000	-1.04681600
H	-2.43464300	4.29775000	1.26558000
H	-4.60261500	4.08948900	0.06001200
H	-5.02221700	2.14303800	-1.40782600
H	-3.30629000	0.38170000	-1.69811400
C	-0.96551400	-0.89043600	-0.67881100
O	-1.70922800	-1.46782500	0.26660700

O	-0.48030900	-1.43288400	-1.63901000
C	-2.04878800	-2.92243100	0.24530500
C	-2.89576100	-3.07620100	1.50638400
H	-2.31950400	-2.80423300	2.39328800
H	-3.22177300	-4.11346600	1.60946200
H	-3.77974500	-2.43715100	1.45591900
C	-2.86485500	-3.23915000	-1.00771900
H	-3.74215900	-2.59039200	-1.06558400
H	-3.21130100	-4.27433500	-0.95703200
H	-2.27051900	-3.11670700	-1.91187400
C	-0.76501100	-3.74656800	0.33830900
H	-0.18372100	-3.44972500	1.21458400
H	-0.14997700	-3.62767400	-0.55234500
H	-1.02426300	-4.80285700	0.44429900
C	2.16868800	0.85389500	-0.69035100
C	2.34229600	0.03794400	1.55745500
C	3.53243400	0.63913500	-0.88495400
C	4.30983900	0.12387400	0.15799200
C	3.70252400	-0.17638500	1.38332100
H	1.57228500	1.23910400	-1.50885100
H	3.97192700	0.86990200	-1.84584200
O	5.64754300	-0.12483000	0.07835700
H	1.88606700	-0.20739500	2.51126500
H	4.31315200	-0.58332700	2.18052300
C	6.31980500	0.15261200	-1.14910500
H	7.36084300	-0.11820700	-0.98400200
H	6.25781300	1.21460400	-1.40675000
H	5.91473500	-0.44718500	-1.97014200

Frequencies

Mode	IR frequency	IR intensity	Raman intensity
1	-36.27880000	1.36450000	0.00000000
2	23.90970000	0.56790000	0.00000000
3	34.48770000	1.64770000	0.00000000
4	43.42590000	0.73440000	0.00000000
5	51.23320000	1.06210000	0.00000000
6	60.82660000	1.33420000	0.00000000
7	94.61380000	3.08630000	0.00000000
8	101.24510000	1.47260000	0.00000000
9	136.99380000	3.31660000	0.00000000
10	143.98350000	1.45380000	0.00000000
11	163.96070000	1.92810000	0.00000000
12	174.91670000	1.13020000	0.00000000
13	201.33630000	0.07770000	0.00000000
14	208.58390000	4.65140000	0.00000000
15	239.91730000	3.27660000	0.00000000
16	245.46820000	4.28490000	0.00000000
17	251.65940000	0.59780000	0.00000000
18	257.59030000	3.92140000	0.00000000

19	266.50050000	0.10100000	0.00000000
20	280.98010000	2.52270000	0.00000000
21	318.15910000	8.56900000	0.00000000
22	340.56570000	1.52430000	0.00000000
23	353.01010000	1.38450000	0.00000000
24	374.41900000	4.38050000	0.00000000
25	408.00820000	2.36830000	0.00000000
26	428.17290000	0.81420000	0.00000000
27	429.52710000	7.05640000	0.00000000
28	455.48440000	3.37980000	0.00000000
29	463.76690000	3.90510000	0.00000000
30	471.10430000	4.22630000	0.00000000
31	485.91660000	4.72650000	0.00000000
32	514.53300000	11.31170000	0.00000000
33	540.08740000	10.64430000	0.00000000
34	554.06910000	20.58810000	0.00000000
35	561.88130000	33.23170000	0.00000000
36	610.18720000	6.51980000	0.00000000
37	617.81910000	15.07290000	0.00000000
38	645.97780000	1.92890000	0.00000000
39	685.26610000	2.50460000	0.00000000
40	731.87470000	6.44940000	0.00000000
41	741.80090000	3.22180000	0.00000000
42	751.05560000	51.57730000	0.00000000
43	759.05420000	34.06900000	0.00000000
44	774.27470000	24.09820000	0.00000000
45	799.40740000	12.63290000	0.00000000
46	828.59690000	4.94420000	0.00000000
47	830.29850000	18.86810000	0.00000000
48	838.06560000	9.36290000	0.00000000
49	844.61120000	142.83770000	0.00000000
50	845.36160000	18.11960000	0.00000000
51	862.54910000	8.78100000	0.00000000
52	882.35890000	3.99280000	0.00000000
53	927.63270000	0.04190000	0.00000000
54	929.28260000	0.16600000	0.00000000
55	934.53330000	6.68750000	0.00000000
56	957.05350000	0.67250000	0.00000000
57	962.83670000	30.63890000	0.00000000
58	972.11800000	0.16090000	0.00000000
59	976.34450000	1.13540000	0.00000000
60	979.37640000	0.18640000	0.00000000
61	1005.07180000	53.43130000	0.00000000

62	1024.90050000	1.16500000	0.00000000
63	1038.06440000	18.92690000	0.00000000
64	1046.37770000	38.68930000	0.00000000
65	1050.14820000	40.78800000	0.00000000
66	1050.86930000	2.32540000	0.00000000
67	1053.74640000	38.87140000	0.00000000
68	1071.51950000	49.01710000	0.00000000
69	1127.15850000	62.57020000	0.00000000
70	1134.26150000	47.71690000	0.00000000
71	1154.64710000	788.52910000	0.00000000
72	1169.29790000	1.15250000	0.00000000
73	1174.97960000	8.48530000	0.00000000
74	1181.59750000	27.98850000	0.00000000
75	1190.91310000	51.35060000	0.00000000
76	1199.08820000	4.86900000	0.00000000
77	1202.38900000	21.74730000	0.00000000
78	1228.10340000	11.62000000	0.00000000
79	1239.60850000	213.72990000	0.00000000
80	1260.76340000	74.79360000	0.00000000
81	1267.88470000	30.94630000	0.00000000
82	1271.08680000	334.89620000	0.00000000
83	1277.86530000	87.28210000	0.00000000
84	1286.97060000	242.35020000	0.00000000
85	1310.40700000	91.78550000	0.00000000
86	1330.95670000	11.32540000	0.00000000
87	1332.06410000	22.52730000	0.00000000
88	1343.68940000	13.42750000	0.00000000
89	1369.10730000	60.18580000	0.00000000
90	1394.54880000	12.35160000	0.00000000
91	1398.00130000	27.33930000	0.00000000
92	1400.42250000	31.38700000	0.00000000
93	1423.83510000	19.14240000	0.00000000
94	1453.79950000	9.26420000	0.00000000
95	1467.69360000	0.55090000	0.00000000
96	1476.82950000	17.68270000	0.00000000
97	1478.05950000	4.22170000	0.00000000
98	1484.16360000	0.25950000	0.00000000
99	1485.05230000	2.04170000	0.00000000
100	1485.75830000	11.23890000	0.00000000
101	1490.14260000	62.19140000	0.00000000
102	1491.59310000	5.60860000	0.00000000
103	1497.65410000	2.68830000	0.00000000
104	1499.63070000	54.01850000	0.00000000

105	1509.99850000	160.85760000	0.00000000
106	1517.66460000	22.90360000	0.00000000
107	1542.86910000	163.03270000	0.00000000
108	1615.79390000	20.51840000	0.00000000
109	1629.82290000	8.02210000	0.00000000
110	1642.53680000	77.08360000	0.00000000
111	1650.93410000	94.20100000	0.00000000
112	1778.31550000	521.62630000	0.00000000
113	2995.20850000	41.22390000	0.00000000
114	3011.44550000	69.04320000	0.00000000
115	3025.77750000	40.98700000	0.00000000
116	3036.77920000	13.06500000	0.00000000
117	3038.91720000	31.30190000	0.00000000
118	3045.60640000	16.22790000	0.00000000
119	3072.96760000	49.47620000	0.00000000
120	3093.02240000	23.70540000	0.00000000
121	3100.86480000	7.48380000	0.00000000
122	3102.32760000	19.99140000	0.00000000
123	3110.44030000	45.44060000	0.00000000
124	3113.77590000	61.03510000	0.00000000
125	3136.15080000	1.10560000	0.00000000
126	3138.63400000	31.89920000	0.00000000
127	3139.00070000	26.32440000	0.00000000
128	3159.92530000	17.45670000	0.00000000
129	3161.79020000	5.07670000	0.00000000
130	3168.50610000	15.14230000	0.00000000
131	3180.90500000	37.71380000	0.00000000
132	3183.32030000	11.28540000	0.00000000
133	3188.62830000	12.80330000	0.00000000
134	3189.93970000	25.63050000	0.00000000
135	3207.51810000	9.36170000	0.00000000

Thermochemistry Data for *N*-Boc-2-(*p*-methoxyphenyl)indoline Calculations

N-Boc-2-(*p*-methoxyphenyl)indoline Rotamer **2d-1**:

	Temperature		
	298 K	223 K	195 K
ε_0	–1056.71131073 a.u.	–1056.71131073 a.u.	–1056.71131073 a.u.
ε_{ZPE}	0.390886 a.u.	0.390886 a.u.	0.390886 a.u.
E_{tot}	0.413409 a.u.	0.406383 a.u.	0.401342 a.u.
H_{corr}	0.414353 a.u.	0.407152 a.u.	0.401960 a.u.
G_{corr}	0.337476 a.u.	0.350964 a.u.	0.361496 a.u.
S_{tot}	161.802 calmol^{-1}K^{-1}	145.097 calmol^{-1}K^{-1}	130.210 calmol^{-1}K^{-1}
$\varepsilon_0 + \varepsilon_{ZPE}$	–1056.320425 a.u.	–1056.320425 a.u.	–1056.320425 a.u.
$\varepsilon_0 + E_{tot}$	–1056.297902 a.u.	–1056.304928 a.u.	–1056.309969 a.u.
$\varepsilon_0 + H_{corr}$	–1056.296958 a.u.	–1056.304159 a.u.	–1056.309351 a.u.
$\varepsilon_0 + G_{corr}$	–1056.373835 a.u.	–1056.360347 a.u.	–1056.349815 a.u.

N-Boc-2-(*p*-methoxyphenyl)indoline Rotamer **2d-2**:

	Temperature		
	298 K	223 K	195 K
ε_0	–1056.70957881 a.u.	–1056.70957881 a.u.	–1056.70957881 a.u.
ε_{ZPE}	0.390816 a.u.	0.390816 a.u.	0.390816 a.u.
E_{tot}	0.413363 a.u.	0.406333 a.u.	0.401290 a.u.
H_{corr}	0.414307 a.u.	0.407103 a.u.	0.401908 a.u.
G_{corr}	0.337143 a.u.	0.350684 a.u.	0.361262 a.u.
S_{tot}	162.405 calmol^{-1}K^{-1}	145.692 calmol^{-1}K^{-1}	130.798 calmol^{-1}K^{-1}
$\varepsilon_0 + \varepsilon_{ZPE}$	–1056.318763 a.u.	–1056.318763 a.u.	–1056.318763 a.u.
$\varepsilon_0 + E_{tot}$	–1056.296216 a.u.	–1056.303246 a.u.	–1056.308289 a.u.
$\varepsilon_0 + H_{corr}$	–1056.295272 a.u.	–1056.302476 a.u.	–1056.307671 a.u.
$\varepsilon_0 + G_{corr}$	–1056.372436 a.u.	–1056.358895 a.u.	–1056.348317 a.u.

TS for *N*-Boc-2-(*p*-methoxyphenyl)indoline Clockwise Rotation of Boc Group from Rotamer **2d-1** to Rotamer **2d-2**:

	Temperature	
	298 K	195 K
ε_0	–1056.69041046 a.u.	–1056.69041046 a.u.
ε_{ZPE}	0.389963 a.u.	0.389963 a.u.
E_{tot}	0.411756 a.u.	0.399970 a.u.
H_{corr}	0.412700 a.u.	0.400587 a.u.
G_{corr}	0.338620 a.u.	0.361729 a.u.
S_{tot}	155.915 calmol^{-1}K^{-1}	125.046 calmol^{-1}K^{-1}
$\varepsilon_0 + \varepsilon_{ZPE}$	–1056.300448 a.u.	–1056.300447 a.u.
$\varepsilon_0 + E_{tot}$	–1056.278654 a.u.	–1056.290440 a.u.
$\varepsilon_0 + H_{corr}$	–1056.277710 a.u.	–1056.289823 a.u.
$\varepsilon_0 + G_{corr}$	–1056.351790 a.u.	–1056.328681 a.u.

TS for *N*-Boc-2-(*p*-methoxyphenyl)indoline Anticlockwise Rotation of Boc Group from Rotamer **2d-1** to Rotamer **2d-2**:

	Temperature	
	298 K	195 K
ε_0	–1056.69013047 a.u.	–1056.69013047 a.u.
ε_{ZPE}	0.389973 a.u.	0.389973 a.u.
E_{tot}	0.411738 a.u.	0.399956 a.u.
H_{corr}	0.412682 a.u.	0.400573 a.u.
G_{corr}	0.338666 a.u.	0.361753 a.u.
S_{tot}	155.779 calmol^{-1}K^{-1}	124.923 calmol^{-1}K^{-1}
$\varepsilon_0 + \varepsilon_{ZPE}$	–1056.300158 a.u.	–1056.300157 a.u.
$\varepsilon_0 + E_{tot}$	–1056.278392 a.u.	–1056.290174 a.u.
$\varepsilon_0 + H_{corr}$	–1056.277448 a.u.	–1056.289557 a.u.
$\varepsilon_0 + G_{corr}$	–1056.351464 a.u.	–1056.328377 a.u.

10.2 Calculations Performed Using the 6-311G(d,p) Basis Set with dispersion

N-Boc-2-phenylindoline 2a-1

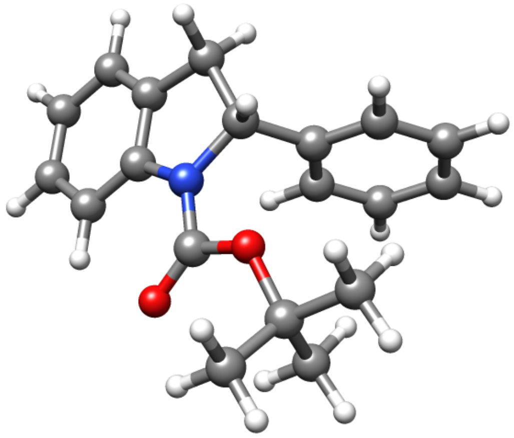

```
Route : # opt freq b3lyp/6-311g(d,p) scrf=(solvent=thf)
          geom=connectivity empiricaldispersion=gd3bj
          int=ultrafine pop=(regular,mk)
```

SMILES : CC(C)(C)OC(=O)N2c1ccccc1CC2c3ccccc3

Formula : $C_{19}H_{21}NO_2$

Charge : 0

Multiplicity : 1

Dipole : 3.8085 Debye

Energy : -942.25114587 a.u.

Gibbs Energy : -941.94134100 a.u.

Number of imaginary frequencies : 0

Cartesian Coordinates (XYZ format)

43

C	-4.80438700	-0.20274800	-0.37722400
C	-4.13728800	-1.28841400	-0.94125100
C	-4.11260900	0.69248100	0.44531100
C	-2.76483700	0.48538300	0.68851800
C	-2.10343800	-0.60451800	0.11015800
C	-2.77662900	-1.50771900	-0.70915900
C	-1.81059700	1.26862900	1.55377500
C	-0.41501500	0.67229400	1.20979700
H	-2.03833800	1.12211400	2.61332700
H	-1.83956200	2.34053700	1.35496300
N	-0.74553900	-0.58396400	0.48609600
H	-5.85850900	-0.05092300	-0.57593600

H	-4.62422200	1.53871400	0.89004100
H	-4.67769300	-1.97863800	-1.57875600
H	-2.25305000	-2.34034700	-1.15128800
C	0.21143500	-1.47444700	0.07273100
O	0.00448900	-2.41594600	-0.67077600
O	1.39334000	-1.14878900	0.62611300
C	2.66412700	-1.68122400	0.09622000
C	2.75568100	-3.18111500	0.36690600
H	2.60616000	-3.38051400	1.43067900
H	3.74923600	-3.54007000	0.08715500
H	2.00696500	-3.72643500	-0.20397600
C	2.77626000	-1.34151500	-1.38903700
H	3.76990900	-1.61837100	-1.74850800
H	2.63929800	-0.26817600	-1.53895300
H	2.03176100	-1.88001100	-1.97348000
C	3.70266300	-0.91094900	0.90612100
H	3.57977000	-1.11149300	1.97257200
H	3.59426200	0.16190200	0.73728000
H	4.70746800	-1.21681200	0.60762000
H	0.13506500	0.42343400	2.11467400
C	0.43381400	1.59544300	0.35919400
C	0.19614700	1.73644200	-1.01012700
C	1.46111100	2.33213200	0.94832300
C	0.97704800	2.59838300	-1.77565100
C	2.24224600	3.19750400	0.18531100
C	2.00315400	3.33148100	-1.18080300
H	1.66108900	2.21487600	2.00793300
H	3.04282500	3.75724800	0.65500200
H	2.61379700	3.99803700	-1.77842500
H	-0.59049600	1.16092400	-1.48353400
H	0.78708800	2.69448400	-2.83842800

Frequencies

Mode	IR frequency	IR intensity	Raman intensity
1	27.70460000	0.18940000	0.00000000
2	30.59620000	0.02020000	0.00000000
3	36.81050000	0.90930000	0.00000000
4	43.12840000	0.54160000	0.00000000
5	69.63090000	2.86340000	0.00000000
6	87.70220000	1.39260000	0.00000000
7	102.38310000	0.71310000	0.00000000
8	135.79680000	0.30090000	0.00000000
9	181.35080000	0.91870000	0.00000000
10	211.97040000	1.66420000	0.00000000
11	214.72100000	0.22390000	0.00000000
12	220.21150000	6.54170000	0.00000000
13	229.38340000	5.80630000	0.00000000
14	259.00980000	0.15870000	0.00000000
15	273.42980000	3.30890000	0.00000000

16	279.81670000	0.35780000	0.00000000
17	295.06060000	1.30030000	0.00000000
18	332.16660000	15.76150000	0.00000000
19	344.22730000	1.81430000	0.00000000
20	358.03030000	1.44440000	0.00000000
21	407.72320000	4.37680000	0.00000000
22	412.83430000	0.16000000	0.00000000
23	429.04240000	1.01330000	0.00000000
24	432.15280000	5.39900000	0.00000000
25	461.66730000	6.65490000	0.00000000
26	473.21310000	3.04020000	0.00000000
27	491.79040000	0.96810000	0.00000000
28	543.53280000	2.64910000	0.00000000
29	561.73790000	7.36820000	0.00000000
30	596.00460000	10.47030000	0.00000000
31	627.03240000	22.33390000	0.00000000
32	635.87460000	9.04180000	0.00000000
33	637.27530000	0.96500000	0.00000000
34	713.91480000	64.45340000	0.00000000
35	725.67180000	6.67570000	0.00000000
36	747.73350000	30.49820000	0.00000000
37	763.77110000	60.92090000	0.00000000
38	771.37430000	20.69470000	0.00000000
39	780.05580000	32.42570000	0.00000000
40	782.49400000	24.28450000	0.00000000
41	800.64910000	5.93370000	0.00000000
42	821.15840000	5.75930000	0.00000000
43	854.82180000	58.40180000	0.00000000
44	858.07680000	2.44200000	0.00000000
45	869.76700000	5.91260000	0.00000000
46	880.08740000	3.73680000	0.00000000
47	928.88980000	3.14150000	0.00000000
48	930.91720000	0.37570000	0.00000000
49	931.89140000	0.23300000	0.00000000
50	945.58260000	1.05220000	0.00000000
51	973.96770000	0.17870000	0.00000000
52	982.07030000	9.02420000	0.00000000
53	985.78870000	0.23860000	0.00000000
54	991.68390000	0.29110000	0.00000000
55	1006.04840000	0.17780000	0.00000000
56	1020.68320000	0.60580000	0.00000000
57	1024.30470000	3.35510000	0.00000000
58	1037.19760000	148.75490000	0.00000000

59	1049.21810000	12.21460000	0.00000000
60	1053.05770000	24.35520000	0.00000000
61	1054.33690000	3.80970000	0.00000000
62	1062.70470000	28.71140000	0.00000000
63	1103.14270000	45.48280000	0.00000000
64	1111.21960000	2.76230000	0.00000000
65	1156.41850000	349.92500000	0.00000000
66	1174.79610000	7.82210000	0.00000000
67	1177.27660000	34.68920000	0.00000000
68	1178.98240000	14.07800000	0.00000000
69	1188.21760000	309.83500000	0.00000000
70	1199.81790000	41.93940000	0.00000000
71	1202.13720000	19.15830000	0.00000000
72	1232.30620000	7.06970000	0.00000000
73	1245.60300000	9.85820000	0.00000000
74	1270.37600000	44.29310000	0.00000000
75	1276.89040000	75.32360000	0.00000000
76	1287.27440000	38.98830000	0.00000000
77	1303.80300000	171.46670000	0.00000000
78	1315.57840000	32.93020000	0.00000000
79	1334.37450000	47.15460000	0.00000000
80	1352.44800000	104.49630000	0.00000000
81	1353.91950000	17.32470000	0.00000000
82	1389.56970000	105.57060000	0.00000000
83	1396.56130000	31.11210000	0.00000000
84	1399.78700000	31.95340000	0.00000000
85	1415.72760000	632.56360000	0.00000000
86	1422.64970000	19.78570000	0.00000000
87	1468.09490000	0.45530000	0.00000000
88	1479.90030000	20.27750000	0.00000000
89	1485.76560000	2.74980000	0.00000000
90	1487.90080000	3.14890000	0.00000000
91	1488.90140000	18.63560000	0.00000000
92	1491.29390000	17.16350000	0.00000000
93	1495.17160000	34.79050000	0.00000000
94	1499.73930000	2.05490000	0.00000000
95	1514.61640000	219.35140000	0.00000000
96	1520.87710000	63.53100000	0.00000000
97	1529.48120000	14.78730000	0.00000000
98	1629.22140000	0.36310000	0.00000000
99	1634.96500000	1.89890000	0.00000000
100	1644.26620000	43.47420000	0.00000000
101	1647.59870000	5.71030000	0.00000000

102	1735.58440000	580.88920000	0.00000000
103	3036.90820000	14.02000000	0.00000000
104	3038.55010000	34.78320000	0.00000000
105	3041.86220000	37.76740000	0.00000000
106	3045.43130000	24.25160000	0.00000000
107	3094.04330000	16.06100000	0.00000000
108	3099.69900000	16.69130000	0.00000000
109	3106.06790000	15.41590000	0.00000000
110	3110.64190000	41.42260000	0.00000000
111	3111.31840000	39.41440000	0.00000000
112	3117.30070000	52.12140000	0.00000000
113	3135.48600000	11.59000000	0.00000000
114	3145.04130000	18.27870000	0.00000000
115	3160.52170000	7.32410000	0.00000000
116	3166.20860000	1.28400000	0.00000000
117	3167.36260000	0.62870000	0.00000000
118	3174.27560000	28.00180000	0.00000000
119	3175.83900000	21.98590000	0.00000000
120	3183.69760000	38.72750000	0.00000000
121	3189.99520000	39.48640000	0.00000000
122	3192.38040000	20.18810000	0.00000000
123	3243.20970000	1.59040000	0.00000000

N-Boc-2-phenylindoline 2a-2

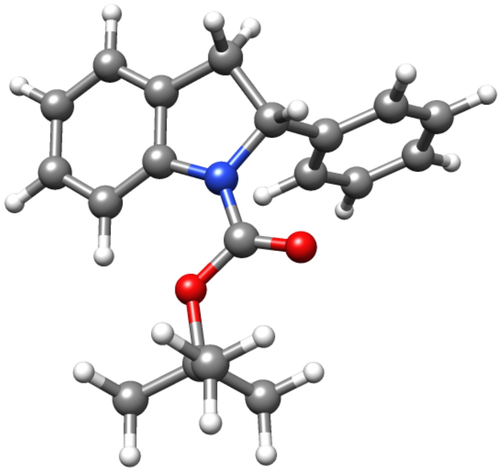

Route : # opt freq b3lyp/6-311g(d,p) scrf=(solvent=thf)
geom=connectivity empiricaldispersion=gd3bj
int=ultrafine pop=(regular,mk)

SMILES : CC(C)(C)OC(=O)N2c1ccccc1CC2c3ccccc3

Formula : $C_{19}H_{21}NO_2$

Charge : 0

Multiplicity : 1

Dipole : 1.5358 Debye

Energy : -942.24834487 a.u.

Gibbs Energy : -941.93777100 a.u.

Number of imaginary frequencies : 0

Cartesian Coordinates (XYZ format)

43

C	-0.17674700	4.28256400	0.59547000
C	0.94278800	3.56381700	1.00768100
C	-1.14105300	3.66770500	-0.20934300
C	-0.96754000	2.34650400	-0.58589800
C	0.15968400	1.62878900	-0.16198400
C	1.12878500	2.22832500	0.63951700
C	-1.82934400	1.47939700	-1.46742600
C	-1.25948000	0.05194500	-1.24142100
H	-1.72496200	1.77297000	-2.51627100
H	-2.88804700	1.52741500	-1.21204600
N	0.07531500	0.30626900	-0.65258500
H	-0.30145600	5.31519600	0.89829000
H	-2.01588700	4.21754200	-0.53797000
H	1.68808600	4.04170900	1.63302500
H	1.99411800	1.67711900	0.96819700
C	0.98503800	-0.72052200	-0.62259900

O	0.74810500	-1.81246200	-1.10665300
O	2.12272700	-0.35563100	-0.01283800
C	3.24906100	-1.30255100	0.13977200
C	2.81086700	-2.49307000	0.99009800
H	2.39167300	-2.14438900	1.93685600
H	3.67958900	-3.11831300	1.20937300
H	2.06577300	-3.09282400	0.47155200
C	3.76570200	-1.71935300	-1.23555900
H	4.67628300	-2.31070900	-1.11410200
H	4.00818300	-0.83520000	-1.82981100
H	3.02685600	-2.31455200	-1.76809700
C	4.28637600	-0.46048400	0.87691400
H	3.89747700	-0.13156600	1.84288200
H	4.55324400	0.41961500	0.28840900
H	5.18797600	-1.05164400	1.04869700
H	-1.12946900	-0.47864000	-2.18245200
C	-2.13399600	-0.78145700	-0.32476800
C	-2.02744600	-0.69434600	1.06492000
C	-3.10780100	-1.61430700	-0.87876500
C	-2.88355700	-1.42603700	1.88481100
C	-3.96571500	-2.34555200	-0.06092800
C	-3.85636700	-2.25237600	1.32534300
H	-3.18892000	-1.69643700	-1.95755900
H	-4.71225500	-2.99371600	-0.50532500
H	-4.51938200	-2.82396600	1.96421000
H	-1.26972500	-0.06050600	1.50906400
H	-2.78854700	-1.35218200	2.96209900

Frequencies

Mode	IR frequency	IR intensity	Raman intensity
1	18.37770000	0.06160000	0.00000000
2	22.93670000	0.66040000	0.00000000
3	35.90740000	0.50450000	0.00000000
4	36.57520000	0.11530000	0.00000000
5	74.77780000	2.11800000	0.00000000
6	91.53660000	0.76200000	0.00000000
7	95.07120000	0.53300000	0.00000000
8	127.41030000	0.92690000	0.00000000
9	173.38920000	1.39530000	0.00000000
10	212.38630000	0.58920000	0.00000000
11	214.44660000	4.64200000	0.00000000
12	220.90240000	3.87200000	0.00000000
13	239.73710000	2.37140000	0.00000000
14	253.22930000	0.65290000	0.00000000
15	273.65810000	0.28060000	0.00000000
16	276.76310000	0.09060000	0.00000000
17	302.65730000	4.77120000	0.00000000
18	328.99990000	11.87110000	0.00000000

19	347.70920000	4.17760000	0.00000000
20	356.76400000	4.98070000	0.00000000
21	405.37740000	8.78900000	0.00000000
22	413.44850000	0.35020000	0.00000000
23	424.86660000	2.23370000	0.00000000
24	430.54590000	5.77050000	0.00000000
25	458.96570000	4.21650000	0.00000000
26	471.28390000	6.06330000	0.00000000
27	488.02960000	8.78150000	0.00000000
28	542.08980000	2.46350000	0.00000000
29	560.81220000	7.30550000	0.00000000
30	594.36110000	10.46850000	0.00000000
31	621.05000000	10.79490000	0.00000000
32	635.77580000	1.00890000	0.00000000
33	665.34700000	23.68520000	0.00000000
34	713.81200000	58.35410000	0.00000000
35	724.60460000	4.37750000	0.00000000
36	735.01920000	11.48230000	0.00000000
37	761.49500000	107.54160000	0.00000000
38	766.15060000	2.02390000	0.00000000
39	777.93870000	25.74240000	0.00000000
40	784.97350000	11.55220000	0.00000000
41	797.88340000	0.82830000	0.00000000
42	827.56780000	3.38480000	0.00000000
43	849.52670000	6.29740000	0.00000000
44	857.25560000	0.51910000	0.00000000
45	871.81850000	24.92830000	0.00000000
46	876.74830000	52.80870000	0.00000000
47	927.76720000	4.36040000	0.00000000
48	929.63700000	0.10060000	0.00000000
49	931.80390000	0.87420000	0.00000000
50	942.86550000	0.56620000	0.00000000
51	972.02150000	0.17030000	0.00000000
52	979.82220000	12.49580000	0.00000000
53	984.53560000	0.01180000	0.00000000
54	987.39290000	0.04650000	0.00000000
55	1006.32010000	0.94240000	0.00000000
56	1019.87240000	0.17750000	0.00000000
57	1023.82410000	27.93500000	0.00000000
58	1041.11970000	69.91900000	0.00000000
59	1051.91340000	16.18480000	0.00000000
60	1052.46770000	0.47320000	0.00000000
61	1053.58880000	5.58590000	0.00000000

62	1065.64550000	41.95080000	0.00000000
63	1102.23480000	34.92520000	0.00000000
64	1113.17260000	3.53620000	0.00000000
65	1148.36250000	229.16200000	0.00000000
66	1176.28460000	50.25620000	0.00000000
67	1178.11550000	40.75780000	0.00000000
68	1178.65720000	0.51980000	0.00000000
69	1187.84750000	233.46790000	0.00000000
70	1194.14730000	100.01980000	0.00000000
71	1202.28170000	5.56810000	0.00000000
72	1231.29920000	4.52050000	0.00000000
73	1241.00050000	31.65900000	0.00000000
74	1269.92740000	23.77360000	0.00000000
75	1279.10840000	89.95420000	0.00000000
76	1291.08750000	77.88810000	0.00000000
77	1300.15350000	146.06800000	0.00000000
78	1316.46490000	13.58500000	0.00000000
79	1340.77900000	39.30030000	0.00000000
80	1354.01180000	4.31480000	0.00000000
81	1363.14050000	202.45450000	0.00000000
82	1387.44650000	261.30140000	0.00000000
83	1395.72810000	30.07810000	0.00000000
84	1398.70330000	63.90470000	0.00000000
85	1409.27070000	350.42840000	0.00000000
86	1420.80760000	15.45370000	0.00000000
87	1467.66090000	0.30420000	0.00000000
88	1478.69980000	24.55800000	0.00000000
89	1485.47530000	2.20510000	0.00000000
90	1486.65240000	2.07100000	0.00000000
91	1487.66980000	4.08970000	0.00000000
92	1488.79100000	53.02200000	0.00000000
93	1496.05360000	36.62810000	0.00000000
94	1499.75320000	2.20060000	0.00000000
95	1513.44140000	193.14990000	0.00000000
96	1519.66010000	83.33620000	0.00000000
97	1529.32620000	15.85080000	0.00000000
98	1628.47340000	1.86810000	0.00000000
99	1633.33820000	11.34900000	0.00000000
100	1646.42380000	65.42250000	0.00000000
101	1647.58410000	8.06480000	0.00000000
102	1727.40430000	769.13700000	0.00000000
103	3036.84980000	14.53590000	0.00000000
104	3037.45580000	38.49160000	0.00000000

105	3038.92000000	36.71530000	0.00000000
106	3045.75090000	19.40070000	0.00000000
107	3099.52730000	17.43430000	0.00000000
108	3100.96760000	7.33950000	0.00000000
109	3102.75250000	27.86280000	0.00000000
110	3109.47750000	45.48100000	0.00000000
111	3110.86610000	24.69960000	0.00000000
112	3115.63650000	69.68110000	0.00000000
113	3143.00760000	0.86820000	0.00000000
114	3146.20190000	24.63250000	0.00000000
115	3158.44170000	9.47810000	0.00000000
116	3166.53110000	0.51500000	0.00000000
117	3166.62480000	1.06940000	0.00000000
118	3174.34730000	27.19640000	0.00000000
119	3175.88260000	28.71850000	0.00000000
120	3185.60050000	41.32930000	0.00000000
121	3190.46370000	37.23680000	0.00000000
122	3192.83120000	16.24250000	0.00000000
123	3254.27350000	2.13250000	0.00000000

N-Boc-2-phenylindoline Clockwise TS from 2a-1 to 2a-2

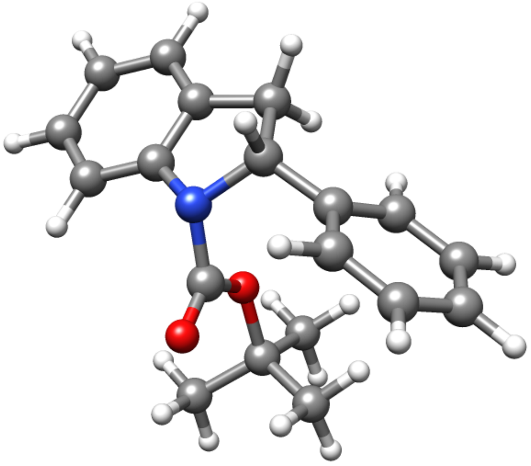

```
Route : # opt=qst3 freq b3lyp/6-311g(d,p) scrf=(solvent=thf)
          geom=connectivity empiricaldispersion=gd3bj
          int=ultrafine pop=(regular,mk)
```

SMILES : CC(C)(C)OC(=O)N2c1ccccc1CC2c3ccccc3

Formula : $C_{19}H_{21}NO_2$

Charge : 0

Multiplicity : 1

Dipole : 3.7517 Debye

Energy : -942.22236289 a.u.

Gibbs Energy : -941.90904000 a.u.

Number of imaginary frequencies : 1

Cartesian Coordinates (XYZ format)

43

C	4.61977900	0.54376700	0.56942000
C	4.34086400	-0.36280300	-0.45376600
C	3.61992600	1.39723500	1.04816400
C	2.35175700	1.33379600	0.48973800
C	2.09042400	0.43724800	-0.54991900
C	3.07066800	-0.42363200	-1.03128800
C	1.07455700	2.04097600	0.87031000
C	0.15969900	1.76602600	-0.34391700
H	1.19347100	3.11296300	1.03389900
H	0.68495300	1.60472100	1.79393900
N	0.75992300	0.54922000	-1.02690900
H	5.61480300	0.58585400	0.99610600
H	3.83150900	2.08882700	1.85620100
H	5.12123600	-1.02201700	-0.81587400
H	2.85502400	-1.11374900	-1.83824400
C	-0.01190200	-0.65765800	-1.02244700
O	-0.51060900	-1.11875400	-2.01772500

O	-0.08238500	-1.16936000	0.20632000
C	-0.83890600	-2.41544900	0.49777500
C	-2.30331100	-2.23939700	0.10361700
H	-2.71134700	-1.33481600	0.55708600
H	-2.87307200	-3.09762700	0.46782700
H	-2.41856300	-2.17485600	-0.97613400
C	-0.16777800	-3.58269700	-0.21995700
H	-0.64368100	-4.51697700	0.08670300
H	0.89050600	-3.63229200	0.04644800
H	-0.25935400	-3.48361300	-1.30049000
C	-0.69126900	-2.53525400	2.01049300
H	-1.14193100	-1.67269600	2.50533500
H	0.36312400	-2.58749200	2.28883400
H	-1.19124400	-3.44030000	2.36134100
H	0.30630700	2.57977800	-1.05997800
C	-1.32917200	1.63008300	-0.11024300
C	-1.89502900	1.46223600	1.15458900
C	-2.17693100	1.65725300	-1.22412200
C	-3.27398800	1.32653600	1.30433600
C	-3.55236600	1.50840600	-1.07991800
C	-4.10650100	1.34454800	0.18850600
H	-1.74845100	1.78305900	-2.21193100
H	-4.19081600	1.52532300	-1.95533000
H	-5.17813300	1.23394700	0.30548900
H	-1.26775300	1.43335900	2.03507600
H	-3.69521700	1.20165300	2.29507900

Frequencies

Mode	IR frequency	IR intensity	Raman intensity
1	-48.72140000	0.60230000	0.00000000
2	15.26890000	1.50480000	0.00000000
3	42.00050000	0.55280000	0.00000000
4	52.73220000	0.49740000	0.00000000
5	73.01830000	0.06490000	0.00000000
6	84.16190000	2.31930000	0.00000000
7	104.41690000	0.77710000	0.00000000
8	149.85610000	2.62190000	0.00000000
9	153.39570000	1.42330000	0.00000000
10	189.59820000	2.48660000	0.00000000
11	208.40080000	0.19230000	0.00000000
12	211.93230000	0.09020000	0.00000000
13	247.19550000	7.96510000	0.00000000
14	249.10580000	0.77260000	0.00000000
15	269.65150000	0.01740000	0.00000000
16	273.89990000	0.05610000	0.00000000
17	288.28760000	3.24190000	0.00000000
18	321.61860000	7.49030000	0.00000000
19	355.49360000	3.59510000	0.00000000

20	379.93150000	1.80960000	0.00000000
21	412.53060000	0.21870000	0.00000000
22	416.79810000	2.35810000	0.00000000
23	433.03330000	11.43980000	0.00000000
24	445.27650000	2.22320000	0.00000000
25	466.96910000	1.99200000	0.00000000
26	475.17330000	4.16160000	0.00000000
27	499.69530000	2.75030000	0.00000000
28	520.73830000	7.89500000	0.00000000
29	571.04840000	15.61450000	0.00000000
30	573.88200000	4.57580000	0.00000000
31	609.07050000	8.88820000	0.00000000
32	635.94050000	10.96630000	0.00000000
33	638.15340000	2.60330000	0.00000000
34	683.68690000	19.74310000	0.00000000
35	711.45210000	62.24030000	0.00000000
36	737.14120000	11.85270000	0.00000000
37	753.27930000	33.40350000	0.00000000
38	762.30950000	49.46560000	0.00000000
39	775.01250000	31.52280000	0.00000000
40	790.10670000	10.05080000	0.00000000
41	825.77000000	1.45830000	0.00000000
42	837.43260000	8.88330000	0.00000000
43	854.61610000	72.37060000	0.00000000
44	857.10160000	1.31430000	0.00000000
45	873.04880000	6.85760000	0.00000000
46	877.00500000	3.21160000	0.00000000
47	932.08500000	0.06090000	0.00000000
48	933.64230000	1.06140000	0.00000000
49	934.29150000	3.51090000	0.00000000
50	945.13690000	2.51380000	0.00000000
51	973.04750000	0.12880000	0.00000000
52	985.26680000	34.29820000	0.00000000
53	986.05150000	1.06730000	0.00000000
54	987.52680000	10.11710000	0.00000000
55	989.94300000	17.39910000	0.00000000
56	1009.55900000	0.05730000	0.00000000
57	1019.09670000	1.03330000	0.00000000
58	1025.99450000	5.48480000	0.00000000
59	1044.77710000	17.97270000	0.00000000
60	1052.98870000	4.21510000	0.00000000
61	1053.40870000	13.22690000	0.00000000
62	1056.51710000	8.78670000	0.00000000

63	1070.32120000	21.14560000	0.00000000
64	1108.60060000	11.98240000	0.00000000
65	1132.71280000	53.51770000	0.00000000
66	1159.17220000	692.38550000	0.00000000
67	1174.88560000	2.30530000	0.00000000
68	1180.49460000	0.31270000	0.00000000
69	1187.91790000	4.76490000	0.00000000
70	1202.92900000	10.25410000	0.00000000
71	1215.06120000	2.68800000	0.00000000
72	1235.07120000	6.47530000	0.00000000
73	1243.78280000	133.93400000	0.00000000
74	1263.47280000	26.61880000	0.00000000
75	1271.85390000	209.18310000	0.00000000
76	1272.55870000	14.84860000	0.00000000
77	1285.92260000	37.79530000	0.00000000
78	1312.34770000	43.13480000	0.00000000
79	1332.49560000	2.23500000	0.00000000
80	1351.71660000	43.50850000	0.00000000
81	1355.85470000	12.63290000	0.00000000
82	1363.72470000	13.74710000	0.00000000
83	1397.84350000	28.82170000	0.00000000
84	1400.64820000	30.11230000	0.00000000
85	1405.20510000	46.38610000	0.00000000
86	1423.55490000	18.56080000	0.00000000
87	1468.92330000	0.40110000	0.00000000
88	1474.86240000	3.30440000	0.00000000
89	1485.32910000	3.94940000	0.00000000
90	1485.92790000	16.05480000	0.00000000
91	1486.54910000	2.83100000	0.00000000
92	1491.89920000	46.10350000	0.00000000
93	1493.33740000	6.10040000	0.00000000
94	1499.64500000	2.65690000	0.00000000
95	1511.65800000	141.50250000	0.00000000
96	1520.01520000	21.93180000	0.00000000
97	1532.88260000	16.43220000	0.00000000
98	1626.70340000	2.03250000	0.00000000
99	1631.20430000	23.56780000	0.00000000
100	1645.42350000	36.08420000	0.00000000
101	1648.63430000	3.87930000	0.00000000
102	1778.20310000	524.00990000	0.00000000
103	3029.82170000	38.06890000	0.00000000
104	3037.97710000	16.46920000	0.00000000
105	3041.79660000	22.26620000	0.00000000

106	3046.06450000	32.96440000	0.00000000
107	3049.57510000	28.49670000	0.00000000
108	3096.82860000	31.44560000	0.00000000
109	3104.06090000	14.91390000	0.00000000
110	3110.61400000	11.33890000	0.00000000
111	3112.93460000	37.75610000	0.00000000
112	3117.53990000	54.06270000	0.00000000
113	3136.54740000	12.38140000	0.00000000
114	3147.56280000	14.28260000	0.00000000
115	3164.38190000	3.96150000	0.00000000
116	3166.01600000	1.61700000	0.00000000
117	3171.77040000	13.79230000	0.00000000
118	3172.51950000	3.97810000	0.00000000
119	3181.34700000	31.23230000	0.00000000
120	3183.14110000	34.29800000	0.00000000
121	3191.64660000	40.89320000	0.00000000
122	3192.34990000	21.33230000	0.00000000
123	3202.58570000	11.33340000	0.00000000

N-Boc-2-phenylindoline Anticlockwise TS from 2a-1 to 2a-2

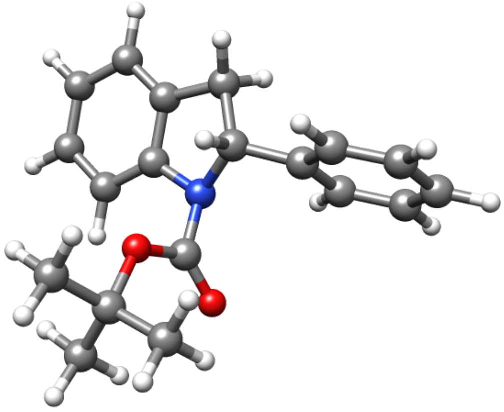

Route : # opt=qst3 freq b3lyp/6-311g(d,p) scrf=(solvent=thf)
geom=connectivity empiricaldispersion=gd3bj
int=ultrafine pop=(regular,mk)

SMILES : CC(C)(C)OC(=O)N2c1ccccc1CC2c3ccccc3

Formula : $C_{19}H_{21}NO_2$

Charge : 0

Multiplicity : 1

Dipole : 3.3472 Debye

Energy : -942.22758600 a.u.

Gibbs Energy : -941.91764200 a.u.

Number of imaginary frequencies : 1

Cartesian Coordinates (XYZ format)

43

C	-3.42196500	3.11286000	0.16442800
C	-3.66570300	1.99170700	-0.62700900
C	-2.15115100	3.32217800	0.71528000
C	-1.14843500	2.39864200	0.47267200
C	-1.40534200	1.27811100	-0.32757400
C	-2.65890300	1.05680400	-0.88618600
C	0.30311100	2.37262700	0.88367400
C	0.70078300	0.89866600	0.61551300
H	0.48059000	2.64904500	1.92329300
H	0.89647200	3.04081800	0.25031900
N	-0.23443400	0.50741900	-0.48008300
H	-4.21761500	3.82258300	0.35613900
H	-1.95770800	4.19296000	1.33196400
H	-4.65226100	1.83482400	-1.04760100
H	-2.85410500	0.18937100	-1.50507700
C	-0.35485000	-0.89757300	-0.72065600
O	0.06908600	-1.43087200	-1.71360300
O	-0.96877700	-1.49170400	0.30131000

C	-1.21149500	-2.95960900	0.33323300
C	-2.12056400	-3.34852500	-0.82906600
H	-3.03410100	-2.74988000	-0.81098200
H	-2.39879200	-4.39994900	-0.72848000
H	-1.62134300	-3.20989000	-1.78622500
C	0.12662700	-3.69278000	0.31012100
H	-0.04751300	-4.76045900	0.46138200
H	0.76915300	-3.33252400	1.11675400
H	0.63771900	-3.55297400	-0.64055900
C	-1.91957600	-3.14265100	1.67041600
H	-2.84310000	-2.56110500	1.69583500
H	-1.27808300	-2.81622100	2.49134300
H	-2.16526900	-4.19622100	1.81737900
H	0.45875600	0.30694800	1.50716800
C	2.14873600	0.68245200	0.25753000
C	2.62199000	0.94828500	-1.03032100
C	3.04530400	0.24792100	1.23519300
C	3.97143900	0.78930500	-1.32994900
C	4.39830500	0.09334900	0.93821700
C	4.86446400	0.36456800	-0.34594000
H	2.68106600	0.02775300	2.23317400
H	5.08413500	-0.24513100	1.70620000
H	5.91478300	0.23894300	-0.58174700
H	1.92187000	1.25983800	-1.79522700
H	4.32726000	0.99193200	-2.33358200

Frequencies

Mode	IR frequency	IR intensity	Raman intensity
1	-31.95910000	1.44090000	0.00000000
2	23.63620000	0.32820000	0.00000000
3	35.91530000	0.14320000	0.00000000
4	48.16560000	0.31830000	0.00000000
5	58.89940000	1.43930000	0.00000000
6	76.26050000	0.82810000	0.00000000
7	101.78560000	1.38570000	0.00000000
8	149.12920000	2.51900000	0.00000000
9	158.87710000	2.19100000	0.00000000
10	187.55420000	0.42100000	0.00000000
11	200.61330000	3.40470000	0.00000000
12	210.04110000	0.16750000	0.00000000
13	237.31140000	4.25430000	0.00000000
14	254.12730000	0.96760000	0.00000000
15	269.62300000	4.35880000	0.00000000
16	270.03160000	0.01850000	0.00000000
17	292.24380000	0.96380000	0.00000000
18	320.83170000	8.53220000	0.00000000
19	353.56690000	2.29790000	0.00000000
20	372.26620000	5.75230000	0.00000000

21	403.46970000	1.87580000	0.00000000
22	415.71110000	0.55040000	0.00000000
23	426.87430000	7.47100000	0.00000000
24	438.33810000	1.82970000	0.00000000
25	465.67280000	4.26810000	0.00000000
26	473.42040000	7.07640000	0.00000000
27	514.71920000	13.33060000	0.00000000
28	545.53070000	3.21880000	0.00000000
29	555.83130000	16.35470000	0.00000000
30	576.50210000	7.48950000	0.00000000
31	620.01900000	9.42640000	0.00000000
32	635.36230000	0.44760000	0.00000000
33	644.38930000	8.72550000	0.00000000
34	694.26780000	2.15430000	0.00000000
35	715.59920000	59.62080000	0.00000000
36	734.64900000	8.20290000	0.00000000
37	754.81300000	81.64990000	0.00000000
38	766.59710000	6.31860000	0.00000000
39	774.59530000	45.59110000	0.00000000
40	787.17630000	16.21630000	0.00000000
41	834.19970000	5.91720000	0.00000000
42	838.43180000	17.90290000	0.00000000
43	854.00520000	64.25180000	0.00000000
44	861.46440000	0.80620000	0.00000000
45	864.53300000	9.69150000	0.00000000
46	885.29530000	13.03520000	0.00000000
47	931.51080000	0.05970000	0.00000000
48	932.33430000	5.67710000	0.00000000
49	933.66250000	0.31040000	0.00000000
50	936.52740000	4.74500000	0.00000000
51	964.40960000	28.49490000	0.00000000
52	973.33990000	0.15580000	0.00000000
53	979.84930000	0.11830000	0.00000000
54	988.99780000	0.36530000	0.00000000
55	1007.51500000	10.49040000	0.00000000
56	1015.33120000	33.46330000	0.00000000
57	1020.63770000	0.33700000	0.00000000
58	1040.28170000	10.86900000	0.00000000
59	1047.35290000	24.61730000	0.00000000
60	1052.39040000	1.05960000	0.00000000
61	1052.79340000	11.72200000	0.00000000
62	1057.06100000	14.86840000	0.00000000
63	1078.28160000	50.03010000	0.00000000

64	1101.64540000	11.55430000	0.00000000
65	1136.14960000	62.42440000	0.00000000
66	1162.19530000	715.20190000	0.00000000
67	1176.11460000	4.09240000	0.00000000
68	1177.87760000	4.08870000	0.00000000
69	1185.47750000	17.16640000	0.00000000
70	1195.12640000	3.76030000	0.00000000
71	1202.21060000	4.05300000	0.00000000
72	1235.50680000	13.72000000	0.00000000
73	1246.39080000	211.00580000	0.00000000
74	1261.94860000	32.41030000	0.00000000
75	1272.58580000	27.06300000	0.00000000
76	1283.06950000	80.46960000	0.00000000
77	1293.46800000	252.16590000	0.00000000
78	1311.97910000	47.80200000	0.00000000
79	1331.38730000	9.57020000	0.00000000
80	1346.71310000	5.85900000	0.00000000
81	1350.10010000	6.41110000	0.00000000
82	1373.59250000	62.55880000	0.00000000
83	1398.05200000	28.53190000	0.00000000
84	1400.58050000	28.69970000	0.00000000
85	1404.09320000	18.83590000	0.00000000
86	1422.98710000	19.66760000	0.00000000
87	1468.83420000	0.73210000	0.00000000
88	1476.53280000	6.50870000	0.00000000
89	1484.68560000	0.59770000	0.00000000
90	1485.31560000	1.70010000	0.00000000
91	1487.91110000	22.49800000	0.00000000
92	1492.09200000	44.45260000	0.00000000
93	1493.48810000	18.06540000	0.00000000
94	1498.78220000	2.31590000	0.00000000
95	1512.66410000	158.53840000	0.00000000
96	1518.74280000	21.69570000	0.00000000
97	1528.22620000	12.12240000	0.00000000
98	1628.21010000	0.80820000	0.00000000
99	1633.41260000	14.11210000	0.00000000
100	1647.57690000	80.29600000	0.00000000
101	1648.30730000	0.86020000	0.00000000
102	1785.33470000	526.47920000	0.00000000
103	3002.30180000	37.29540000	0.00000000
104	3029.54400000	40.94060000	0.00000000
105	3039.00470000	13.83590000	0.00000000
106	3041.70110000	33.06120000	0.00000000

107	3047.77740000	15.06710000	0.00000000
108	3102.01310000	22.02380000	0.00000000
109	3104.44450000	7.93040000	0.00000000
110	3106.45830000	21.42930000	0.00000000
111	3112.57540000	44.51840000	0.00000000
112	3116.98990000	60.31780000	0.00000000
113	3140.44780000	0.82690000	0.00000000
114	3143.34050000	25.79920000	0.00000000
115	3159.38000000	8.81290000	0.00000000
116	3164.96770000	4.37290000	0.00000000
117	3167.52870000	1.05680000	0.00000000
118	3171.57460000	13.70060000	0.00000000
119	3177.19260000	33.41450000	0.00000000
120	3183.01570000	37.28120000	0.00000000
121	3188.27710000	40.40850000	0.00000000
122	3192.51980000	27.00260000	0.00000000
123	3195.98170000	9.52050000	0.00000000

Thermochemistry Data for *N*-Boc-2-phenylindoline Calculations

N-Boc-2-phenylindoline Rotamer **2a-1**:

	Temperature		
	298 K	223 K	195 K
ε_0	–942.251145874 a.u.	–942.251145874 a.u.	–942.251145874 a.u.
ε_{ZPE}	0.359327 a.u.	0.359327 a.u.	0.359327 a.u.
E_{tot}	0.379140 a.u.	0.370947 a.u.	0.368473 a.u.
H_{corr}	0.380084 a.u.	0.371653 a.u.	0.369091 a.u.
G_{corr}	0.309805 a.u.	0.326306 a.u.	0.331829 a.u.
S_{tot}	147.914 calmol^{-1}K^{-1}	127.603 calmol^{-1}K^{-1}	119.908 calmol^{-1}K^{-1}
$\varepsilon_0 + \varepsilon_{ZPE}$	–941.891819 a.u.	–941.891819 a.u.	–941.891819 a.u.
$\varepsilon_0 + E_{tot}$	–941.872006 a.u.	–941.880199 a.u.	–941.882673 a.u.
$\varepsilon_0 + H_{corr}$	–941.871062 a.u.	–941.879493 a.u.	–941.882055 a.u.
$\varepsilon_0 + G_{corr}$	–941.941341 a.u.	–941.924840 a.u.	–941.919317 a.u.

N-Boc-2-phenylindoline Rotamer **2a-2**:

	Temperature		
	298 K	223 K	195 K
ε_0	–942.248344872 a.u.	–942.248344872 a.u.	–942.248344872 a.u.
ε_{ZPE}	0.359093 a.u.	0.359093 a.u.	0.359093 a.u.
E_{tot}	0.378110 a.u.	0.370145 a.u.	0.367756 a.u.
H_{corr}	0.379054 a.u.	0.370851 a.u.	0.368373 a.u.
G_{corr}	0.310573 a.u.	0.326653 a.u.	0.332037 a.u.
S_{tot}	144.129 calmol^{-1}K^{-1}	124.372 calmol^{-1}K^{-1}	116.931 calmol^{-1}K^{-1}
$\varepsilon_0 + \varepsilon_{ZPE}$	–941.889251 a.u.	–941.889252 a.u.	–941.889252 a.u.
$\varepsilon_0 + E_{tot}$	–941.870235 a.u.	–941.878200 a.u.	–941.880589 a.u.
$\varepsilon_0 + H_{corr}$	–941.869291 a.u.	–941.877494 a.u.	–941.879972 a.u.
$\varepsilon_0 + G_{corr}$	–941.937771 a.u.	–941.921692 a.u.	–941.916308 a.u.

TS for *N*-Boc-2-phenylindoline Clockwise Rotation of Boc Group from Rotamer **2a-1** to Rotamer **2a-2**:

	Temperature	
	298 K	195 K
ε_0	–942.222362891 a.u.	–942.222362891 a.u.
ε_{ZPE}	0.358533 a.u.	0.358533 a.u.
E_{tot}	0.376619 a.u.	0.366570 a.u.
H_{corr}	0.377564 a.u.	0.367188 a.u.
G_{corr}	0.313323 a.u.	0.333379 a.u.
S_{tot}	135.207 calmol^{-1}K^{-1}	108.797 calmol^{-1}K^{-1}
$\varepsilon_0 + \varepsilon_{ZPE}$	–941.863830 a.u.	–941.863830 a.u.
$\varepsilon_0 + E_{tot}$	–941.845743 a.u.	–941.855793 a.u.
$\varepsilon_0 + H_{corr}$	–941.844799 a.u.	–941.855175 a.u.
$\varepsilon_0 + G_{corr}$	–941.909040 a.u.	–941.888984 a.u.

TS for *N*-Boc-2-phenylindoline Anticlockwise Rotation of Boc Group from Rotamer **2a-1** to Rotamer **2a-2**:

	Temperature	
	298 K	195 K
ε_0	–942.227585996 a.u.	–942.227585996 a.u.
ε_{ZPE}	0.358382 a.u.	0.358382 a.u.
E_{tot}	0.377485 a.u.	0.367106 a.u.
H_{corr}	0.378429 a.u.	0.367723 a.u.
G_{corr}	0.309944 a.u.	0.331404 a.u.
S_{tot}	144.139 calmol^{-1}K^{-1}	116.876 calmol^{-1}K^{-1}
$\varepsilon_0 + \varepsilon_{ZPE}$	–941.869204 a.u.	–941.869204 a.u.
$\varepsilon_0 + E_{tot}$	–941.850101 a.u.	–941.860480 a.u.
$\varepsilon_0 + H_{corr}$	–941.849157 a.u.	–941.859863 a.u.
$\varepsilon_0 + G_{corr}$	–941.917642 a.u.	–941.896182 a.u.

N-Boc-2-(*p*-methoxyphenyl)indoline 2d-1

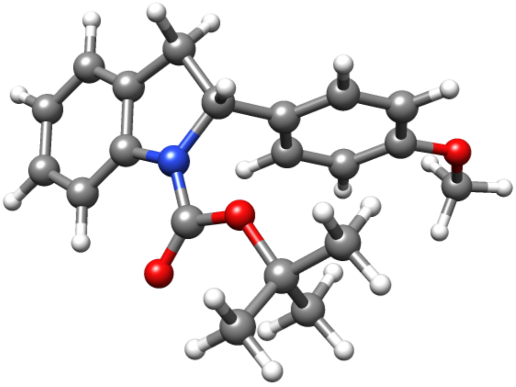

Route : # opt freq b3lyp/6-311g(d,p) scrf=(solvent=thf)
geom=connectivity empiricaldispersion=gd3bj
int=ultrafine pop=(regular,mk)

SMILES : COc3ccc(C2Cc1ccccc1N2C(=O)OC(C)(C)C)cc3

Formula : $C_{20}H_{23}NO_3$

Charge : 0

Multiplicity : 1

Dipole : 3.4256 Debye

Energy : -1056.81277556 a.u.

Gibbs Energy : -1056.47430600 a.u.

Number of imaginary frequencies : 0

Cartesian Coordinates (XYZ format)

47

C	2.79152100	-1.30032600	0.73762000
C	2.57960500	-0.16990100	-0.06107500
C	1.77052200	-1.34712400	1.84557300
C	0.73433700	-0.25640400	1.44927000
H	2.23255100	-1.10524500	2.80706300
H	1.29393100	-2.32304100	1.94391300
N	1.44735000	0.52835500	0.40323100
H	0.51735600	0.39653500	2.29186500
C	-0.56677500	-0.82047700	0.91930600
C	3.84049300	-2.16201500	0.46262100
C	4.68836700	-1.88553400	-0.61505700
C	4.47300200	-0.75294600	-1.39777200
C	3.41572800	0.12292600	-1.13621500
H	4.00207100	-3.03971000	1.07851100
H	5.51173600	-2.55253300	-0.84075300
H	5.13235200	-0.54348100	-2.23222800
H	3.23802900	0.99457800	-1.74583900
C	0.90978500	1.64903800	-0.17471000
O	-0.15780300	2.05272200	0.53752400

O	1.35093900	2.19250700	-1.17106300
C	-1.16434500	2.96528200	-0.03953400
C	-2.24258300	2.98932900	1.03964900
H	-2.64086100	1.98612100	1.20059600
H	-3.05778900	3.64853600	0.73423800
H	-1.83077900	3.35843400	1.98136000
C	-0.55984100	4.35282500	-0.24237500
H	-0.12649400	4.71378300	0.69341800
H	-1.34498300	5.04911800	-0.54747900
H	0.21476300	4.33268800	-1.00598000
C	-1.70809500	2.36256000	-1.33397400
H	-2.06988000	1.34814900	-1.15155900
H	-0.94083200	2.33252700	-2.10636800
H	-2.54178800	2.96981300	-1.69373100
C	-0.65769300	-1.35316200	-0.36454400
C	-1.71227400	-0.81876400	1.71919700
C	-1.85705800	-1.87113700	-0.85184500
C	-2.99499800	-1.86201200	-0.04028200
C	-2.91316300	-1.33267100	1.25294200
H	0.21150900	-1.35444200	-1.01179100
H	-1.89178100	-2.26933600	-1.85609100
O	-4.21600500	-2.33565700	-0.41562600
H	-1.66722500	-0.39374300	2.71601900
H	-3.80499200	-1.32390000	1.86751900
C	-4.35312400	-2.87059600	-1.72996500
H	-5.39234300	-3.18027700	-1.81765900
H	-3.70125600	-3.73744900	-1.87639000
H	-4.13225400	-2.11554600	-2.49091500

Frequencies

Mode	IR frequency	IR intensity	Raman intensity
1	23.07350000	0.14690000	0.00000000
2	25.11850000	0.20020000	0.00000000
3	34.49960000	0.50460000	0.00000000
4	42.76280000	1.52780000	0.00000000
5	56.19720000	4.32450000	0.00000000
6	83.46890000	2.24860000	0.00000000
7	87.50260000	1.17110000	0.00000000
8	107.41160000	2.60840000	0.00000000
9	135.81280000	0.34530000	0.00000000
10	150.47700000	0.87950000	0.00000000
11	170.22790000	1.61520000	0.00000000
12	209.44680000	0.12700000	0.00000000
13	212.39030000	2.22850000	0.00000000
14	224.49000000	6.73570000	0.00000000
15	235.18990000	3.31450000	0.00000000
16	248.26570000	3.39630000	0.00000000
17	256.50980000	1.58260000	0.00000000
18	267.62130000	2.96170000	0.00000000

19	275.79130000	0.38010000	0.00000000
20	291.99240000	1.05010000	0.00000000
21	322.47420000	0.35550000	0.00000000
22	330.38740000	16.17690000	0.00000000
23	353.93640000	3.59430000	0.00000000
24	383.20170000	0.68520000	0.00000000
25	407.71590000	4.86220000	0.00000000
26	423.77070000	0.35090000	0.00000000
27	431.92670000	4.66260000	0.00000000
28	441.87900000	1.17800000	0.00000000
29	461.34470000	7.45300000	0.00000000
30	471.74290000	2.86950000	0.00000000
31	476.88150000	0.89240000	0.00000000
32	515.27260000	1.52380000	0.00000000
33	536.74430000	16.89100000	0.00000000
34	561.11290000	10.88700000	0.00000000
35	569.21500000	20.56620000	0.00000000
36	622.33620000	21.38660000	0.00000000
37	632.87240000	12.12250000	0.00000000
38	650.56150000	0.86220000	0.00000000
39	715.86070000	2.98570000	0.00000000
40	744.17260000	22.48970000	0.00000000
41	747.82200000	19.60450000	0.00000000
42	752.02320000	18.06180000	0.00000000
43	763.91470000	55.25150000	0.00000000
44	773.94820000	30.91300000	0.00000000
45	790.15850000	14.92930000	0.00000000
46	820.10950000	9.86980000	0.00000000
47	826.61200000	0.87170000	0.00000000
48	834.60160000	11.04510000	0.00000000
49	846.31880000	73.24500000	0.00000000
50	855.45400000	59.80630000	0.00000000
51	869.57200000	7.72690000	0.00000000
52	879.99410000	5.07940000	0.00000000
53	929.63970000	0.44410000	0.00000000
54	931.35890000	0.17410000	0.00000000
55	945.29960000	0.99210000	0.00000000
56	953.87790000	0.04650000	0.00000000
57	971.62690000	0.28900000	0.00000000
58	972.85410000	0.12740000	0.00000000
59	983.43080000	11.67440000	0.00000000
60	991.02560000	0.39810000	0.00000000
61	1023.85810000	3.12930000	0.00000000

62	1029.34100000	8.92070000	0.00000000
63	1036.78510000	159.49990000	0.00000000
64	1049.45560000	17.35030000	0.00000000
65	1053.01330000	2.08920000	0.00000000
66	1054.21810000	78.85640000	0.00000000
67	1061.86430000	24.55100000	0.00000000
68	1106.62220000	23.63690000	0.00000000
69	1133.84340000	65.92270000	0.00000000
70	1154.55240000	298.98620000	0.00000000
71	1170.99230000	1.08370000	0.00000000
72	1174.81560000	7.22730000	0.00000000
73	1178.82090000	49.35830000	0.00000000
74	1187.05270000	296.54080000	0.00000000
75	1199.57160000	23.12220000	0.00000000
76	1200.08780000	96.32180000	0.00000000
77	1204.17530000	31.66000000	0.00000000
78	1232.91700000	12.38680000	0.00000000
79	1245.27060000	14.07100000	0.00000000
80	1269.37010000	51.80370000	0.00000000
81	1275.09300000	305.11960000	0.00000000
82	1276.32220000	116.77340000	0.00000000
83	1286.14190000	42.93430000	0.00000000
84	1304.59420000	182.12430000	0.00000000
85	1321.54340000	77.61120000	0.00000000
86	1335.10800000	65.07790000	0.00000000
87	1336.88950000	19.25990000	0.00000000
88	1352.34600000	99.27720000	0.00000000
89	1385.96550000	94.62110000	0.00000000
90	1395.91690000	30.12630000	0.00000000
91	1399.12860000	30.93570000	0.00000000
92	1415.30340000	657.23290000	0.00000000
93	1421.59020000	20.00120000	0.00000000
94	1457.17820000	10.07060000	0.00000000
95	1467.32370000	0.94580000	0.00000000
96	1477.41680000	15.95560000	0.00000000
97	1480.90650000	3.88870000	0.00000000
98	1485.30380000	1.74910000	0.00000000
99	1485.84510000	11.48050000	0.00000000
100	1487.65920000	4.36580000	0.00000000
101	1490.13250000	17.92350000	0.00000000
102	1494.90790000	39.42800000	0.00000000
103	1499.21700000	2.90610000	0.00000000
104	1500.16140000	49.26200000	0.00000000

105	1514.11040000	223.79880000	0.00000000
106	1520.26150000	62.64080000	0.00000000
107	1546.76150000	172.78570000	0.00000000
108	1621.93750000	24.62030000	0.00000000
109	1634.45780000	1.41220000	0.00000000
110	1644.02280000	41.48910000	0.00000000
111	1655.54280000	76.81250000	0.00000000
112	1734.19590000	571.56040000	0.00000000
113	3012.08150000	64.80150000	0.00000000
114	3036.88140000	13.28400000	0.00000000
115	3038.56140000	34.76350000	0.00000000
116	3040.32070000	40.63050000	0.00000000
117	3045.17820000	25.77310000	0.00000000
118	3072.96660000	49.83960000	0.00000000
119	3094.49200000	17.10180000	0.00000000
120	3099.59290000	16.63080000	0.00000000
121	3105.93560000	12.76480000	0.00000000
122	3109.57680000	22.49340000	0.00000000
123	3110.50900000	64.39110000	0.00000000
124	3118.00020000	47.84160000	0.00000000
125	3133.93830000	12.83500000	0.00000000
126	3141.46120000	31.26130000	0.00000000
127	3146.54550000	16.72260000	0.00000000
128	3164.77430000	16.83270000	0.00000000
129	3165.75020000	1.32370000	0.00000000
130	3173.77280000	27.48900000	0.00000000
131	3177.35640000	8.94900000	0.00000000
132	3189.63890000	41.04860000	0.00000000
133	3190.56940000	11.27620000	0.00000000
134	3211.35910000	8.31930000	0.00000000
135	3242.23600000	1.74490000	0.00000000

N-Boc-2-(*p*-methoxyphenyl)indoline 2d-2

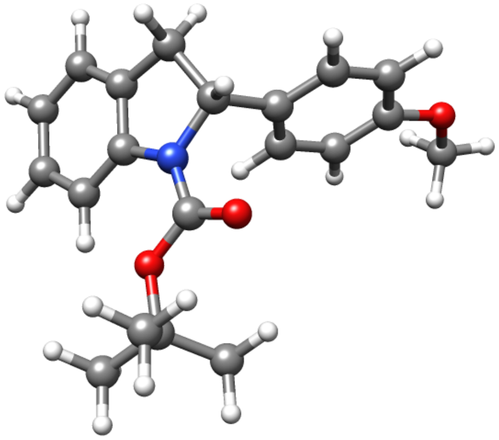

Route : # opt freq b3lyp/6-311g(d,p) scrf=(solvent=thf)
geom=connectivity empiricaldispersion=gd3bj
int=ultrafine pop=(regular,mk)

SMILES : COc3ccc(C2Cc1ccccc1N2C(=O)OC(C)(C)C)cc3

Formula : $C_{20}H_{23}NO_3$

Charge : 0

Multiplicity : 1

Dipole : 2.7913 Debye

Energy : -1056.80977169 a.u.

Gibbs Energy : - 1056.47029200 a.u.

Number of imaginary frequencies : 0

Cartesian Coordinates (XYZ format)

47

C	-0.58610600	2.69462100	0.50250800
C	-1.28038600	1.57554300	0.02082500
C	0.30587200	2.29151800	1.64865600
C	0.33395300	0.74120000	1.55739400
H	-0.12561600	2.61325500	2.60156200
H	1.31021400	2.70944700	1.57891700
N	-0.85633500	0.43007500	0.73100900
H	0.20594100	0.28268500	2.53591700
C	1.60881400	0.21472700	0.93006100
C	-0.79632700	3.94440100	-0.05535100
C	-1.71349800	4.08177800	-1.10223400
C	-2.40318800	2.96606100	-1.57047800
C	-2.19753200	1.69741700	-1.02084700
H	-0.25489500	4.80618100	0.31867700
H	-1.88584000	5.05391300	-1.54819600
H	-3.11228000	3.07440500	-2.38313100
H	-2.72960900	0.83793700	-1.39355600

C	-1.33518800	-0.85532900	0.72569700
O	-2.37408400	-0.99249200	-0.11261900
O	-0.85050100	-1.73308100	1.41663100
C	-3.05548800	-2.29415400	-0.28265900
C	-4.13723100	-1.96536000	-1.30745900
H	-4.80169300	-1.18732000	-0.92628000
H	-4.73003400	-2.85739900	-1.51885800
H	-3.68882000	-1.61720700	-2.24031500
C	-2.07276600	-3.32215700	-0.83947800
H	-1.60663900	-2.94073400	-1.75102700
H	-2.61233600	-4.23907200	-1.08805000
H	-1.29594700	-3.55488700	-0.11415200
C	-3.67689000	-2.73159400	1.04204800
H	-4.32213100	-1.94167600	1.43367600
H	-2.90907600	-2.96159700	1.77778000
H	-4.28755000	-3.62240000	0.87688700
C	1.77051800	0.13034400	-0.45015600
C	2.68456900	-0.14825400	1.74698800
C	2.97076000	-0.30163500	-1.01549400
C	4.03782200	-0.65610400	-0.18684900
C	3.88515700	-0.57727700	1.20264600
H	0.95087900	0.39504800	-1.10698200
H	3.05729800	-0.35937200	-2.09143500
O	5.25079800	-1.09218000	-0.63020200
H	2.57751600	-0.10134600	2.82560300
H	4.71682900	-0.86691300	1.83324700
C	5.45094300	-1.20025500	-2.03713800
H	6.46919300	-1.56130100	-2.16571300
H	5.34416700	-0.22878400	-2.52997200
H	4.75222400	-1.91431900	-2.48422000

Frequencies

Mode	IR frequency	IR intensity	Raman intensity
1	18.59240000	0.14230000	0.00000000
2	19.31860000	0.89470000	0.00000000
3	31.23420000	0.75850000	0.00000000
4	35.65130000	0.01070000	0.00000000
5	60.46150000	2.12340000	0.00000000
6	81.02520000	2.18070000	0.00000000
7	93.64190000	0.86450000	0.00000000
8	98.87460000	3.09680000	0.00000000
9	125.71150000	0.87770000	0.00000000
10	142.98020000	0.95650000	0.00000000
11	174.40020000	2.47130000	0.00000000
12	209.67610000	0.95060000	0.00000000
13	213.52670000	2.84780000	0.00000000
14	225.29810000	4.35520000	0.00000000
15	234.20200000	2.47150000	0.00000000
16	248.98350000	0.26030000	0.00000000

17	254.10260000	1.71070000	0.00000000
18	274.19460000	2.01980000	0.00000000
19	276.11280000	1.90410000	0.00000000
20	301.04380000	3.84440000	0.00000000
21	326.77390000	5.59880000	0.00000000
22	335.75110000	5.31760000	0.00000000
23	352.36140000	2.48400000	0.00000000
24	361.59380000	11.96350000	0.00000000
25	414.86660000	2.31310000	0.00000000
26	424.04720000	0.78350000	0.00000000
27	430.33460000	3.51450000	0.00000000
28	442.49500000	9.41130000	0.00000000
29	458.53230000	4.81550000	0.00000000
30	471.89090000	4.71010000	0.00000000
31	479.73000000	1.75280000	0.00000000
32	508.44920000	8.42530000	0.00000000
33	536.56140000	15.51210000	0.00000000
34	560.72440000	10.81360000	0.00000000
35	565.83360000	21.14490000	0.00000000
36	617.91640000	11.11920000	0.00000000
37	648.71770000	3.39530000	0.00000000
38	665.66300000	19.67350000	0.00000000
39	712.77090000	2.90370000	0.00000000
40	731.33430000	3.52550000	0.00000000
41	742.42010000	20.02010000	0.00000000
42	754.38420000	46.34050000	0.00000000
43	763.99980000	48.91490000	0.00000000
44	769.49300000	21.27360000	0.00000000
45	792.37450000	4.13230000	0.00000000
46	822.10570000	3.94700000	0.00000000
47	825.10350000	1.53250000	0.00000000
48	835.35770000	9.95960000	0.00000000
49	844.54810000	59.69090000	0.00000000
50	851.43690000	12.26330000	0.00000000
51	873.13690000	34.59300000	0.00000000
52	877.67940000	52.32010000	0.00000000
53	929.04410000	0.07980000	0.00000000
54	931.69540000	0.51350000	0.00000000
55	943.21310000	0.34720000	0.00000000
56	952.46950000	0.35940000	0.00000000
57	969.67540000	0.03860000	0.00000000
58	971.82390000	0.15540000	0.00000000
59	980.85050000	14.94140000	0.00000000

60	987.36350000	0.07760000	0.00000000
61	1023.36670000	28.95470000	0.00000000
62	1028.78940000	8.58660000	0.00000000
63	1041.58860000	70.27320000	0.00000000
64	1052.42680000	1.15730000	0.00000000
65	1053.08410000	8.76000000	0.00000000
66	1054.78530000	76.27650000	0.00000000
67	1065.57520000	53.03310000	0.00000000
68	1107.16990000	26.68470000	0.00000000
69	1134.29790000	32.80620000	0.00000000
70	1148.46830000	216.35050000	0.00000000
71	1171.55970000	1.00580000	0.00000000
72	1177.69340000	84.67350000	0.00000000
73	1178.18030000	7.75410000	0.00000000
74	1187.75080000	230.27120000	0.00000000
75	1194.73730000	104.10000000	0.00000000
76	1198.93510000	65.03400000	0.00000000
77	1204.15960000	23.12780000	0.00000000
78	1231.29030000	6.72530000	0.00000000
79	1241.20240000	41.08380000	0.00000000
80	1269.65980000	27.59480000	0.00000000
81	1273.70910000	347.24640000	0.00000000
82	1279.47250000	83.56620000	0.00000000
83	1291.46810000	77.35840000	0.00000000
84	1300.24460000	148.60340000	0.00000000
85	1323.68390000	68.70310000	0.00000000
86	1336.47460000	22.03120000	0.00000000
87	1343.68180000	44.34020000	0.00000000
88	1362.49530000	184.22810000	0.00000000
89	1385.36350000	243.97760000	0.00000000
90	1395.38760000	30.39120000	0.00000000
91	1398.83960000	56.21370000	0.00000000
92	1409.16530000	378.08630000	0.00000000
93	1421.55810000	17.28620000	0.00000000
94	1456.32700000	6.26750000	0.00000000
95	1467.34320000	0.35020000	0.00000000
96	1477.52030000	16.22670000	0.00000000
97	1479.48820000	11.08990000	0.00000000
98	1485.08070000	1.94180000	0.00000000
99	1485.65950000	11.27640000	0.00000000
100	1487.06570000	5.38820000	0.00000000
101	1489.02530000	46.77750000	0.00000000
102	1495.95510000	40.61400000	0.00000000

103	1499.28590000	2.30440000	0.00000000
104	1499.87300000	48.21120000	0.00000000
105	1513.20020000	202.08520000	0.00000000
106	1519.95890000	79.24830000	0.00000000
107	1546.29930000	168.90510000	0.00000000
108	1621.31990000	22.48580000	0.00000000
109	1633.03270000	10.95740000	0.00000000
110	1646.50890000	65.34240000	0.00000000
111	1655.87910000	76.10060000	0.00000000
112	1727.45940000	774.87110000	0.00000000
113	3010.93210000	67.13930000	0.00000000
114	3034.98480000	42.21540000	0.00000000
115	3036.88230000	15.05340000	0.00000000
116	3039.09010000	36.58870000	0.00000000
117	3045.61150000	19.55890000	0.00000000
118	3071.43980000	50.36500000	0.00000000
119	3100.16390000	18.33650000	0.00000000
120	3101.17550000	7.26860000	0.00000000
121	3102.66240000	27.88490000	0.00000000
122	3108.78630000	19.70360000	0.00000000
123	3109.36200000	53.14380000	0.00000000
124	3115.45460000	70.58790000	0.00000000
125	3140.54430000	32.02960000	0.00000000
126	3143.25070000	0.61230000	0.00000000
127	3146.12480000	24.54460000	0.00000000
128	3162.34210000	17.92930000	0.00000000
129	3166.16430000	1.32190000	0.00000000
130	3173.87750000	27.04780000	0.00000000
131	3182.40410000	11.30290000	0.00000000
132	3190.18830000	0.37240000	0.00000000
133	3190.28100000	10.64540000	0.00000000
134	3210.67070000	9.19070000	0.00000000
135	3254.91230000	2.23620000	0.00000000

N-Boc-2-(*p*-methoxyphenyl)indoline Clockwise TS from 2d-1 to 2d-2

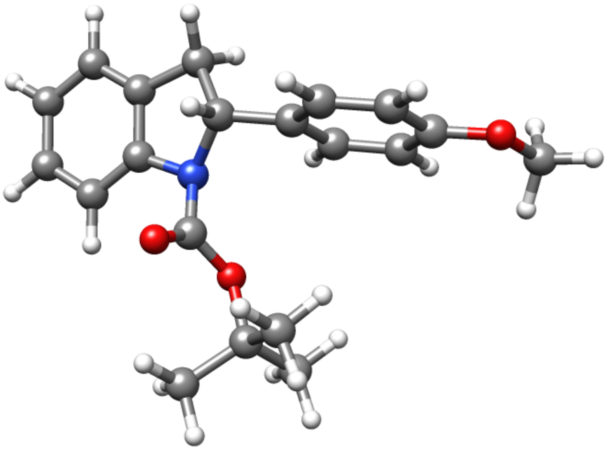

Route : # opt=qst3 freq b3lyp/6-311g(d,p) scrf=(solvent=thf)
geom=connectivity empiricaldispersion=gd3bj
int=ultrafine pop=(regular,mk)

SMILES : COc3ccc(C2Cc1ccccc1N2C(=O)OC(C)(C)C)cc3

Formula : $C_{20}H_{23}NO_3$

Charge : 0

Multiplicity : 1

Dipole : 4.0097 Debye

Energy : -1056.78980787 a.u.

Gibbs Energy : -1056.45203500 a.u.

Number of imaginary frequencies : 1

Cartesian Coordinates (XYZ format)

47

C	2.73389200	-1.93314100	-0.06724400
C	2.59904300	-0.54303900	-0.19730100
C	1.37620000	-2.51951800	0.23899300
C	0.58517500	-1.28322200	0.73017400
H	1.38814700	-3.30956400	0.99028600
H	0.91597900	-2.92342600	-0.66941000
N	1.25662100	-0.16979000	-0.00443700
H	0.77686800	-1.15234400	1.80310900
C	-0.90227500	-1.29166600	0.49532200
C	3.96606400	-2.53090200	-0.26674100
C	5.07254300	-1.73087900	-0.58295600
C	4.92633700	-0.35024300	-0.70240700
C	3.68507000	0.26560000	-0.51164100
H	4.07669600	-3.60540800	-0.17007000
H	6.04434700	-2.18644700	-0.72997400
H	5.78795300	0.26261300	-0.94108200

H	3.57903900	1.33993300	-0.60352900
C	0.95002000	1.13134800	0.50615400
O	0.29066000	1.82018900	-0.41451900
O	1.23141100	1.49672500	1.62194500
C	-0.29968900	3.15285900	-0.11445400
C	-0.98428300	3.50495800	-1.43037600
H	-0.25493200	3.54762700	-2.24163700
H	-1.46992200	4.47901800	-1.34559500
H	-1.73989800	2.75706600	-1.67809600
C	-1.31993700	3.00426900	1.01170200
H	-2.04207000	2.22276000	0.76456700
H	-1.85878400	3.94667100	1.13218000
H	-0.83720000	2.75588900	1.95507500
C	0.81042100	4.14681800	0.21474800
H	1.55107400	4.16574200	-0.58810500
H	1.30467200	3.89226600	1.15030900
H	0.37964500	5.14656500	0.30551400
C	-1.43119700	-1.15211400	-0.78728100
C	-1.78864400	-1.42677600	1.56760800
C	-2.80600900	-1.15418600	-1.00860000
C	-3.68086400	-1.29914300	0.07396400
C	-3.16163200	-1.43566600	1.36592900
H	-0.75899900	-1.01623500	-1.62564800
H	-3.18098200	-1.03745000	-2.01567400
O	-5.03866800	-1.31250700	-0.02840900
H	-1.39796400	-1.51941600	2.57521400
H	-3.85126300	-1.53810700	2.19470100
C	-5.61732000	-1.16812800	-1.32348800
H	-6.69412800	-1.20549200	-1.17315000
H	-5.31372900	-1.98335300	-1.98745100
H	-5.34562500	-0.20903400	-1.77524600

Frequencies

Mode	IR frequency	IR intensity	Raman intensity
1	-41.27260000	1.76100000	0.00000000
2	16.18630000	0.85630000	0.00000000
3	20.28350000	0.22540000	0.00000000
4	40.85910000	0.06450000	0.00000000
5	47.56630000	0.89650000	0.00000000
6	65.03070000	2.55970000	0.00000000
7	93.01110000	2.24540000	0.00000000
8	105.68140000	1.02940000	0.00000000
9	137.91210000	4.81000000	0.00000000
10	148.59830000	2.01230000	0.00000000
11	158.69330000	1.00400000	0.00000000
12	174.85020000	0.58080000	0.00000000
13	211.22560000	0.06910000	0.00000000
14	225.29760000	6.36920000	0.00000000
15	240.03660000	1.15570000	0.00000000

16	248.52820000	2.76330000	0.00000000
17	254.34970000	0.78410000	0.00000000
18	266.35520000	2.72690000	0.00000000
19	272.88740000	2.01540000	0.00000000
20	274.87130000	1.04460000	0.00000000
21	304.75840000	0.66360000	0.00000000
22	322.44570000	8.50820000	0.00000000
23	356.32970000	3.03010000	0.00000000
24	365.74260000	0.44030000	0.00000000
25	420.77600000	4.79460000	0.00000000
26	426.16900000	0.61730000	0.00000000
27	427.93640000	4.44020000	0.00000000
28	460.03690000	2.16850000	0.00000000
29	469.82580000	5.23900000	0.00000000
30	474.47800000	2.33310000	0.00000000
31	488.60640000	5.21640000	0.00000000
32	530.73910000	5.50690000	0.00000000
33	538.53080000	13.91350000	0.00000000
34	557.63190000	29.20540000	0.00000000
35	563.70100000	31.37460000	0.00000000
36	609.15290000	2.38630000	0.00000000
37	618.18370000	27.35320000	0.00000000
38	651.89480000	6.39530000	0.00000000
39	708.57960000	15.77920000	0.00000000
40	738.30670000	33.66800000	0.00000000
41	742.38720000	4.94450000	0.00000000
42	748.73590000	60.10270000	0.00000000
43	754.94820000	7.98190000	0.00000000
44	772.04940000	19.49140000	0.00000000
45	805.43650000	23.61860000	0.00000000
46	826.54480000	3.33400000	0.00000000
47	831.23520000	5.49460000	0.00000000
48	842.09450000	12.56560000	0.00000000
49	844.39030000	83.03830000	0.00000000
50	854.32110000	33.15220000	0.00000000
51	859.92100000	18.81310000	0.00000000
52	888.05850000	2.43640000	0.00000000
53	931.84140000	5.26080000	0.00000000
54	932.70180000	0.60740000	0.00000000
55	934.37600000	0.10280000	0.00000000
56	956.31660000	0.25400000	0.00000000
57	966.09860000	40.15610000	0.00000000
58	974.28850000	0.13970000	0.00000000

59	974.57810000	1.06960000	0.00000000
60	977.57740000	1.13140000	0.00000000
61	1016.46150000	34.41370000	0.00000000
62	1028.35630000	0.43400000	0.00000000
63	1041.66980000	11.07310000	0.00000000
64	1047.47040000	30.91920000	0.00000000
65	1053.66230000	2.46430000	0.00000000
66	1055.67240000	77.64390000	0.00000000
67	1058.65300000	21.88520000	0.00000000
68	1079.24710000	50.80030000	0.00000000
69	1129.91620000	21.76000000	0.00000000
70	1139.67780000	22.73470000	0.00000000
71	1170.70660000	0.47160000	0.00000000
72	1172.45370000	644.67600000	0.00000000
73	1175.97930000	16.43310000	0.00000000
74	1184.91430000	56.70890000	0.00000000
75	1193.02860000	107.24010000	0.00000000
76	1201.94690000	2.65310000	0.00000000
77	1204.39600000	20.79120000	0.00000000
78	1239.10880000	6.53350000	0.00000000
79	1258.07530000	74.19860000	0.00000000
80	1269.36640000	262.95250000	0.00000000
81	1273.18400000	18.92440000	0.00000000
82	1276.18430000	494.15020000	0.00000000
83	1282.68100000	129.72490000	0.00000000
84	1292.46050000	301.00460000	0.00000000
85	1312.49290000	126.64830000	0.00000000
86	1332.16390000	3.66090000	0.00000000
87	1336.41860000	87.43320000	0.00000000
88	1344.48620000	12.41930000	0.00000000
89	1377.66420000	87.13400000	0.00000000
90	1398.73670000	29.19340000	0.00000000
91	1400.89460000	24.51980000	0.00000000
92	1402.27730000	17.35390000	0.00000000
93	1424.66280000	19.41880000	0.00000000
94	1458.53580000	13.05070000	0.00000000
95	1468.72620000	0.39900000	0.00000000
96	1477.57210000	6.90460000	0.00000000
97	1477.90130000	11.84790000	0.00000000
98	1485.46420000	1.84400000	0.00000000
99	1485.87760000	0.14650000	0.00000000
100	1486.30980000	11.06860000	0.00000000
101	1491.55900000	30.19200000	0.00000000

102	1493.04670000	22.37880000	0.00000000
103	1499.55910000	1.06050000	0.00000000
104	1500.13950000	51.65170000	0.00000000
105	1511.65390000	156.13010000	0.00000000
106	1519.65040000	24.26500000	0.00000000
107	1546.54940000	165.54180000	0.00000000
108	1620.65970000	16.95460000	0.00000000
109	1632.46890000	2.07680000	0.00000000
110	1647.72680000	66.57820000	0.00000000
111	1655.05940000	94.25220000	0.00000000
112	1767.53590000	396.58960000	0.00000000
113	2994.20540000	45.10270000	0.00000000
114	3012.50700000	66.63010000	0.00000000
115	3027.59620000	40.54810000	0.00000000
116	3039.13580000	13.45570000	0.00000000
117	3041.14950000	31.83100000	0.00000000
118	3047.41320000	17.00320000	0.00000000
119	3073.73190000	50.19590000	0.00000000
120	3101.24470000	24.73350000	0.00000000
121	3104.04520000	11.76630000	0.00000000
122	3106.31340000	16.57360000	0.00000000
123	3112.96890000	44.13580000	0.00000000
124	3117.54260000	59.33760000	0.00000000
125	3140.13750000	2.53340000	0.00000000
126	3141.83160000	31.54540000	0.00000000
127	3143.52340000	25.32970000	0.00000000
128	3163.94510000	5.15290000	0.00000000
129	3165.74640000	15.31570000	0.00000000
130	3170.54150000	14.62920000	0.00000000
131	3182.08950000	37.52900000	0.00000000
132	3184.99130000	12.12930000	0.00000000
133	3191.74880000	36.38520000	0.00000000
134	3191.89630000	4.28100000	0.00000000
135	3211.15600000	8.71500000	0.00000000

_N_-Boc-2-(_p_-methoxyphenyl)indoline Anticlockwise TS from 2d-1 to 2d-2

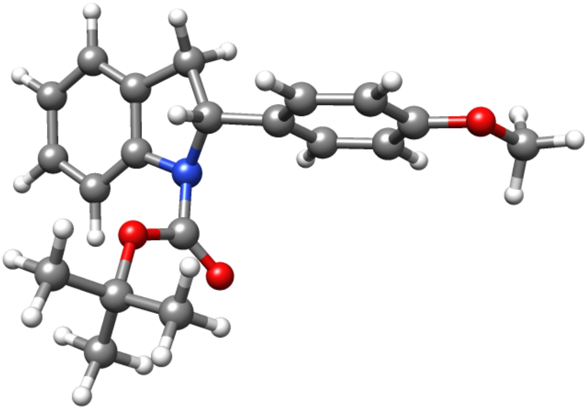

```
Route : # opt=qst3 freq b3lyp/6-311g(d,p) scrf=(solvent=thf)
          geom=connectivity empiricaldispersion=gd3bj
          int=ultrafine pop=(regular,mk)
```

SMILES : COc3ccc(C2Cc1ccccc1N2C(=O)OC(C)(C)C)cc3

Formula : $C_{20}H_{23}NO_3$

Charge : 0

Multiplicity : 1

Dipole : 1.6900 Debye

Energy : -1056.78931762 a.u.

Gibbs Energy : - 1056.45126800 a.u.

Number of imaginary frequencies : 1

Cartesian Coordinates (XYZ format)

47

C	-1.75493500	2.41895000	0.45091700
C	-1.96228700	1.30556800	-0.37409400
C	-0.34433300	2.36433400	0.98379400
C	0.04555000	0.88313000	0.74976600
H	-0.25020900	2.63880900	2.03483200
H	0.31417400	3.01948300	0.40317900
N	-0.79583800	0.51617300	-0.42998800
H	-0.29586300	0.29705500	1.61261200
C	1.51112600	0.62147500	0.52670200
C	-2.75820700	3.35966700	0.61107200
C	-3.98066600	3.17579300	-0.04794300
C	-4.17550600	2.06215300	-0.86311400
C	-3.16711900	1.10969200	-1.03947900
H	-2.60282200	4.22466200	1.24645900
H	-4.77702600	3.89924300	0.07852500
H	-5.12498700	1.92481700	-1.36758900
H	-3.32462100	0.24791700	-1.67679600
C	-0.91202600	-0.88450800	-0.69170400

O	-1.63656100	-1.47533300	0.25766800
O	-0.39475700	-1.41963200	-1.63851900
C	-1.89443800	-2.94048600	0.25869100
C	-2.74070500	-3.12057200	1.51353900
H	-2.18628200	-2.80134200	2.39822700
H	-3.00900200	-4.17234700	1.63074400
H	-3.65701600	-2.53126300	1.44384200
C	-2.67945500	-3.31887000	-0.99412100
H	-3.58534200	-2.71306200	-1.06941400
H	-2.97468700	-4.36845900	-0.92797500
H	-2.08142800	-3.18041800	-1.89292600
C	-0.56754900	-3.68534600	0.37491000
H	-0.00912800	-3.32881200	1.24345500
H	0.04097200	-3.55169800	-0.51751800
H	-0.76550100	-4.75120400	0.50859500
C	2.12152100	0.89669900	-0.69561400
C	2.30152900	0.12346000	1.56722500
C	3.48630300	0.69246200	-0.88566400
C	4.26656600	0.20397500	0.16736100
C	3.66321300	-0.08044000	1.39754000
H	1.51645500	1.25651000	-1.51847700
H	3.92459500	0.90830000	-1.84989000
O	5.60564400	-0.03325100	0.09068800
H	1.84354700	-0.10971700	2.52265700
H	4.27837400	-0.46766700	2.20044100
C	6.26552000	0.22964200	-1.14556000
H	7.31049400	-0.02665500	-0.98444300
H	6.18793300	1.28608200	-1.42060000
H	5.85902300	-0.38837500	-1.95205300

Frequencies

Mode	IR frequency	IR intensity	Raman intensity
1	-34.51150000	1.52180000	0.00000000
2	21.90910000	0.52330000	0.00000000
3	23.84470000	1.83390000	0.00000000
4	42.85790000	0.20980000	0.00000000
5	47.14670000	1.18330000	0.00000000
6	60.73980000	1.34550000	0.00000000
7	91.83900000	3.21400000	0.00000000
8	100.15590000	1.32020000	0.00000000
9	137.43900000	3.22900000	0.00000000
10	143.18950000	1.53040000	0.00000000
11	166.27200000	1.81650000	0.00000000
12	174.54680000	1.17350000	0.00000000
13	206.15270000	0.08430000	0.00000000
14	212.49800000	4.54360000	0.00000000
15	242.53440000	4.50160000	0.00000000
16	249.70290000	2.65220000	0.00000000
17	252.80350000	1.01760000	0.00000000

18	260.81560000	3.58150000	0.00000000
19	270.64430000	0.14620000	0.00000000
20	283.95430000	2.51460000	0.00000000
21	320.71920000	9.20260000	0.00000000
22	342.46410000	1.17130000	0.00000000
23	356.11230000	1.65480000	0.00000000
24	376.94090000	3.76920000	0.00000000
25	409.57310000	2.47390000	0.00000000
26	426.48860000	0.84570000	0.00000000
27	429.07580000	7.27070000	0.00000000
28	457.82370000	2.77710000	0.00000000
29	468.13170000	3.46310000	0.00000000
30	473.88820000	3.80770000	0.00000000
31	487.65380000	4.68300000	0.00000000
32	516.87420000	10.76360000	0.00000000
33	540.67570000	8.22310000	0.00000000
34	555.08860000	21.15720000	0.00000000
35	561.54800000	34.76120000	0.00000000
36	613.54890000	5.27330000	0.00000000
37	622.46470000	15.12210000	0.00000000
38	648.34570000	1.96370000	0.00000000
39	687.94660000	3.22950000	0.00000000
40	733.56130000	8.98160000	0.00000000
41	741.46530000	3.99280000	0.00000000
42	751.81070000	63.53280000	0.00000000
43	763.40530000	22.53910000	0.00000000
44	779.68340000	18.40240000	0.00000000
45	802.71870000	14.51610000	0.00000000
46	826.92420000	2.17240000	0.00000000
47	835.05290000	23.18600000	0.00000000
48	842.81400000	14.31510000	0.00000000
49	844.24590000	72.95900000	0.00000000
50	854.57180000	58.80190000	0.00000000
51	864.34850000	20.37960000	0.00000000
52	888.54210000	5.53110000	0.00000000
53	930.90550000	0.02770000	0.00000000
54	933.14860000	0.57240000	0.00000000
55	934.36960000	6.14400000	0.00000000
56	955.95940000	1.43510000	0.00000000
57	965.56990000	34.02230000	0.00000000
58	973.51910000	0.22080000	0.00000000
59	974.02290000	1.03950000	0.00000000
60	978.85940000	0.24090000	0.00000000

61	1013.59090000	52.38870000	0.00000000
62	1028.46490000	0.73430000	0.00000000
63	1041.74760000	14.26530000	0.00000000
64	1048.24170000	32.69340000	0.00000000
65	1053.09720000	1.08840000	0.00000000
66	1054.03080000	44.96910000	0.00000000
67	1056.63960000	42.42820000	0.00000000
68	1076.93820000	49.79830000	0.00000000
69	1128.75770000	47.54240000	0.00000000
70	1136.82690000	32.01260000	0.00000000
71	1162.21690000	747.59040000	0.00000000
72	1170.78500000	1.25160000	0.00000000
73	1175.84720000	6.63610000	0.00000000
74	1183.33170000	34.84160000	0.00000000
75	1192.46410000	45.74670000	0.00000000
76	1202.20480000	4.91510000	0.00000000
77	1203.96400000	21.87150000	0.00000000
78	1237.92600000	12.78250000	0.00000000
79	1245.89340000	229.52940000	0.00000000
80	1260.39240000	62.39740000	0.00000000
81	1272.03420000	28.43350000	0.00000000
82	1275.06660000	338.81380000	0.00000000
83	1282.84000000	70.58300000	0.00000000
84	1294.44800000	252.56640000	0.00000000
85	1314.77000000	91.39030000	0.00000000
86	1332.38310000	6.51500000	0.00000000
87	1336.59670000	38.28430000	0.00000000
88	1347.34810000	17.57060000	0.00000000
89	1374.02020000	65.79630000	0.00000000
90	1398.21760000	26.85760000	0.00000000
91	1399.17830000	19.33380000	0.00000000
92	1401.22910000	28.61080000	0.00000000
93	1423.85650000	19.21980000	0.00000000
94	1457.24920000	10.24990000	0.00000000
95	1468.17600000	0.65790000	0.00000000
96	1477.36820000	12.91160000	0.00000000
97	1477.67660000	6.06370000	0.00000000
98	1484.73490000	1.23680000	0.00000000
99	1485.32500000	1.22420000	0.00000000
100	1486.04730000	11.34630000	0.00000000
101	1491.60750000	45.14270000	0.00000000
102	1493.00100000	20.97840000	0.00000000
103	1498.91680000	2.38700000	0.00000000

104	1500.79750000	53.19340000	0.00000000
105	1512.15850000	166.73860000	0.00000000
106	1518.91890000	22.38780000	0.00000000
107	1546.60280000	167.38420000	0.00000000
108	1620.66190000	19.50210000	0.00000000
109	1633.19500000	9.23720000	0.00000000
110	1647.40030000	77.38600000	0.00000000
111	1655.39070000	90.68920000	0.00000000
112	1784.89090000	523.88070000	0.00000000
113	2997.40160000	40.44550000	0.00000000
114	3012.27740000	68.37780000	0.00000000
115	3028.24410000	42.37050000	0.00000000
116	3038.97550000	13.72490000	0.00000000
117	3041.29940000	33.26440000	0.00000000
118	3047.45410000	15.32270000	0.00000000
119	3073.51990000	49.43250000	0.00000000
120	3100.79490000	22.91650000	0.00000000
121	3104.32800000	6.32480000	0.00000000
122	3105.83320000	23.35850000	0.00000000
123	3112.34120000	45.31080000	0.00000000
124	3116.95330000	60.21050000	0.00000000
125	3140.13530000	0.91560000	0.00000000
126	3141.22090000	31.87680000	0.00000000
127	3143.02870000	26.10500000	0.00000000
128	3162.87700000	17.18220000	0.00000000
129	3164.49140000	4.48600000	0.00000000
130	3171.15460000	14.24280000	0.00000000
131	3182.76600000	37.74250000	0.00000000
132	3187.28130000	11.58570000	0.00000000
133	3191.26070000	12.20210000	0.00000000
134	3192.19970000	26.53340000	0.00000000
135	3211.46070000	9.12730000	0.00000000

Thermochemistry Data for *N*-Boc-2-(*p*-methoxyphenyl)indoline Calculations

N-Boc-2-(*p*-methoxyphenyl)indoline Rotamer **2d-1**:

	Temperature		
	298 K	223 K	195 K
ε_0	–1056.81277556 a.u.	–1056.81277556 a.u.	–1056.81277556 a.u.
ε_{ZPE}	0.391552 a.u.	0.391552 a.u.	0.391552 a.u.
E_{tot}	0.414003 a.u.	0.404781 a.u.	0.401968 a.u.
H_{corr}	0.414947 a.u.	0.405488 a.u.	0.402585 a.u.
G_{corr}	0.338469 a.u.	0.356388 a.u.	0.362359 a.u.
S_{tot}	160.961 calmol^{-1}K^{-1}	138.163 calmol^{-1}K^{-1}	129.450 calmol^{-1}K^{-1}
$\varepsilon_0 + \varepsilon_{ZPE}$	–1056.421224 a.u.	–1056.421224 a.u.	–1056.421224 a.u.
$\varepsilon_0 + E_{tot}$	–1056.398773 a.u.	–1056.407995 a.u.	–1056.410808 a.u.
$\varepsilon_0 + H_{corr}$	–1056.397828 a.u.	–1056.407288 a.u.	–1056.410191 a.u.
$\varepsilon_0 + G_{corr}$	–1056.474306 a.u.	–1056.456388 a.u.	–1056.450417 a.u.

N-Boc-2-(*p*-methoxyphenyl)indoline Rotamer **2d-2**:

	Temperature		
	298 K	223 K	195 K
ε_0	–1056.80977169 a.u.	–1056.80977169 a.u.	–1056.80977169 a.u.
ε_{ZPE}	0.391414 a.u.	0.391414 a.u.	0.391414 a.u.
E_{tot}	0.413045 a.u.	0.404056 a.u.	0.401330 a.u.
H_{corr}	0.413989 a.u.	0.404762 a.u.	0.401948 a.u.
G_{corr}	0.339480 a.u.	0.356935 a.u.	0.362751 a.u.
S_{tot}	156.818 calmol^{-1}K^{-1}	134.583 calmol^{-1}K^{-1}	126.135 calmol^{-1}K^{-1}
$\varepsilon_0 + \varepsilon_{ZPE}$	–1056.418358 a.u.	–1056.418358 a.u.	–1056.418358 a.u.
$\varepsilon_0 + E_{tot}$	–1056.396727 a.u.	–1056.405716 a.u.	–1056.408442 a.u.
$\varepsilon_0 + H_{corr}$	–1056.395783 a.u.	–1056.405010 a.u.	–1056.407824 a.u.
$\varepsilon_0 + G_{corr}$	–1056.470292 a.u.	–1056.452837 a.u.	–1056.447021 a.u.

TS for *N*-Boc-2-(*p*-methoxyphenyl)indoline Clockwise Rotation of Boc Group from Rotamer **2d-1** to Rotamer **2d-2**:

	Temperature	
	298 K	195 K
ε_0	–1056.78980787 a.u.	–1056.78980787 a.u.
ε_{ZPE}	0.390628 a.u.	0.390628 a.u.
E_{tot}	0.412419 a.u.	0.400670 a.u.
H_{corr}	0.413363 a.u.	0.401288 a.u.
G_{corr}	0.337773 a.u.	0.361412 a.u.
S_{tot}	159.092 calmol^{-1}K^{-1}	128.320 calmol^{-1}K^{-1}
$\varepsilon_0 + \varepsilon_{ZPE}$	–1056.399180 a.u.	–1056.399180 a.u.
$\varepsilon_0 + E_{tot}$	–1056.377389 a.u.	–1056.389138 a.u.
$\varepsilon_0 + H_{corr}$	–1056.376445 a.u.	–1056.388520 a.u.
$\varepsilon_0 + G_{corr}$	–1056.452035 a.u.	–1056.428396 a.u.

TS for *N*-Boc-2-(*p*-methoxyphenyl)indoline Anticlockwise Rotation of Boc Group from Rotamer **2d-1** to Rotamer **2d-2**:

	Temperature	
	298 K	195 K
ε_0	–1056.78931762 a.u.	–1056.78931762 a.u.
ε_{ZPE}	0.390553 a.u.	0.390553 a.u.
E_{tot}	0.412346 a.u.	0.400595 a.u.
H_{corr}	0.413290 a.u.	0.401213 a.u.
G_{corr}	0.338049 a.u.	0.361566 a.u.
S_{tot}	158.358 calmol^{-1}K^{-1}	127.581 calmol^{-1}K^{-1}
$\varepsilon_0 + \varepsilon_{ZPE}$	–1056.398764 a.u.	–1056.398765 a.u.
$\varepsilon_0 + E_{tot}$	–1056.376972 a.u.	–1056.388723 a.u.
$\varepsilon_0 + H_{corr}$	–1056.376028 a.u.	–1056.388105 a.u.
$\varepsilon_0 + G_{corr}$	–1056.451268 a.u.	–1056.427752 a.u.

10.3 Calculations Performed Using the def2tzvp Basis Set

N-Boc-2-phenylindoline 2a-1

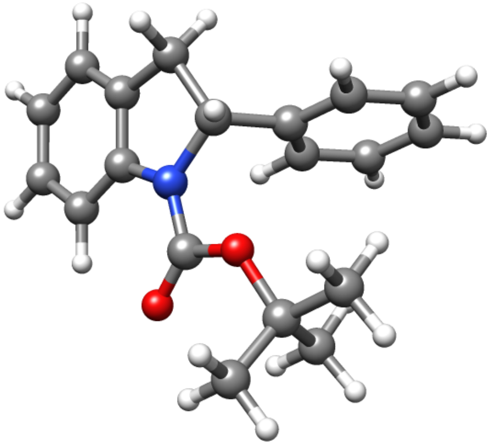

```
Route : # opt freq b3lyp scrf=(solvent=thf) geom=connectivity
          def2tzvp empiricaldispersion=gd3bj int=ultrafine
          pop=(regular,mk)
```

SMILES : CC(C)(C)OC(=O)N2c1ccccc1CC2c3ccccc3

Formula : $C_{19}H_{21}NO_2$

Charge : 0

Multiplicity : 1

Dipole : 3.9585 Debye

Energy : -942.36377881 a.u.

Gibbs Energy : -942.05358500 a.u.

Number of imaginary frequencies : 0

Cartesian Coordinates (XYZ format)

43

C	-4.80291400	-0.20980000	-0.36091600
C	-4.13933600	-1.29127600	-0.92956300
C	-4.10666800	0.68538600	0.45271700
C	-2.75899400	0.48270100	0.68212800
C	-2.10007300	-0.60262900	0.09933500
C	-2.77848500	-1.50564500	-0.71130100
C	-1.80316300	1.26848800	1.53641700
C	-0.41148900	0.67402900	1.18687800
H	-2.02383400	1.12765800	2.59662900
H	-1.83551500	2.33819200	1.33432400
N	-0.74147900	-0.57754100	0.46158800
H	-5.85804800	-0.06147000	-0.54878000
H	-4.61593800	1.52905400	0.90181100
H	-4.68318400	-1.98270100	-1.56039800

H	-2.26206100	-2.33787300	-1.15922400
C	0.20969200	-1.47647400	0.06460400
O	-0.00289000	-2.42765700	-0.66393000
O	1.39152800	-1.15295200	0.61210300
C	2.66047400	-1.69786800	0.10153500
C	2.75072300	-3.18985000	0.39667900
H	2.58712200	-3.37472600	1.45938700
H	3.74866700	-3.54745200	0.13812900
H	2.01652600	-3.74949100	-0.17657600
C	2.78662300	-1.38441900	-1.38484000
H	3.78310100	-1.66588700	-1.72752500
H	2.65242800	-0.31550900	-1.55607400
H	2.05165600	-1.93262000	-1.96956700
C	3.69614400	-0.92011500	0.90304100
H	3.57036500	-1.10387400	1.97064400
H	3.59405300	0.14929900	0.71846500
H	4.69937300	-1.23311400	0.61311300
H	0.13731400	0.42462600	2.09093700
C	0.43530000	1.60398100	0.34737400
C	0.22299000	1.73681200	-1.02346100
C	1.43466700	2.36090100	0.95201500
C	0.99938200	2.61036300	-1.77507400
C	2.21153700	3.23758400	0.20351800
C	1.99673300	3.36370300	-1.16438800
H	1.61730600	2.25094100	2.01426200
H	2.99114500	3.81278700	0.68637000
H	2.60500100	4.03951500	-1.75136000
H	-0.54168400	1.14622300	-1.51093700
H	0.82859700	2.69885500	-2.84032100

Frequencies

Mode	IR frequency	IR intensity	Raman intensity
1	27.84060000	0.23310000	0.00000000
2	32.67300000	0.00230000	0.00000000
3	36.26860000	0.87090000	0.00000000
4	42.66240000	0.57770000	0.00000000
5	69.15230000	3.15020000	0.00000000
6	87.44720000	1.59090000	0.00000000
7	101.57290000	0.89830000	0.00000000
8	131.52780000	0.33400000	0.00000000
9	180.91450000	0.93710000	0.00000000
10	212.17810000	0.36100000	0.00000000
11	212.93440000	1.46430000	0.00000000
12	220.08470000	6.80900000	0.00000000
13	228.71150000	5.56440000	0.00000000
14	254.98570000	0.42880000	0.00000000
15	275.42560000	3.08980000	0.00000000
16	279.50850000	0.50330000	0.00000000
17	294.85300000	1.08050000	0.00000000

18	331.91620000	14.52490000	0.00000000
19	344.77570000	1.66710000	0.00000000
20	359.19090000	1.17700000	0.00000000
21	407.00600000	4.39690000	0.00000000
22	414.42270000	0.17200000	0.00000000
23	430.61080000	0.82760000	0.00000000
24	435.03020000	6.41060000	0.00000000
25	463.56520000	6.23900000	0.00000000
26	474.61110000	3.98860000	0.00000000
27	492.80540000	1.01020000	0.00000000
28	546.55430000	3.41560000	0.00000000
29	564.06030000	7.16690000	0.00000000
30	598.33210000	11.07510000	0.00000000
31	628.97390000	22.30440000	0.00000000
32	637.02700000	8.50490000	0.00000000
33	638.59950000	0.48450000	0.00000000
34	715.34530000	68.59780000	0.00000000
35	729.43630000	12.97340000	0.00000000
36	749.71020000	28.58320000	0.00000000
37	765.02550000	66.30130000	0.00000000
38	773.49780000	12.28460000	0.00000000
39	783.44960000	40.46020000	0.00000000
40	786.32410000	21.96290000	0.00000000
41	803.45110000	6.90330000	0.00000000
42	821.61530000	5.28570000	0.00000000
43	857.36370000	60.90520000	0.00000000
44	859.70490000	4.12660000	0.00000000
45	870.29350000	5.09850000	0.00000000
46	880.70550000	3.76830000	0.00000000
47	930.23770000	3.23830000	0.00000000
48	931.99870000	0.15010000	0.00000000
49	932.99670000	0.33780000	0.00000000
50	940.29560000	1.52470000	0.00000000
51	975.63970000	0.08070000	0.00000000
52	978.76380000	0.07810000	0.00000000
53	980.42320000	0.24450000	0.00000000
54	985.66960000	8.39930000	0.00000000
55	996.63100000	0.27360000	0.00000000
56	1025.30130000	0.33090000	0.00000000
57	1027.67850000	3.08630000	0.00000000
58	1039.85860000	140.61040000	0.00000000
59	1051.76350000	12.43720000	0.00000000
60	1055.43750000	12.40300000	0.00000000

61	1055.96380000	16.09570000	0.00000000
62	1065.11470000	30.29850000	0.00000000
63	1105.07280000	51.09760000	0.00000000
64	1113.69370000	2.37570000	0.00000000
65	1158.44760000	327.39840000	0.00000000
66	1177.42730000	15.28540000	0.00000000
67	1179.24720000	25.24850000	0.00000000
68	1181.07250000	6.20050000	0.00000000
69	1189.92030000	336.50980000	0.00000000
70	1202.71520000	20.66970000	0.00000000
71	1204.83150000	12.54070000	0.00000000
72	1234.82180000	5.35490000	0.00000000
73	1248.68350000	11.80120000	0.00000000
74	1271.24580000	42.91870000	0.00000000
75	1276.95690000	75.36920000	0.00000000
76	1288.23870000	28.43540000	0.00000000
77	1307.13170000	202.24450000	0.00000000
78	1317.24960000	35.31060000	0.00000000
79	1338.24930000	52.36740000	0.00000000
80	1354.44980000	95.77220000	0.00000000
81	1357.83180000	19.30920000	0.00000000
82	1392.08640000	119.30410000	0.00000000
83	1399.53200000	26.25030000	0.00000000
84	1402.25300000	27.26310000	0.00000000
85	1415.47310000	617.93150000	0.00000000
86	1426.07380000	32.13660000	0.00000000
87	1468.52610000	0.39110000	0.00000000
88	1481.84800000	14.99560000	0.00000000
89	1485.41520000	1.75210000	0.00000000
90	1486.28320000	1.58110000	0.00000000
91	1490.12670000	19.46130000	0.00000000
92	1493.45120000	14.42270000	0.00000000
93	1498.52110000	7.29740000	0.00000000
94	1498.68830000	33.46990000	0.00000000
95	1516.78730000	119.08960000	0.00000000
96	1519.34350000	138.85600000	0.00000000
97	1534.70530000	14.50900000	0.00000000
98	1629.76330000	0.36630000	0.00000000
99	1634.48460000	4.03760000	0.00000000
100	1643.01230000	50.35580000	0.00000000
101	1648.27150000	7.46670000	0.00000000
102	1718.28640000	600.13870000	0.00000000
103	3042.24140000	14.71800000	0.00000000

104	3044.10850000	35.29450000	0.00000000
105	3046.21080000	33.81660000	0.00000000
106	3050.85080000	23.52360000	0.00000000
107	3096.18090000	14.41080000	0.00000000
108	3102.80490000	14.92480000	0.00000000
109	3108.62630000	15.49510000	0.00000000
110	3111.02870000	23.57270000	0.00000000
111	3113.13580000	47.57560000	0.00000000
112	3119.96570000	50.59100000	0.00000000
113	3137.93010000	10.45020000	0.00000000
114	3146.44890000	17.87370000	0.00000000
115	3164.47240000	7.84690000	0.00000000
116	3171.12800000	2.00800000	0.00000000
117	3172.11160000	0.31230000	0.00000000
118	3179.18510000	23.29850000	0.00000000
119	3180.56800000	17.81790000	0.00000000
120	3188.39670000	34.03970000	0.00000000
121	3195.00030000	32.36640000	0.00000000
122	3197.00550000	17.26020000	0.00000000
123	3248.28590000	1.86900000	0.00000000

N-Boc-2-phenylindoline 2a-2

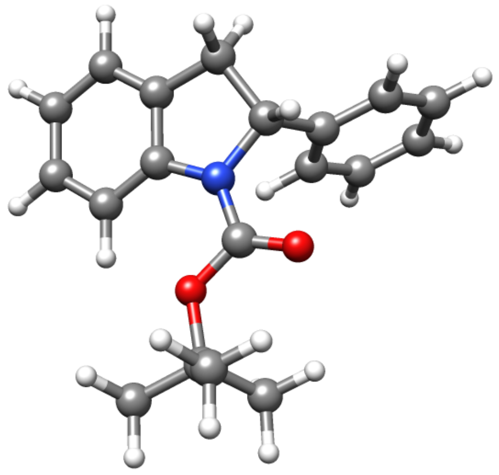

Route : # opt freq b3lyp scrf=(solvent=thf) geom=connectivity
def2tzvp empiricaldispersion=gd3bj int=ultrafine
pop=(regular,mk)

SMILES : CC(C)(C)OC(=O)N2c1ccccc1CC2c3ccccc3

Formula : $C_{19}H_{21}NO_2$

Charge : 0

Multiplicity : 1

Dipole : 1.6882 Debye

Energy : -942.36163469 a.u.

Gibbs Energy : -942.05288700 a.u.

Number of imaginary frequencies : 0

Cartesian Coordinates (XYZ format)

43

C	-0.16173900	4.27257200	0.58928600
C	0.94894200	3.55092100	1.01015800
C	-1.12208200	3.66005700	-0.21668600
C	-0.95335900	2.33932600	-0.58593400
C	0.16498500	1.61803600	-0.15455000
C	1.12980500	2.21594800	0.64869400
C	-1.81442200	1.47591600	-1.46522300
C	-1.25574700	0.04886200	-1.22988400
H	-1.70426200	1.76308700	-2.51368600
H	-2.87272600	1.53269900	-1.21647900
N	0.07644800	0.29649800	-0.63967600
H	-0.28272700	5.30568100	0.88678700
H	-1.99123700	4.21234300	-0.55189000
H	1.69205000	4.02657500	1.63731700
H	1.99068700	1.66699600	0.98827400

C	0.98323800	-0.72830100	-0.61063400
O	0.73962600	-1.82332900	-1.08392100
O	2.12635800	-0.36392400	-0.01881300
C	3.25504100	-1.30215100	0.13300400
C	2.83514800	-2.48357100	0.99896000
H	2.42001500	-2.13004000	1.94388600
H	3.71174400	-3.09450000	1.21911700
H	2.09568100	-3.10051900	0.49573400
C	3.76412400	-1.73089800	-1.23769500
H	4.68203800	-2.30715800	-1.11358700
H	3.99174200	-0.85484400	-1.84676100
H	3.03316200	-2.34463400	-1.75705400
C	4.29463000	-0.45104200	0.85084600
H	3.91523400	-0.11300500	1.81571500
H	4.55630500	0.42176200	0.25203200
H	5.19705600	-1.03826800	1.02107600
H	-1.12737200	-0.48343800	-2.16861300
C	-2.14208600	-0.77432400	-0.32046000
C	-2.03024900	-0.71045500	1.06651800
C	-3.13388700	-1.57634200	-0.88090100
C	-2.89821300	-1.43270700	1.87779600
C	-4.00336500	-2.29854300	-0.07265400
C	-3.88865900	-2.22757900	1.31170600
H	-3.22063300	-1.64189900	-1.95906700
H	-4.76425900	-2.92283900	-0.52336300
H	-4.56131500	-2.79267100	1.94379600
H	-1.25881800	-0.10121800	1.51807500
H	-2.79800300	-1.37591900	2.95421800

Frequencies

Mode	IR frequency	IR intensity	Raman intensity
1	23.63270000	0.56780000	0.00000000
2	27.03750000	0.21770000	0.00000000
3	36.08920000	0.01330000	0.00000000
4	37.88640000	0.62840000	0.00000000
5	74.10650000	2.21820000	0.00000000
6	91.70550000	0.63430000	0.00000000
7	96.03310000	0.70200000	0.00000000
8	122.21130000	1.28530000	0.00000000
9	173.79740000	1.31760000	0.00000000
10	207.59450000	0.04890000	0.00000000
11	213.24520000	5.16360000	0.00000000
12	221.54990000	4.24400000	0.00000000
13	237.47340000	1.51360000	0.00000000
14	248.57490000	1.43970000	0.00000000
15	274.93460000	0.22910000	0.00000000
16	277.06810000	0.06270000	0.00000000
17	303.57340000	4.60240000	0.00000000
18	329.23450000	11.55250000	0.00000000

19	348.02220000	3.24290000	0.00000000
20	358.22050000	5.43030000	0.00000000
21	405.82900000	7.80730000	0.00000000
22	414.41460000	0.32170000	0.00000000
23	425.54770000	2.44310000	0.00000000
24	433.95990000	6.34110000	0.00000000
25	461.13150000	3.77520000	0.00000000
26	472.33870000	6.89870000	0.00000000
27	489.28540000	10.10290000	0.00000000
28	544.96160000	3.17620000	0.00000000
29	562.90470000	7.63580000	0.00000000
30	596.29540000	9.74490000	0.00000000
31	622.82330000	10.23610000	0.00000000
32	636.98680000	0.86770000	0.00000000
33	666.06920000	23.31540000	0.00000000
34	715.04430000	64.76380000	0.00000000
35	728.74460000	10.58290000	0.00000000
36	736.21730000	11.06130000	0.00000000
37	763.75210000	100.96520000	0.00000000
38	770.55860000	4.19860000	0.00000000
39	780.44860000	29.39920000	0.00000000
40	789.10790000	13.29400000	0.00000000
41	800.00250000	0.86150000	0.00000000
42	827.50810000	3.71630000	0.00000000
43	850.25750000	9.30210000	0.00000000
44	858.21070000	0.67140000	0.00000000
45	874.03490000	34.80150000	0.00000000
46	878.94770000	42.59670000	0.00000000
47	928.18780000	4.57450000	0.00000000
48	931.62330000	0.07110000	0.00000000
49	931.80340000	1.03030000	0.00000000
50	940.25390000	0.96070000	0.00000000
51	972.17740000	0.01900000	0.00000000
52	973.72330000	0.08760000	0.00000000
53	975.82240000	0.06740000	0.00000000
54	983.15180000	11.79180000	0.00000000
55	994.00020000	0.56560000	0.00000000
56	1024.32980000	0.36700000	0.00000000
57	1027.16310000	24.25180000	0.00000000
58	1043.51090000	64.36570000	0.00000000
59	1053.62670000	1.24500000	0.00000000
60	1054.41770000	14.92990000	0.00000000
61	1056.49850000	5.15120000	0.00000000

62	1068.22130000	41.02830000	0.00000000
63	1104.25790000	37.53430000	0.00000000
64	1115.13610000	4.37010000	0.00000000
65	1150.94430000	211.70100000	0.00000000
66	1177.62820000	44.67920000	0.00000000
67	1180.88570000	3.17390000	0.00000000
68	1181.04660000	58.51720000	0.00000000
69	1189.63040000	249.55340000	0.00000000
70	1196.38370000	52.37360000	0.00000000
71	1204.96430000	3.15860000	0.00000000
72	1233.43000000	5.01400000	0.00000000
73	1243.75220000	36.09970000	0.00000000
74	1270.76390000	22.88230000	0.00000000
75	1278.78470000	78.27650000	0.00000000
76	1293.78810000	87.46400000	0.00000000
77	1302.55640000	172.80960000	0.00000000
78	1318.40290000	11.15130000	0.00000000
79	1344.76210000	34.83800000	0.00000000
80	1357.59860000	18.16740000	0.00000000
81	1364.49350000	181.32470000	0.00000000
82	1389.28240000	291.07790000	0.00000000
83	1398.42080000	26.74860000	0.00000000
84	1401.12350000	51.61320000	0.00000000
85	1408.62780000	333.92970000	0.00000000
86	1423.48600000	22.02850000	0.00000000
87	1467.54870000	0.16340000	0.00000000
88	1480.80150000	20.85550000	0.00000000
89	1484.88870000	2.27300000	0.00000000
90	1485.57320000	1.72400000	0.00000000
91	1487.57590000	30.29540000	0.00000000
92	1491.71160000	13.38100000	0.00000000
93	1497.60670000	3.16280000	0.00000000
94	1499.85700000	40.64390000	0.00000000
95	1515.30130000	55.93300000	0.00000000
96	1518.14040000	212.52370000	0.00000000
97	1534.30800000	14.76190000	0.00000000
98	1628.77810000	2.82830000	0.00000000
99	1633.24230000	15.23790000	0.00000000
100	1645.71250000	85.96260000	0.00000000
101	1647.79940000	4.73510000	0.00000000
102	1708.28150000	786.76560000	0.00000000
103	3041.07560000	35.04310000	0.00000000
104	3041.99000000	15.57450000	0.00000000

105	3044.49040000	36.53690000	0.00000000
106	3051.12500000	18.73320000	0.00000000
107	3102.53050000	15.90500000	0.00000000
108	3103.94700000	5.91810000	0.00000000
109	3105.55980000	26.06660000	0.00000000
110	3111.84260000	42.06050000	0.00000000
111	3112.89940000	21.92330000	0.00000000
112	3118.72720000	62.80900000	0.00000000
113	3144.88170000	0.40790000	0.00000000
114	3147.91520000	23.99510000	0.00000000
115	3163.07130000	9.02650000	0.00000000
116	3171.64380000	1.55580000	0.00000000
117	3171.78870000	0.41690000	0.00000000
118	3179.30180000	22.35120000	0.00000000
119	3180.96240000	23.42520000	0.00000000
120	3190.47900000	35.57840000	0.00000000
121	3195.61930000	30.30750000	0.00000000
122	3197.51020000	14.03130000	0.00000000
123	3258.48110000	2.29970000	0.00000000

N-Boc-2-phenylindoline Clockwise TS from 2a-1 to 2a-2

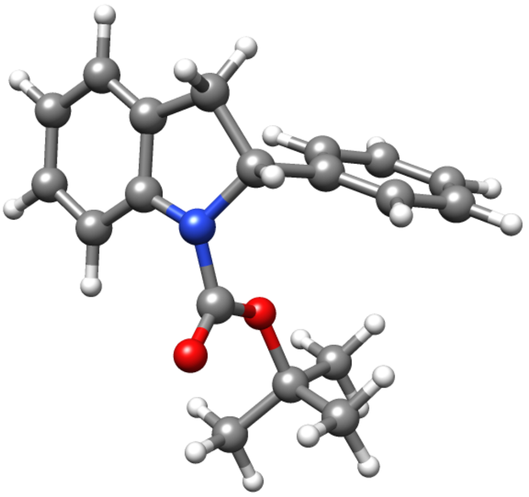

Route : # opt=qst3 freq b3lyp scrf=(solvent=thf)geom=connectivity
def2tzvp empiricaldispersion=gd3bj int=ultrafine
pop=(regular,mk)

SMILES : CC(C)(C)OC(=O)N2c1ccccc1CC2c3ccccc3

Formula : $C_{19}H_{21}NO_2$

Charge : 0

Multiplicity : 1

Dipole : 3.8957 Debye

Energy : -942.33946482 a.u.

Gibbs Energy : -942.02806900 a.u.

Number of imaginary frequencies : 1

Cartesian Coordinates (XYZ format)

43

C	-0.16173900	4.27257200	0.58928600
C	0.94894200	3.55092100	1.01015800
C	-1.12208200	3.66005700	-0.21668600
C	-0.95335900	2.33932600	-0.58593400
C	0.16498500	1.61803600	-0.15455000
C	1.12980500	2.21594800	0.64869400
C	-1.81442200	1.47591600	-1.46522300
C	-1.25574700	0.04886200	-1.22988400
H	-1.70426200	1.76308700	-2.51368600
H	-2.87272600	1.53269900	-1.21647900
N	0.07644800	0.29649800	-0.63967600
H	-0.28272700	5.30568100	0.88678700
H	-1.99123700	4.21234300	-0.55189000
H	1.69205000	4.02657500	1.63731700
H	1.99068700	1.66699600	0.98827400
C	0.98323800	-0.72830100	-0.61063400
O	0.73962600	-1.82332900	-1.08392100

O	2.12635800	-0.36392400	-0.01881300
C	3.25504100	-1.30215100	0.13300400
C	2.83514800	-2.48357100	0.99896000
H	2.42001500	-2.13004000	1.94388600
H	3.71174400	-3.09450000	1.21911700
H	2.09568100	-3.10051900	0.49573400
C	3.76412400	-1.73089800	-1.23769500
H	4.68203800	-2.30715800	-1.11358700
H	3.99174200	-0.85484400	-1.84676100
H	3.03316200	-2.34463400	-1.75705400
C	4.29463000	-0.45104200	0.85084600
H	3.91523400	-0.11300500	1.81571500
H	4.55630500	0.42176200	0.25203200
H	5.19705600	-1.03826800	1.02107600
H	-1.12737200	-0.48343800	-2.16861300
C	-2.14208600	-0.77432400	-0.32046000
C	-2.03024900	-0.71045500	1.06651800
C	-3.13388700	-1.57634200	-0.88090100
C	-2.89821300	-1.43270700	1.87779600
C	-4.00336500	-2.29854300	-0.07265400
C	-3.88865900	-2.22757900	1.31170600
H	-3.22063300	-1.64189900	-1.95906700
H	-4.76425900	-2.92283900	-0.52336300
H	-4.56131500	-2.79267100	1.94379600
H	-1.25881800	-0.10121800	1.51807500
H	-2.79800300	-1.37591900	2.95421800

Frequencies

Mode	IR frequency	IR intensity	Raman intensity
1	-51.33260000	2.34450000	0.00000000
2	36.93920000	0.03150000	0.00000000
3	43.21810000	0.80840000	0.00000000
4	48.22220000	0.22530000	0.00000000
5	63.63360000	1.26160000	0.00000000
6	77.58320000	0.92450000	0.00000000
7	92.04360000	0.68950000	0.00000000
8	145.05560000	2.18520000	0.00000000
9	151.36880000	1.45290000	0.00000000
10	196.99910000	4.48590000	0.00000000
11	200.79600000	0.86450000	0.00000000
12	205.35880000	0.32690000	0.00000000
13	242.11400000	4.08540000	0.00000000
14	247.42160000	1.16730000	0.00000000
15	269.47970000	0.35070000	0.00000000
16	272.34120000	0.35610000	0.00000000
17	293.15480000	6.72960000	0.00000000
18	320.99740000	8.31720000	0.00000000
19	352.96800000	5.43520000	0.00000000

20	356.83290000	1.81260000	0.00000000
21	407.51100000	2.19640000	0.00000000
22	414.13710000	0.08860000	0.00000000
23	426.58340000	5.35030000	0.00000000
24	432.54800000	2.15520000	0.00000000
25	466.63270000	3.66750000	0.00000000
26	475.32210000	7.12760000	0.00000000
27	518.13110000	4.75800000	0.00000000
28	543.81370000	2.89590000	0.00000000
29	556.36500000	15.98910000	0.00000000
30	567.06230000	1.92310000	0.00000000
31	616.81370000	24.36800000	0.00000000
32	635.15590000	4.18640000	0.00000000
33	641.59530000	13.80850000	0.00000000
34	715.79740000	72.66640000	0.00000000
35	725.31600000	14.18300000	0.00000000
36	742.98130000	12.75120000	0.00000000
37	754.83460000	81.94140000	0.00000000
38	763.39470000	11.37730000	0.00000000
39	772.17160000	19.91660000	0.00000000
40	794.17080000	22.72650000	0.00000000
41	825.84900000	13.45650000	0.00000000
42	830.95450000	8.69470000	0.00000000
43	848.40830000	70.59380000	0.00000000
44	857.37940000	12.12340000	0.00000000
45	861.91650000	0.58970000	0.00000000
46	868.73830000	2.38920000	0.00000000
47	926.72310000	2.88090000	0.00000000
48	933.76470000	0.07360000	0.00000000
49	934.99450000	0.09510000	0.00000000
50	936.79480000	3.64040000	0.00000000
51	959.36910000	0.11250000	0.00000000
52	976.12820000	0.18120000	0.00000000
53	978.23470000	17.57370000	0.00000000
54	983.47260000	1.19070000	0.00000000
55	1003.36580000	0.23640000	0.00000000
56	1013.79210000	0.71070000	0.00000000
57	1025.11450000	11.97310000	0.00000000
58	1027.19470000	13.48740000	0.00000000
59	1048.59170000	22.67170000	0.00000000
60	1054.87260000	1.06280000	0.00000000
61	1056.22960000	11.23130000	0.00000000
62	1061.90440000	13.77090000	0.00000000

63	1087.47210000	62.38910000	0.00000000
64	1110.28620000	6.33370000	0.00000000
65	1138.85310000	68.81770000	0.00000000
66	1172.96080000	509.37370000	0.00000000
67	1174.83720000	33.12990000	0.00000000
68	1179.42640000	50.13180000	0.00000000
69	1181.91700000	2.26880000	0.00000000
70	1192.03670000	7.57420000	0.00000000
71	1207.04150000	1.81220000	0.00000000
72	1233.29000000	18.71880000	0.00000000
73	1250.14080000	41.54310000	0.00000000
74	1266.77520000	238.50550000	0.00000000
75	1274.44850000	31.05600000	0.00000000
76	1284.88070000	42.97430000	0.00000000
77	1291.20530000	245.81610000	0.00000000
78	1316.78030000	23.31740000	0.00000000
79	1328.73460000	43.85240000	0.00000000
80	1353.63600000	33.12100000	0.00000000
81	1357.97600000	10.70290000	0.00000000
82	1387.89460000	192.84180000	0.00000000
83	1398.21900000	14.63020000	0.00000000
84	1400.26990000	24.95220000	0.00000000
85	1403.67800000	24.74000000	0.00000000
86	1427.04380000	17.31650000	0.00000000
87	1468.40330000	0.32210000	0.00000000
88	1479.22750000	12.76730000	0.00000000
89	1484.90100000	2.16300000	0.00000000
90	1485.56370000	1.70100000	0.00000000
91	1490.99160000	22.59480000	0.00000000
92	1494.76400000	55.65890000	0.00000000
93	1495.83940000	1.23350000	0.00000000
94	1497.65790000	3.61560000	0.00000000
95	1516.91960000	17.03500000	0.00000000
96	1517.85540000	191.60830000	0.00000000
97	1534.17040000	14.44540000	0.00000000
98	1628.91350000	0.87610000	0.00000000
99	1632.64890000	2.50760000	0.00000000
100	1643.91490000	112.33440000	0.00000000
101	1647.68920000	3.39510000	0.00000000
102	1753.34560000	632.43310000	0.00000000
103	3040.07470000	31.63970000	0.00000000
104	3044.75040000	10.81770000	0.00000000
105	3046.91810000	31.83960000	0.00000000

106	3053.28240000	21.04930000	0.00000000
107	3079.27460000	50.49750000	0.00000000
108	3103.56350000	17.20520000	0.00000000
109	3106.91280000	11.25180000	0.00000000
110	3110.54510000	12.50290000	0.00000000
111	3116.47750000	54.89170000	0.00000000
112	3124.89190000	26.12100000	0.00000000
113	3140.32530000	0.56980000	0.00000000
114	3143.05220000	25.12210000	0.00000000
115	3162.55890000	8.77140000	0.00000000
116	3168.48960000	6.72570000	0.00000000
117	3171.28430000	0.24800000	0.00000000
118	3174.74170000	13.82630000	0.00000000
119	3180.24140000	19.45980000	0.00000000
120	3187.51760000	27.57340000	0.00000000
121	3188.80410000	32.79600000	0.00000000
122	3196.65270000	19.73520000	0.00000000
123	3197.00170000	18.82560000	0.00000000

N-Boc-2-phenylindoline Anticlockwise TS from 2a-1 to 2a-2

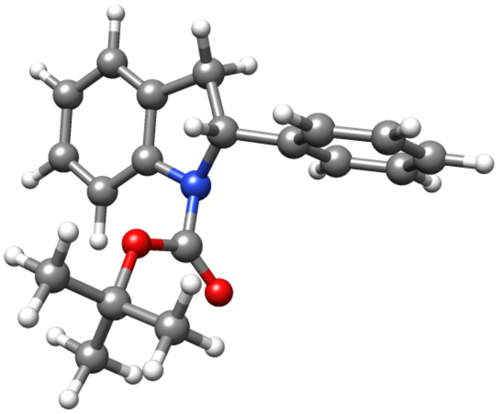

Route : # opt=qst3 freq b3lyp scrf=(solvent=thf)geom=connectivity
def2tzvp empiricaldispersion=gd3bj int=ultrafine
pop=(regular,mk)

SMILES : CC(C)(C)OC(=O)N2c1ccccc1CC2c3ccccc3

Formula : $C_{19}H_{21}NO_2$

Charge : 0

Multiplicity : 1

Dipole : 3.4772 Debye

Energy : -942.34119272 a.u.

Gibbs Energy : -942.03124700 a.u.

Number of imaginary frequencies : 1

Cartesian Coordinates (XYZ format)

43

C	-3.46394900	3.06310300	0.17496700
C	-3.68663400	1.94515100	-0.62223400
C	-2.20250500	3.28565700	0.73492400
C	-1.18765500	2.37942700	0.49465500
C	-1.42371700	1.26149400	-0.31044100
C	-2.66749900	1.02746800	-0.87862900
C	0.25819400	2.37251500	0.91482800
C	0.68530900	0.91312900	0.63291100
H	0.42231800	2.63870100	1.95777300
H	0.84264400	3.06099900	0.29793300
N	-0.24430900	0.51126400	-0.46200800
H	-4.26927300	3.76017400	0.36455700
H	-2.02600200	4.15491700	1.35652900
H	-4.66680500	1.77774400	-1.04999900
H	-2.84824500	0.16030900	-1.50014800
C	-0.33524500	-0.88919100	-0.72062900
O	0.11050500	-1.39882200	-1.71585100
O	-0.94677000	-1.51076800	0.28174400
C	-1.16126800	-2.97957600	0.29707400
C	-2.05096200	-3.37637500	-0.87301000

H	-2.97488700	-2.79672100	-0.86039100
H	-2.31029900	-4.43160700	-0.77940800
H	-1.54860400	-3.22619300	-1.82513900
C	0.18646100	-3.68795400	0.28165600
H	0.02789200	-4.75655000	0.43174900
H	0.81743600	-3.31971700	1.09168900
H	0.70233900	-3.54265500	-0.66389500
C	-1.87637500	-3.19223500	1.62297800
H	-2.81336200	-2.63544200	1.64673300
H	-1.25190700	-2.86148000	2.45329400
H	-2.09757900	-4.25137600	1.75540100
H	0.45815000	0.31004200	1.51905800
C	2.13310900	0.72191100	0.27329400
C	2.63496800	1.16133900	-0.95182900
C	2.99983300	0.12122400	1.18201800
C	3.98036100	1.00870700	-1.25608000
C	4.35039000	-0.02858300	0.88226400
C	4.84351400	0.41551200	-0.33811300
H	2.61424800	-0.23386500	2.13031300
H	5.01264300	-0.49770000	1.59847300
H	5.89216100	0.29531700	-0.57755100
H	1.96189500	1.60804400	-1.67157600
H	4.35754100	1.34860100	-2.21210500

Frequencies

Mode	IR frequency	IR intensity	Raman intensity
1	-32.35990000	1.75210000	0.00000000
2	21.83840000	0.12260000	0.00000000
3	31.93760000	0.30950000	0.00000000
4	48.28880000	0.47580000	0.00000000
5	57.81380000	1.49080000	0.00000000
6	76.56770000	0.92430000	0.00000000
7	99.00930000	1.44780000	0.00000000
8	148.89750000	2.60740000	0.00000000
9	158.77580000	2.40030000	0.00000000
10	191.73410000	0.50110000	0.00000000
11	200.08640000	3.40070000	0.00000000
12	208.69560000	0.08820000	0.00000000
13	236.43080000	3.63920000	0.00000000
14	249.08540000	0.86300000	0.00000000
15	267.80170000	5.49540000	0.00000000
16	271.70680000	0.02490000	0.00000000
17	292.34150000	0.44780000	0.00000000
18	320.05660000	8.23850000	0.00000000
19	353.72100000	1.95450000	0.00000000
20	372.89190000	6.32630000	0.00000000
21	402.72370000	2.08100000	0.00000000

22	415.94140000	0.39400000	0.00000000
23	428.82720000	6.67990000	0.00000000
24	439.57390000	3.30940000	0.00000000
25	466.75590000	4.06600000	0.00000000
26	474.86440000	6.48470000	0.00000000
27	517.25120000	14.14650000	0.00000000
28	546.63530000	3.76710000	0.00000000
29	558.43390000	16.20990000	0.00000000
30	577.72450000	7.71430000	0.00000000
31	621.53400000	8.35940000	0.00000000
32	636.53690000	0.58300000	0.00000000
33	644.76190000	10.22400000	0.00000000
34	695.69920000	2.71970000	0.00000000
35	715.30010000	66.43800000	0.00000000
36	737.81030000	16.89880000	0.00000000
37	756.05930000	82.13200000	0.00000000
38	768.10540000	6.82490000	0.00000000
39	777.37610000	51.18020000	0.00000000
40	789.97400000	11.97710000	0.00000000
41	835.27660000	18.15920000	0.00000000
42	838.01800000	11.62450000	0.00000000
43	853.80430000	61.93980000	0.00000000
44	860.84740000	0.84450000	0.00000000
45	868.61060000	7.74720000	0.00000000
46	887.84020000	12.57010000	0.00000000
47	932.70180000	6.29540000	0.00000000
48	933.61110000	0.05350000	0.00000000
49	933.98770000	0.18500000	0.00000000
50	935.85530000	3.15010000	0.00000000
51	965.35250000	0.19500000	0.00000000
52	967.28610000	33.67000000	0.00000000
53	975.49090000	0.08150000	0.00000000
54	980.91060000	0.26660000	0.00000000
55	997.74090000	3.88570000	0.00000000
56	1015.54540000	40.23840000	0.00000000
57	1025.10550000	0.53770000	0.00000000
58	1042.39510000	8.84780000	0.00000000
59	1049.72460000	27.99710000	0.00000000
60	1054.24750000	0.56620000	0.00000000
61	1055.96220000	10.20990000	0.00000000
62	1058.21090000	17.57130000	0.00000000
63	1078.47410000	44.76870000	0.00000000
64	1105.12680000	10.07950000	0.00000000

65	1138.03370000	47.47020000	0.00000000
66	1164.49310000	693.67990000	0.00000000
67	1178.54010000	5.12490000	0.00000000
68	1180.47540000	2.07720000	0.00000000
69	1187.01070000	18.65830000	0.00000000
70	1200.29220000	1.81520000	0.00000000
71	1205.13020000	1.96450000	0.00000000
72	1237.89030000	14.45060000	0.00000000
73	1249.38030000	226.17510000	0.00000000
74	1265.22530000	31.81980000	0.00000000
75	1273.62790000	26.66720000	0.00000000
76	1283.05320000	72.27040000	0.00000000
77	1293.77120000	282.48140000	0.00000000
78	1313.26970000	37.30150000	0.00000000
79	1335.05450000	6.42100000	0.00000000
80	1348.17700000	7.94440000	0.00000000
81	1355.83700000	8.23560000	0.00000000
82	1376.40130000	63.10470000	0.00000000
83	1401.02330000	24.20480000	0.00000000
84	1403.08680000	27.46020000	0.00000000
85	1408.44260000	12.76290000	0.00000000
86	1425.97240000	17.30510000	0.00000000
87	1469.10790000	0.56090000	0.00000000
88	1478.94160000	5.55000000	0.00000000
89	1483.88510000	0.10200000	0.00000000
90	1484.97400000	2.30680000	0.00000000
91	1491.08560000	21.07270000	0.00000000
92	1493.24890000	25.75940000	0.00000000
93	1496.99790000	33.53760000	0.00000000
94	1497.55690000	2.78170000	0.00000000
95	1516.57330000	138.18920000	0.00000000
96	1516.70570000	42.30910000	0.00000000
97	1534.10950000	10.50110000	0.00000000
98	1629.44820000	0.52200000	0.00000000
99	1633.12110000	17.02260000	0.00000000
100	1646.42860000	83.16630000	0.00000000
101	1649.01820000	2.89470000	0.00000000
102	1768.10290000	546.82970000	0.00000000
103	3004.05740000	39.23200000	0.00000000
104	3034.14840000	36.18410000	0.00000000
105	3044.23060000	14.51780000	0.00000000
106	3047.12020000	33.46270000	0.00000000
107	3053.16750000	14.49820000	0.00000000

108	3104.37250000	19.65620000	0.00000000
109	3107.47140000	6.88180000	0.00000000
110	3109.39510000	19.98910000	0.00000000
111	3114.77300000	41.98280000	0.00000000
112	3120.17090000	54.54470000	0.00000000
113	3142.54410000	1.26960000	0.00000000
114	3145.56440000	23.63970000	0.00000000
115	3165.01030000	7.75200000	0.00000000
116	3169.72840000	5.17540000	0.00000000
117	3172.60160000	0.31350000	0.00000000
118	3176.12680000	11.09970000	0.00000000
119	3181.58950000	22.06270000	0.00000000
120	3187.76390000	30.54930000	0.00000000
121	3190.37310000	33.32490000	0.00000000
122	3197.21340000	30.05970000	0.00000000
123	3197.51950000	8.55030000	0.00000000

Thermochemistry Data for *N*-Boc-2-phenylindoline Calculations

N-Boc-2-phenylindoline Rotamer **2a-1**:

	Temperature		
	298 K	223 K	195 K
ε_0	–942.363778810 a.u.	–942.363778810 a.u.	–942.363778810 a.u.
ε_{ZPE}	0.359698 a.u.	0.359698 a.u.	0.359698 a.u.
E_{tot}	0.379500 a.u.	0.371318 a.u.	0.368847 a.u.
H_{corr}	0.380444 a.u.	0.372024 a.u.	0.369465 a.u.
G_{corr}	0.310194 a.u.	0.326690 a.u.	0.332211 a.u.
S_{tot}	147.853 calmol^{-1}K^{-1}	127.569 calmol^{-1}K^{-1}	119.883 calmol^{-1}K^{-1}
$\varepsilon_0 + \varepsilon_{ZPE}$	–942.004081 a.u.	–942.004081 a.u.	–942.004081 a.u.
$\varepsilon_0 + E_{tot}$	–941.984279 a.u.	–941.992461 a.u.	–941.994932 a.u.
$\varepsilon_0 + H_{corr}$	–941.983335 a.u.	–941.991755 a.u.	–941.994314 a.u.
$\varepsilon_0 + G_{corr}$	–942.053585 a.u.	–942.037089 a.u.	–942.031568 a.u.

N-Boc-2-phenylindoline Rotamer **2a-2**:

	Temperature		
	298 K	223 K	195 K
ε_0	–942.361634694 a.u.	–942.361634694 a.u.	–942.361634694 a.u.
ε_{ZPE}	0.359482 a.u.	0.359482 a.u.	0.359482 a.u.
E_{tot}	0.379391 a.u.	0.371197 a.u.	0.368722 a.u.
H_{corr}	0.380335 a.u.	0.371904 a.u.	0.369340 a.u.
G_{corr}	0.308748 a.u.	0.325578 a.u.	0.331224 a.u.
S_{tot}	150.668 calmol^{-1}K^{-1}	130.356 calmol^{-1}K^{-1}	122.657 calmol^{-1}K^{-1}
$\varepsilon_0 + \varepsilon_{ZPE}$	–942.002153 a.u.	–942.002153 a.u.	–942.002153 a.u.
$\varepsilon_0 + E_{tot}$	–941.982244 a.u.	–941.990438 a.u.	–941.992913 a.u.
$\varepsilon_0 + H_{corr}$	–941.981300 a.u.	–941.989731 a.u.	–941.992295 a.u.
$\varepsilon_0 + G_{corr}$	–942.052887 a.u.	–942.036057 a.u.	–942.030411 a.u.

TS for *N*-Boc-2-phenylindoline Clockwise Rotation of Boc Group from Rotamer **2a-1** to Rotamer **2a-2**:

	Temperature	
	298 K	195 K
ε_0	–942.339464824 a.u.	–942.339464824 a.u.
ε_{ZPE}	0.358800 a.u.	0.358800 a.u.
E_{tot}	0.377867 a.u.	0.367481 a.u.
H_{corr}	0.378811 a.u.	0.368098 a.u.
G_{corr}	0.311395 a.u.	0.332484 a.u.
S_{tot}	141.888 calmol^{-1}K^{-1}	114.607 calmol^{-1}K^{-1}
$\varepsilon_0 + \varepsilon_{ZPE}$	–941.980664 a.u.	–941.980665 a.u.
$\varepsilon_0 + E_{tot}$	–941.961598 a.u.	–941.971984 a.u.
$\varepsilon_0 + H_{corr}$	–941.960654 a.u.	–941.971367 a.u.
$\varepsilon_0 + G_{corr}$	–942.028069 a.u.	–942.006981 a.u.

TS for *N*-Boc-2-phenylindoline Anticlockwise Rotation of Boc Group from Rotamer **2a-1** to Rotamer **2a-2**:

	Temperature	
	298 K	195 K
ε_0	–942.341192722 a.u.	–942.341192722 a.u.
ε_{ZPE}	0.358718 a.u.	0.358718 a.u.
E_{tot}	0.377831 a.u.	0.367462 a.u.
H_{corr}	0.378776 a.u.	0.368079 a.u.
G_{corr}	0.309946 a.u.	0.331527 a.u.
S_{tot}	144.864 calmol^{-1}K^{-1}	117.625 calmol^{-1}K^{-1}
$\varepsilon_0 + \varepsilon_{ZPE}$	–941.982475 a.u.	–941.982475 a.u.
$\varepsilon_0 + E_{tot}$	–941.963361 a.u.	–941.973731 a.u.
$\varepsilon_0 + H_{corr}$	–941.962417 a.u.	–941.973114 a.u.
$\varepsilon_0 + G_{corr}$	–942.031247 a.u.	–942.009666 a.u.

N-Boc-2-(*p*-methoxyphenyl)indoline 2d-1

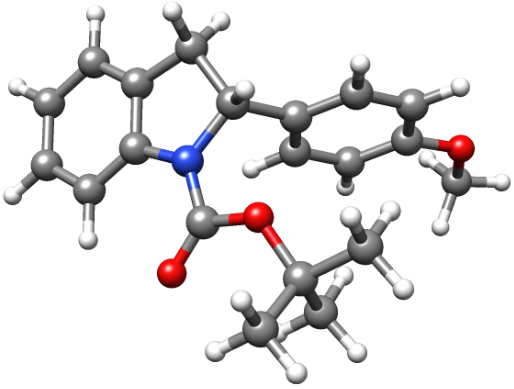

```
Route : # opt freq b3lyp scrf=(solvent=thf) geom=connectivity
          def2tzvp empiricaldispersion=gd3bj int=ultrafine
          pop=(regular,mk)
```

SMILES : COc3ccc(C2Cc1ccccc1N2C(=O)OC(C)(C)C)cc3

Formula : $C_{20}H_{23}NO_3$

Charge : 0

Multiplicity : 1

Dipole : 3.5230 Debye

Energy : -1056.94178703 a.u.

Gibbs Energy : -1056.60313300 a.u.

Number of imaginary frequencies : 0

Cartesian Coordinates (XYZ format)

47

C	2.76617300	-1.34184700	0.72738300
C	2.57360500	-0.20885300	-0.06744700
C	1.73475100	-1.38549800	1.82037300
C	0.71850700	-0.28064200	1.42319500
H	2.18725600	-1.15981700	2.78853900
H	1.24639300	-2.35526500	1.90458600
N	1.44379500	0.49582800	0.38416600
H	0.51056400	0.36971600	2.26856200
C	-0.58940800	-0.82517600	0.89832200
C	3.80863700	-2.20991100	0.46346700
C	4.67119300	-1.93838800	-0.59980500
C	4.47622200	-0.80340200	-1.37893300
C	3.42528400	0.07875700	-1.12820300
H	3.95459300	-3.08998500	1.07755500
H	5.49056200	-2.61084800	-0.81681100
H	5.14744500	-0.59669900	-2.20269700
H	3.26838700	0.95255100	-1.73801000
C	0.93834000	1.63738100	-0.17349600

O	-0.12747900	2.05054600	0.53021900
O	1.40523100	2.19204300	-1.15113700
C	-1.10487900	2.99964500	-0.02741700
C	-2.19345200	3.01474000	1.03779300
H	-2.61296400	2.01714200	1.16708000
H	-2.99133100	3.69568500	0.74072100
H	-1.78878100	3.35069600	1.99316900
C	-0.47543800	4.37880100	-0.18071400
H	-0.03821400	4.70094800	0.76554000
H	-1.24759100	5.09699300	-0.46133800
H	0.29773100	4.37596600	-0.94416100
C	-1.64899100	2.45254600	-1.34216800
H	-2.03237100	1.44148500	-1.19848700
H	-0.88094200	2.43458300	-2.11198800
H	-2.46792400	3.08674500	-1.68387700
C	-0.70407000	-1.32775300	-0.39190200
C	-1.71904700	-0.84064000	1.71518200
C	-1.90981900	-1.83294800	-0.86906400
C	-3.03069900	-1.84112000	-0.03997600
C	-2.92625500	-1.34179500	1.25997100
H	0.15193600	-1.31630500	-1.05421300
H	-1.96250400	-2.20694300	-1.88028400
O	-4.25659100	-2.30344800	-0.40869400
H	-1.65738500	-0.43801000	2.71908100
H	-3.80405000	-1.34303900	1.89256800
C	-4.41462000	-2.81032700	-1.72849000
H	-5.45413600	-3.11683400	-1.80914900
H	-3.76622400	-3.67285700	-1.90164100
H	-4.20251000	-2.04124700	-2.47545000

Frequencies

Mode	IR frequency	IR intensity	Raman intensity
1	22.38850000	0.13750000	0.00000000
2	25.75420000	0.22960000	0.00000000
3	33.74460000	0.45260000	0.00000000
4	41.26720000	1.34790000	0.00000000
5	54.51210000	4.71560000	0.00000000
6	82.01600000	2.16530000	0.00000000
7	86.29360000	1.34930000	0.00000000
8	106.30700000	2.62550000	0.00000000
9	130.22680000	0.40680000	0.00000000
10	149.35520000	0.80920000	0.00000000
11	171.44020000	1.34090000	0.00000000
12	205.94490000	0.11090000	0.00000000
13	211.97250000	2.40640000	0.00000000
14	223.47230000	7.04850000	0.00000000
15	233.01350000	2.58010000	0.00000000
16	246.36890000	1.71770000	0.00000000
17	252.83350000	3.91110000	0.00000000

18	264.69170000	2.68330000	0.00000000
19	275.20430000	0.35420000	0.00000000
20	292.71640000	1.03980000	0.00000000
21	324.38680000	0.29080000	0.00000000
22	330.77660000	15.14580000	0.00000000
23	353.78670000	3.09000000	0.00000000
24	383.63340000	0.59970000	0.00000000
25	406.90290000	4.64900000	0.00000000
26	426.28960000	0.40020000	0.00000000
27	434.21250000	5.12300000	0.00000000
28	442.73860000	1.59580000	0.00000000
29	463.07280000	7.01810000	0.00000000
30	472.89460000	3.42660000	0.00000000
31	479.19100000	1.38300000	0.00000000
32	516.64320000	1.97310000	0.00000000
33	538.57950000	18.85800000	0.00000000
34	563.03710000	9.39610000	0.00000000
35	571.89600000	21.67960000	0.00000000
36	624.48310000	21.53780000	0.00000000
37	634.02740000	11.64040000	0.00000000
38	652.01900000	1.01960000	0.00000000
39	718.83040000	4.98320000	0.00000000
40	748.46180000	36.70860000	0.00000000
41	752.32460000	17.75920000	0.00000000
42	756.10750000	19.42790000	0.00000000
43	766.03730000	46.13800000	0.00000000
44	776.58670000	24.33750000	0.00000000
45	793.66700000	16.73620000	0.00000000
46	820.40870000	10.10390000	0.00000000
47	829.46970000	0.96310000	0.00000000
48	836.74640000	10.31080000	0.00000000
49	852.82700000	72.51900000	0.00000000
50	857.92740000	65.50870000	0.00000000
51	870.07830000	6.55480000	0.00000000
52	880.04320000	5.18280000	0.00000000
53	931.44670000	0.16350000	0.00000000
54	931.59560000	0.38870000	0.00000000
55	939.41270000	1.31190000	0.00000000
56	959.20930000	0.03650000	0.00000000
57	969.04960000	0.32960000	0.00000000
58	974.12410000	0.06170000	0.00000000
59	977.60820000	0.04140000	0.00000000
60	986.90230000	10.56460000	0.00000000

61	1027.67120000	2.55900000	0.00000000
62	1033.01260000	11.43240000	0.00000000
63	1039.63300000	147.79710000	0.00000000
64	1051.96460000	20.84380000	0.00000000
65	1054.13450000	1.25070000	0.00000000
66	1055.81550000	80.03200000	0.00000000
67	1064.45280000	26.50120000	0.00000000
68	1108.98820000	32.92080000	0.00000000
69	1137.67590000	61.40770000	0.00000000
70	1156.77110000	278.02080000	0.00000000
71	1172.54530000	1.31330000	0.00000000
72	1177.44010000	10.55270000	0.00000000
73	1180.42370000	37.28640000	0.00000000
74	1188.61650000	318.38320000	0.00000000
75	1202.34780000	24.34560000	0.00000000
76	1202.69440000	54.84380000	0.00000000
77	1204.77170000	51.73620000	0.00000000
78	1234.96380000	13.64990000	0.00000000
79	1248.36890000	18.18880000	0.00000000
80	1270.08010000	49.26220000	0.00000000
81	1275.14360000	318.13730000	0.00000000
82	1276.37390000	114.92320000	0.00000000
83	1287.07850000	28.47140000	0.00000000
84	1307.86770000	214.79850000	0.00000000
85	1322.83970000	85.02090000	0.00000000
86	1338.48350000	60.82220000	0.00000000
87	1341.53540000	22.95060000	0.00000000
88	1354.63100000	82.44950000	0.00000000
89	1386.95740000	103.13190000	0.00000000
90	1398.62590000	24.88680000	0.00000000
91	1401.53280000	26.93280000	0.00000000
92	1415.02650000	648.05440000	0.00000000
93	1424.63990000	35.89780000	0.00000000
94	1460.72690000	9.70170000	0.00000000
95	1467.48320000	0.89480000	0.00000000
96	1477.55530000	11.25440000	0.00000000
97	1482.26660000	4.18530000	0.00000000
98	1484.84190000	1.77590000	0.00000000
99	1485.88330000	1.72620000	0.00000000
100	1487.64250000	10.75310000	0.00000000
101	1489.40300000	15.50270000	0.00000000
102	1497.61630000	2.75060000	0.00000000
103	1498.59000000	42.11030000	0.00000000

104	1500.51690000	41.01300000	0.00000000
105	1516.17110000	96.34410000	0.00000000
106	1518.50970000	165.74970000	0.00000000
107	1550.00130000	181.97740000	0.00000000
108	1623.22000000	25.29640000	0.00000000
109	1634.06770000	3.84850000	0.00000000
110	1642.81030000	49.74810000	0.00000000
111	1655.60720000	71.78750000	0.00000000
112	1716.67860000	591.06880000	0.00000000
113	3017.77150000	63.71560000	0.00000000
114	3041.88490000	14.20570000	0.00000000
115	3043.92340000	34.12990000	0.00000000
116	3044.38610000	37.60550000	0.00000000
117	3050.55940000	24.31210000	0.00000000
118	3076.62070000	45.31510000	0.00000000
119	3096.35780000	15.52360000	0.00000000
120	3102.51460000	14.87930000	0.00000000
121	3108.33850000	10.81220000	0.00000000
122	3108.94640000	27.68130000	0.00000000
123	3112.95470000	49.23220000	0.00000000
124	3119.95210000	48.90480000	0.00000000
125	3136.71100000	11.14510000	0.00000000
126	3141.72970000	28.64500000	0.00000000
127	3147.02830000	16.86820000	0.00000000
128	3167.41370000	15.41340000	0.00000000
129	3170.70750000	1.87680000	0.00000000
130	3178.67040000	23.08350000	0.00000000
131	3181.38170000	9.17650000	0.00000000
132	3193.11130000	12.37760000	0.00000000
133	3194.63210000	31.19640000	0.00000000
134	3217.15580000	6.85570000	0.00000000
135	3247.36770000	1.93250000	0.00000000

N-Boc-2-(*p*-methoxyphenyl)indoline 2d-2

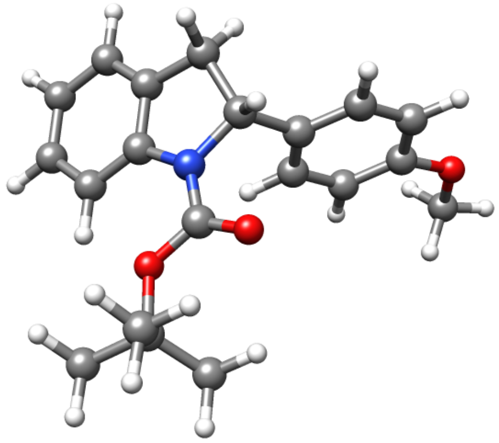

```
Route : # opt freq b3lyp scrf=(solvent=thf) geom=connectivity
          def2tzvp empiricaldispersion=gd3bj int=ultrafine
          pop=(regular,mk)
```

SMILES : COc3ccc(C2Cc1ccccc1N2C(=O)OC(C)(C)C)cc3

Formula : $C_{20}H_{23}NO_3$

Charge : 0

Multiplicity : 1

Dipole : 2.8016 Debye

Energy : -1056.93946076 a.u.

Gibbs Energy : -1056.60220000 a.u.

Number of imaginary frequencies : 0

Cartesian Coordinates (XYZ format)

47

C	-0.57218600	2.68601400	0.49891000
C	-1.26800800	1.57238300	0.01550600
C	0.31739300	2.27861000	1.64024200
C	0.34190800	0.73165800	1.54395700
H	-0.11125300	2.59795100	2.59353300
H	1.32088000	2.69498500	1.57210700
N	-0.84877400	0.42564600	0.72134600
H	0.21479300	0.27459900	2.52180800
C	1.61568000	0.20611900	0.92068400
C	-0.77755100	3.93572600	-0.05343100
C	-1.69192500	4.08010100	-1.09769500
C	-2.38336500	2.97039700	-1.56872800
C	-2.18262100	1.70210000	-1.02389000
H	-0.23400100	4.79347600	0.32299400
H	-1.86069500	5.05302700	-1.53979500
H	-3.09088700	3.08335200	-2.38020400
H	-2.71907600	0.85019900	-1.40381800

C	-1.33704100	-0.85253800	0.71847100
O	-2.38540200	-0.98209400	-0.10341100
O	-0.85085600	-1.73536900	1.40158300
C	-3.08382000	-2.27033700	-0.27289100
C	-4.16941900	-1.92420600	-1.28385900
H	-4.82167400	-1.14210900	-0.89428200
H	-4.77325200	-2.80732100	-1.49279600
H	-3.72780100	-1.57835300	-2.21904600
C	-2.12664800	-3.30896200	-0.84502700
H	-1.65865400	-2.93000500	-1.75475700
H	-2.68705400	-4.20961100	-1.09977200
H	-1.35140200	-3.56930600	-0.12958900
C	-3.69994300	-2.70735000	1.05049600
H	-4.32672000	-1.91182800	1.45615700
H	-2.93386800	-2.96209200	1.77783100
H	-4.32818500	-3.58295200	0.88072800
C	1.77468900	0.09364100	-0.45416500
C	2.69543400	-0.12921900	1.73899700
C	2.97524100	-0.33749900	-1.01344800
C	4.04548100	-0.66341800	-0.18307200
C	3.89637300	-0.55730800	1.20186500
H	0.95271200	0.33577200	-1.11458000
H	3.05883700	-0.41695200	-2.08681800
O	5.25863400	-1.09662000	-0.62408200
H	2.59173300	-0.06114700	2.81552100
H	4.72901400	-0.82454800	1.83909200
C	5.45490700	-1.22981700	-2.02637200
H	6.47492800	-1.58252600	-2.15416800
H	5.33493300	-0.27045200	-2.53605500
H	4.76162700	-1.95760100	-2.45553400

Frequencies

Mode	IR frequency	IR intensity	Raman intensity
1	20.63880000	0.24390000	0.00000000
2	21.91250000	0.71880000	0.00000000
3	32.17230000	0.76370000	0.00000000
4	36.14010000	0.05460000	0.00000000
5	60.74200000	2.19130000	0.00000000
6	80.29130000	1.99060000	0.00000000
7	94.44780000	1.04210000	0.00000000
8	98.31970000	2.90580000	0.00000000
9	122.37750000	1.31730000	0.00000000
10	142.58290000	0.91830000	0.00000000
11	175.77390000	2.23260000	0.00000000
12	207.83470000	0.19790000	0.00000000
13	212.13120000	4.07430000	0.00000000
14	224.57620000	3.49420000	0.00000000
15	233.79590000	2.45760000	0.00000000
16	244.48730000	0.25910000	0.00000000

17	251.66300000	2.17700000	0.00000000
18	274.28910000	3.31620000	0.00000000
19	275.82290000	0.30260000	0.00000000
20	302.50890000	3.62100000	0.00000000
21	327.61240000	5.83310000	0.00000000
22	337.87730000	4.81970000	0.00000000
23	353.03030000	2.32530000	0.00000000
24	362.04980000	11.67490000	0.00000000
25	414.80950000	2.06550000	0.00000000
26	425.94330000	0.75630000	0.00000000
27	433.20870000	3.70780000	0.00000000
28	443.91430000	9.23970000	0.00000000
29	460.63810000	4.41020000	0.00000000
30	473.55120000	5.81650000	0.00000000
31	481.60470000	1.74110000	0.00000000
32	509.95300000	10.57480000	0.00000000
33	538.16580000	17.88590000	0.00000000
34	562.88040000	10.34600000	0.00000000
35	568.27890000	20.67920000	0.00000000
36	619.87550000	10.60650000	0.00000000
37	650.08080000	3.55780000	0.00000000
38	666.38720000	18.99800000	0.00000000
39	715.37560000	3.69620000	0.00000000
40	734.40150000	5.76780000	0.00000000
41	748.10230000	37.17820000	0.00000000
42	758.31110000	41.50420000	0.00000000
43	767.55770000	35.54020000	0.00000000
44	774.25240000	21.84590000	0.00000000
45	795.48530000	5.15080000	0.00000000
46	822.63880000	2.42510000	0.00000000
47	826.79090000	2.01710000	0.00000000
48	836.51450000	8.48430000	0.00000000
49	848.42170000	48.63090000	0.00000000
50	854.46860000	26.26020000	0.00000000
51	875.49690000	45.31760000	0.00000000
52	879.94020000	45.09660000	0.00000000
53	931.12940000	0.05530000	0.00000000
54	931.76800000	0.50160000	0.00000000
55	940.50810000	0.68260000	0.00000000
56	955.06450000	0.33620000	0.00000000
57	963.91600000	0.02370000	0.00000000
58	972.23240000	0.02650000	0.00000000
59	973.92720000	0.07760000	0.00000000

60	984.38480000	14.12930000	0.00000000
61	1026.94130000	25.78030000	0.00000000
62	1032.59300000	7.79940000	0.00000000
63	1043.92350000	64.99610000	0.00000000
64	1054.03540000	1.49480000	0.00000000
65	1055.65130000	44.20640000	0.00000000
66	1056.60800000	40.30280000	0.00000000
67	1068.31250000	51.51790000	0.00000000
68	1109.13010000	32.05810000	0.00000000
69	1138.17370000	30.68310000	0.00000000
70	1151.30220000	196.41990000	0.00000000
71	1173.04230000	1.13460000	0.00000000
72	1179.04290000	55.58850000	0.00000000
73	1181.14260000	46.79400000	0.00000000
74	1189.71020000	253.08110000	0.00000000
75	1197.09160000	61.75730000	0.00000000
76	1201.88490000	49.70350000	0.00000000
77	1204.71220000	36.75840000	0.00000000
78	1232.93620000	7.49750000	0.00000000
79	1243.99260000	51.04030000	0.00000000
80	1270.36170000	27.33090000	0.00000000
81	1273.75850000	356.80000000	0.00000000
82	1278.94820000	72.25220000	0.00000000
83	1293.83320000	85.40820000	0.00000000
84	1303.12710000	173.47310000	0.00000000
85	1325.60210000	74.24270000	0.00000000
86	1340.40950000	9.64060000	0.00000000
87	1348.23660000	36.15530000	0.00000000
88	1362.88770000	174.05030000	0.00000000
89	1386.62930000	265.66240000	0.00000000
90	1398.54880000	26.51790000	0.00000000
91	1401.36760000	41.35700000	0.00000000
92	1408.29400000	376.59680000	0.00000000
93	1424.58580000	24.07550000	0.00000000
94	1459.39840000	5.66320000	0.00000000
95	1467.50640000	0.20850000	0.00000000
96	1477.49950000	11.18520000	0.00000000
97	1480.85870000	12.43850000	0.00000000
98	1484.69120000	1.92530000	0.00000000
99	1485.85330000	2.61690000	0.00000000
100	1487.51170000	11.01210000	0.00000000
101	1488.18720000	35.14580000	0.00000000
102	1498.01300000	3.09030000	0.00000000

103	1499.71760000	46.19930000	0.00000000
104	1500.36900000	38.91700000	0.00000000
105	1515.32830000	93.77040000	0.00000000
106	1518.46650000	179.34120000	0.00000000
107	1549.35210000	176.57950000	0.00000000
108	1622.31110000	22.31870000	0.00000000
109	1632.87440000	15.41000000	0.00000000
110	1645.60690000	82.11450000	0.00000000
111	1655.51430000	72.67160000	0.00000000
112	1708.36350000	791.93270000	0.00000000
113	3016.94180000	65.70510000	0.00000000
114	3038.52190000	38.53980000	0.00000000
115	3042.02480000	15.94830000	0.00000000
116	3044.48770000	36.39830000	0.00000000
117	3050.94810000	18.77860000	0.00000000
118	3075.50350000	45.54620000	0.00000000
119	3102.76520000	17.20700000	0.00000000
120	3103.93820000	6.05360000	0.00000000
121	3105.45760000	26.12340000	0.00000000
122	3110.46920000	19.46870000	0.00000000
123	3111.62020000	46.75220000	0.00000000
124	3118.59000000	63.63030000	0.00000000
125	3140.95150000	29.25970000	0.00000000
126	3144.88910000	0.85550000	0.00000000
127	3147.92140000	23.37600000	0.00000000
128	3166.09450000	15.94540000	0.00000000
129	3171.14870000	2.01350000	0.00000000
130	3178.87300000	22.22270000	0.00000000
131	3186.22380000	11.11920000	0.00000000
132	3193.01250000	11.57190000	0.00000000
133	3195.29270000	30.15320000	0.00000000
134	3216.68660000	7.59750000	0.00000000
135	3259.05750000	2.38350000	0.00000000

N-Boc-2-(*p*-methoxyphenyl)indoline Clockwise TS from 2d-1 to 2d-2

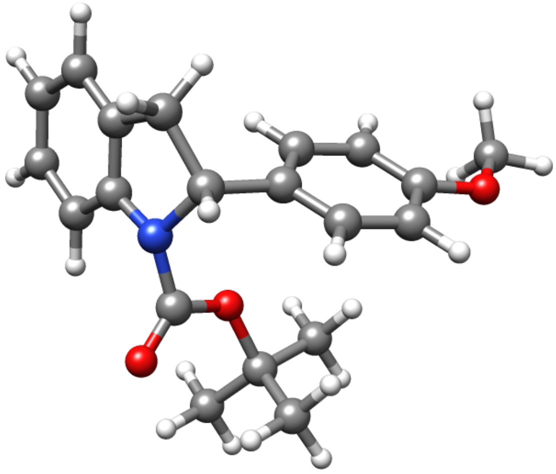

```
Route : # opt=qst3 freq b3lyp scrf=(solvent=thf)
          geom=connectivity def2tzvp empiricaldispersion=gd3bj
          int=ultrafine pop=(regular,mk)
```

SMILES : COc3ccc(C2Cc1ccccc1N2C(=O)OC(C)(C)C)cc3

Formula : $C_{20}H_{23}NO_3$

Charge : 0

Multiplicity : 1

Dipole : 4.8614 Debye

Energy : -1056.91794736 a.u.

Gibbs Energy : -1056.57782300 a.u.

Number of imaginary frequencies : 1

Cartesian Coordinates (XYZ format)

47

C	2.67147600	-1.60352400	0.35337100
C	2.49307200	-0.24172100	0.08475100
C	1.81457500	-1.97952400	1.53423700
C	0.82887800	-0.78795600	1.67552200
H	2.42640800	-2.06421300	2.43573500
H	1.28336600	-2.92099200	1.40566300
N	1.57984100	0.30769600	0.98225800
H	0.71654200	-0.50201000	2.72046200
C	-0.54550700	-1.04623900	1.10498200
C	3.54467300	-2.35097200	-0.40966200
C	4.24654700	-1.73407400	-1.45188400
C	4.06217100	-0.38011300	-1.70761300
C	3.18049900	0.38773600	-0.94453500
H	3.68280900	-3.40633400	-0.20802300
H	4.93036400	-2.31175900	-2.05922700
H	4.60504900	0.09170800	-2.51674400
H	3.03956700	1.43992000	-1.15442500

C	1.00906000	1.58707200	0.74913100
O	0.00714300	1.51287800	-0.11379800
O	1.42440500	2.58854000	1.27897300
C	-0.83516600	2.68004900	-0.47098400
C	-1.84922400	2.06741200	-1.42602600
H	-1.34758400	1.64347200	-2.29624500
H	-2.54506500	2.83528000	-1.76480500
H	-2.41069700	1.27686900	-0.92956500
C	-1.52004200	3.21224900	0.78056500
H	-2.06457600	2.41058200	1.28025100
H	-2.23489600	3.98415800	0.49309100
H	-0.80386100	3.64549900	1.47445600
C	0.01806400	3.72822200	-1.17173900
H	0.54860500	3.28204800	-2.01410900
H	0.74041900	4.17299500	-0.49201300
H	-0.63048200	4.51626700	-1.55677100
C	-0.72877400	-1.64874700	-0.13622400
C	-1.67749200	-0.63608000	1.80744700
C	-1.99639000	-1.82896500	-0.67506000
C	-3.11764000	-1.40111300	0.03744000
C	-2.95009000	-0.80743500	1.28866200
H	0.12876700	-1.97219500	-0.71166100
H	-2.09778400	-2.29335800	-1.64424500
O	-4.39891200	-1.51802400	-0.40565800
H	-1.56064600	-0.16223700	2.77492700
H	-3.82516900	-0.48246200	1.83568800
C	-4.61837000	-2.09633300	-1.68703400
H	-5.69382700	-2.08003200	-1.84238000
H	-4.26130600	-3.12850300	-1.72213300
H	-4.12790200	-1.51538000	-2.47195500

Frequencies

Mode	IR frequency	IR intensity	Raman intensity
1	-50.65380000	2.06750000	0.00000000
2	28.81450000	0.10070000	0.00000000
3	38.68030000	0.19880000	0.00000000
4	44.58180000	0.68580000	0.00000000
5	48.28470000	1.88630000	0.00000000
6	70.90780000	3.33030000	0.00000000
7	85.89820000	0.50590000	0.00000000
8	100.57400000	2.17470000	0.00000000
9	135.48250000	2.11680000	0.00000000
10	145.94870000	3.10960000	0.00000000
11	162.11750000	0.93610000	0.00000000
12	177.91550000	0.38950000	0.00000000
13	200.71940000	0.33260000	0.00000000
14	216.76090000	5.83150000	0.00000000
15	239.33490000	2.54320000	0.00000000
16	242.46680000	0.18330000	0.00000000

17	245.15190000	1.30540000	0.00000000
18	269.43440000	4.15050000	0.00000000
19	269.97850000	1.54130000	0.00000000
20	289.82960000	2.46770000	0.00000000
21	319.57840000	9.54090000	0.00000000
22	339.70490000	8.39180000	0.00000000
23	351.83270000	0.24030000	0.00000000
24	368.37240000	2.79830000	0.00000000
25	412.03670000	2.29850000	0.00000000
26	425.90300000	0.61350000	0.00000000
27	428.80260000	5.22410000	0.00000000
28	456.93530000	2.21380000	0.00000000
29	463.82250000	2.88180000	0.00000000
30	470.36440000	1.54150000	0.00000000
31	485.90740000	5.30990000	0.00000000
32	522.37180000	7.03100000	0.00000000
33	533.42250000	18.35920000	0.00000000
34	553.47770000	15.68180000	0.00000000
35	567.04460000	3.84490000	0.00000000
36	602.49950000	30.09610000	0.00000000
37	635.32450000	20.33710000	0.00000000
38	651.70010000	2.78120000	0.00000000
39	711.77980000	4.09300000	0.00000000
40	738.31120000	20.59560000	0.00000000
41	744.83290000	37.52580000	0.00000000
42	753.06510000	31.11840000	0.00000000
43	758.16640000	25.15390000	0.00000000
44	771.73010000	21.89360000	0.00000000
45	812.84540000	9.78190000	0.00000000
46	828.30410000	19.79070000	0.00000000
47	830.74610000	12.92370000	0.00000000
48	837.02380000	24.43600000	0.00000000
49	848.18490000	94.84150000	0.00000000
50	852.13480000	16.24820000	0.00000000
51	857.75490000	14.22440000	0.00000000
52	874.41040000	18.41810000	0.00000000
53	926.72400000	2.73720000	0.00000000
54	933.33170000	0.01870000	0.00000000
55	933.66530000	0.10090000	0.00000000
56	958.87310000	0.17330000	0.00000000
57	960.40710000	2.92920000	0.00000000
58	970.88850000	0.25880000	0.00000000
59	975.70090000	0.12560000	0.00000000

60	981.34710000	20.76230000	0.00000000
61	1014.57080000	0.66250000	0.00000000
62	1022.60180000	25.63460000	0.00000000
63	1035.18980000	4.54800000	0.00000000
64	1048.54720000	17.63740000	0.00000000
65	1053.69730000	0.77410000	0.00000000
66	1055.85680000	81.08670000	0.00000000
67	1060.48830000	24.58180000	0.00000000
68	1085.40590000	75.52520000	0.00000000
69	1136.52560000	61.67370000	0.00000000
70	1140.83100000	21.03850000	0.00000000
71	1172.04790000	499.74350000	0.00000000
72	1172.37380000	3.67250000	0.00000000
73	1176.29430000	5.62540000	0.00000000
74	1179.20090000	43.85800000	0.00000000
75	1192.40890000	8.41430000	0.00000000
76	1203.47000000	22.27560000	0.00000000
77	1204.62080000	99.66090000	0.00000000
78	1234.76270000	25.97350000	0.00000000
79	1250.78510000	42.55090000	0.00000000
80	1265.93000000	211.01670000	0.00000000
81	1274.05100000	41.56400000	0.00000000
82	1277.39370000	415.86190000	0.00000000
83	1284.44670000	32.13460000	0.00000000
84	1290.64130000	243.64270000	0.00000000
85	1321.72310000	78.67470000	0.00000000
86	1328.53210000	65.91020000	0.00000000
87	1341.27670000	0.93700000	0.00000000
88	1353.13670000	33.19470000	0.00000000
89	1387.32250000	193.50810000	0.00000000
90	1392.87140000	19.25480000	0.00000000
91	1399.14930000	23.94080000	0.00000000
92	1403.12250000	27.76420000	0.00000000
93	1425.72350000	18.08610000	0.00000000
94	1463.98330000	8.20900000	0.00000000
95	1467.75740000	0.09580000	0.00000000
96	1477.57710000	11.27850000	0.00000000
97	1480.56060000	6.18120000	0.00000000
98	1484.86120000	3.73500000	0.00000000
99	1485.67590000	0.41290000	0.00000000
100	1488.23800000	10.66070000	0.00000000
101	1489.91610000	19.74760000	0.00000000
102	1494.93450000	47.50760000	0.00000000

103	1496.70200000	3.21140000	0.00000000
104	1501.01280000	41.75670000	0.00000000
105	1516.56570000	14.10540000	0.00000000
106	1517.70440000	198.27840000	0.00000000
107	1550.03110000	169.23340000	0.00000000
108	1621.81490000	23.68390000	0.00000000
109	1632.01280000	2.49140000	0.00000000
110	1643.84970000	111.66220000	0.00000000
111	1653.69630000	97.68680000	0.00000000
112	1751.25740000	634.43970000	0.00000000
113	3018.56780000	64.08660000	0.00000000
114	3038.85030000	32.69050000	0.00000000
115	3045.09330000	11.95070000	0.00000000
116	3046.75520000	30.46770000	0.00000000
117	3053.43940000	21.36650000	0.00000000
118	3077.93980000	45.35820000	0.00000000
119	3080.14430000	51.46230000	0.00000000
120	3103.49330000	18.01200000	0.00000000
121	3106.20240000	12.19250000	0.00000000
122	3110.85700000	13.61930000	0.00000000
123	3116.87780000	53.64360000	0.00000000
124	3125.87820000	25.14640000	0.00000000
125	3139.84940000	1.58520000	0.00000000
126	3142.09350000	28.84220000	0.00000000
127	3142.82210000	24.40450000	0.00000000
128	3165.31510000	15.20000000	0.00000000
129	3168.00720000	7.13170000	0.00000000
130	3174.44340000	14.30200000	0.00000000
131	3182.62330000	8.78130000	0.00000000
132	3187.51330000	28.72470000	0.00000000
133	3192.94130000	11.95860000	0.00000000
134	3196.79560000	21.17980000	0.00000000
135	3216.91590000	7.27140000	0.00000000

N-Boc-2-(*p*-methoxyphenyl)indoline Anticlockwise TS from 2d-1 to 2d-2

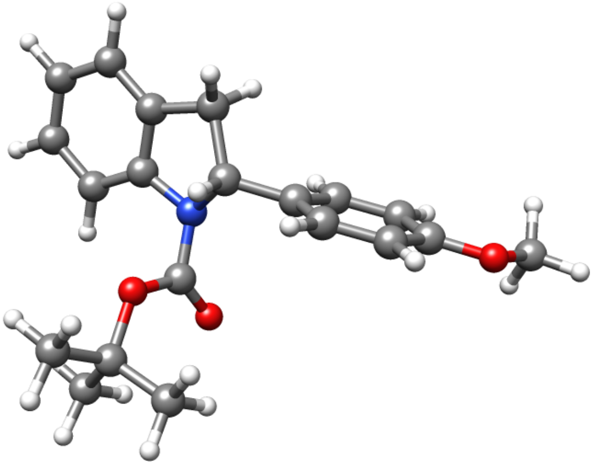

Route : # opt=qst3 freq b3lyp scrf=(solvent=thf)
geom=connectivity def2tzvp empiricaldispersion=gd3bj
int=ultrafine pop=(regular,mk)

SMILES : COc3ccc(C2Cc1ccccc1N2C(=O)OC(C)(C)C)cc3

Formula : $C_{20}H_{23}NO_3$

Charge : 0

Multiplicity : 1

Dipole : 1.8551 Debye

Energy : -1056.91935219 a.u.

Gibbs Energy : -1056.58091100 a.u.

Number of imaginary frequencies : 1

Cartesian Coordinates (XYZ format)

47

C	-1.75320500	2.41423800	0.48531600
C	-1.95946500	1.31274600	-0.35075700
C	-0.34880100	2.35119600	1.02441400
C	0.04780000	0.87978400	0.76734000
H	-0.26295200	2.60558200	2.07969600
H	0.30950800	3.02172100	0.46463600
N	-0.79772300	0.52417800	-0.41328200
H	-0.28782200	0.28032800	1.62127400
C	1.50948400	0.61901100	0.53980100
C	-2.75227900	3.35421900	0.65027900
C	-3.96887500	3.18321900	-0.01693100
C	-4.16237300	2.08186500	-0.84427600
C	-3.15823900	1.13019500	-1.02506100
H	-2.59837800	4.21069400	1.29527700
H	-4.76225700	3.90719400	0.11285900

H	-5.10798600	1.95427100	-1.35549300
H	-3.31659500	0.27624200	-1.67053300
C	-0.90763200	-0.86893800	-0.69746700
O	-1.64096800	-1.47860200	0.22828400
O	-0.37549800	-1.38606800	-1.64547400
C	-1.89485800	-2.94043700	0.20957600
C	-2.75571300	-3.13914200	1.44831200
H	-2.21748900	-2.82719300	2.34372100
H	-3.01669600	-4.19262200	1.54961800
H	-3.67537800	-2.55847700	1.37332700
C	-2.66159000	-3.30935200	-1.05314400
H	-3.56590100	-2.70522100	-1.13728100
H	-2.95719600	-4.35768800	-0.99583400
H	-2.05449100	-3.16835600	-1.94364500
C	-0.57203600	-3.68391600	0.33705700
H	-0.02330600	-3.33280100	1.21201900
H	0.04710900	-3.55073800	-0.54629800
H	-0.77275300	-4.74831200	0.46492900
C	2.16794500	1.10828300	-0.58377700
C	2.24615500	-0.11639600	1.46761300
C	3.52420000	0.88343100	-0.78404600
C	4.24957200	0.15280800	0.15912400
C	3.60036100	-0.34685800	1.28869300
H	1.61078500	1.66151600	-1.32857900
H	4.00012400	1.27256300	-1.67136300
O	5.57750500	-0.12373700	0.05843100
H	1.75115500	-0.51741000	2.34403800
H	4.16962200	-0.91866400	2.00941900
C	6.28150100	0.35429500	-1.08207400
H	7.30913100	0.02260900	-0.95975400
H	6.25493000	1.44547100	-1.13476500
H	5.87087000	-0.06420300	-2.00418100

Frequencies

Mode	IR frequency	IR intensity	Raman intensity
1	-30.87700000	1.64890000	0.00000000
2	20.54860000	0.38590000	0.00000000
3	24.64960000	1.57020000	0.00000000
4	41.21990000	0.64810000	0.00000000
5	45.69620000	1.03740000	0.00000000
6	61.78080000	1.41050000	0.00000000
7	93.81540000	2.72100000	0.00000000
8	99.76590000	1.61050000	0.00000000
9	139.32060000	3.15310000	0.00000000
10	142.32420000	2.16180000	0.00000000
11	167.24690000	1.82840000	0.00000000
12	174.61750000	1.01680000	0.00000000
13	202.83570000	0.08420000	0.00000000
14	210.92380000	4.78260000	0.00000000
15	240.19110000	3.83760000	0.00000000

16	245.56150000	1.16650000	0.00000000
17	248.51160000	2.63120000	0.00000000
18	261.31880000	3.54140000	0.00000000
19	269.75570000	0.09680000	0.00000000
20	283.67140000	2.26840000	0.00000000
21	320.49020000	8.76010000	0.00000000
22	341.89090000	1.38940000	0.00000000
23	355.86290000	1.39760000	0.00000000
24	376.67290000	3.87820000	0.00000000
25	409.06920000	2.16530000	0.00000000
26	428.11020000	0.66510000	0.00000000
27	432.18570000	8.13690000	0.00000000
28	459.46090000	3.43430000	0.00000000
29	468.05570000	3.63320000	0.00000000
30	475.39910000	2.14880000	0.00000000
31	488.77140000	4.31080000	0.00000000
32	520.16450000	12.11710000	0.00000000
33	541.30720000	11.03120000	0.00000000
34	557.09980000	22.63670000	0.00000000
35	562.75730000	33.18760000	0.00000000
36	614.01030000	6.79050000	0.00000000
37	624.01930000	14.70110000	0.00000000
38	650.25870000	2.05370000	0.00000000
39	688.07710000	4.03650000	0.00000000
40	737.47940000	17.91500000	0.00000000
41	749.52640000	24.01050000	0.00000000
42	755.11160000	44.05970000	0.00000000
43	764.75200000	21.57840000	0.00000000
44	781.10650000	19.42730000	0.00000000
45	803.72390000	15.23370000	0.00000000
46	828.47320000	1.62890000	0.00000000
47	834.33990000	14.48990000	0.00000000
48	843.43100000	6.94970000	0.00000000
49	850.88540000	127.22840000	0.00000000
50	854.70230000	30.41080000	0.00000000
51	868.82580000	13.51570000	0.00000000
52	891.32820000	5.54830000	0.00000000
53	932.53870000	1.97290000	0.00000000
54	933.03140000	4.14790000	0.00000000
55	933.68480000	0.09620000	0.00000000
56	960.19660000	0.20250000	0.00000000
57	964.29460000	0.10970000	0.00000000
58	967.67950000	24.32070000	0.00000000

59	970.65040000	20.61200000	0.00000000
60	975.42060000	0.11940000	0.00000000
61	1013.87180000	51.60700000	0.00000000
62	1032.03520000	0.42980000	0.00000000
63	1043.47600000	11.97600000	0.00000000
64	1049.73020000	47.25190000	0.00000000
65	1054.40280000	1.91620000	0.00000000
66	1056.22630000	25.73890000	0.00000000
67	1057.12930000	56.50060000	0.00000000
68	1076.37890000	40.69060000	0.00000000
69	1132.99100000	41.87920000	0.00000000
70	1138.41340000	17.24980000	0.00000000
71	1164.30360000	730.32940000	0.00000000
72	1172.69860000	1.21400000	0.00000000
73	1178.13520000	7.51270000	0.00000000
74	1185.36230000	32.89620000	0.00000000
75	1197.95030000	48.99390000	0.00000000
76	1203.66050000	22.67830000	0.00000000
77	1205.63830000	9.80690000	0.00000000
78	1241.11520000	20.94260000	0.00000000
79	1248.36950000	250.13730000	0.00000000
80	1263.48990000	61.96870000	0.00000000
81	1272.74470000	25.63480000	0.00000000
82	1276.82520000	349.83560000	0.00000000
83	1282.70790000	61.60970000	0.00000000
84	1294.71520000	290.88700000	0.00000000
85	1315.62980000	82.38810000	0.00000000
86	1337.33290000	17.45590000	0.00000000
87	1339.81650000	7.42160000	0.00000000
88	1348.83880000	13.09490000	0.00000000
89	1376.94920000	69.82040000	0.00000000
90	1400.89650000	21.87930000	0.00000000
91	1401.89310000	18.88300000	0.00000000
92	1403.58290000	23.97580000	0.00000000
93	1426.90390000	17.01560000	0.00000000
94	1462.46510000	9.83710000	0.00000000
95	1468.19010000	0.35160000	0.00000000
96	1477.52050000	10.93700000	0.00000000
97	1479.56840000	3.12300000	0.00000000
98	1483.66350000	0.22460000	0.00000000
99	1484.83380000	2.04870000	0.00000000
100	1488.04940000	10.89190000	0.00000000
101	1490.42130000	21.89390000	0.00000000

102	1496.30890000	41.03600000	0.00000000
103	1497.30230000	2.94950000	0.00000000
104	1501.03770000	45.04210000	0.00000000
105	1515.94210000	172.16120000	0.00000000
106	1516.84000000	19.16940000	0.00000000
107	1550.42400000	170.20470000	0.00000000
108	1622.14480000	17.47130000	0.00000000
109	1632.79070000	11.89020000	0.00000000
110	1646.09320000	89.23490000	0.00000000
111	1654.73580000	90.71640000	0.00000000
112	1767.12690000	545.73070000	0.00000000
113	2999.63760000	41.79730000	0.00000000
114	3019.15860000	66.95780000	0.00000000
115	3033.41070000	36.77960000	0.00000000
116	3044.06270000	14.46770000	0.00000000
117	3046.55930000	33.42890000	0.00000000
118	3052.73660000	14.86790000	0.00000000
119	3078.67980000	44.07980000	0.00000000
120	3103.13890000	20.56290000	0.00000000
121	3106.99780000	5.67920000	0.00000000
122	3108.67340000	21.54350000	0.00000000
123	3114.44110000	42.23580000	0.00000000
124	3120.03440000	54.74500000	0.00000000
125	3142.11480000	28.73390000	0.00000000
126	3142.57470000	0.61150000	0.00000000
127	3145.46000000	24.61330000	0.00000000
128	3167.42290000	15.07610000	0.00000000
129	3169.23250000	5.40890000	0.00000000
130	3175.65150000	11.43830000	0.00000000
131	3183.51720000	11.46950000	0.00000000
132	3187.42120000	30.99140000	0.00000000
133	3194.22860000	10.88470000	0.00000000
134	3196.88990000	23.48930000	0.00000000
135	3217.59800000	7.49350000	0.00000000

Thermochemistry Data for *N*-Boc-2-(*p*-methoxyphenyl)indoline Calculations

N-Boc-2-(*p*-methoxyphenyl)indoline Rotamer **2d-1**:

	Temperature		
	298 K	223 K	195 K
ε_0	–1056.94178703 a.u.	–1056.94178703 a.u.	–1056.94178703 a.u.
ε_{ZPE}	0.391930 a.u.	0.391930 a.u.	0.391930 a.u.
E_{tot}	0.414398 a.u.	0.405188 a.u.	0.402377 a.u.
H_{corr}	0.415342 a.u.	0.405894 a.u.	0.402995 a.u.
G_{corr}	0.338654 a.u.	0.356627 a.u.	0.362619 a.u.
S_{tot}	161.403 calmol^{-1}K^{-1}	138.635 calmol^{-1}K^{-1}	129.928 calmol^{-1}K^{-1}
$\varepsilon_0 + \varepsilon_{ZPE}$	–1056.549857 a.u.	–1056.549857 a.u.	–1056.549857 a.u.
$\varepsilon_0 + E_{tot}$	–1056.527389 a.u.	–1056.536599 a.u.	–1056.539410 a.u.
$\varepsilon_0 + H_{corr}$	–1056.526445 a.u.	–1056.535893 a.u.	–1056.538792 a.u.
$\varepsilon_0 + G_{corr}$	–1056.603133 a.u.	–1056.585160 a.u.	–1056.579168 a.u.

N-Boc-2-(*p*-methoxyphenyl)indoline Rotamer **2d-2**:

	Temperature		
	298 K	223 K	195 K
ε_0	–1056.93946076 a.u.	–1056.93946076 a.u.	–1056.93946076 a.u.
ε_{ZPE}	0.391877 a.u.	0.391877 a.u.	0.391877 a.u.
E_{tot}	0.414403 a.u.	0.405192 a.u.	0.402381 a.u.
H_{corr}	0.415347 a.u.	0.405898 a.u.	0.402999 a.u.
G_{corr}	0.337261 a.u.	0.355586 a.u.	0.361709 a.u.
S_{tot}	164.347 calmol^{-1}K^{-1}	141.577 calmol^{-1}K^{-1}	132.870 calmol^{-1}K^{-1}
$\varepsilon_0 + \varepsilon_{ZPE}$	–1056.547584 a.u.	–1056.547584 a.u.	–1056.547584 a.u.
$\varepsilon_0 + E_{tot}$	–1056.525058 a.u.	–1056.534269 a.u.	–1056.537080 a.u.
$\varepsilon_0 + H_{corr}$	–1056.524114 a.u.	–1056.533563 a.u.	–1056.536462 a.u.
$\varepsilon_0 + G_{corr}$	–1056.602200 a.u.	–1056.583875 a.u.	–1056.577752 a.u.

TS for *N*-Boc-2-(*p*-methoxyphenyl)indoline Clockwise Rotation of Boc Group from Rotamer **2d-1** to Rotamer **2d-2**:

	Temperature	
	298 K	195 K
ε_0	–1056.91794736 a.u.	–1056.91794736 a.u.
ε_{ZPE}	0.391063 a.u.	0.391063 a.u.
E_{tot}	0.412781 a.u.	0.401029 a.u.
H_{corr}	0.413725 a.u.	0.401646 a.u.
G_{corr}	0.340125 a.u.	0.363074 a.u.
S_{tot}	154.905 calmol^{-1}K^{-1}	124.124 calmol^{-1}K^{-1}
$\varepsilon_0 + \varepsilon_{ZPE}$	–1056.526885 a.u.	–1056.526884 a.u.
$\varepsilon_0 + E_{tot}$	–1056.505167 a.u.	–1056.516918 a.u.
$\varepsilon_0 + H_{corr}$	–1056.504222 a.u.	–1056.516301 a.u.
$\varepsilon_0 + G_{corr}$	–1056.577823 a.u.	–1056.554873 a.u.

TS for *N*-Boc-2-(*p*-methoxyphenyl)indoline Anticlockwise Rotation of Boc Group from Rotamer **2d-1** to Rotamer **2d-2**:

	Temperature	
	298 K	195 K
ε_0	–1056.91935219 a.u.	–1056.91935219 a.u.
ε_{ZPE}	0.390957 a.u.	0.390957 a.u.
E_{tot}	0.412743 a.u.	0.401005 a.u.
H_{corr}	0.413687 a.u.	0.401623 a.u.
G_{corr}	0.338442 a.u.	0.361963 a.u.
S_{tot}	158.367 calmol^{-1}K^{-1}	127.623 calmol^{-1}K^{-1}
$\varepsilon_0 + \varepsilon_{ZPE}$	–1056.528396 a.u.	–1056.528395 a.u.
$\varepsilon_0 + E_{tot}$	–1056.506609 a.u.	–1056.518347 a.u.
$\varepsilon_0 + H_{corr}$	–1056.505665 a.u.	–1056.517729 a.u.
$\varepsilon_0 + G_{corr}$	–1056.580911 a.u.	–1056.557389 a.u.
